# Gut microbial community of patients with Parkinson’s disease analyzed using metagenome-assembled genomes

**DOI:** 10.4103/NRR.NRR-D-25-00420

**Published:** 2025-09-29

**Authors:** Yi Zhang, Chengjun Mo, Xiaoqin He, Qin Xiao, Xiaodong Yang

**Affiliations:** 1 Department of Neurology and Institute of Neurology, Ruijin Hospital, Shanghai Jiao Tong University School of Medicine, Shanghai, China; 2 Department of General Practice, Ruijin Hospital, Shanghai Jiao Tong University School of Medicine, Shanghai, China

**Keywords:** constipation, dysbiosis, gut microbiota, metagenome-assembled genomes, metagenomic binning, nerve regeneration, Parkinson’s disease, shotgun metagenomic sequencing, α-synuclein

## Abstract

Previous investigations into gut microbiota dysbiosis in patients with Parkinson’s disease have relied on 16S rRNA amplicon sequencing and assembly-free metagenomic approaches. However, there is an urgent need to study the function of the gut microbiome at the genome level using metagenome-assembled genomes. Here, we conducted single-sample metagenomic binning analysis using shotgun metagenomic sequencing data and retrieved 2837 metagenome-assembled genomes to explore the gut microbiota profile at the genome level. Reconstructing microbial genomes from metagenomic sequences greatly enriched the diversity and number of microbial genomes, especially those of uncultivable strains. By integrating the analysis of metagenome-assembled genomes with clinical parameters, we observed higher α-diversity indexes and a very different composition of microbial communities in patients with Parkinson’s disease. We also identified microbial species and metagenome-assembled genomes that were significantly associated with clinical characteristics, including disease severity, medication, motor complications, and non-motor symptoms. The genes of Parkinson’s disease severity-associated metagenome-assembled genomes were distributed across multiple pathways, such as carbon metabolism, phosphonate metabolism, carbohydrate metabolism, amino acid metabolism, fatty acid metabolism, bile acid metabolism, metabolism of cofactors and vitamins, neuroprotective molecules, immunogenic components, toxic metabolites, translation, and bacterial secretion. Our work provides a comprehensive resource for investigating the gut microbiota–Parkinson’s disease relationship at the genome level, which may enhance our comprehension of the underlying mechanisms of this disease.

## Graphical abstract

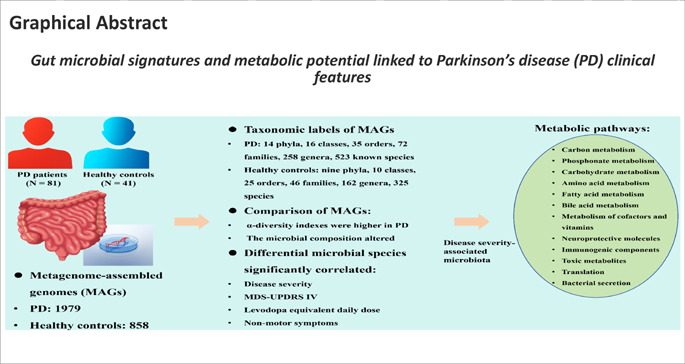

## Introduction

Parkinson’s disease (PD) presents both motor and non-motor symptoms (Dorsey et al., 2018). Constipation is one of the most common non-motor symptoms in PD (Knudsen et al., 2017). Importantly, constipation can occur over 10 years prior to a PD diagnosis, and might be a risk factor for PD development (Adams-Carr et al., 2016). Research has revealed the existence of pathological hallmarks of PD in colonic biopsies from patients with PD but not controls (Lebouvier et al., 2010). Additionally, α-synuclein accumulation has been detected in colonic biopsies performed nearly a decade prior to the emergence of motor symptoms (Shannon et al., 2012; Hilton et al., 2014). In a PD mouse model, gastrointestinal dysfunction reportedly precedes motor deficits, and increased α-synuclein protein expression appears in the colon before it occurs in the brain (Yang et al., 2018). This early gastrointestinal involvement supports the “ascending anatomical theory,” which suggests that PD pathology may progress from the gut to the brain (Braak et al., 2003).

The gut microbiota is currently recognized as a key player for regulating physical health (Heintz-Buschart and Wilmes, 2018). In recent years, growing numbers of studies have explored how the gut microbiota affects brain function. Increasing evidence points to the gut microbiota as a risk factor for various neurological disorders (Cryan et al., 2020). Notably, accumulating evidence connects the gut microbiota to the symptomatology and pathophysiology of PD (dos Santos et al., 2023; Fang et al., 2024; Neufeld et al., 2024). To examine gut microbiota dysbiosis in patients with PD, 16S rRNA amplicon sequencing has been widely used (Scheperjans et al., 2015; Hill-Burns et al., 2017; Heintz-Buschart et al., 2018); however, this method has several limitations. It is prone to bias, has limited resolution, and lacks functional information. These drawbacks restrict the universality and significance of related studies, preventing the discovery of more PD-associated gut microbiome features. Additionally, it is challenging to assign abundance changes of different genes detected through 16S rRNA amplicon sequencing to specific microbial species or strains. Although high-resolution shotgun metagenomic sequencing has been gradually adopted to infer the biological functions of microbial communities (Bedarf et al., 2017; Qian et al., 2020; Wallen et al., 2022; Palacios et al., 2023), this method often generates misassemblies and chimeric contigs, introducing substantial biases into the analysis (Teeling and Glöckner, 2012; Howe and Chain, 2015). Assembly-free-based metagenomic approaches can profile low-abundance microorganisms that cannot be assembled because of low sequence coverage (Quince et al., 2017). Nevertheless, it remains challenging to identify the characteristics of these unrecognized microorganisms. There is therefore an urgent need to explore gut microbial functions at the genome level.

Bacterial genomes have traditionally been obtained through conventional culturing methods; however, culturing gut microbiota in artificial culture media remains difficult. By contrast, metagenomic binning offers a powerful approach for reconstructing the genomes of rare community members (Wang et al., 2024). Metagenome-assembled genomes (MAGs), which are generated from metagenomic data as draft microbial genomes, have been extensively used to investigate microbiota characteristics in human diseases (Zhu et al., 2021; Hu et al., 2023; Yang et al., 2023). Nonetheless, the application of gut microbiota MAGs of patients with PD remains scarce.

Here, we performed metagenomic binning analysis to retrieve MAGs based on shotgun metagenomic sequencing data. We sought to explore the gut microbiota profile at the genome level to achieve a detailed understanding of the role that the gut microbiota plays in PD development.

## Methods

### Study design and patient recruitment

This case–control study was conducted in Ruijin Hospital. All subjects were recruited from the movement disorder clinic within the Neurology Department from March 2021 to February 2023. The inclusion criterion was patients with primary PD, identified using the diagnostic guidelines of the United Kingdom PD Society Brain Bank criteria (Daniel and Lees, 1993). Exclusion criteria were as follows: (1) secondary parkinsonism; (2) history of deep brain stimulation or ablative or lesioning procedures; (3) any chronic health conditions; or (4) recent use of antibiotics, probiotics, or proton pump inhibitors within the last 3 months. Healthy controls were recruited through advertisement during an identical timeframe. Inclusion criteria were as follows: (1) no abnormalities detected in a standard physical examination; (2) an absence of gastrointestinal symptoms or conditions; and (3) no history or diagnosis of any neurodegenerative disorder. The exclusion criteria for control subjects were the same as those for patients with PD. The study was conducted in accordance with the tenets of the *Declaration of Helsinki*. Ethical approval for the study was obtained from the Research Ethics Committee at Ruijin Hospital (approval No. 2019226) on December 27, 2019. Each participant was thoroughly informed about the study’s objectives and provided their written consent to partake.

### Clinical data collection

Information about participants’ age, sex, and body mass index was collected. Clinical features were also noted, including disease duration, Hoehn and Yahr (H&Y) stage (Goetz et al., 2004), and Movement Disorder Society Unified PD Rating Scale (MDS-UPDRS) score (Goetz et al., 2008). Levodopa equivalent daily dose (LEDD) was calculated (Tomlinson et al., 2010), and non-motor symptoms, including scores from the Non-Motor Symptom Scale (van Wamelen et al., 2021), 39-item PD questionnaire (PDQ-39) (Suratos et al., 2018), Hamilton Anxiety Rating Scale (Hamilton, 1959), PD Sleep Scale 2 (Trenkwalder et al., 2011), Mini-Mental State Examination (Mossello and Boncinelli, 2006), and Montreal Cognitive Assessment (Nasreddine et al., 2005), were recorded.

### Biomaterial collection, genetic material extraction, and shotgun metagenomic sequencing

Fecal samples were collected and total genomic DNA was extracted. The extraction process strictly adhered to the manufacturer’s instructions (PF Mag-Bind Stool DNA Kit, Omega Bio-Tek, Georgia, USA). Subsequently, the extracted DNA was fragmented to an average size of approximately 400 bp for the construction of paired-end libraries. Paired-end sequencing was then performed.

### Metagenomic assembly and binning, prediction of taxonomy label, and phylogenetic analysis

Quality control and sequence assembly were performed. Briefly, the paired-end Illumina reads were processed using fastp (version 0.20.1) (Chen et al., 2018). The reads were then quality-filtered, trimmed, and screened using SeqPrep v1.1 (https://github.com/jstjohn/SeqPrep) (John, 2011) and Sickle (https://github.com/najoshi/sickle) (Joshi et al., 2011). Metagenomic data were assembled using MEGAHIT (version 1.1.2) (Li et al., 2015). Binning was conducted, and data were further integrated to generate non-redundant bins. The completeness and contamination of these bins were estimated using CheckM (Parks et al., 2015). An estimated completeness of MAG > 50% and an estimated contamination < 10% was considered a medium-quality draft genome, and an estimated completeness of MAG > 90% and an estimated contamination < 5% was considered a high-quality draft genome (Bowers et al., 2017).

Taxonomic annotation was performed using the Genome Taxonomy Database (GTDB, https://gtdb.ecogenomic.org) with GTDB-Tk (Parks et al., 2022). For a recovered genome (bin) annotated at the genus level, average nucleotide identity (ANI) analysis was conducted with reference to the reference genome using fastANI (version 1.0) (Jain et al., 2018), to further classify the bin at the species level. When the ANI between two bacteria was ≤ 99%, it was generally regarded as a new species. Using the reference genome, the core genes of bins were predicted with BLASTP (Chaumeil et al., 2020). Subsequently, evolutionary analysis was performed using MEGA (version 7) (Kumar et al., 2016), Mafft (version 7) (Katoh et al., 2018), trimAl (version 7) (Capella-Gutiérrez et al., 2009), RAxML (version 8) (Stamatakis, 2014), and IQ-TREE software. This analysis was based on the predicted 16S rRNA or core genes, and aimed to explore the evolutionary relationships between the bins, and between the bins and the reference genomes.

### Species-level clustering of metagenome-assembled genomes

MAGs were grouped into species-level genome clusters, which were termed species-level genome bins (SGBs), using the ‘cluster’ program in dRep (v2.2.3) (Olm et al., 2017) with a 95% ANI cutoff. SGBs were classified as known if they included at least one reference genome or a genome assembled from metagenomes present in the GTDB. SGBs that lacked reference genomes were categorized as unknown SGBs (uSGBs).

### Microbiota data analysis

Analyses of microbial α- and β-diversity were performed at the species level using the vegan package in R software (version 4.0.3, Vienna, Austria) (R Core Team, 2024). Principal coordinate analysis plots were created based on Bray–Curtis dissimilarity, and differences in microbial community structures were assessed via permutational multivariate analysis of variance with 9999 permutations using the “adonis” function in the vegan package (Oksanen et al., 2019).

### Machine-learning approach for disease condition and severity classification

We used the python scikit-learn (v1.5.1) (Pedregosa et al., 2011) to implement the random forest algorithm in each classification task. The prediction performance of random forest models was evaluated using a five-fold cross validation approach.

### Functional annotation of metagenome-assembled genomes

The functional annotation for the coding sequence of bins was performed using Diamond (http://www.diamondsearch.org/index.php, version 0.8.35) (Buchfink et al., 2014) against the Kyoto Encyclopedia of Genes and Genomes (KEGG) database (http://www.genome.jp/kegg/, version 202209) with an *e*-value cutoff of 1*e*^–5^.

### Statistical analysis

IBM SPSS Statistics, version 22.0 (IBM Corp., Armonk, NY, USA) and R software (version 4.0.3) were used to perform the statistical analysis. Quantitative variables were evaluated using Student’s *t*-test or the Mann–Whitney *U* test, and categorical variables were analyzed using Fisher’s exact test to determine the statistical differences among clinical variables. Differences in microbial taxa were analyzed using the Mann–Whitney *U* test, and false discovery rate-corrected *P* < 0.05 was considered significant. Spearman’s rank correlation analysis was used to examine the associations between clinical characteristics and gut microbial taxa. In the exploratory analyses probing the links between these clinical traits and microbial taxa, findings with *P* < 0.05 following multiple hypothesis testing are presented and elaborated upon.

### Data availability

The sequences that were generated have been submitted to the National Center for Biotechnology Information Sequence Read Archive (https://www.ncbi.nlm.nih.gov/sra) under accession code SRP515491. This published article and its supplementary files contain the main datasets. Further datasets are available from the corresponding author upon reasonable request.

## Results

### Demographic characteristics and clinical features of participants

This study enrolled 81 patients with PD and 41 healthy controls. The two groups exhibited no significant differences in age, sex, or body mass index. The patients with PD had a mean H&Y stage of 2.3 ± 0.6, a disease duration of 6.5 ± 3.9 years, and a total MDS-UPDRS score of 57.9 ± 22.2 points. All patients with PD were on anti-parkinsonian medications. The demographic characteristics and clinical features are outlined in **Table 1**.

**Table 1:** Demographic characteristics and clinical features of the participants

	Patients with PD (*n* = 81)	Healthy controls (*n* = 41)	*P*-value
Age, yr	67.8±6.9	67.7±7.4	0.989
Female, *n* (%)	44 (54.3)	20 (48.8)	0.572
BMI, kg/m^2^	23.6±2.6	23.2±2.2	0.242
Disease duration, yr	6.5±3.9		
H&Y stage	2.3±0.6		
MDS-UPDRS total score	57.9±22.2		
MDS-UPDRS-I score	10.0±5.2		
MDS-UPDRS-II score	13.4±7.4		
MDS-UPDRS-III score	33.0±12.1		
MDS-UPDRS-IV score	1.4±2.8		
LEDD, mg	517.4±230.9		
NMSS	36.5±30.1		
PDQ39	31.4±23.5		
HAMA	6.3±5.1		
PDSS_2	12.6±9.0		
MMSE	27.4±2.8		
MoCA	22.1±4.9		
Medication for PD			
Levodopa/benserazide, *n* (%)	77 (95.1)		
Levodopa/carbidopa, *n* (%)	23 (28.4)		
Dopamine agonist, *n* (%)	50 (61.7)		
COMT inhibitor, *n* (%)	2 (2.5)		
MAO-B inhibitor, *n* (%)	17 (21.0)		
Trihexyphenidyl, *n* (%)	6 (7.4)		
Amantadine, *n* (%)	10 (12.3)		

Results are presented as mean ± standard deviation or *n* (%). The *P* value reflects the difference between patients with PD and healthy controls. Differences between groups were assessed using the Mann–Whitney U test for quantitative variables and Fisher’s exact tests for categorical variables. BMI: Body mass index; COMT: catechol-O-methyl transferase; H&Y: Hoehn and Yahr; HAMA: Hamilton Anxiety Rating Scale; LEDD: levodopa equivalent daily dose; MAO-B: monoamine oxidase-B; MDS-UPDRS: Movement Disorder Society Unified PD Rating Scale; MMSE: Mini-Mental State Examination; MoCA: Montreal Cognitive Assessment; PD: Parkinson’s disease; PDQ-39: 39-item Parkinson's Disease Questionnaire; PDSS-2: Parkinson’s Disease Sleep Scale 2.

### Reconstruction of 2837 metagenome-assembled genomes from patients with Parkinson’s disease and healthy controls

Single-sample metagenomic binning from 81 patients with PD and 41 healthy controls were performed. We obtained non-redundant MAGs with ANI > 99%, and generated 1979 MAGs from patients with PD and 858 MAGs from healthy controls. All MAGs were of medium quality or better. Of these, 950 MAGs from patients with PD and 412 MAGs from healthy controls were considered high quality (**Additional Figure 1** and **Additional Table 1**).

### Taxonomic labels of metagenome-assembled genomes from fecal metagenomes

**Figure 1:**
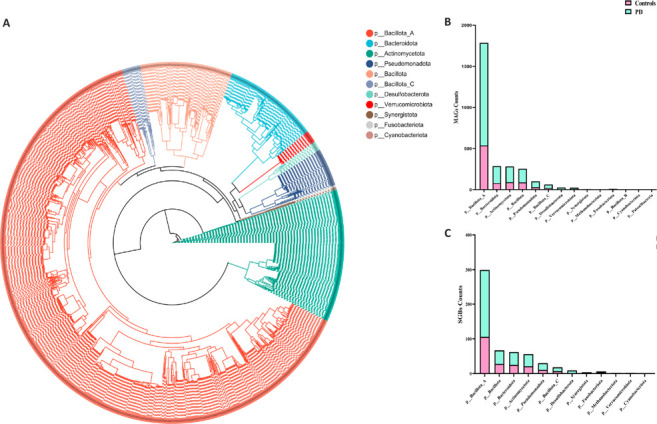
The taxonomic labels of MAGs. (A) Phylogenomic tree of MAGs recovered from this study, with clades colored by phylum. (B, C) The number of MAGs (B) and SGBs at phylum level (C). MAGs: Metagenome-assembled genomes; SGBs: species-level genome bins.

The 2837 MAGs were subsequently classified into taxa using GTDB-Tk (**Additional Table 2**). All 2837 MAGs were classified from phylum to genus level, and only 48 MAGs from patients with PD and 10 MAGs from healthy controls were not classified at the species level (**Table 2**). A phylogenetic tree of the MAGs was constructed (**Figure 1A**). The 1979 MAGs from patients with PD were identified to 14 phyla, 16 classes, 35 orders, 72 families, 258 genera, and 523 known species. Of the MAGs identified to phyla, most belonged to *Bacillota_A* (*n* = 1248), followed by *Bacteroidota* (*n* = 206), *Actinomycetota* (*n* = 192), *Bacillota* (*n* = 165), *Pseudomonadota* (*n* = 71), *Bacillota_C* (*n* = 43), *Desulfobacterota* (*n* = 22), and *Verrucomicrobiota* (*n* = 17). The 858 MAGs from healthy controls were identified to nine phyla, 10 classes, 25 orders, 46 families, 162 genera, and 325 species. Most MAGs from healthy controls belonged to *Bacillota_A* (*n* = 537), followed by *Actinomycetota* (*n* = 90), *Bacillota* (*n* = 88), *Bacteroidota* (*n* = 80), *Pseudomonadota* (*n* = 29), and *Bacillota_C* (*n* = 19) (**Figure 1B**).

**Table 2:** The taxonomic labels of MAGs from fecal metagenomes from patients with PD and healthy controls

Taxonomic level		Healthy controls				Patients with PD		
			
		Classified MAGs	Identified taxa	Unclassified MAGs		Classified MAGs	Identified taxa	Unclassified MAGs
Phylum		858	9	0		1979	14	0
Class		858	10	0		1979	16	0
Order		858	25	0		1979	35	0
Family		858	46	0		1979	72	0
Genus		858	162	0		1979	258	0
Species		848	324	10		1931	523	48

MAGs: Metagenome-assembled genomes; PD: Parkinson’s disease.

All members of *Bacillota_A* in patients with PD belonged to the class *Clostridia* (*n* = 1248). The members of class *Clostridia* included *Oscillospirales* (*n* = 590), *Lachnospirales* (*n* = 482), *Christensenellales* (*n* = 83), *Peptostreptococcales* (*n* = 32), *UBA1381* (*n* = 28), *TANB77* (*n* = 25), *Clostridiales* (*n* = 5), *Monoglobales* (*n* = 1), *Tissierellales* (*n* = 1), and *UMGS1883* (*n* = 1). All members of Bacillota_A in healthy controls belonged to the class *Clostridia* (*n* = 537). The members of class *Clostridia* included *Lachnospirales* (*n* = 267), *Oscillospirales* (*n* = 212), *UBA1381* (*n* = 17), *Christensenellales* (*n* = 13), *TANB77* (*n* = 11), *Peptostreptococcales* (*n* = 11), *Clostridiales* (*n* = 3), and *Eubacteriales* (*n* = 1).

The 2837 MAGs were further clustered into 578 SGBs, of which 22 were defined as uSGB (**Additional Table 3**). Of these uSGBs, 20 were from patients with PD and two were from healthy controls. Of the 354 identified SGBs in patients with PD, most belonged to *Bacillota_A* (*n* = 193), followed by *Bacillota* (*n* = 39), *Bacteroidota* (*n* = 37), *Actinomycetota* (*n* = 35), *Pseudomonadota* (*n* = 19), *Bacillota_C* (*n* = 11), *Desulfobacterota* (*n* = 8), *Synergistota* (*n* = 4), *Fusobacteriota* (*n* = 3), *Methanobacteriota* (*n* = 2), V*errucomicrobiota* (*n* = 2), and *Cyanobacteriota* (*n* = 1). The 202 identified SGBs in healthy controls included *Bacillota_A* (*n* = 106), followed by *Bacillota* (*n* = 28), *Bacteroidota* (*n* = 25), *Actinomycetota* (*n* = 21), *Pseudomonadota* (*n* = 11), *Bacillota_C* (*n* = 7), *Fusobacteriota* (*n* = 3), and *Desulfobacterot*a (*n* = 1) (**Figure 1C**).

**Additional Table 3:** Number of species-level genome bins (SGBs) at phylum level

phylum	PD_class_sgb	PD_unclass_sgb	Con_class_sgb	Con_unclass_sgb
p_Bacillota_A	193	5	106	0
p_Bacillota	39	1	28	1
p_Bacteroidota	37	2	25	0
p_Actinomycetota	35	9	21	1
p_Pseudomonadota	19	2	11	0
p_Bacillota_C	11	1	7	0
p_Desulfobacterota	8	0	1	0
p_Synergistota	4	0	0	0
p_Fusobacteriota	3	0	3	0
p_Methanobacteriota	2	0	0	0
p_Verrucomicrobiota	2	0	0	0
p_Cyanobacteriota	1	0	0	0

### Comparison of metagenome-assembled genomes between patients with Parkinson’s disease and healthy controls

**Figure 2:**
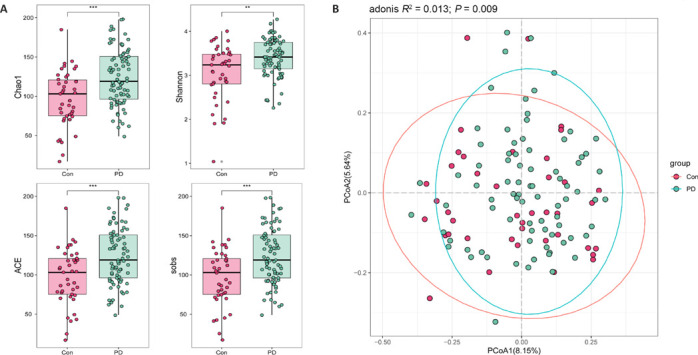
Alteration of metagenome-assembled genomes-annotated microbial taxa. (A, B) Alpha diversity (A) and PCoA based on Bray–Curtis dissimilarity of microbial species (B). **P < 0.01, ***P < 0.001. ACE: Abundance-based coverage estimator; Con: control; PCoA: principal coordinates analysis.

**Figure 3:**
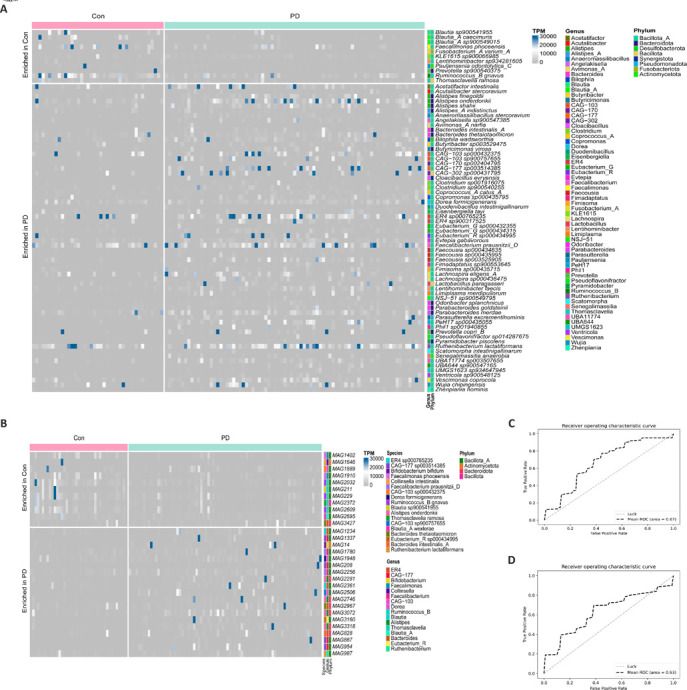
The difference of microbial taxa between patients with PD and healthy controls. (A, B) Different MAGs-annotated species (A) and MAGs (B) between patients with PD and healthy controls. (C, D) The receiver operating characteristic in discriminating patients with PD from healthy controls based on species (C) and MAGs (D) biomarkers. MAGs: Metagenome-assembled genomes; PD: Parkinson’s disease.

To compare the microbial composition between patients with PD and healthy controls, we quantified and compared MAGs abundance. Several α-diversity indexes—namely, the abundance-based coverage estimator (*P* < 0.001), Chao1 (*P* < 0.001), Shannon (*P* < 0.01), and Sobs (*P* < 0.001)—were significantly higher in patients with PD than in healthy controls (**Figure 2A**). Principal coordinate analysis plots revealed that the samples from the two groups were significantly separated (*R*^2^ = 0.013, *P* = 0.009; **Figure 2B**). Subsequently, we identified MAGs with significantly different abundance levels between the two groups. We then constructed heatmaps at both the species and strain levels. At the species level, *Blautia* spp., *Faecalimonas phoceensis*, *Fusobacterium_A varium_A*, *Prevotella sp900540375*, and *Ruminococcus_B gnavus* were enriched in healthy controls, whereas *Acetatifactor intestinalis*, *Acutalibacter stercoravium*, *Alistipes* spp., *Bacteroides* spp., *Clostridium* spp., *Eubacterium* spp., *Lachnospira* spp., and *Prevotella copri_B* were enriched in patients with PD (**Figure 3A**). At the strain level (MAGs), we identified 348 MAGs with differential abundance levels (**Additional Table 4**). The majority of these MAGs were annotated as known phyla. Specifically, there were 200 MAGs belonging to *Bacillota_A*, 31 belonging to *Bacteroidota*, 15 belonging to *Bacillota*, five belonging to *Bacillota_C*, four belonging to *Actinomycetota*, three belonging to *Desulfobacterota*, three belonging to *Pseudomonadota*, and two belonging to *Synergistota* (the top 30 of the 348 MAGs are shown in **Figure 3B**).

We further compared the abundance levels of the 2837 MAGs from the phylum level to the genus level. At the phylum level, *Bacteroidota*, *Bacillota_C*, *Desulfobacterota*, *Fusobacteriota*, and *Synergistota* showed high abundance in patients with PD, whereas *Bacillota* was present at low levels. At the family level, *Bacteroidaceae*, *Oscillospiraceae*, *Rikenellaceae*, *CAG-74*, *Acidaminococcaceae*, *Desulfovibrionaceae*, *Burkholderiaceae_A*, *Fusobacteriaceae*, *Anaerovoracaceae*, *Marinifilaceae*, *CAG-138*, *UBA644*, *Synergistaceae*, *Dethiosulfovibrionaceae*, and *CAG-239* showed high abundance in patients with PD, whereas *Clostridiaceae* was present at low levels. At the genus level, *Alistipes*, *ER4*, *CAG-103*, *Lachnospira*, *Vescimonas*, *UBA11774*, *Desulfovibrio*, *Lentihominibacter*, *Faecousia*, *Odoribacter*, *Butyricimonas*, *Anaeromassilibacillus*, *Zhenpiania*, *PeH17*, *UMGS1623*, *Cloacibacillus*, *Pyramidobacter*, *Pullichristensenella*, and *Avimonas_A* showed high abundance in patients with PD, whereas *Ruminococcus_B*, *Faecalimonas*, *KLE1615*, and *Thomasclavelia* were present at low levels (**Additional Figure 2**).

We then trained a classification model for the disease condition using the random forest algorithm based on differential species and MAGs. Among the models developed, one that was built on 30 differential species biomarkers demonstrated the highest accuracy (area under the receiver operating characteristic curve [AUC] = 0.67). By contrast, for the model constructed using differential MAGs, the most accurate version (AUC = 0.63) comprised 50 differential MAG biomarkers (**Figure 3C** and **D**).

### Correlation analysis between metagenome-assembled genomes and clinical indexes in patients with Parkinson’s disease

**Figure 4:**
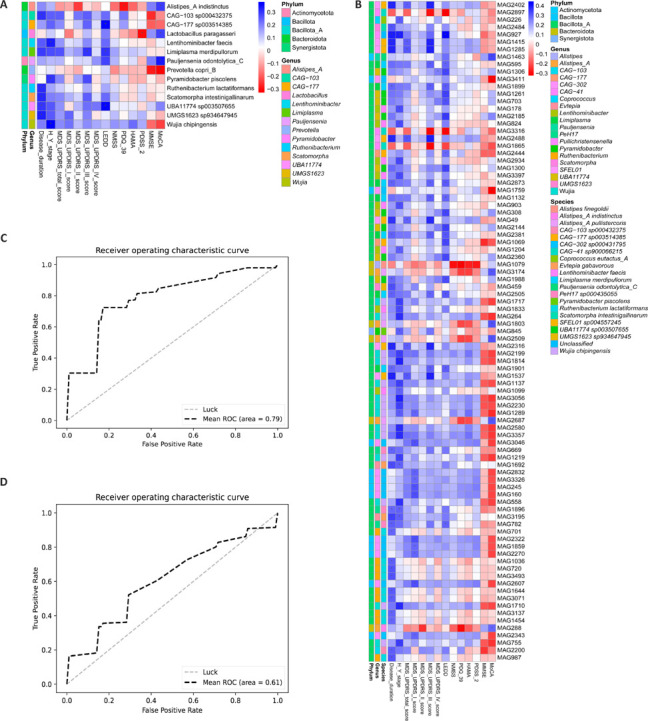
The correlation analysis of MAGs and clinical indexes. (A, B) The correlation analysis of MAGs-annotated species (A) and MAGs (B) with clinical indexes in patients with PD. (C, D) The receiver operating characteristic curve in discriminating advanced patients with PD from patients with early PD based on MAGs (C) and species (D) biomarkers. The correlations of clinical characteristics with gut microbial taxa were performed using Spearman’s rank correlation analysis. *P < 0.01. MAGs: Metagenome-assembled genomes; PD: Parkinson’s disease.

Next, we further explored MAGs that exhibited significantly differential abundance levels between patients with PD and healthy controls. At the species level, *CAG−103 sp000432375*, *Lentihominibacter faecis*, *Limiplasma merdipullorum*, *Pyramidobacter piscolens*, *Ruthenibacterium lactatiformans*, *Scatomorpha intestinigallinarum*, *UBA11774 sp003507655*, *UMGS1623 sp934647945*, and *Wujia chipingensis* were positively correlated with disease severity (disease duration, H&Y stage, or MDS-UPDRS III score, *P* < 0.01; **Figure 4A**). In addition, *Acutalibacter stercoravium*, *Anaeromassilibacillus stercoravium*, *CAG−170 sp002404795*, *CAG−177 sp003514385*, *Cloacibacillus evryensis*, *Eisenbergiella tayi*, *Evtepia gabavorous*, *Faecousia sp000434635*, *Faecousia sp000435995*, *Faecousia sp003525905*, *Fimisoma sp000435715*, *Parabacteroides merdae*, *PeH17 sp000435055*, *Pseudoflavonifractor sp014287675*, *Senegalimassilia anaerobia*, and *UBA644 sp900547165* were positively correlated with disease severity (disease duration, H&Y stage, or MDS-UPDRS III score), whereas Bilophila wadsworthia and Blautia sp900541955 were negatively correlated with H&Y stage (*P* < 0.05; **Additional Figure 3**). *Blautia_A caecimuris* was negatively correlated with MDS-UPDRS IV score, whereas *Clostridium sp001916075*, *Clostridium sp900540255*, and *KLE1615 sp900066985* were positively correlated with MDS-UPDRS IV score (*P* < 0.05; **Additional Figure 3**). Moreover, *CAG−177 sp00351438*5, *Limiplasma merdipullorum*, and *UBA11774 sp003507655* were positively correlated with LEDD (*P* < 0.01; **Figure 4A**). In addition, *Anaeromassilibacillus stercoravium*, *CAG−103 sp000432375*, C*AG−302 sp000431795*, *Evtepia gabavorous*, *Faecousia sp000434635*, *Lentihominibacter faecis*, *Pauljensenia odontolytica_C*, *PeH17 sp000435055*, and *Scatomorpha intestinigallinarum* were positively correlated with LEDD (*P* < 0.05; **Additional Figure 3**). *Alistipes_A indistinctus*, *Lactobacillus paragasseri*, *Pauljensenia odontolytica_C*, and *Prevotella copri_B* were significantly associated with non-motor symptoms (Non-Motor Symptom Scale, PDQ-39, Hamilton Anxiety Rating Scale, PD Sleep Scale 2, Mini-Mental State Examination, or Montreal Cognitive Assessment, *P* < 0.01; **Figure 4A**). In addition, *Alistipes finegoldii*, *Anaeromassilibacillus stercoravium*, *Butyricimonas virosa*, *CAG−103 sp000432375*, *CAG−103 sp900757655*, *CAG−170 sp002404795*, *CAG−302 sp000431795*, *Faecousia sp000435995*, *Fimisoma sp000435715*, *KLE1615 sp900066985*, *NSJ−51 sp900549795*, *Parasutterella excrementihominis*, and *Wujia chipingensis* were significantly associated with non-motor symptoms (*P* < 0.05; **Additional Figure 3**). The correlations with clinical indexes in patients with PD were further investigated at strain level (MAGs). Of the 188 MAGs that were significantly correlated with clinical characteristics (*P* < 0.05, **Additional Figure 4**), 89 are shown in **Figure 4B** (*P* < 0.01). Furthermore, among the 188 significant MAGs, 132 were correlated with disease severity; these belonged mostly to the *phylum Bacillota_A*, followed by *Bacteroidota*, *Actinomycetota*, *Synergistota*, and *Bacillota_C* (*P* < 0.05; **Additional Figure 4**). Moreover, 18 MAGs were correlated with MDS-UPDRS IV score, belonging to *phyla Bacillota_A*, *Bacteroidota*, and *Bacillota*. In addition, 59 MAGs were associated with LEDDs, and 51 MAGs were correlated with non-motor symptoms (*P* < 0.05; **Additional Figure 4**).

We used the random forest algorithm to train a model for discriminating early PD from advanced PD (early PD: H&Y stage < 2.5; advanced PD: H&Y stage ≥ 2.5). This model was based on the intestinal species and MAGs associated with disease severity. Of the models, the one constructed using intestinal MAGs achieved the highest accuracy (AUC = 0.79); this model incorporated 26 associated MAG biomarkers. Conversely, for the model built upon intestinal species, the most accurate version (AUC = 0.61) consisted of three associated species biomarkers (**Figure 4C** and **D**).

### Functional potential of metagenome-assembled genomes in patients with Parkinson’s disease

**Figure 5:**
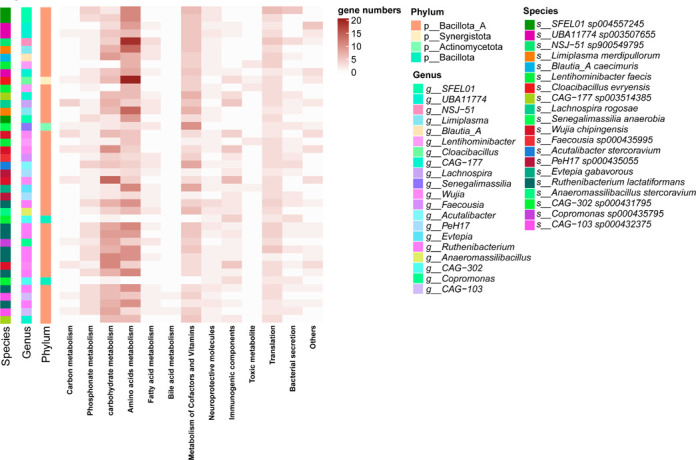
A heatmap profile showing the distribution of metagenome-assembled genomes in the microbial function associated with Parkinson’s disease.

Because we identified that several MAGs were associated with disease severity, we used the KEGG annotation database to elucidate the functional potential of these MAGs and explore their potential mechanistic implications. In PD, alterations in the abundance levels of several functional pathways have been reported (Wallen et al., 2022). Many pathways and KEGG Orthology groups in the KEGG annotation database are not specific to PD; however, we focused on PD-associated functional pathways to investigate the functional profiles of the MAGs associated with disease severity. After annotating the functional pathways of the 132 MAGs correlated with disease severity using the KEGG database, 41 MAGs were successfully annotated. The genes of these MAGs were distributed across multiple pathways, such as carbon metabolism, phosphonate metabolism, carbohydrate metabolism, amino acid metabolism, fatty acid metabolism, bile acid metabolism, metabolism of cofactors and vitamins, neuroprotective molecules, immunogenic components, toxic metabolites, translation, and bacterial secretion (**Figure 5**).

## Discussion

Our investigation of microbial genomes has greatly enhanced the understanding of how gut microbiota affect host disease development. Previous studies using 16S rRNA gene amplicon sequencing or assembly-free-based metagenomic approaches have demonstrated gut microbiota dysbiosis in PD (Heintz-Buschart et al., 2018; Boktor et al., 2023). Nevertheless, it is essential to further explore the gut microbiota at the genome level. In the present study, we reconstructed microbial genomes from metagenomic sequences. This approach markedly extends the diversity and number of microbial genomes, particularly those of uncultivable strains (Bowers et al., 2017). By combining the analysis of MAGs with clinical parameters, we identified gut microbiota dysbiosis in patients with PD. We also identified microbial species and MAGs that were significantly associated with clinical characteristics and explored the functional potential of PD-related MAGs. Our results provide in-depth insights into the gut microbiota at the genome level and offer detailed information about their functional potential, thereby improving our comprehension of the role of gut microbiota in PD development.

High-throughput shotgun metagenomic sequencing serves as a robust tool for exploring microbial communities. When short metagenomic reads are assembled into longer genomic fragments (contigs), full-length genomes often fail to be generated. Thus, metagenomic binning is a crucial technique for the genome-resolved characterization of uncultured microorganisms. It can be used to construct draft genomes from assembled metagenomic contigs, thereby serving as a substitute for full-length genomes (Kang et al., 2015). Previous studies have explored the microbial profiles in patients with PD through metagenome-assembled genomes (Duru et al., 2024; Lee et al., 2025). Using single-sample metagenomic binning, we reconstructed 2837 MAGs from patients with PD and healthy controls. Nearly half of these MAGs were categorized as high-quality draft genomes. Subsequently, we classified these MAGs into taxa using the GTDB, which contains nearly 250,000 bacterial and archaeal genomes and provides a phylogenetically consistent taxonomy for bacteria and archaea, thus accommodating both isolated genomes and thousands of MAGs (Kang et al., 2015). On the basis of the taxonomic classification, the four most abundant phyla in both patients with PD and healthy controls were *Bacillota*, *Bacteroidota*, *Actinomycetota*, and *Pseudomonadota*, which aligns with prior research findings (Qian et al., 2020). To accurately profile the taxonomy of all species represented by the available MAGs, we used the SGB approach to divide the MAGs into SGBs or uSGBs. uSGBs represent the portion of the microbial genome that is not covered by existing reference genomes. They serve as a substitute for an uncultivated microbial species whose existence is inferred from metagenomic assembly data. In the present study, the 2837 MAGs were clustered into 578 SGBs, of which 22 were identified as uSGBs. These uSGBs are potentially novel species, which might expand the number of available microbial reference genomes.

Gut microbiota characteristics are markedly altered in patients with PD (Keshavarzian et al., 2015; Qian et al., 2018). In the current study, by analyzing the abundance of MAGs, we also observed higher α-diversity indexes and a very different composition of microbial communities in patients with PD. Several MAG taxa with differential abundance levels were identified. Consistent with a previous study (Keshavarzian et al., 2015), *Bacteroidota*, *Bacteroidaceae*, and *Bacteroides spp*. showed high abundance in patients with PD, whereas *Bacillota* (also known as *Firmicutes*) was present at low levels. The *phylum Bacteroidota*, which is mainly composed of gram-negative bacteria, is often linked to gut inflammation (Bank et al., 2024). Notably, reduced enterocyte-protective *Firmicutes* bacteria and the increased growth of *Bacteroidota*, which produce highly inflammatory lipopolysaccharides, are related to high-level systemic inflammation (McCracken and Nathalia Garcia, 2021). Moreover, *Desulfobacterota*, *Desulfovibrionaceae*, and *Desulfovibrio* were enriched in patients with PD. *Desulfobacterota* are gram-negative sulfate-reducing bacteria; they generate hydrogen sulfide and lipopolysaccharides and induce the oligomerization and aggregation of α-synuclein (Huynh et al., 2023). These effects are pro-inflammatory and contribute to PD pathology, indicating that *Desulfobacterota* might be implicated in PD development (McCracken and Nathalia Garcia, 2021; Xu et al., 2023). In addition, *Fusobacteriota* and *Fusobacteriaceae* showed high abundance in patients with PD. Some taxa within *Fusobacteriota* exhibit pro-inflammatory properties and can adhere to the intestinal mucosa, which is associated with the development of intestinal inflammation (Maldonado-Arriaga et al., 2021). In the present study, short-chain fatty acid (SCFA)-producing bacteria such as *Blautia* spp., *Ruminococcus_B gnavus*, and *Ruminococcus_B* were present at low levels in patients with PD. Given that SCFA has anti-inflammatory properties (Dalile et al., 2019), the decline in these taxa may lead to decreased SCFAs, thus promoting the development of PD. However, several SCFA-producing bacteria, including *Alistipes* spp., *Lachnospira* spp., *Butyricimonas*, *Odoribacter*, *Clostridium* spp., *Eubacterium* spp., and *Acidaminococcaceae* showed high abundance in patients with PD. Similarly, *Synergistota* and *Synergistaceae*, which play anti-inflammatory roles or have beneficial effects on microbiota maintenance and homeostasis (McCracken and Nathalia Garcia, 2021), were also enriched in patients with PD. These findings suggest that the elevated abundance of SCFA-producing bacteria and anti-inflammatory bacteria might represent a compensatory response of the microbiome to the disease.

Our classification model for disease condition based on differential MAGs between patients with PD and healthy controls showed moderate predictive performance. MAGs can reconstruct individual microbial genomes from metagenomic data, offering high microbial resolution and enriching gut microbiome databases. They also enable the reconstruction of uncultured microbial genomes, thereby assisting in identifying novel species and broadening our comprehension of gut microbiota diversity (Huang et al., 2024). However, because of potentially unidentified species, MAGs may not be superior to known-species-based markers in diagnostics. Additionally, the complexity, susceptibility, and individual variation of gut microbiota makes it challenging to identify efficient predictive markers; this can result in low prediction accuracy (Yan and Li, 2020; Fonseca et al., 2024).

Previous studies have explored the correlations between microbial taxa and the clinical characteristics of patients with PD (Qian et al., 2018; Barichella et al., 2019). However, these studies were mainly conducted at phylum, family, and genus levels. By contrast, our study delved deeper into microbial genomes. We identified that an increasing number of microbial taxa at both species and MAG levels showed links to clinical traits of PD, including disease severity, medication, motor complications, and non-motor symptoms. Most of these relationships between taxa and clinical characteristics have not yet been previously reported. *Pyramidobacter* *piscolens* and *Cloacibacillus evryensis*, which belong to the phylum Synergistetes, were positively associated with disease severity (as measured by H&Y stage, MDS-UPDRS III score, and disease duration). Given that the phylum *Synergistetes* may have an anti-inflammatory function or exert beneficial effects (McCracken and Nathalia Garcia, 2021), the positive associations between its members and disease severity further imply a compensatory response of the microbiome to PD. *Eisenbergiella tayi*, which is a member of the family *Lachnospiraceae*, was positively associated with disease duration; this aligns with prior research findings (Barichella et al., 2019). *Parabacteroides merdae*, from the phylum *Bacteroidetes*, had a positive correlation with H&Y stage. This finding indicates that its abundance may increase as the H&Y stage progresses. Furthermore, the phylum *Bacteroidetes* was positively correlated with disease duration in PD (Keshavarzian et al., 2015), and the genus *Bacteroides* was also positively associated with UPDRS III score (Lin et al., 2019). Given that *Parabacteroides merdae* is involved in branched-chain amino acid (BCAA) catabolism (Qiao et al., 2022), its increased abundance may lead to lower BCAA levels as the H&Y stage advances, which aligns with our previous findings (Zhang et al., 2022). BCAAs are beneficial in the pathogenesis of PD (Yan et al., 2022), indicating that BCAAs may be key mediators for the interaction between *Parabacteroides merdae* and H&Y stage. In addition, *Bilophila wadsworthia*, belonging to the family *Desulfovibrionaceae*, was negatively associated with H&Y stage. Because *Bilophila wadsworthia* participates in bile acid metabolism (Devkota et al., 2012), and bile acid-mediated oxidative stress and neuroinflammation might be related to PD pathogenesis (Mao et al., 2023), this species may interact with PD disease severity through bile acid metabolism. *Alistipes_A indistinctus* and *Alistipes finegoldii*, two members of the phylum *Bacteroidetes*, were negatively associated with PDQ-39 score. The genus *Alistipes* plays a critical role in inflammation and might affect the gut–brain axis, thus affecting diseases outside the gut (Parker et al., 2020). This might explain the relationship between these gut bacteria and non-motor symptoms of PD. Furthermore, we revealed that *Prevotella copri_B* was negatively associated with cognitive performance, whereas *Lactobacillus paragasseri* was positively associated with cognitive performance. Similarly, in a randomized clinical trial, *Prevotella spp*. was correlated with cognitive impairment in middle-aged and older adults, and probiotic *Lactobacillus rhamnosus* GG treatment improved cognitive scores (Aljumaah et al., 2022). Furthermore, the *Blautia_A caecimuris* and *Clostridium* spp. were related to MDS-UPDRS IV score, which reflects the motor complications of PD. A previous report also demonstrated links between gut microbiota and unpredictable motor fluctuations in patients with PD (Zhang et al., 2025). Moreover, *Lentihominibacter faecis*, *Limiplasma merdipullorum*, *Pauljensenia odontolytica_C*, *Scatomorpha intestinigallinarum*, *Anaeromassilibacillus stercoravium*, and *Evtepia gabavorous* were positively correlated with LEDD. These findings indicate that the microbiota may affect drug metabolism, or that drugs might alter the microbiota (Zhang et al., 2025).

Given the associations between MAGs and disease severity, we investigated the metabolic functions of MAGs to uncover any potential mechanistic links. The MAGs associated with disease severity were involved in carbohydrate metabolism, fatty acid metabolism, and amino acid metabolism. These functional alterations in the microbiome of PD have been reported in previous studies (Hill-Burns et al., 2017; Cirstea et al., 2020; Qian et al., 2020). A deficiency in gut SCFA content has been linked to PD severity, constipation, changes in gut–blood barrier permeability, and neuroinflammation (Yang et al., 2022; Kalyanaraman et al., 2024). Our recent study also detected disturbances in microbial amino acid metabolism in patients with PD (Zhang et al., 2022). The disease severity-associated MAGs were also related to cofactors and vitamins metabolism. Previous findings have documented alterations in microbial cofactors and vitamin metabolism in patients with PD (Hill-Burns et al., 2017; Qian et al., 2020; Huang et al., 2023). Vitamins play crucial roles in the central nervous system through various physiological processes as well as via the synthesis and metabolism of neurotransmitters and neurohormones (Rudzki et al., 2021). In addition, disease severity-associated MAGs were annotated to metabolic pathways including bile acid metabolism, neuroprotective molecules, immunogenic components, and toxic metabolites. The gut microbiota participates in bile acid biotransformation reactions, and bile acids interact with the host and exert immunomodulatory effects through different bile acid receptors and cell signaling pathways (Cai et al., 2022). A study has provided evidence that bile acids might play a role in PD development, and potential treatment strategies for PD based on bile acids are currently being explored (Hurley et al., 2022).

Common gut microbiota-derived neuroprotective molecules include nicotinate, nicotinamide, and trehalose. Previous studies have reported metabolic disturbances of these neuroprotective molecules in patients with PD (Barichella et al., 2019; Wallen et al., 2022), and both nicotinamide and trehalose are possible therapeutic candidates for PD treatment (Pupyshev et al., 2019; Brakedal et al., 2022). Immunogenic component genes in the microbiome are increased in patients with PD (Wallen et al., 2022) and are highly immunostimulatory because they trigger immune reactions and inflammation. Given that inflammation is a major driver of PD pathogenesis (Pajares et al., 2020), microbial immunogenic components may contribute to PD pathogenesis through an inflammatory mechanism. Trimethylamine is a PD-associated toxic metabolite, and microbial trimethylamine production is increased in patients with PD (Wallen et al., 2022). Trimethylamine N-oxide, an oxidation product of trimethylamine that is derived from the gut microbiome, was reportedly increased in patients with PD and positively correlated with disease severity and motor symptom progression in a longitudinal follow-up study (Chen et al., 2020). In our study, disease severity-associated MAGs were annotated to bacterial secretion. Similarly, it has been reported that type III secretion systems show high abundance in the PD microbiota (Keshavarzian et al., 2015). These findings indicate that gut microbiota dysbiosis might drive PD through type III secretion system-associated neuroinflammation. However, the functional analysis of MAGs linked to disease severity has not yet been mechanically validated, and more research is needed. This may include measuring metabolites or studying host–microbe interactions. Such studies should be performed in animal models or *in vitro* systems to confirm our findings.

The current study had some limitations. First, although the patients with PD included in the study were taking medication, the potential effects of these drugs on the gut microbiota were not explored. Because anti-parkinsonian drugs may influence microbial composition (Zhang et al., 2025), the use of medication may interfere with the interpretation of PD-related microbial changes. Second, the relatively small sample size and lack of external validation in the present study may affect the robustness and generalizability of its findings.

In conclusion, the current study harnessed the advantages of single-sample metagenomic binning rather than using 16S rRNA gene amplicon sequencing or assembly-free-based metagenomic methods. In this way, it provided insights into the gut microbiota at the genome level and revealed detailed information about their functional potential. By conducting a correlation analysis using microbial genome data, the study further elucidated the relationships between clinical parameters and specific gut microorganisms.

## Additional files:

***Additional Figure 1:*** *Annotation characterization in MAGs.*

Additional Figure 1 Annotation characterization in MAGs.
The distributions of average length of contigs (A), genome size (B), contigs numbers (C), and the completeness and contamination of MAGs (D). MAGs: Metagenome-assembled genomes; PD: Parkinson’s disease.

***Additional Figure 2:*** *Comparative analysis of taxonomic labels of MAGs at different levels between patients with Parkinson’s disease and healthy controls.*

Additional Figure 2 Comparative analysis of taxonomic labels of MAGs at different levels between patients with Parkinson’s disease and healthy controls.
The difference of microbial taxa was analyzed using a Mann–Whitney *U* test, and FDR-*P* < 0.05 was considered to be statistically significant. *FDR*-P* < 0.05; **FDR-*P* < 0.01. MAGs: Metagenome-assembled genomes.

***Additional Figure 3:*** *Correlation analysis of MAGs-annotated species with clinical indexes in patients with Parkinson’s disease.*

Additional Figure 3 Correlation analysis of MAGs-annotated species with clinical indexes in patients with Parkinson’s disease.
The correlations of clinical characteristics with gut microbial taxa were performed using Spearman’s rank correlation analysis. *P < 0.05. MAGs: Metagenome-assembled genomes.

***Additional Figure 4:*** *Correlation analysis of MAGs with clinical indexes in patients with Parkinson’s disease.*

Additional Figure 4 Correlation analysis of MAGs with clinical indexes in patients with Parkinson’s disease.
The correlations of clinical characteristics with gut microbial taxa were performed using Spearman’s rank correlation analysis. *P < 0.05. MAGs: Metagenome-assembled genomes.

***Additional Table 1:*** *Characterization of completeness, contamination, and genomes of MAGs.*

Additional Table 1 Characterization of completeness, contamination, and genomes of MAGs

***Additional Table 2:*** *Taxonomy labels of MAGs annotated by GTDB-TK.*

Additional Table 2 Taxonomy labels of MAGs annotated by GTDB-TK

***Additional Table 3:*** *Number of species-level genome bins (SGBs) at phylum level.*

***Additional Table 4:*** *Correlation analysis of metagenome-assembled genomes with clinical indexes in patients with Parkinson’s disease.*

Additional Table 4 MAGs with different abundance between PD patients and healthy controls

## Data availability statement:

*All relevant data are within the paper and its Additional files.*
