# Supplementary material for: Gut microbial community of patients with Parkinson’s disease analyzed using metagenome-assembled genomes
Source: Neural Regen Res. 2025 Sep 29;21(8):3815–23. doi: 10.4103/NRR.NRR-D-25-00420 (PMC13460800; doi:10.4103/NRR.NRR-D-25-00420)
Supplement: Supplementary file 1 [file NRR-21-3815_Suppl1.pdf]

Additional Table 1 Characterization of completeness, contamination, and genomes of MAGs

Group	Bin Id	Bin	MAG Id	Contigs Num	Genome size (bp)	Longest contig (bp)	N50 (contigs) (bp)	Mean contig length (bp)	Completeness (%)	Contamination (%)
PD	PD100	bin45	MAG2105	48	2186067	198142	84093	45543	100	0.81
PD	PD11	bin8	MAG1135	51	2387906	210894	79840	46822	100	0
PD	PD12	bin29	MAG2966	40	2389199	234454	100971	59730	100	0
PD	PD14	bin44	MAG2536	40	2378170	475387	160102	59454	100	0.58
PD	PD16	bin66	MAG1775	36	2601340	453334	270026	72259	100	0
PD	PD19	bin33	MAG2860	38	2237533	172415	83226	58882	100	1.61
PD	PD20	bin11	MAG2256	97	2381252	181961	82668	24549	100	0.17
PD	PD20	bin37	MAG2906	56	2194081	180561	77067	39180	100	1.61
PD	PD21	bin30	MAG2547	30	1686567	254006	140670	56219	100	0.12
PD	PD21	bin1	MAG2773	28	2263244	210275	124626	80830	100	0.12
PD	PD21	bin9	MAG795	236	3057969	90393	23999	12957	100	2.25
PD	PD27	bin1	MAG3197	44	2078823	300529	107699	47246	100	0.83
PD	PD31	bin39	MAG1802	258	2258449	107680	14161	8754	100	0
PD	PD32	bin40	MAG2804	50	2155039	130639	67279	43101	100	1.61
PD	PD35	bin36	MAG1588	34	2228598	280198	148082	65547	100	1.61
PD	PD36	bin21	MAG1101	36	1797984	186284	87109	49944	100	2
PD	PD37	bin37	MAG2240	96	2292502	164656	35082	23880	100	0
PD	PD37	bin22	MAG5	86	2116049	176405	47793	24605	100	3.23
PD	PD39	bin15	MAG2770	46	2120612	250593	119655	46100	100	0.77
PD	PD40	bin15	MAG371	61	2157074	424175	112142	35362	100	0.49
PD	PD42	bin28	MAG2022	47	1812146	121474	72811	38556	100	0
PD	PD48	bin11	MAG2475	38	2198604	341323	93600	57858	100	0.23
PD	PD50	bin39	MAG524	51	1752851	113188	59949	34370	100	0
PD	PD56	bin44	MAG2988	58	2271181	313857	87166	39158	100	1.61
PD	PD57	bin20	MAG1245	40	2635734	310851	113854	65893	100	0
PD	PD58	bin71	MAG286	196	2219426	115795	22356	11324	100	1.27
PD	PD60	bin36	MAG1780	47	2338727	203601	92916	49760	100	0
PD	PD66	bin28	MAG928	65	2276071	170604	79125	35016	100	0.15
PD	PD69	bin65	MAG1290	47	2107028	248503	103849	44830	100	1.4
PD	PD70	bin33	MAG439	42	2133347	243705	93863	50794	100	0.57

PD	PD71	bin16	MAG1662	420	2750763	48724	9489	6549	100	1.08
PD	PD71	bin62	MAG2374	133	2840085	167702	52529	21354	100	0.68
PD	PD71	bin52	MAG487	46	2669198	277606	103744	58026	100	0
PD	PD73	bin50	MAG404	45	1843942	187448	130578	40976	100	0
PD	PD8	bin53	MAG1504	187	2241900	160267	28118	11989	100	0.63
PD	PD81	bin20	MAG1848	47	2173654	190203	103721	46248	100	0.81
PD	PD81	bin1	MAG3050	36	2216270	233463	125239	61563	100	0.12
PD	PD81	bin13	MAG766	153	2990746	95401	32887	19547	100	1.34
PD	PD82	bin2	MAG764	61	2159429	145789	69780	35400	100	0.81
PD	PD86	bin4	MAG2645	73	2518679	343363	108320	34502	100	4.57
PD	PD87	bin52	MAG1834	76	2301220	159495	80001	30279	100	2.42
PD	PD87	bin70	MAG2209	18	1298179	424607	262966	72121	100	0.12
PD	PD94	bin29	MAG1613	55	2566399	204033	108835	46662	100	2.27
PD	PD94	bin47	MAG626	35	2372011	372084	171760	67772	100	0
PD	PD95	bin18	MAG46	55	2206810	172758	75767	40124	100	1.61
PD	PD98	bin33	MAG3184	55	2185917	220292	75798	39744	100	0.81
PD	PD18	bin38	MAG2152	120	4744390	223568	96234	39537	99.96	0.08
PD	PD6	bin23	MAG2	131	4987270	285399	99729	38071	99.96	0.13
PD	PD75	bin25	MAG1924	144	2424773	250782	56232	16839	99.89	1.67
PD	PD58	bin84	MAG1794	149	2491146	134921	53286	16719	99.88	6.9
PD	PD37	bin36	MAG52	120	2227468	109200	31815	18562	99.85	1.01
PD	PD9	bin67	MAG2129	75	2627836	154798	74595	35038	99.84	0.48
PD	PD41	bin38	MAG2905	67	2465060	232209	59751	36792	99.76	0.24
PD	PD25	bin13	MAG2588	79	2089450	203061	48941	26449	99.75	0.94
PD	PD10	bin53	MAG599	30	2152493	284224	143892	71750	99.7	1.04
PD	PD14	bin60	MAG398	45	2138456	291463	95251	47521	99.7	0.91
PD	PD57	bin5	MAG2950	190	2254337	143704	24967	11865	99.68	0.63
PD	PD24	bin22	MAG3065	97	4820368	232315	87857	49695	99.65	0.39
PD	PD70	bin26	MAG405	59	4547975	568396	152239	77084	99.65	0.08
PD	PD56	bin31	MAG2123	109	1681214	72513	26160	15424	99.63	0.62
PD	PD76	bin37	MAG2513	100	2797497	238400	71275	27975	99.63	2.53
PD	PD77	bin61	MAG608	62	2769624	329926	82328	44671	99.63	0.37
PD	PD59	bin35	MAG2886	117	4512148	201891	63703	38565	99.61	0.08

PD	PD100	bin27	MAG2840	66	2095428	302389	47558	31749	99.58	1.5
PD	PD86	bin9	MAG2464	70	2161795	306578	118826	30883	99.55	3.34
PD	PD27	bin32	MAG647	31	2155435	356635	165724	69530	99.54	0
PD	PD86	bin20	MAG3341	41	2165394	214665	103978	52814	99.54	0.23
PD	PD87	bin44	MAG2059	51	2271927	168849	76378	44548	99.54	1.15
PD	PD4	bin6	MAG2146	66	2852108	173436	67205	43214	99.53	0
PD	PD85	bin18	MAG1729	141	2201739	102945	26373	15615	99.53	0.94
PD	PD1	bin10	MAG3308	24	2207093	383585	271242	91962	99.52	0.96
PD	PD28	bin50	MAG2163	86	3011304	159426	55102	35015	99.52	1.93
PD	PD31	bin11	MAG2603	103	2722045	178534	52446	26428	99.52	0.48
PD	PD45	bin10	MAG1322	102	3193813	231081	54929	31312	99.52	0.97
PD	PD47	bin14	MAG1867	90	3027093	140611	77771	33634	99.52	0
PD	PD5	bin27	MAG2553	59	2603014	151100	91950	44119	99.52	0.12
PD	PD69	bin15	MAG2512	111	2823063	144097	39467	25433	99.52	0.62
PD	PD71	bin24	MAG2708	60	2712580	146998	79282	45210	99.52	0.24
PD	PD77	bin40	MAG1047	88	2796247	176570	53850	31776	99.52	0.48
PD	PD85	bin6	MAG1392	66	2869835	274115	91760	43482	99.52	0
PD	PD35	bin16	MAG832	67	1893120	242130	67878	28256	99.48	0
PD	PD4	bin9	MAG2370	77	2187708	133904	46743	28412	99.48	0.35
PD	PD76	bin1	MAG2045	50	1870225	282890	88421	37405	99.48	0
PD	PD18	bin12	MAG964	92	1857690	77789	36183	20192	99.46	0
PD	PD50	bin14	MAG2872	78	2204436	115248	45013	28262	99.46	0.89
PD	PD22	bin20	MAG2835	314	2451351	45104	12587	7807	99.44	3.37
PD	PD9	bin59	MAG2388	89	3086931	137254	39636	34685	99.44	0.48
PD	PD100	bin25	MAG220	113	2586692	84800	44580	22891	99.42	0.41
PD	PD26	bin3	MAG1993	75	1962815	132301	32345	26171	99.4	0.1
PD	PD8	bin5	MAG872	138	2877543	133247	49716	20852	99.36	0.48
PD	PD99	bin16	MAG2766	62	2637954	150648	80583	42548	99.36	0
PD	PD91	bin35	MAG736	56	4542574	308675	156508	81117	99.34	0.08
PD	PD100	bin16	MAG1266	74	2084789	320888	144652	28173	99.33	2.01
PD	PD13	bin32	MAG939	319	3248963	84888	21980	10185	99.33	1.55
PD	PD23	bin20	MAG961	46	2057269	179499	77018	44723	99.33	1.01
PD	PD26	bin19	MAG2398	235	3119058	131322	36493	13273	99.33	1.24

PD	PD34	bin73	MAG1549	315	3430186	105722	24456	10889	99.33	5.37
PD	PD35	bin44	MAG2158	124	2535140	147281	38856	20445	99.33	0
PD	PD36	bin46	MAG706	51	2090257	155796	68447	40985	99.33	0.67
PD	PD4	bin14	MAG1602	57	2736321	445730	110101	48006	99.33	0.89
PD	PD43	bin30	MAG2337	194	2874818	134786	50255	14819	99.33	7.38
PD	PD44	bin37	MAG1400	75	2700897	116188	52865	36012	99.33	1.68
PD	PD45	bin28	MAG1735	69	2419711	179112	76321	35068	99.33	0.67
PD	PD46	bin31	MAG1190	119	2824458	144793	51097	23735	99.33	2.01
PD	PD46	bin54	MAG619	54	2733847	227537	100361	50627	99.33	1.34
PD	PD47	bin36	MAG914	83	2746846	138601	50566	33095	99.33	0.67
PD	PD48	bin34	MAG281	138	2502078	128368	33496	18131	99.33	3.36
PD	PD5	bin30	MAG1000	38	2792264	436017	135538	73481	99.33	0
PD	PD53	bin2	MAG2030	33	2007540	218067	131059	60835	99.33	0.84
PD	PD56	bin29	MAG2519	78	1967957	170464	50183	25230	99.33	2.01
PD	PD61	bin4	MAG2561	313	3303950	138170	41772	10556	99.33	5.61
PD	PD62	bin27	MAG2171	52	2831081	403049	124328	54444	99.33	0.24
PD	PD66	bin20	MAG45	69	2105514	238183	80019	30515	99.33	1.01
PD	PD70	bin24	MAG1092	119	2631638	111382	33796	22115	99.33	2.35
PD	PD75	bin24	MAG1269	119	2928922	168391	42040	24613	99.33	2.01
PD	PD76	bin10	MAG559	72	2679199	226181	60137	37211	99.33	2.01
PD	PD78	bin1	MAG2891	44	2346585	175611	104229	53331	99.33	0.67
PD	PD78	bin13	MAG790	114	2552614	169946	37047	22391	99.33	0.69
PD	PD8	bin1	MAG2598	89	2038562	318541	50361	22905	99.33	1.34
PD	PD81	bin5	MAG1304	52	3228780	311306	149571	62092	99.33	5.37
PD	PD82	bin39	MAG2698	71	2410768	244269	61954	33954	99.33	0
PD	PD92	bin57	MAG2098	36	2070817	300142	98483	57523	99.33	1.01
PD	PD94	bin46	MAG2688	33	2662390	391936	101420	80678	99.33	0
PD	PD95	bin10	MAG2576	30	2030757	219838	114036	67692	99.33	1.01
PD	PD29	bin6	MAG3356	50	2220427	199555	92507	44409	99.32	0.25
PD	PD32	bin55	MAG2590	78	2407941	148890	52278	30871	99.32	0
PD	PD99	bin17	MAG2011	98	2732058	143300	46178	27878	99.32	1.06
PD	PD86	bin12	MAG3109	168	4230235	152879	47485	25180	99.31	4.34
PD	PD12	bin54	MAG227	149	2927700	141876	30904	19649	99.3	1.17

PD	PD2	bin1	MAG1842	189	2880086	98456	26986	15239	99.3	2.56
PD	PD27	bin10	MAG3419	205	2918927	61794	21172	14239	99.3	2.21
PD	PD28	bin8	MAG1197	188	2987943	94281	28206	15893	99.3	1.17
PD	PD32	bin33	MAG387	158	2912230	111946	29882	18432	99.3	1.17
PD	PD66	bin7	MAG222	154	2989718	98747	30121	19414	99.3	1.17
PD	PD97	bin73	MAG945	130	2894473	98784	31338	22265	99.3	1.17
PD	PD35	bin5	MAG2196	61	2635515	177465	67101	43205	99.28	0
PD	PD59	bin16	MAG2746	60	3183157	393222	112251	53053	99.28	0.48
PD	PD91	bin3	MAG654	82	2844212	162200	65991	34686	99.28	0.24
PD	PD98	bin47	MAG7	70	2674964	133489	61068	38214	99.28	0.24
PD	PD85	bin16	MAG489	203	3548988	94284	25558	17483	99.26	0.67
PD	PD67	bin4	MAG1056	50	2178190	299153	89516	43564	99.24	0.23
PD	PD75	bin44	MAG838	252	6796232	335566	84348	26969	99.24	0.6
PD	PD20	bin20	MAG1720	23	1871397	625378	207104	81365	99.22	0.09
PD	PD85	bin1	MAG2702	17	1843871	625095	156451	108463	99.22	0.84
PD	PD82	bin55	MAG1912	304	2439339	35928	11702	8024	99.21	0.68
PD	PD9	bin61	MAG581	208	3310554	250406	62368	15916	99.21	1.93
PD	PD71	bin10	MAG2714	37	2102843	220182	94108	56834	99.2	0.72
PD	PD81	bin17	MAG840	259	3189273	214928	64991	12314	99.2	0.81
PD	PD2	bin6	MAG2286	40	1740151	358326	86477	43504	99.19	0.09
PD	PD29	bin46	MAG2057	68	1910276	182928	43841	28092	99.19	1.08
PD	PD45	bin47	MAG707	64	2259846	104519	61870	35310	99.19	0.81
PD	PD51	bin35	MAG2287	207	3218321	242333	31844	15547	99.19	2.74
PD	PD62	bin44	MAG1120	42	2186051	221872	86260	52049	99.19	0
PD	PD70	bin38	MAG812	59	2314642	270252	81461	39231	99.19	0
PD	PD20	bin49	MAG2758	41	2092950	206186	105832	51048	99.18	1.09
PD	PD94	bin53	MAG89	32	1860408	195510	122530	58138	99.18	0
PD	PD44	bin30	MAG2018	47	2289630	196767	82918	48716	99.16	1.34
PD	PD23	bin40	MAG1983	131	2114978	174075	38049	16145	99.15	0.68
PD	PD46	bin82	MAG643	191	2394077	83790	20022	12534	99.15	1.69
PD	PD64	bin32	MAG731	117	2943692	130873	34624	25160	99.13	0.72
PD	PD39	bin20	MAG2793	203	4450007	359967	92056	21921	99.12	0.07
PD	PD42	bin21	MAG2704	94	2153881	137139	34350	22914	99.12	2.02

PD	PD44	bin60	MAG2386	90	2460770	219532	73546	27342	99.12	7.26
PD	PD78	bin32	MAG1921	89	3016279	168736	63917	33891	99.12	1.21
PD	PD13	bin54	MAG2306	155	2609135	74331	28906	16833	99.11	0.72
PD	PD19	bin12	MAG2747	77	2114340	103538	41835	27459	99.09	0.06
PD	PD69	bin10	MAG1191	198	4525877	117954	48811	22858	99.07	0.55
PD	PD10	bin30	MAG2727	161	2711503	71197	31480	16842	99.06	0.47
PD	PD18	bin58	MAG2660	44	1791805	193180	75134	40723	99.06	0
PD	PD31	bin35	MAG1448	168	2066651	63821	19402	12301	99.06	0.94
PD	PD37	bin49	MAG3404	129	3375281	168715	51714	26165	99.06	0.94
PD	PD6	bin34	MAG1060	105	2629020	286325	42160	25038	99.06	0
PD	PD69	bin57	MAG1022	124	2661412	123483	32442	21463	99.06	0
PD	PD71	bin61	MAG3134	185	2723298	87666	23871	14721	99.06	0.99
PD	PD8	bin22	MAG2572	181	2790302	86994	25823	15416	99.06	0
PD	PD97	bin60	MAG292	162	2682831	92187	23884	16561	99.06	0
PD	PD100	bin21	MAG433	48	2545955	250262	71100	53041	99.04	0.48
PD	PD91	bin19	MAG401	29	2177143	229727	157192	75074	99.04	0
PD	PD98	bin48	MAG2687	33	2664687	433183	123038	80748	99.04	0
PD	PD12	bin50	MAG1801	85	2105274	106470	40606	24768	99.03	0.09
PD	PD40	bin49	MAG1872	108	2648644	322328	46784	24524	99.03	0.48
PD	PD14	bin19	MAG627	108	2628432	168197	33581	24337	98.99	0
PD	PD24	bin8	MAG3490	90	2516900	176303	46699	27966	98.99	0
PD	PD37	bin35	MAG2657	148	2149793	334898	61821	14526	98.98	0.45
PD	PD39	bin43	MAG2913	337	2753239	61587	11878	8170	98.98	0.68
PD	PD67	bin26	MAG1042	311	2819555	53188	13159	9066	98.98	0.68
PD	PD67	bin10	MAG26	194	5821347	201632	71237	30007	98.98	1.05
PD	PD44	bin3	MAG2571	195	2517864	146306	24004	12912	98.96	3.58
PD	PD13	bin65	MAG3220	278	2944817	54325	15800	10593	98.95	1.17
PD	PD18	bin8	MAG340	57	2954226	171969	65656	51829	98.95	0.63
PD	PD42	bin12	MAG670	152	2803226	158608	43779	18442	98.95	0.63
PD	PD83	bin26	MAG2461	47	2858789	250145	110236	60825	98.95	0.63
PD	PD97	bin12	MAG746	48	3127491	219432	149911	65156	98.92	0.48
PD	PD10	bin2	MAG251	53	2243478	199052	71231	42330	98.9	1.21
PD	PD26	bin7	MAG2858	147	2525849	160212	39511	17183	98.85	0.25

PD	PD44	bin43	MAG760	70	2835995	142688	68887	40514	98.83	0.7
PD	PD48	bin55	MAG2700	90	2858095	156419	47171	31757	98.8	0.72
PD	PD72	bin11	MAG3318	43	3401506	391491	133684	79105	98.8	0.48
PD	PD3	bin13	MAG3018	252	3659089	131912	29509	14520	98.79	0.48
PD	PD81	bin30	MAG374	127	3219715	193867	82028	25352	98.79	6.89
PD	PD19	bin8	MAG480	165	3698410	124496	42457	22415	98.73	0.95
PD	PD47	bin53	MAG234	277	2361286	57006	12930	8524	98.73	0.16
PD	PD95	bin28	MAG2138	183	3305156	113458	37470	18061	98.73	0.95
PD	PD28	bin41	MAG321	287	3412647	134182	46634	11891	98.7	1.86
PD	PD20	bin8	MAG2681	31	2079951	248588	109281	67095	98.67	0.08
PD	PD10	bin26	MAG610	172	2701654	65009	25526	15707	98.66	2.24
PD	PD17	bin11	MAG1212	157	1940383	96674	18689	12359	98.66	0.67
PD	PD17	bin4	MAG2691	53	2195887	206472	63007	41432	98.66	0
PD	PD18	bin3	MAG1089	179	2564083	106607	23967	14324	98.66	0
PD	PD19	bin16	MAG1432	45	2150839	297728	88030	47796	98.66	0
PD	PD19	bin35	MAG3455	66	2185645	209109	62752	33116	98.66	0.34
PD	PD24	bin21	MAG1710	30	2763585	256022	155499	92120	98.66	0.34
PD	PD26	bin18	MAG1004	107	2900077	151288	51607	27104	98.66	0.75
PD	PD29	bin63	MAG2580	55	2720581	194120	77575	49465	98.66	0
PD	PD32	bin81	MAG755	35	2728653	320834	126068	77962	98.66	0
PD	PD36	bin31	MAG3160	52	1882394	336849	122613	36200	98.66	0
PD	PD39	bin1	MAG1025	249	3417051	105054	28454	13723	98.66	2.18
PD	PD4	bin2	MAG2890	32	1797147	180889	101684	56161	98.66	0
PD	PD44	bin38	MAG3100	68	1943017	217201	48297	28574	98.66	0.34
PD	PD45	bin49	MAG1384	117	2807424	122420	41340	23995	98.66	0.19
PD	PD45	bin2	MAG3291	140	2564505	469273	166375	18318	98.66	5
PD	PD47	bin4	MAG330	27	1838898	234521	105001	68107	98.66	0
PD	PD51	bin4	MAG90	175	3210116	119101	34343	18344	98.66	1.41
PD	PD57	bin53	MAG1219	39	2745450	313038	102856	70396	98.66	0
PD	PD61	bin16	MAG2351	47	1814630	148845	84553	38609	98.66	0.67
PD	PD62	bin30	MAG796	101	2125195	91036	38271	21042	98.66	0
PD	PD64	bin15	MAG3024	76	2167122	303188	139222	28515	98.66	0
PD	PD64	bin8	MAG3357	47	2608049	162669	78915	55490	98.66	0.04

PD	PD66	bin4	MAG1221	47	1845203	142453	63691	39260	98.66	0
PD	PD68	bin54	MAG3248	71	2062325	113589	49152	29047	98.66	0.67
PD	PD7	bin23	MAG200	86	2806727	272204	50783	32636	98.66	0.67
PD	PD71	bin39	MAG1109	69	2732352	223783	91924	39599	98.66	0.67
PD	PD76	bin39	MAG1061	115	2526733	100864	42224	21972	98.66	0.67
PD	PD77	bin34	MAG1422	75	2034031	139065	50110	27120	98.66	0.34
PD	PD77	bin20	MAG3307	133	2578861	117663	35750	19390	98.66	1.34
PD	PD81	bin6	MAG2067	98	2194160	221555	142256	22389	98.66	1.34
PD	PD82	bin46	MAG1065	95	2870008	185143	46832	30211	98.66	1.34
PD	PD85	bin9	MAG2944	166	2510643	72953	23389	15124	98.66	0.79
PD	PD87	bin28	MAG1640	106	2658965	118069	44764	25085	98.66	0
PD	PD94	bin65	MAG2345	38	1972643	212116	88855	51912	98.66	1.01
PD	PD95	bin16	MAG2751	72	2322101	161149	52227	32251	98.66	0.67
PD	PD11	bin28	MAG2236	367	2790773	53500	11361	7604	98.64	0.87
PD	PD17	bin15	MAG1598	373	2596436	44305	11055	6961	98.64	0.68
PD	PD37	bin2	MAG446	121	2434283	197856	31172	20118	98.64	0.67
PD	PD45	bin45	MAG148	315	2409164	35585	11022	7648	98.64	0.68
PD	PD6	bin16	MAG2834	288	2305904	36910	11064	8007	98.64	0.91
PD	PD85	bin12	MAG2754	32	1881157	318967	83830	58786	98.64	0
PD	PD23	bin18	MAG741	150	2661056	184796	34008	17740	98.63	1.37
PD	PD47	bin48	MAG1265	93	2722314	278054	55933	29272	98.63	0
PD	PD14	bin10	MAG1332	142	2921919	134694	33981	20577	98.6	1.17
PD	PD46	bin1	MAG797	258	2946508	65225	15654	11421	98.6	0.47
PD	PD62	bin16	MAG3139	170	2963337	61560	26608	17431	98.6	1.17
PD	PD70	bin7	MAG2422	34	1408035	197715	58035	41413	98.58	0.24
PD	PD43	bin3	MAG28	100	2165919	169277	48208	21659	98.56	0.73
PD	PD94	bin34	MAG378	236	4610144	139070	29898	19535	98.56	0.59
PD	PD69	bin25	MAG2450	111	2513474	109090	46307	22644	98.55	0.48
PD	PD71	bin36	MAG604	107	2898452	228671	40724	27088	98.55	0.98
PD	PD4	bin22	MAG3458	135	2536702	72980	30667	18790	98.54	0
PD	PD28	bin37	MAG660	172	3282662	160842	37129	19085	98.52	0.72
PD	PD20	bin23	MAG195	208	2586024	55709	18455	12433	98.5	1.37
PD	PD50	bin12	MAG1516	87	2117054	144705	59400	24334	98.49	0.68

PD	PD94	bin14	MAG3354	212	2490554	124225	19285	11748	98.44	1.19
PD	PD37	bin8	MAG2326	142	2583365	111913	38052	18193	98.43	2.68
PD	PD61	bin49	MAG1347	351	4640734	96274	19603	13221	98.43	1.91
PD	PD2	bin35	MAG2316	88	1722286	69792	32304	19571	98.39	1.88
PD	PD27	bin6	MAG3185	205	2362837	76343	16566	11526	98.39	1.61
PD	PD29	bin15	MAG1537	95	1676023	53570	24746	17642	98.39	1.08
PD	PD68	bin57	MAG1777	66	2588967	174973	60675	39227	98.39	0.89
PD	PD73	bin28	MAG1312	94	2179744	158918	58876	23189	98.39	1.61
PD	PD77	bin10	MAG2085	90	2178053	155520	45388	24201	98.39	2.58
PD	PD94	bin66	MAG2515	160	1627486	48762	14980	10172	98.39	2
PD	PD66	bin17	MAG1114	155	4658972	226837	58379	30058	98.38	0.4
PD	PD48	bin13	MAG690	131	2171386	145389	38408	16575	98.37	0.63
PD	PD41	bin53	MAG2652	205	2301123	77153	17531	11225	98.33	0
PD	PD8	bin52	MAG1962	198	2435642	109855	32700	12301	98.33	1.87
PD	PD46	bin10	MAG3072	38	2964673	673614	323761	78018	98.32	0.67
PD	PD48	bin31	MAG434	88	2512836	151655	43679	28555	98.32	0.67
PD	PD73	bin73	MAG1553	203	2489618	80500	17850	12264	98.32	2.35
PD	PD45	bin35	MAG3187	119	3380916	280239	104367	28411	98.31	3.13
PD	PD75	bin9	MAG179	116	2872058	101031	38125	24759	98.31	0.06
PD	PD70	bin22	MAG727	320	2419698	40112	10082	7562	98.3	0.7
PD	PD68	bin31	MAG2274	109	3061303	176459	37683	28085	98.29	0.72
PD	PD19	bin18	MAG2046	102	3696468	221551	69558	36240	98.25	0
PD	PD25	bin34	MAG1651	236	2931060	56141	19958	12420	98.25	1.17
PD	PD4	bin12	MAG1372	57	2925977	225474	85942	51333	98.25	0
PD	PD25	bin6	MAG123	62	2343267	153839	53749	37795	98.24	0.48
PD	PD3	bin27	MAG1289	185	2485848	67075	18424	13437	98.24	0.84
PD	PD97	bin44	MAG16	239	4019206	125870	49752	16817	98.24	1.05
PD	PD70	bin53	MAG1833	85	2003501	90472	34248	23571	98.23	0.35
PD	PD3	bin5	MAG2424	57	1791182	156947	50914	31424	98.2	1.5
PD	PD77	bin26	MAG1055	148	3039770	98344	29572	20539	98.2	1.68
PD	PD76	bin15	MAG2366	27	2359894	456362	217885	87403	98.19	0.72
PD	PD58	bin10	MAG1204	208	1934570	38422	12626	9301	98.18	0.35
PD	PD86	bin11	MAG1869	245	4345316	72519	27504	17736	98.18	0.88

PD	PD28	bin55	MAG3475	109	3442162	127657	53005	31579	98.16	1.99
PD	PD68	bin26	MAG76	111	3387853	170456	63498	30521	98.14	0.34
PD	PD10	bin18	MAG1594	102	1915201	124521	29099	18776	98.12	0.12
PD	PD10	bin43	MAG129	257	3811542	96514	23460	14831	98.11	0.94
PD	PD44	bin44	MAG2649	264	2520287	47933	14703	9547	98.11	0.94
PD	PD69	bin1	MAG2822	161	2144898	124483	26376	13322	98.1	1.59
PD	PD97	bin70	MAG1417	55	2716708	217338	76395	49395	98.1	0.21
PD	PD11	bin1	MAG2668	125	2265146	84122	33578	18121	98.07	0.62
PD	PD76	bin41	MAG449	198	2014480	181677	33854	10174	98.06	3.65
PD	PD29	bin77	MAG1121	259	3019781	69128	23256	11659	98.04	0
PD	PD57	bin3	MAG2140	149	2384544	68064	22522	16004	98.04	0.88
PD	PD92	bin17	MAG3301	113	2139985	124124	39047	18938	98.04	0.81
PD	PD97	bin48	MAG2065	376	3027300	100180	16477	8051	98.04	1.48
PD	PD71	bin37	MAG2524	71	2977029	213498	91906	41930	98.01	0.02
PD	PD1	bin7	MAG1584	58	2677922	217416	77715	46171	97.99	0.84
PD	PD16	bin5	MAG800	33	1786384	170840	90027	54133	97.99	0.67
PD	PD18	bin46	MAG681	65	2600969	171836	61691	40015	97.99	0.11
PD	PD3	bin49	MAG2010	72	2039941	157345	85411	28333	97.99	0.34
PD	PD36	bin29	MAG878	73	2644262	164167	56966	36223	97.99	0
PD	PD37	bin3	MAG2335	71	1911789	147096	49731	26927	97.99	0
PD	PD60	bin10	MAG1922	76	2773317	180443	64717	36491	97.99	2.01
PD	PD66	bin1	MAG209	237	2411569	80348	15416	10175	97.99	1.34
PD	PD69	bin7	MAG20	86	2012888	147239	42860	23406	97.99	0.67
PD	PD87	bin11	MAG335	41	2698813	200873	100300	65825	97.99	0
PD	PD9	bin17	MAG861	248	2721997	78415	18149	10976	97.99	3.58
PD	PD100	bin6	MAG1624	69	2697463	295081	91896	39094	97.96	0
PD	PD12	bin77	MAG1359	50	2673102	202718	113802	53462	97.96	1.25
PD	PD16	bin34	MAG1558	46	2638070	291508	94349	57349	97.96	0.71
PD	PD27	bin36	MAG2259	42	2703495	349086	175890	64369	97.96	0.17
PD	PD46	bin59	MAG1393	76	3097558	203425	88384	40757	97.96	0.68
PD	PD5	bin32	MAG2100	56	2680051	230069	99845	47858	97.96	0
PD	PD59	bin21	MAG490	51	2999062	271233	94393	58805	97.96	0
PD	PD64	bin84	MAG649	49	2739848	283581	100184	55915	97.96	0.68

PD	PD67	bin3	MAG1090	44	2673846	238309	118569	60769	97.96	0
PD	PD7	bin32	MAG778	47	3041033	460026	130854	64703	97.96	0
PD	PD72	bin25	MAG2495	69	3034296	304445	131662	43975	97.96	0.68
PD	PD77	bin39	MAG2178	302	2370599	54178	11862	7850	97.96	0.68
PD	PD97	bin58	MAG1649	340	2850969	66945	11760	8385	97.96	0.68
PD	PD99	bin46	MAG6	60	2671720	192246	60667	44529	97.96	0
PD	PD11	bin32	MAG334	60	2307452	149214	57942	38458	97.95	0
PD	PD57	bin70	MAG1019	61	2548567	308246	74152	41780	97.95	0.68
PD	PD11	bin12	MAG2807	445	3852278	76606	11851	8657	97.93	0.59
PD	PD31	bin60	MAG2895	86	2200426	208481	48450	25586	97.93	1.61
PD	PD32	bin78	MAG486	477	2219987	25851	6073	4654	97.92	0.68
PD	PD82	bin50	MAG1587	299	2741952	60450	13721	9170	97.92	0.36
PD	PD19	bin21	MAG1140	150	2156294	55882	24277	14375	97.91	1.42
PD	PD3	bin31	MAG1850	112	2714589	179760	48661	24237	97.9	0.84
PD	PD5	bin42	MAG667	197	2583098	139103	37443	13112	97.9	1.1
PD	PD71	bin42	MAG624	199	2922676	100864	26107	14687	97.9	1.86
PD	PD1	bin3	MAG1968	43	2283467	311475	117279	53104	97.87	0
PD	PD9	bin45	MAG3158	70	2558578	158062	52727	36551	97.86	0.17
PD	PD69	bin56	MAG171	309	2378535	36912	11255	7698	97.85	2.38
PD	PD95	bin19	MAG1232	225	2251681	86106	13775	10007	97.85	0
PD	PD50	bin49	MAG2452	121	2207906	79073	29557	18247	97.84	0.06
PD	PD34	bin58	MAG951	321	2071044	31188	10707	6452	97.79	3.34
PD	PD75	bin45	MAG1234	53	2012116	123181	59754	37964	97.76	0.89
PD	PD26	bin1	MAG202	129	2404051	94342	26208	18636	97.74	0.34
PD	PD68	bin19	MAG2742	71	2989833	280712	72707	42110	97.74	0
PD	PD42	bin4	MAG1336	167	2209759	67097	20991	13232	97.71	1.99
PD	PD59	bin51	MAG3451	111	6268785	435301	88492	56476	97.7	1.72
PD	PD81	bin40	MAG3394	53	2864668	221753	77483	54050	97.68	0.63
PD	PD9	bin60	MAG1388	155	2173456	66984	18687	14022	97.67	0.12
PD	PD32	bin59	MAG2556	110	2572133	159738	40985	23383	97.65	0
PD	PD5	bin29	MAG535	90	2533230	90718	40021	28147	97.65	0
PD	PD67	bin12	MAG2684	384	2454521	41226	8803	6392	97.65	2.13
PD	PD95	bin21	MAG172	335	2970745	83081	15916	8868	97.65	2.87

PD	PD24	bin16	MAG1668	82	2864921	165960	49729	34938	97.64	0.68
PD	PD22	bin9	MAG2166	107	2488917	136332	33709	23261	97.63	0.59
PD	PD29	bin1	MAG2438	129	3188724	113379	38832	24719	97.63	0
PD	PD47	bin13	MAG2334	175	2811851	74989	25438	16068	97.61	1.69
PD	PD22	bin8	MAG2638	41	2860324	342745	195034	69764	97.6	0
PD	PD19	bin27	MAG291	103	2961621	146094	46284	28754	97.58	1.61
PD	PD60	bin30	MAG938	125	3344998	105106	50699	26760	97.58	1.5
PD	PD72	bin34	MAG2488	373	1716418	23546	5900	4602	97.58	2.11
PD	PD85	bin35	MAG3044	69	2044387	168955	43256	29629	97.58	0
PD	PD44	bin8	MAG3225	154	2469089	76230	22721	16033	97.56	0
PD	PD77	bin19	MAG2709	183	2282017	166180	30363	12470	97.56	0.87
PD	PD21	bin8	MAG1182	36	1946330	188086	87894	54065	97.55	0
PD	PD43	bin39	MAG1545	38	2677054	269882	146144	70449	97.55	1.05
PD	PD50	bin42	MAG1082	224	2518439	57563	17093	11243	97.47	0.48
PD	PD76	bin9	MAG1066	183	2226331	81421	18008	12166	97.43	1.12
PD	PD100	bin22	MAG2235	188	4209159	189138	35095	22389	97.37	0
PD	PD25	bin41	MAG2667	81	2180436	103619	49823	26919	97.36	1.77
PD	PD48	bin30	MAG748	61	2476660	235035	65416	40601	97.36	0
PD	PD70	bin59	MAG2995	181	2372325	61861	18677	13107	97.36	1.22
PD	PD7	bin13	MAG2197	136	6778762	245993	77588	49844	97.35	4.41
PD	PD82	bin49	MAG1635	119	1839501	66100	26395	15458	97.35	1.55
PD	PD10	bin37	MAG2068	114	2721440	124098	36741	23872	97.32	1.68
PD	PD19	bin17	MAG2824	163	2485044	90945	28833	15246	97.32	0.67
PD	PD20	bin31	MAG1503	227	2556611	79018	21310	11263	97.32	1.12
PD	PD29	bin52	MAG1268	96	2020076	72683	32169	21042	97.32	0.34
PD	PD29	bin60	MAG2983	56	1953244	229833	52845	34879	97.32	0.34
PD	PD35	bin39	MAG3407	53	2054253	130374	54122	38759	97.32	0
PD	PD4	bin43	MAG2471	325	2230004	52791	11684	6862	97.32	0.84
PD	PD42	bin18	MAG3164	213	2667674	74068	19252	12524	97.32	0.56
PD	PD44	bin53	MAG1328	17	1873081	854166	358638	110181	97.32	0
PD	PD57	bin11	MAG383	83	2145444	148508	44617	25849	97.32	0.34
PD	PD62	bin15	MAG1860	25	1871050	328797	158259	74842	97.32	0.22
PD	PD64	bin83	MAG2060	283	3394047	85385	33872	11993	97.32	0.99

PD	PD68	bin36	MAG2820	15	1869485	243500	157102	124632	97.32	0
PD	PD72	bin22	MAG2939	114	2707994	165048	36096	23754	97.32	0
PD	PD77	bin33	MAG717	20	1906804	678304	175217	95340	97.32	0
PD	PD9	bin3	MAG3194	43	1906871	172967	74564	44346	97.32	0.67
PD	PD92	bin38	MAG1893	112	5185740	453823	83900	46301	97.31	0.44
PD	PD44	bin28	MAG1864	170	3589163	140675	39711	21113	97.3	1.35
PD	PD76	bin31	MAG473	190	2340506	92926	23214	12318	97.3	0.84
PD	PD28	bin35	MAG973	59	2776762	238296	89387	47064	97.28	0.68
PD	PD29	bin13	MAG1684	68	3070911	192675	79467	45160	97.28	0
PD	PD53	bin9	MAG112	305	2319630	38224	10831	7605	97.28	0.68
PD	PD57	bin46	MAG3448	71	2630540	256226	69282	37050	97.28	0
PD	PD86	bin1	MAG2034	324	2483367	34993	11280	7665	97.28	0.68
PD	PD9	bin15	MAG319	398	2332968	30624	8627	5862	97.28	0.68
PD	PD95	bin23	MAG1663	123	2912214	203867	48012	23677	97.28	0.82
PD	PD16	bin19	MAG3349	50	2254466	294372	81601	45089	97.27	0
PD	PD27	bin37	MAG954	49	2291763	270773	136219	46771	97.27	0.34
PD	PD70	bin25	MAG2382	43	2171358	208870	124322	50497	97.27	0
PD	PD75	bin20	MAG420	285	3062955	94513	20529	10747	97.27	1.49
PD	PD94	bin31	MAG887	44	2187148	149675	64726	49708	97.27	0
PD	PD98	bin54	MAG208	15	2137221	336061	160541	142481	97.27	0.34
PD	PD17	bin23	MAG196	55	2506877	241864	90054	45580	97.26	0
PD	PD6	bin32	MAG2744	78	2454347	214068	54839	31466	97.26	0
PD	PD47	bin5	MAG1242	399	2319736	25433	7451	5814	97.24	1.42
PD	PD69	bin21	MAG708	185	2984099	112749	25427	16130	97.2	0.93
PD	PD46	bin83	MAG669	59	1966683	193726	53262	33334	97.18	2.12
PD	PD51	bin31	MAG1180	110	1966799	81942	27698	17880	97.18	0
PD	PD56	bin52	MAG899	70	2002860	181242	48170	28612	97.18	1.4
PD	PD58	bin5	MAG3358	66	2019505	117640	52488	30599	97.18	1.4
PD	PD94	bin5	MAG1837	43	1973098	225355	73099	45886	97.18	1.4
PD	PD98	bin32	MAG1896	51	2061924	194124	72896	40430	97.18	1.4
PD	PD19	bin4	MAG2788	112	2691883	180964	45335	24035	97.17	0
PD	PD40	bin38	MAG184	184	2591521	133977	26176	14084	97.15	0.63
PD	PD7	bin18	MAG2972	227	3935844	112099	24788	17339	97.13	0

PD	PD42	bin27	MAG3116	292	2155863	38930	11099	7383	97.11	3.02
PD	PD64	bin94	MAG1106	591	2174915	19481	4735	3680	97.11	0.68
PD	PD19	bin13	MAG2160	55	2604656	228005	84175	47357	97.1	0
PD	PD50	bin40	MAG2314	41	2228237	390702	82145	54347	97.09	0
PD	PD36	bin48	MAG2336	123	2999510	178813	37541	24386	97.05	0
PD	PD37	bin14	MAG1852	78	2852513	145113	53176	36571	97.05	0.63
PD	PD40	bin48	MAG1799	829	4892139	55184	8840	5901	97.02	7.49
PD	PD44	bin9	MAG365	50	2167051	143924	77991	43341	97.01	0
PD	PD11	bin31	MAG1348	107	4448772	298361	79366	41577	96.99	0
PD	PD76	bin48	MAG1153	105	1761504	100235	31792	16776	96.99	0
PD	PD100	bin13	MAG1263	67	3034165	276346	76835	45286	96.98	0
PD	PD32	bin8	MAG304	282	2548192	74156	13705	9036	96.98	1.68
PD	PD46	bin18	MAG2241	89	3320753	226374	48020	37312	96.98	0.85
PD	PD77	bin31	MAG231	435	4027879	91299	24503	9259	96.98	2.68
PD	PD87	bin46	MAG136	98	2471162	136238	43590	25216	96.98	0
PD	PD1	bin30	MAG1812	207	3026194	79013	26307	14619	96.94	3.13
PD	PD53	bin34	MAG1012	70	2352215	136098	57625	33603	96.94	0.48
PD	PD1	bin17	MAG730	93	5578629	368545	101761	59985	96.92	0
PD	PD72	bin52	MAG3000	78	4371429	264011	91488	56044	96.92	0
PD	PD23	bin43	MAG1027	131	3017513	150307	36271	23034	96.86	0
PD	PD23	bin17	MAG1088	89	2882505	328511	85391	32388	96.86	1.21
PD	PD21	bin5	MAG2078	45	2581373	301995	101953	57364	96.84	0
PD	PD25	bin20	MAG925	67	2017829	152108	61785	30117	96.83	1.4
PD	PD71	bin60	MAG634	147	3272011	212494	46395	22259	96.83	0.13
PD	PD1	bin24	MAG1325	82	3255717	198906	67414	39704	96.77	0.81
PD	PD22	bin7	MAG1340	192	2059773	70409	16195	10728	96.77	2.65
PD	PD40	bin12	MAG1728	69	2368725	139847	82148	34329	96.77	0.81
PD	PD41	bin20	MAG1934	57	1934576	111014	46968	33940	96.77	0.81
PD	PD41	bin65	MAG2210	219	2095597	54277	13855	9569	96.77	2.42
PD	PD57	bin50	MAG169	130	3039885	146292	39825	23384	96.77	1.88
PD	PD59	bin31	MAG113	136	2021657	75462	24240	14865	96.77	1.25
PD	PD70	bin5	MAG164	186	2943275	93656	23489	15824	96.77	1.08
PD	PD75	bin28	MAG710	232	3137561	69792	21983	13524	96.77	0

PD	PD14	bin28	MAG1277	311	3360151	48690	14336	10804	96.76	0.74
PD	PD70	bin17	MAG518	47	2372024	339609	102101	50469	96.73	1.17
PD	PD83	bin33	MAG58	287	2012427	41981	11376	7012	96.73	0.12
PD	PD16	bin26	MAG2928	398	2223500	39687	8034	5587	96.72	2.82
PD	PD46	bin47	MAG2538	403	2460872	34051	8193	6106	96.7	0.47
PD	PD40	bin37	MAG1456	279	4699255	119707	30887	16843	96.69	2.46
PD	PD25	bin15	MAG2577	28	1886701	365565	172403	67382	96.64	0
PD	PD29	bin49	MAG3330	114	2348126	133610	32388	20598	96.64	0
PD	PD29	bin68	MAG3509	178	2507504	70149	21189	14087	96.64	0.11
PD	PD41	bin62	MAG3462	97	1896540	94406	34121	19552	96.64	0.34
PD	PD46	bin43	MAG3250	108	2017181	150164	31882	18678	96.64	1.42
PD	PD46	bin52	MAG628	143	2528223	93455	29994	17680	96.64	0
PD	PD57	bin45	MAG1875	56	2262596	272337	101095	40404	96.64	0
PD	PD6	bin2	MAG1938	33	1874339	244391	103314	56798	96.64	0
PD	PD68	bin35	MAG1622	116	2463206	167367	31585	21235	96.64	1.01
PD	PD70	bin32	MAG1441	84	1879792	121980	53520	22378	96.64	1.01
PD	PD73	bin24	MAG217	173	1790530	105663	17252	10350	96.64	1.85
PD	PD75	bin6	MAG865	14	1838579	354444	240250	131327	96.64	0
PD	PD92	bin1	MAG617	82	2479867	155868	45186	30242	96.64	0
PD	PD94	bin44	MAG2692	35	1925421	291515	157294	55012	96.64	0
PD	PD94	bin49	MAG3266	325	2182050	38947	9107	6714	96.64	0.48
PD	PD82	bin53	MAG946	94	2718752	392578	66176	28923	96.62	0
PD	PD46	bin21	MAG571	314	2249886	37096	10469	7165	96.61	0
PD	PD1	bin14	MAG1084	94	2421082	102828	54179	25756	96.6	0
PD	PD10	bin25	MAG716	540	4485872	62755	12407	8307	96.6	1.66
PD	PD14	bin56	MAG2245	130	2518677	109158	37871	19374	96.6	0.51
PD	PD66	bin37	MAG372	110	2307538	127382	43943	20978	96.6	0
PD	PD92	bin45	MAG856	49	2967648	225325	85237	60564	96.6	0.68
PD	PD46	bin34	MAG1727	125	3243467	141089	48050	25948	96.59	0.9
PD	PD56	bin61	MAG377	488	2957774	33577	8220	6061	96.58	2.62
PD	PD94	bin20	MAG1569	85	3462274	222606	68950	40733	96.57	1.92
PD	PD17	bin36	MAG2203	229	2040427	60280	18843	8910	96.54	0.23
PD	PD53	bin11	MAG851	392	1988262	44753	6835	5072	96.51	1.36

PD	PD62	bin52	MAG260	117	1932942	79669	24560	16521	96.51	1.05
PD	PD22	bin51	MAG2432	274	2282243	43973	11368	8329	96.49	0.68
PD	PD23	bin24	MAG1371	25	3918412	457419	252799	156736	96.49	0
PD	PD8	bin18	MAG1321	230	2400967	136878	27636	10439	96.47	2.18
PD	PD78	bin26	MAG2673	434	2800536	58664	14450	6453	96.45	4.2
PD	PD23	bin7	MAG2451	49	2722094	224008	76321	55553	96.43	0
PD	PD37	bin48	MAG2831	257	2782916	56977	17391	10828	96.38	0
PD	PD12	bin12	MAG2784	334	3127374	91979	17512	9363	96.37	4.32
PD	PD86	bin5	MAG2218	306	2989061	64725	14356	9768	96.37	2.37
PD	PD73	bin5	MAG2632	214	2101575	50691	16564	9820	96.36	0.37
PD	PD17	bin27	MAG2328	272	6259848	187118	53424	23014	96.35	1.3
PD	PD97	bin35	MAG3395	145	2452690	72732	30316	16915	96.35	0.68
PD	PD39	bin18	MAG687	59	2552275	230189	84325	43259	96.33	1.38
PD	PD62	bin42	MAG239	301	2360507	47171	11757	7842	96.31	1.34
PD	PD76	bin32	MAG1818	161	2542201	91019	28038	15790	96.31	2.22
PD	PD11	bin19	MAG1057	334	5733486	94342	32074	17166	96.3	0.39
PD	PD47	bin6	MAG2791	107	2685871	129391	32080	25102	96.3	0.12
PD	PD94	bin32	MAG2208	201	4271599	148574	36768	21252	96.3	0
PD	PD31	bin15	MAG2818	180	2613498	123255	21283	14519	96.28	0.79
PD	PD47	bin45	MAG3361	93	3049569	234386	63988	32791	96.28	0.84
PD	PD10	bin8	MAG1616	200	2107804	86803	17134	10539	96.27	2.15
PD	PD14	bin59	MAG2341	208	1895548	57262	15467	9113	96.27	1.34
PD	PD2	bin9	MAG1069	193	1947439	66911	15669	10090	96.27	0
PD	PD13	bin44	MAG3435	186	2582506	139863	33892	13884	96.26	0.53
PD	PD71	bin23	MAG2394	738	3545461	30020	5970	4804	96.26	1.27
PD	PD40	bin54	MAG3199	293	2537258	53903	13022	8660	96.25	4.82
PD	PD78	bin2	MAG1238	211	2538141	59535	16985	12029	96.25	1.51
PD	PD21	bin12	MAG3340	191	1735876	52830	14882	9088	96.24	0.64
PD	PD3	bin8	MAG1638	204	2512076	77901	19809	12314	96.22	0.5
PD	PD40	bin33	MAG2214	145	2197939	61557	22803	15158	96.22	1.33
PD	PD17	bin37	MAG59	237	2039973	100469	17862	8607	96.21	1.8
PD	PD58	bin62	MAG2721	140	2362837	124203	27169	16877	96.2	1.52
PD	PD94	bin12	MAG556	284	2008916	41262	9898	7074	96.2	0

PD	PD70	bin13	MAG3145	356	2935577	42273	11706	8246	96.15	1.19
PD	PD97	bin39	MAG2468	108	2199432	136056	40118	20365	96.15	2.38
PD	PD58	bin12	MAG2102	81	2540528	192301	57011	31365	96.14	0.97
PD	PD100	bin42	MAG204	533	2361996	26663	6274	4432	96.12	2.21
PD	PD11	bin26	MAG2081	48	2360053	177134	77553	49168	96.1	0
PD	PD32	bin34	MAG3155	200	2090482	61148	24948	10452	96.08	0.97
PD	PD82	bin5	MAG1273	39	2310238	266717	107432	59237	96.06	0.91
PD	PD13	bin27	MAG219	415	3492230	61847	14028	8415	96.05	1.42
PD	PD24	bin50	MAG683	251	3026549	126210	20591	12058	96.04	1.97
PD	PD32	bin62	MAG1259	131	3102847	96923	45017	23686	96.02	0.94
PD	PD91	bin46	MAG1445	60	2632222	210561	72216	43870	95.99	0
PD	PD14	bin14	MAG2748	30	1889461	265951	85383	62982	95.97	0
PD	PD32	bin6	MAG2997	18	1867813	399924	161774	103767	95.97	0.67
PD	PD39	bin31	MAG1013	56	2001166	211460	49696	35735	95.97	0
PD	PD48	bin65	MAG546	77	2094537	117508	44991	27202	95.97	0.81
PD	PD57	bin33	MAG1501	46	1896043	344892	216171	41218	95.97	0
PD	PD75	bin31	MAG230	102	2096287	191082	39583	20552	95.97	0.34
PD	PD76	bin58	MAG895	46	2369692	196912	84973	51515	95.97	1.68
PD	PD82	bin32	MAG296	83	1978702	141816	39192	23840	95.97	0
PD	PD83	bin7	MAG2255	22	1755210	253685	129087	79782	95.97	0
PD	PD97	bin29	MAG806	64	1991626	111889	56039	31119	95.97	0.22
PD	PD98	bin35	MAG2974	353	3431738	109632	25482	9722	95.97	3.36
PD	PD34	bin34	MAG201	45	2108206	137226	81150	46849	95.93	0.34
PD	PD23	bin38	MAG2365	99	2261721	286480	51539	22846	95.92	1.36
PD	PD66	bin30	MAG2730	283	2250160	44666	11240	7951	95.92	0.68
PD	PD69	bin38	MAG1885	116	2834906	154682	59968	24439	95.91	2.66
PD	PD31	bin21	MAG317	483	2139580	28106	5629	4430	95.9	1.08
PD	PD44	bin59	MAG2109	111	2488274	108075	37985	22417	95.89	1.1
PD	PD21	bin20	MAG2826	134	3925871	129589	57849	29298	95.86	0
PD	PD78	bin23	MAG3477	105	4741114	398714	123466	45153	95.86	0.75
PD	PD23	bin39	MAG1733	66	4446507	252781	109721	67371	95.83	0.38
PD	PD39	bin40	MAG1494	635	3058844	44117	7249	4817	95.82	1.48
PD	PD39	bin19	MAG80	88	2568379	113378	72003	29186	95.82	1.3

PD	PD2	bin8	MAG2223	139	2153161	90714	29463	15490	95.81	1.42
PD	PD94	bin28	MAG1660	171	2800403	86647	27266	16377	95.8	0.93
PD	PD26	bin17	MAG337	245	2106419	65017	13147	8598	95.79	1.25
PD	PD78	bin33	MAG50	47	3012314	278733	85910	64092	95.78	0.63
PD	PD25	bin82	MAG1426	232	2577887	51442	16322	11112	95.75	1.96
PD	PD98	bin1	MAG3414	123	2202150	93379	27752	17904	95.75	0.17
PD	PD16	bin68	MAG2405	48	1371886	171639	49759	28581	95.74	0
PD	PD12	bin53	MAG1414	233	2131879	54741	13508	9150	95.72	0.99
PD	PD58	bin42	MAG543	390	1988424	34184	6731	5099	95.7	0.4
PD	PD59	bin23	MAG2819	50	1875910	177296	84017	37518	95.7	0
PD	PD41	bin43	MAG3337	315	2871292	49194	12525	9115	95.68	0.94
PD	PD68	bin6	MAG1477	84	3005396	128951	57149	35779	95.65	1.45
PD	PD50	bin23	MAG941	179	2065126	75249	18282	11537	95.64	2.24
PD	PD14	bin38	MAG754	114	2701713	132307	38502	23699	95.61	1.36
PD	PD87	bin26	MAG3089	158	2353931	69513	21083	14898	95.61	2.46
PD	PD91	bin21	MAG2362	58	2930744	313894	69189	50530	95.61	0.84
PD	PD25	bin72	MAG873	124	2471866	85452	32114	19934	95.6	0.34
PD	PD41	bin34	MAG2462	32	2188140	273157	141924	68379	95.6	1.01
PD	PD91	bin7	MAG3261	246	3040102	85644	19245	12358	95.58	5.12
PD	PD100	bin14	MAG659	36	2574419	226286	115506	71512	95.57	0
PD	PD19	bin36	MAG905	64	3000180	192059	119135	46878	95.57	0.95
PD	PD72	bin9	MAG1395	244	2534083	39806	14486	10386	95.56	1.08
PD	PD8	bin45	MAG645	179	1698486	41454	12730	9489	95.56	0.27
PD	PD94	bin6	MAG255	781	3326230	34222	5157	4259	95.56	0.12
PD	PD9	bin19	MAG393	273	2464840	66062	15790	9029	95.52	0.12
PD	PD83	bin36	MAG918	289	2418416	56958	12583	8368	95.47	1.34
PD	PD17	bin32	MAG894	101	2337356	160445	43936	23142	95.45	0.68
PD	PD48	bin77	MAG1187	101	2668754	152376	49327	26423	95.45	1.28
PD	PD53	bin10	MAG3198	317	4002487	117746	26930	12626	95.44	1.84
PD	PD73	bin39	MAG464	134	1858830	57035	21237	13872	95.43	1.4
PD	PD77	bin13	MAG1876	228	2473846	82512	16462	10850	95.43	2.37
PD	PD41	bin27	MAG3228	52	1953532	122917	68665	37568	95.41	1.4
PD	PD67	bin37	MAG94	202	2046937	47897	14292	10133	95.41	0

PD	PD8	bin56	MAG3096	48	3132975	269595	93871	65270	95.38	0.48
PD	PD71	bin54	MAG2111	113	2677925	142046	36072	23698	95.37	0.63
PD	PD77	bin62	MAG1718	212	2484390	91681	20186	11719	95.37	1.36
PD	PD35	bin10	MAG775	505	3648886	61472	11485	7226	95.32	9.89
PD	PD14	bin17	MAG1011	105	2033958	205052	96098	19371	95.3	0.12
PD	PD32	bin32	MAG2172	141	2148434	97086	35634	15237	95.3	0.34
PD	PD48	bin75	MAG848	21	1797671	290635	143691	85603	95.3	0
PD	PD5	bin8	MAG2600	220	2706721	66062	19680	12303	95.3	3.97
PD	PD82	bin30	MAG244	290	2387596	51901	12325	8233	95.3	0.02
PD	PD97	bin25	MAG2703	245	2585768	61664	14707	10554	95.3	3.02
PD	PD98	bin49	MAG826	41	1787132	168791	66187	43589	95.3	0
PD	PD94	bin45	MAG3496	40	2616028	310762	118474	65401	95.27	1.18
PD	PD62	bin50	MAG1604	178	2657982	90446	25924	14932	95.24	0.06
PD	PD72	bin8	MAG1102	121	2346350	133640	44958	19391	95.24	0
PD	PD5	bin43	MAG198	68	2166689	125876	52525	31863	95.21	1.5
PD	PD22	bin10	MAG3206	93	1521109	66455	26724	16356	95.2	0.4
PD	PD18	bin6	MAG541	341	3153389	67986	14489	9247	95.19	2.89
PD	PD3	bin4	MAG823	274	5607730	120772	39556	20466	95.18	3.73
PD	PD66	bin38	MAG3165	156	2002944	51202	18112	12839	95.16	1.19
PD	PD72	bin32	MAG2771	406	2280093	30558	7482	5616	95.16	1.36
PD	PD5	bin49	MAG876	429	2282046	36317	7134	5319	95.13	1.59
PD	PD46	bin70	MAG1760	541	2756468	25604	6257	5095	95.12	0.47
PD	PD57	bin26	MAG912	224	2265558	55370	15093	10114	95.08	1.29
PD	PD17	bin38	MAG828	128	5774652	276577	101935	45114	95.07	4.18
PD	PD53	bin12	MAG272	445	2628779	42953	8654	5907	95.01	2.88
PD	PD64	bin59	MAG1963	54	1832877	133826	57170	33942	95.01	3.91
PD	PD43	bin32	MAG3297	387	2815474	51353	10634	7275	94.99	1.03
PD	PD18	bin4	MAG593	56	1923781	306371	64092	34353	94.97	0.67
PD	PD50	bin37	MAG3465	291	4249718	96695	22769	14604	94.97	1.03
PD	PD13	bin7	MAG56	50	1977858	195328	83478	39557	94.95	0
PD	PD75	bin30	MAG3171	406	2294195	34189	7241	5651	94.95	1.36
PD	PD12	bin25	MAG3053	121	968638	46960	12450	8005	94.94	2.97
PD	PD25	bin32	MAG1803	363	2441225	30557	8922	6725	94.92	2.9

PD	PD58	bin24	MAG2574	184	6961637	217835	61910	37835	94.9	8.93
PD	PD42	bin16	MAG1959	268	1989417	46259	10499	7423	94.87	1.41
PD	PD77	bin66	MAG1570	137	4357506	164764	82916	31807	94.86	2.07
PD	PD72	bin29	MAG1571	352	2431841	55527	12045	6909	94.84	2.46
PD	PD27	bin26	MAG2836	350	2310607	25916	8611	6602	94.83	0.67
PD	PD48	bin48	MAG1855	289	2086459	31091	9866	7220	94.82	2.46
PD	PD71	bin53	MAG2648	92	2009069	177742	47461	21838	94.82	1.23
PD	PD18	bin13	MAG3351	147	2684853	135575	44453	18264	94.79	0.35
PD	PD16	bin30	MAG1086	402	2581362	27182	8751	6421	94.76	1.69
PD	PD58	bin34	MAG2254	61	1821775	150477	41422	29865	94.76	0.79
PD	PD1	bin31	MAG2965	765	6080321	53092	11548	7948	94.75	5.06
PD	PD97	bin21	MAG2207	48	2870771	255374	101513	59808	94.74	1.17
PD	PD41	bin56	MAG1176	76	2170008	132870	55118	28553	94.72	0
PD	PD94	bin7	MAG1560	165	2163888	87275	18831	13114	94.72	0
PD	PD98	bin4	MAG1428	436	2637747	28675	8285	6050	94.71	2.42
PD	PD78	bin37	MAG2967	200	5858221	264061	82999	29291	94.7	0.99
PD	PD59	bin18	MAG3067	124	2477145	120268	33227	19977	94.69	0.26
PD	PD99	bin1	MAG1024	254	2048676	129379	22741	8066	94.66	3.34
PD	PD40	bin50	MAG3030	338	2591105	33682	11141	7666	94.65	1.44
PD	PD72	bin48	MAG467	51	3306830	345191	115604	64840	94.65	0.4
PD	PD13	bin3	MAG1572	300	1558955	26388	6811	5197	94.64	1.61
PD	PD77	bin58	MAG2339	212	1901532	120603	18365	8969	94.64	0.72
PD	PD64	bin79	MAG2273	271	2462093	54665	14310	9085	94.63	3.08
PD	PD67	bin42	MAG992	27	1963998	354498	140784	72741	94.63	1.44
PD	PD82	bin24	MAG3009	409	2105778	23563	6814	5149	94.63	3.8
PD	PD9	bin40	MAG2415	294	2725190	60016	12514	9269	94.63	0.67
PD	PD22	bin29	MAG2621	142	2061298	65487	22027	14516	94.59	0.47
PD	PD34	bin67	MAG1257	332	2224879	28372	8743	6701	94.57	1.27
PD	PD40	bin5	MAG1565	305	2336700	40419	11038	7661	94.56	0.68
PD	PD71	bin15	MAG2904	89	2227381	125372	48666	25027	94.56	0.02
PD	PD35	bin25	MAG3200	283	1795192	37528	8286	6343	94.52	0.71
PD	PD13	bin14	MAG2882	252	2438037	42995	14086	9675	94.51	0.81
PD	PD75	bin50	MAG2228	245	3230375	101737	27356	13185	94.51	1.58

PD	PD9	bin41	MAG471	576	2758935	33860	6466	4790	94.48	2.69
PD	PD27	bin18	MAG3101	352	2078752	43426	8507	5906	94.47	2.73
PD	PD71	bin11	MAG1377	168	2060123	82592	18263	12263	94.47	2.53
PD	PD7	bin20	MAG2861	527	3576717	61808	13755	6787	94.46	1.98
PD	PD71	bin12	MAG331	239	2529267	48232	16184	10583	94.44	2.11
PD	PD10	bin48	MAG1949	100	2709994	107282	46382	27100	94.42	1.27
PD	PD17	bin14	MAG1846	235	2720700	73094	17133	11577	94.41	0.81
PD	PD67	bin40	MAG308	264	2107976	33468	11370	7985	94.39	1.64
PD	PD37	bin10	MAG1236	117	2209246	93555	37331	18882	94.38	0.69
PD	PD64	bin86	MAG2947	365	2341610	33437	8194	6415	94.34	2.36
PD	PD18	bin47	MAG1091	147	1932510	72790	21257	13146	94.33	0
PD	PD40	bin31	MAG769	287	2839134	73387	14421	9892	94.33	0
PD	PD26	bin24	MAG1178	465	2319764	24606	6581	4989	94.31	0.89
PD	PD100	bin30	MAG2137	101	2554787	151725	41144	25295	94.3	0.13
PD	PD28	bin6	MAG2720	243	1992465	48190	11406	8199	94.27	2.62
PD	PD9	bin26	MAG413	185	2403287	78290	19575	12991	94.26	1.78
PD	PD19	bin6	MAG1719	133	6843562	237863	94429	51455	94.25	2.59
PD	PD35	bin45	MAG902	189	2305408	86735	22345	12198	94.25	1.17
PD	PD11	bin20	MAG388	280	2484206	39640	12025	8872	94.22	1.9
PD	PD7	bin19	MAG3146	485	4120796	61569	11922	8496	94.21	1.79
PD	PD36	bin41	MAG279	560	3441674	42681	7917	6146	94.2	2
PD	PD37	bin50	MAG1883	389	2222141	43087	7955	5712	94.16	1.5
PD	PD98	bin11	MAG2501	321	2950311	66675	14530	9191	94.16	2.74
PD	PD4	bin8	MAG2991	189	2394173	99092	32709	12668	94.15	0.03
PD	PD81	bin8	MAG237	107	2419312	110789	40767	22610	94.15	0.35
PD	PD92	bin37	MAG2722	48	2476561	181448	79308	51595	94.15	1.46
PD	PD50	bin62	MAG1315	235	2310139	58872	15262	9830	94.13	2.24
PD	PD13	bin1	MAG2665	575	2234041	32465	5048	3885	94.1	0
PD	PD73	bin35	MAG1535	229	1916761	58671	13217	8370	94.09	2.42
PD	PD61	bin58	MAG737	254	3106598	139045	46645	12231	94.08	3.48
PD	PD83	bin32	MAG1476	400	2908966	38105	9630	7272	94.08	1.03
PD	PD46	bin68	MAG3314	407	2534256	44553	8990	6227	94.07	1.68
PD	PD75	bin4	MAG3504	212	2163369	59769	16859	10205	94.03	2.21

PD	PD31	bin1	MAG2071	138	2963300	110062	37849	21473	93.98	0.68
PD	PD16	bin56	MAG916	470	2751257	35101	7906	5854	93.97	1.36
PD	PD64	bin2	MAG1499	601	5473460	103401	17850	9107	93.97	7.33
PD	PD23	bin9	MAG3180	50	2358438	212927	81607	47169	93.96	1.01
PD	PD29	bin32	MAG1650	222	3216488	78332	29795	14489	93.96	2.35
PD	PD40	bin36	MAG1761	35	2041866	264782	97154	58339	93.96	0.67
PD	PD64	bin9	MAG1046	131	2767353	105157	37088	21125	93.96	1.34
PD	PD81	bin28	MAG2867	85	2444200	146072	46145	28755	93.96	1.01
PD	PD92	bin9	MAG2728	194	2616422	72033	20202	13487	93.96	1.57
PD	PD94	bin39	MAG3056	297	2390787	43407	10685	8050	93.92	1.45
PD	PD58	bin21	MAG772	285	2008011	57303	13738	7046	93.91	5.53
PD	PD1	bin8	MAG2669	339	2177898	50409	10495	6424	93.9	6.97
PD	PD46	bin41	MAG2313	103	2208815	143372	39299	21445	93.88	0
PD	PD73	bin33	MAG120	175	2089780	81864	24213	11942	93.84	3.62
PD	PD2	bin58	MAG864	44	1352078	127407	48211	30729	93.82	3.37
PD	PD26	bin12	MAG3142	443	1658549	22868	4926	3744	93.81	0.55
PD	PD16	bin10	MAG1404	311	2740764	50570	12312	8813	93.79	1.84
PD	PD50	bin1	MAG1800	184	2518296	79301	22566	13686	93.77	0
PD	PD28	bin47	MAG265	277	2289861	44845	12058	8267	93.76	0.74
PD	PD91	bin4	MAG72	151	3892513	240443	79346	25778	93.75	0.87
PD	PD92	bin5	MAG2980	144	1983344	87189	31440	13773	93.74	3.24
PD	PD82	bin36	MAG470	238	2505957	75545	16131	10529	93.71	1.12
PD	PD7	bin40	MAG3102	61	1920660	199292	65952	31486	93.68	1.75
PD	PD70	bin2	MAG2750	44	1873682	181996	67054	42584	93.68	1.4
PD	PD61	bin44	MAG3390	70	2582504	142027	69965	36893	93.67	1.68
PD	PD78	bin34	MAG3327	178	2540025	70756	20052	14270	93.67	0.02
PD	PD25	bin10	MAG842	239	1975525	48833	11330	8266	93.65	1.08
PD	PD9	bin13	MAG2768	186	2072203	86648	18249	11141	93.65	0.67
PD	PD19	bin7	MAG2912	135	2223744	114809	34499	16472	93.62	0.67
PD	PD41	bin9	MAG79	446	1856418	24593	5366	4162	93.62	2.07
PD	PD77	bin29	MAG1632	399	2726485	29680	10025	6833	93.62	3.83
PD	PD20	bin7	MAG3303	298	1807592	35857	8190	6066	93.61	1.86
PD	PD5	bin24	MAG3333	120	2766529	116629	37818	23054	93.6	1.55

PD	PD12	bin28	MAG742	271	2641490	49383	12938	9747	93.57	2.95
PD	PD2	bin42	MAG2689	188	1735297	67901	13469	9230	93.57	0.67
PD	PD57	bin87	MAG2849	190	2606907	54933	20934	13721	93.57	0.95
PD	PD97	bin51	MAG3474	238	2602264	84030	15576	10934	93.57	1.41
PD	PD3	bin52	MAG1706	83	1682020	68501	38113	20265	93.55	0
PD	PD43	bin55	MAG2664	186	2187010	82732	17134	11758	93.55	0.95
PD	PD59	bin13	MAG2846	150	1948143	52226	21528	12988	93.55	2.02
PD	PD75	bin11	MAG498	63	1707744	169459	34830	27107	93.55	0
PD	PD53	bin27	MAG1495	131	2119716	87334	28227	16181	93.53	4.62
PD	PD42	bin8	MAG1360	551	4370520	42384	10977	7932	93.51	1.29
PD	PD43	bin2	MAG3323	386	2728675	39633	9321	7069	93.46	0.01
PD	PD45	bin29	MAG2555	338	2462185	41415	9363	7285	93.42	2.95
PD	PD35	bin4	MAG3278	157	2269925	58740	24903	14458	93.4	0.06
PD	PD44	bin31	MAG3242	682	2291670	22113	4056	3360	93.4	1.76
PD	PD34	bin17	MAG2360	156	1950203	64610	18537	12501	93.39	1.22
PD	PD45	bin30	MAG658	288	2980461	67854	15222	10349	93.34	0.92
PD	PD13	bin58	MAG3229	68	1272899	81680	35277	18719	93.33	1.33
PD	PD87	bin6	MAG1137	144	2304669	66891	21774	16005	93.3	2.01
PD	PD12	bin74	MAG3421	111	1879595	91974	25291	16933	93.29	1.34
PD	PD18	bin34	MAG3478	85	2483118	196700	44029	29213	93.29	8.72
PD	PD24	bin24	MAG1349	54	2121307	275508	77597	39283	93.29	0.34
PD	PD29	bin83	MAG2929	91	2688768	108507	54728	29547	93.29	1.34
PD	PD50	bin28	MAG3493	396	2958173	45989	10534	7470	93.29	1.38
PD	PD59	bin44	MAG526	66	2938305	224942	77996	44520	93.29	0.22
PD	PD7	bin6	MAG2053	275	3100918	74972	17736	11276	93.29	2.65
PD	PD94	bin70	MAG1314	164	3177202	142342	54914	19373	93.29	2.35
PD	PD34	bin36	MAG2033	334	1736591	22792	7285	5199	93.27	2.18
PD	PD10	bin19	MAG554	238	2112371	45016	14284	8876	93.26	0.34
PD	PD64	bin91	MAG2737	11	1289399	544244	390693	117218	93.26	1.28
PD	PD62	bin56	MAG3078	241	2638975	114004	17217	10950	93.23	3.84
PD	PD66	bin13	MAG2526	195	1795904	37667	13347	9210	93.22	1.79
PD	PD35	bin22	MAG1381	455	2196472	45096	7535	4827	93.2	2.4
PD	PD56	bin45	MAG982	118	2516952	146048	42274	21330	93.2	0

PD	PD62	bin23	MAG1185	428	2213461	28860	6573	5172	93.2	0.74
PD	PD73	bin67	MAG14	68	2033625	235646	73658	29906	93.2	0.81
PD	PD98	bin50	MAG3230	74	2097947	221849	61227	28351	93.2	0
PD	PD68	bin4	MAG3457	78	2376915	137719	50743	30473	93.19	0.74
PD	PD44	bin33	MAG879	369	2512738	67101	9716	6810	93.14	8.13
PD	PD58	bin26	MAG562	334	2169426	36673	8388	6495	93.12	1.93
PD	PD43	bin41	MAG2297	581	2082566	39432	4482	3584	93.11	4.08
PD	PD9	bin62	MAG2380	484	2680200	33226	7398	5538	93.11	1.93
PD	PD29	bin19	MAG270	221	2840365	73476	17926	12852	93.09	1.08
PD	PD62	bin7	MAG478	315	2885199	73399	13472	9159	93.09	1.45
PD	PD61	bin1	MAG1346	316	2375642	69111	11067	7518	93.08	5.41
PD	PD53	bin14	MAG3256	183	1995958	136896	25988	10907	93.06	4.42
PD	PD19	bin9	MAG1492	82	2678169	144604	45733	32661	93.04	0.16
PD	PD21	bin4	MAG2853	157	3345742	89373	29179	21310	93.04	0
PD	PD97	bin3	MAG1132	256	2806130	47910	16205	10961	93.04	0.29
PD	PD68	bin32	MAG1600	239	3094885	162963	42577	12949	93.03	3.14
PD	PD73	bin47	MAG824	278	1735772	25535	7803	6244	93.03	3.25
PD	PD68	bin28	MAG2014	409	1894493	36557	6859	4632	93.01	3.45
PD	PD78	bin20	MAG96	77	3087030	169610	64009	40091	93.01	0.13
PD	PD43	bin52	MAG971	509	2169849	20605	5343	4263	92.98	0.53
PD	PD68	bin30	MAG2767	413	2408979	29562	7481	5833	92.95	0
PD	PD46	bin45	MAG2288	429	1951371	28503	5664	4549	92.93	4.47
PD	PD85	bin11	MAG607	226	2590416	58595	16773	11462	92.92	0
PD	PD39	bin7	MAG1337	34	2085270	256089	91495	61331	92.91	0
PD	PD36	bin5	MAG2646	162	2442802	84240	22611	15079	92.87	1.81
PD	PD87	bin24	MAG277	199	2716156	163733	44996	13649	92.81	4.65
PD	PD68	bin15	MAG3268	236	2118236	61041	14409	8976	92.8	4.09
PD	PD97	bin15	MAG1189	249	1722013	32905	9364	6916	92.79	0.71
PD	PD20	bin30	MAG2563	275	4080316	109930	36207	14838	92.75	1.05
PD	PD66	bin3	MAG2541	44	2557454	219931	68579	58124	92.74	0
PD	PD9	bin63	MAG492	363	2061921	67025	8000	5680	92.74	2.94
PD	PD1	bin29	MAG3313	300	2141730	102867	10696	7139	92.71	2.87
PD	PD10	bin21	MAG732	95	1339752	54524	20685	14103	92.7	0.56

PD	PD98	bin58	MAG1397	181	2180787	81977	18316	12049	92.7	0.81
PD	PD35	bin28	MAG3084	533	2486518	28389	6327	4665	92.69	2.35
PD	PD50	bin53	MAG1877	492	1846348	20796	4973	3753	92.69	3.49
PD	PD59	bin38	MAG1179	367	4131948	61483	17521	11259	92.69	1.39
PD	PD76	bin43	MAG2617	202	2744497	115026	26640	13587	92.64	1.92
PD	PD24	bin43	MAG1509	262	2723549	41118	15112	10395	92.63	0.81
PD	PD57	bin81	MAG793	153	3073090	144322	36563	20086	92.62	0.67
PD	PD97	bin53	MAG2506	51	2015147	262640	79267	39513	92.62	0
PD	PD29	bin48	MAG3363	380	2349322	42956	8397	6182	92.61	3.32
PD	PD7	bin38	MAG2500	286	6241442	102552	37336	21823	92.6	1.86
PD	PD18	bin17	MAG638	98	4351905	213396	87576	44407	92.58	0.93
PD	PD57	bin25	MAG1787	51	1885921	151535	70078	36979	92.58	1.34
PD	PD61	bin19	MAG253	448	1971332	40802	5574	4400	92.57	2.59
PD	PD91	bin6	MAG2885	72	2874749	281875	52844	39927	92.56	1.12
PD	PD12	bin21	MAG1951	227	2010182	53828	12679	8855	92.52	3.33
PD	PD56	bin25	MAG3467	81	2266707	117957	40777	27984	92.52	0.85
PD	PD29	bin4	MAG1986	404	2553502	51472	8862	6321	92.51	4.08
PD	PD95	bin32	MAG3013	113	2628254	174784	39989	23259	92.5	0.73
PD	PD43	bin1	MAG722	636	3830924	36178	7392	6023	92.47	1.48
PD	PD57	bin2	MAG1774	223	2816442	53154	16923	12630	92.45	5.17
PD	PD72	bin30	MAG1282	444	2647493	26088	7797	5963	92.45	2.56
PD	PD22	bin41	MAG2343	45	1294964	114151	57893	28777	92.42	1.12
PD	PD25	bin83	MAG2270	51	1316931	168353	53318	25822	92.42	2.25
PD	PD34	bin66	MAG1984	50	1202657	117182	48953	24053	92.42	1.12
PD	PD5	bin33	MAG151	69	1234266	70402	30391	17888	92.42	1.12
PD	PD58	bin59	MAG2607	75	1323969	64921	28071	17653	92.42	2.25
PD	PD70	bin23	MAG1859	77	1293226	80301	26321	16795	92.42	1.12
PD	PD87	bin37	MAG3208	31	1293244	178879	116820	41718	92.42	1.12
PD	PD94	bin60	MAG1759	56	1226509	129994	45043	21902	92.42	0
PD	PD98	bin37	MAG1709	49	1316107	96323	63434	26859	92.42	1.28
PD	PD32	bin35	MAG2618	330	2822711	57267	13525	8554	92.41	3.72
PD	PD18	bin5	MAG2841	152	2627731	302284	181246	17288	92.4	0.58
PD	PD13	bin41	MAG3459	910	3933641	22061	6025	4323	92.38	4.65

PD	PD17	bin41	MAG2881	297	2061038	36034	9099	6940	92.38	3.63
PD	PD26	bin6	MAG1	297	2091102	42112	13222	7041	92.38	1.5
PD	PD25	bin75	MAG859	99	2027999	143070	35880	20485	92.37	1.29
PD	PD28	bin18	MAG576	77	1962157	98373	39370	25483	92.35	0
PD	PD76	bin14	MAG1474	204	2260468	57070	16425	11081	92.34	0.81
PD	PD60	bin15	MAG3320	446	2206318	27348	6252	4947	92.33	1.01
PD	PD64	bin58	MAG235	51	2926856	239061	82799	57389	92.31	0.96
PD	PD77	bin16	MAG1699	189	2363694	63297	19612	12506	92.28	3.36
PD	PD8	bin34	MAG2636	54	1940308	159400	71587	35932	92.28	2.01
PD	PD72	bin16	MAG782	36	1941944	156717	77444	53943	92.26	1.34
PD	PD85	bin17	MAG1111	24	1901122	253622	104002	79213	92.22	0.55
PD	PD3	bin45	MAG773	83	2416138	191807	52641	29110	92.21	0.89
PD	PD94	bin72	MAG2307	263	2204082	41615	12148	8381	92.21	1.7
PD	PD99	bin45	MAG1580	462	2149410	43513	8223	4652	92.21	3.4
PD	PD68	bin70	MAG1425	429	2061591	27304	5930	4806	92.18	1.53
PD	PD100	bin43	MAG2998	134	3370042	156858	61560	25150	92.17	0.96
PD	PD50	bin60	MAG3331	47	1713176	207669	66434	36451	92.17	1.01
PD	PD32	bin41	MAG3175	250	3544568	119497	44473	14178	92.16	1.94
PD	PD46	bin7	MAG3397	188	1915129	56449	14460	10187	92.15	2.45
PD	PD72	bin3	MAG483	147	2262274	68691	24514	15390	92.15	0.68
PD	PD29	bin8	MAG512	406	2565178	44577	8243	6318	92.11	0
PD	PD59	bin10	MAG3375	82	3550598	168257	64697	43300	92.09	0
PD	PD7	bin30	MAG2281	120	3379190	237722	89018	28160	92.07	0.83
PD	PD25	bin87	MAG2930	245	1883311	63619	15774	7687	92.04	2.13
PD	PD91	bin5	MAG2937	613	3889731	37841	8060	6345	92.01	1.08
PD	PD58	bin63	MAG1291	80	2745701	227040	53142	34321	91.98	0.63
PD	PD73	bin64	MAG3122	119	2287126	74178	29228	19220	91.98	0.01
PD	PD12	bin33	MAG2008	305	2745853	46329	11387	9003	91.95	1.71
PD	PD25	bin44	MAG2038	80	1758216	114252	38521	21978	91.95	0
PD	PD41	bin35	MAG3107	108	1948247	112366	32099	18039	91.95	1.01
PD	PD69	bin49	MAG3039	241	3364976	122308	29795	13963	91.95	1.34
PD	PD69	bin36	MAG892	51	2479129	278029	80275	48610	91.94	0
PD	PD83	bin22	MAG1627	228	2571823	44753	16453	11280	91.94	1.36

PD	PD8	bin2	MAG3094	277	2162956	52619	10152	7809	91.93	0.34
PD	PD57	bin13	MAG2633	102	2363250	118188	42060	23169	91.92	3.14
PD	PD3	bin6	MAG3492	330	2634390	42241	11426	7983	91.9	1.55
PD	PD5	bin41	MAG479	120	3257258	151540	52443	27144	91.89	1.35
PD	PD73	bin72	MAG2757	107	2040346	180918	53966	19069	91.86	3.11
PD	PD100	bin41	MAG1882	104	2550259	156116	42979	24522	91.85	0.09
PD	PD85	bin30	MAG1099	39	1896311	147367	78092	48623	91.84	0
PD	PD82	bin23	MAG2042	49	2124677	270546	106174	43361	91.8	2.88
PD	PD75	bin48	MAG3427	241	5815560	252466	63185	24131	91.79	1.21
PD	PD36	bin8	MAG488	59	2362547	178296	76840	40043	91.78	0
PD	PD24	bin47	MAG3350	479	1934848	46181	5308	4039	91.76	6.45
PD	PD68	bin59	MAG2672	240	2203776	72693	16529	9182	91.74	0.68
PD	PD3	bin40	MAG3413	283	1921821	34796	8958	6791	91.73	0.14
PD	PD45	bin1	MAG1522	255	1979669	36240	11338	7763	91.73	1.81
PD	PD39	bin41	MAG2485	138	2087999	71742	25160	15130	91.7	0.17
PD	PD24	bin56	MAG1353	534	2240869	21904	4964	4196	91.68	1.7
PD	PD41	bin26	MAG87	540	2661077	52480	5881	4928	91.65	1.34
PD	PD48	bin67	MAG1798	305	2794232	59006	13780	9161	91.65	1.69
PD	PD47	bin32	MAG1966	52	2414066	199561	72825	46424	91.61	0.67
PD	PD50	bin9	MAG74	291	3655173	84222	18075	12561	91.61	0.89
PD	PD58	bin83	MAG71	49	1777327	146993	59160	36272	91.61	0.67
PD	PD87	bin47	MAG472	571	3945850	62455	10634	6910	91.61	1.9
PD	PD98	bin26	MAG1517	71	1868557	153135	34169	26318	91.61	0.67
PD	PD98	bin25	MAG3399	472	2616861	20407	7166	5544	91.6	1.74
PD	PD2	bin57	MAG2381	528	2553903	43109	5884	4837	91.59	3.09
PD	PD44	bin47	MAG1591	157	2640655	89485	29278	16819	91.58	3.06
PD	PD78	bin27	MAG1762	161	4794186	344352	119827	29778	91.58	1.05
PD	PD10	bin38	MAG3074	63	1247866	79726	45289	19807	91.57	5.06
PD	PD22	bin36	MAG242	188	3167637	102195	26877	16849	91.55	1.63
PD	PD86	bin28	MAG2893	453	1609356	16341	4771	3553	91.55	1.46
PD	PD18	bin33	MAG1967	180	1978260	45819	16084	10990	91.52	0.89
PD	PD87	bin63	MAG395	297	2584249	61673	14578	8701	91.51	2.52
PD	PD64	bin3	MAG1955	202	1658416	41916	11240	8210	91.49	1.42

PD	PD81	bin44	MAG2550	346	2812916	78639	11362	8130	91.47	2.58
PD	PD99	bin4	MAG565	1173	4088264	19024	4568	3485	91.45	1.93
PD	PD66	bin19	MAG854	489	2187249	33761	5791	4473	91.43	0.44
PD	PD61	bin47	MAG1105	536	3307076	83405	13224	6170	91.42	5.33
PD	PD70	bin58	MAG176	144	2029282	87213	23216	14092	91.36	0.22
PD	PD99	bin15	MAG156	148	2338016	71748	24104	15797	91.33	0.18
PD	PD57	bin72	MAG1380	107	2112986	99054	30254	19748	91.32	1.68
PD	PD94	bin21	MAG3244	392	2429850	50310	8330	6199	91.31	1.7
PD	PD17	bin2	MAG2173	223	4729138	148538	37644	21207	91.29	5.74
PD	PD67	bin14	MAG2521	176	1093210	44415	8693	6211	91.29	0
PD	PD10	bin46	MAG1784	543	2974033	26565	6936	5477	91.28	1.53
PD	PD12	bin9	MAG3374	49	1911309	189486	87011	39006	91.28	2.01
PD	PD22	bin3	MAG2825	71	2034339	142561	46364	28653	91.28	0.34
PD	PD46	bin44	MAG1515	111	2046139	76315	28509	18434	91.28	0
PD	PD48	bin23	MAG1401	44	1893596	165313	82012	43036	91.28	0.67
PD	PD41	bin50	MAG3263	317	3932719	89518	17792	12406	91.27	1.8
PD	PD48	bin66	MAG3212	216	1843995	53808	14110	8537	91.23	2.99
PD	PD95	bin1	MAG3371	61	2115608	155894	53451	34682	91.23	0.58
PD	PD95	bin5	MAG685	905	5256295	42455	9631	5808	91.22	1.76
PD	PD37	bin21	MAG2134	600	2917652	38734	6356	4863	91.16	0.74
PD	PD83	bin16	MAG1036	656	2765785	22529	5444	4216	91.14	2.63
PD	PD41	bin42	MAG1626	48	2096526	178908	100948	43678	91.05	0
PD	PD97	bin8	MAG1578	345	2103193	37189	10245	6096	91.02	4.22
PD	PD13	bin19	MAG2656	64	1172758	98487	43406	18324	91.01	5.62
PD	PD34	bin71	MAG391	313	2480332	60578	12599	7924	91.01	2.04
PD	PD9	bin24	MAG162	16	1116209	256300	143994	69763	91.01	1.28
PD	PD46	bin16	MAG1429	331	1875616	31987	8979	5667	91	3.99
PD	PD91	bin1	MAG3471	33	1975574	251290	98065	59866	91	1.5
PD	PD50	bin3	MAG963	219	5573252	158470	38190	25449	90.97	0.38
PD	PD34	bin25	MAG2679	492	1825125	15629	4836	3710	90.95	6.27
PD	PD25	bin74	MAG1824	54	1856189	125865	63635	34374	90.94	0.69
PD	PD47	bin40	MAG180	301	2279342	36955	10633	7573	90.94	2.04
PD	PD92	bin7	MAG3042	458	2335352	30489	6144	5099	90.93	1.41

PD	PD20	bin16	MAG548	337	3546784	58866	13513	10525	90.84	2.15
PD	PD57	bin23	MAG2870	321	2483241	56640	14941	7736	90.82	3.75
PD	PD75	bin5	MAG2491	359	2441709	94972	10005	6801	90.82	1.25
PD	PD18	bin57	MAG2108	469	2002009	25307	5158	4269	90.79	2.91
PD	PD82	bin43	MAG3224	143	2756851	225077	48406	19279	90.72	1.58
PD	PD16	bin53	MAG845	253	2075323	68816	14161	8203	90.68	0
PD	PD12	bin34	MAG368	286	1865127	34548	8751	6521	90.65	0.27
PD	PD25	bin84	MAG1900	50	1873908	197082	52384	37478	90.6	0.69
PD	PD60	bin25	MAG994	112	2035505	88135	30124	18174	90.6	0
PD	PD68	bin42	MAG491	57	2045185	210960	54067	35880	90.6	0.67
PD	PD7	bin12	MAG2792	71	2089071	253344	56751	29424	90.6	0.69
PD	PD85	bin19	MAG827	231	2257934	48243	13163	9775	90.6	0.91
PD	PD32	bin83	MAG3236	256	1673222	33466	9375	6536	90.59	3.25
PD	PD9	bin2	MAG2985	121	3268187	142821	48124	27010	90.57	0.31
PD	PD10	bin35	MAG30	661	2375865	20546	4655	3594	90.56	1.56
PD	PD17	bin40	MAG2961	336	1819837	42623	6779	5416	90.52	0
PD	PD46	bin51	MAG3201	233	1980434	77541	15060	8500	90.49	1.64
PD	PD2	bin21	MAG2595	51	1258727	122277	39388	24681	90.45	2.25
PD	PD72	bin55	MAG1524	541	2119612	18019	5121	3918	90.45	3.42
PD	PD43	bin15	MAG2252	298	3357052	82869	19972	11265	90.43	3.05
PD	PD87	bin61	MAG1373	235	2823959	81433	17259	12017	90.42	2.19
PD	PD69	bin11	MAG807	51	2365997	227260	114776	46392	90.41	0
PD	PD41	bin48	MAG1285	486	1569099	31281	3983	3229	90.39	3.09
PD	PD29	bin11	MAG414	93	2985983	175795	46233	32107	90.35	0.72
PD	PD36	bin44	MAG3319	482	2876853	31805	8090	5969	90.35	1.27
PD	PD25	bin63	MAG3346	156	2824289	102544	28238	18104	90.33	0.23
PD	PD97	bin13	MAG60	397	2259489	60773	7281	5691	90.32	4.34
PD	PD69	bin32	MAG2876	352	1898376	33283	6479	5393	90.29	1.04
PD	PD37	bin4	MAG86	920	4191760	66108	6389	4556	90.28	6.75
PD	PD44	bin22	MAG1405	38	1892885	194656	77990	49813	90.27	0
PD	PD57	bin14	MAG192	72	1956315	178386	37807	27171	90.27	1.34
PD	PD64	bin55	MAG1294	125	1943805	90433	29660	15550	90.27	0.67
PD	PD50	bin38	MAG820	773	2783679	20795	4549	3601	90.26	0.68

PD	PD94	bin15	MAG3133	590	1949249	18949	3878	3304	90.21	5
PD	PD57	bin49	MAG2322	211	1155184	41308	7621	5475	90.17	3.37
PD	PD3	bin24	MAG1053	356	1682752	30680	6085	4727	90.15	1.23
PD	PD97	bin19	MAG1119	433	2166066	25556	6431	5002	90.14	0.67
PD	PD18	bin55	MAG3510	520	2706237	25598	6531	5204	90.13	1.27
PD	PD95	bin35	MAG744	739	2300248	31860	3843	3113	90.06	5.56
PD	PD72	bin24	MAG2246	90	2499185	155887	45022	27769	90.05	0
PD	PD47	bin52	MAG95	377	2285047	33416	8116	6061	90.04	1.4
PD	PD18	bin22	MAG2179	498	2576422	36066	6740	5174	90.03	3.08
PD	PD2	bin33	MAG903	284	1781892	25992	7909	6274	90	3.43
PD	PD58	bin9	MAG2101	590	1576213	17951	3165	2672	89.99	5.16
PD	PD27	bin38	MAG1661	342	1866100	33326	6735	5456	89.95	0
PD	PD12	bin52	MAG381	62	1715099	210753	80622	27663	89.93	0
PD	PD16	bin29	MAG623	106	2184830	148653	37007	20612	89.93	0
PD	PD68	bin29	MAG3329	106	2414766	197476	35394	22781	89.93	1.34
PD	PD97	bin28	MAG2797	48	1880797	158581	79095	39183	89.93	0.67
PD	PD17	bin17	MAG3082	416	2129242	29515	7075	5118	89.88	3.05
PD	PD34	bin38	MAG974	301	2282844	30549	10524	7584	89.84	0.94
PD	PD18	bin53	MAG1615	52	2246405	282772	88180	43200	89.8	0
PD	PD62	bin1	MAG375	848	4317692	34911	7872	5092	89.79	2.91
PD	PD56	bin57	MAG2833	594	2477095	21025	5215	4170	89.78	0.86
PD	PD40	bin10	MAG644	55	2181556	179622	90578	39665	89.75	0.69
PD	PD98	bin51	MAG380	333	2227057	49359	12558	6688	89.75	3.64
PD	PD47	bin31	MAG763	111	1794885	79283	24964	16170	89.74	0.93
PD	PD44	bin36	MAG923	509	2744413	61864	10271	5392	89.73	0.65
PD	PD56	bin62	MAG2654	235	1768757	28601	11523	7527	89.72	1.23
PD	PD71	bin58	MAG2627	297	2947695	85030	13641	9925	89.72	1.14
PD	PD13	bin18	MAG2924	515	2078031	25139	5112	4035	89.71	1.85
PD	PD18	bin2	MAG538	135	1894316	88480	35821	14032	89.69	1.55
PD	PD10	bin52	MAG1459	680	2570742	32215	5205	3781	89.67	7.23
PD	PD71	bin56	MAG757	495	2506904	33259	6125	5064	89.66	0.84
PD	PD61	bin43	MAG661	83	2393791	167765	46833	28841	89.63	0
PD	PD13	bin56	MAG153	828	1942816	12335	2671	2346	89.62	3.9

PD	PD6	bin7	MAG2248	164	2272545	70580	20584	13857	89.6	2.1
PD	PD71	bin30	MAG1589	210	2205010	72556	14715	10500	89.57	3.96
PD	PD72	bin7	MAG1324	240	2911553	80227	19897	12131	89.56	2.95
PD	PD46	bin30	MAG3380	115	3015901	127063	48109	26225	89.52	0.48
PD	PD14	bin5	MAG1131	170	1988083	54035	16873	11695	89.5	2.1
PD	PD77	bin11	MAG35	554	1754065	12714	3795	3166	89.48	0.62
PD	PD77	bin28	MAG453	365	2442405	49752	8583	6692	89.48	2.58
PD	PD85	bin38	MAG287	534	1808294	36738	4309	3386	89.48	3.56
PD	PD36	bin45	MAG3010	55	2448410	190252	74840	44517	89.46	0.68
PD	PD82	bin34	MAG2052	224	2148814	39083	14172	9593	89.46	2.03
PD	PD32	bin52	MAG1390	149	2352674	63937	24267	15790	89.44	0
PD	PD5	bin12	MAG2268	53	1751390	120301	53490	33045	89.43	0
PD	PD8	bin47	MAG3117	308	2303505	48138	10618	7479	89.42	3.93
PD	PD43	bin46	MAG1210	1251	3778944	20799	3575	3021	89.4	6.54
PD	PD19	bin10	MAG3276	344	1257803	18863	4090	3656	89.39	1.33
PD	PD32	bin2	MAG587	131	1187498	43565	13338	9065	89.33	2.81
PD	PD47	bin46	MAG1785	407	1938041	23811	5779	4762	89.27	2.96
PD	PD78	bin14	MAG525	382	2545134	39617	9099	6663	89.27	2.31
PD	PD69	bin42	MAG2200	32	1911121	285724	114099	59723	89.26	0
PD	PD46	bin15	MAG1184	54	1967551	176640	69254	36436	89.22	2.35
PD	PD2	bin50	MAG1692	471	1962114	18627	5189	4166	89.21	2.68
PD	PD25	bin3	MAG712	357	1521050	20131	5035	4261	89.21	3.52
PD	PD48	bin9	MAG1258	739	2924873	31615	4844	3958	89.21	2.16
PD	PD16	bin36	MAG3114	175	2146460	97433	20638	12265	89.17	0.68
PD	PD61	bin39	MAG2295	431	1783481	19148	4999	4138	89.14	1.72
PD	PD13	bin31	MAG600	426	1793622	27594	5360	4210	89.1	1.61
PD	PD41	bin11	MAG2096	322	1802628	23420	7201	5598	89.01	5.37
PD	PD18	bin27	MAG1636	482	2302652	26393	5705	4777	88.94	2.01
PD	PD12	bin20	MAG2144	295	1945236	35395	8197	6594	88.9	3.56
PD	PD73	bin78	MAG3444	918	2762055	14294	3690	3009	88.85	2.7
PD	PD69	bin53	MAG3123	600	3105521	37083	6726	5176	88.76	1.91
PD	PD64	bin23	MAG662	38	1799924	143045	90031	47366	88.7	0
PD	PD31	bin37	MAG680	287	2222699	35582	11227	7745	88.69	1.45

PD	PD29	bin12	MAG1576	328	2297933	48107	9195	7006	88.65	0.35
PD	PD45	bin32	MAG407	361	1701994	38947	6648	4715	88.64	1.31
PD	PD2	bin16	MAG1645	284	2091801	49669	10517	7365	88.6	0.62
PD	PD60	bin12	MAG2644	37	1836543	137049	82988	49636	88.6	1.5
PD	PD36	bin14	MAG3016	140	2104165	117217	30213	15030	88.59	0.34
PD	PD5	bin9	MAG366	132	2053840	166990	33335	15559	88.59	0.34
PD	PD59	bin53	MAG1542	91	2173820	159415	43285	23888	88.59	0
PD	PD59	bin42	MAG2951	102	1816292	65330	29082	17807	88.59	0.69
PD	PD87	bin53	MAG1795	65	1882061	148269	58622	28955	88.59	0.67
PD	PD92	bin12	MAG99	98	2040597	105011	31279	20822	88.59	0
PD	PD29	bin54	MAG415	710	2066494	14976	3367	2911	88.58	1.28
PD	PD50	bin24	MAG1303	195	1799663	86951	14692	9229	88.58	2.57
PD	PD72	bin49	MAG3377	31	1962242	249818	103030	63298	88.55	0
PD	PD77	bin27	MAG3400	621	1922371	13887	3624	3096	88.55	4.07
PD	PD60	bin9	MAG352	532	1626490	13398	3657	3057	88.54	1.85
PD	PD83	bin4	MAG2957	974	2735600	15252	3352	2809	88.41	4.53
PD	PD14	bin29	MAG3342	828	4163262	28086	5867	5028	88.4	2.5
PD	PD73	bin57	MAG1674	489	1786943	21836	4504	3654	88.4	5.93
PD	PD69	bin31	MAG392	207	1952374	56029	16283	9432	88.36	1.01
PD	PD43	bin29	MAG342	744	3660766	25199	6378	4920	88.35	2.5
PD	PD35	bin46	MAG2564	255	2125019	94276	16564	8333	88.3	0.7
PD	PD50	bin26	MAG1656	77	1683512	96993	36712	21864	88.3	0.67
PD	PD14	bin2	MAG3092	77	1732661	207629	46514	22502	88.26	0.34
PD	PD70	bin28	MAG1482	16	1594422	231957	167332	99651	88.26	0
PD	PD72	bin12	MAG3062	128	2168494	95026	25678	16941	88.26	1.23
PD	PD28	bin23	MAG1455	141	2470007	84182	25723	17518	88.25	1.09
PD	PD66	bin31	MAG2613	545	3251165	49981	7486	5965	88.23	3.04
PD	PD9	bin11	MAG1577	652	1989306	15387	3666	3051	88.2	3.95
PD	PD92	bin13	MAG2880	766	2494055	16934	4074	3256	88.19	1.46
PD	PD8	bin14	MAG2765	806	2862640	18480	4267	3552	88.18	9.57
PD	PD9	bin54	MAG2219	486	2443164	49198	8328	5027	88.17	5.75
PD	PD16	bin31	MAG1669	704	3056607	38977	5499	4342	88.15	4.53
PD	PD8	bin15	MAG1595	479	2126462	28147	5546	4439	88.15	2.43

PD	PD31	bin19	MAG1747	471	2754299	49797	7573	5848	88.06	4.95
PD	PD61	bin22	MAG1612	694	1958268	11405	3196	2822	88.05	3.55
PD	PD16	bin54	MAG3190	146	1850282	90580	21834	12673	88.04	1.17
PD	PD27	bin25	MAG566	578	2909329	41022	6373	5033	88.03	4.18
PD	PD45	bin61	MAG2277	566	2193685	32543	4895	3876	87.97	5.93
PD	PD28	bin4	MAG1113	476	2276339	23827	5964	4782	87.96	1.34
PD	PD31	bin28	MAG1610	54	1762768	187772	53241	32644	87.92	0.02
PD	PD8	bin38	MAG1753	482	1722511	25497	4338	3574	87.86	0.12
PD	PD62	bin11	MAG3120	428	2075082	47588	7899	4848	87.79	2.24
PD	PD73	bin68	MAG2425	522	1721890	14342	3753	3299	87.78	8.41
PD	PD5	bin5	MAG2877	435	1876616	19481	5239	4314	87.75	1.88
PD	PD10	bin15	MAG2116	616	2068299	23503	4269	3358	87.74	1.25
PD	PD66	bin39	MAG2150	495	1976336	23294	5334	3993	87.74	1.93
PD	PD13	bin48	MAG1682	144	1801352	97044	24159	12509	87.72	2.1
PD	PD10	bin31	MAG739	502	1557390	14995	3851	3102	87.64	0.75
PD	PD72	bin18	MAG1235	577	3951091	56672	9962	6848	87.64	4.49
PD	PD22	bin31	MAG1691	88	1986791	69057	36573	22577	87.58	0.67
PD	PD41	bin74	MAG1497	699	1963551	12473	3400	2809	87.58	2.97
PD	PD92	bin55	MAG1884	83	2424221	132495	50117	29207	87.5	0.73
PD	PD45	bin38	MAG1826	380	1765236	32452	5729	4645	87.47	0.34
PD	PD94	bin52	MAG2762	590	2010150	23929	4060	3407	87.46	3.81
PD	PD24	bin13	MAG149	271	2232262	51081	12595	8237	87.42	2.62
PD	PD43	bin43	MAG1375	1301	2974425	10705	2497	2286	87.42	2.14
PD	PD35	bin14	MAG919	21	1825135	643133	123432	86911	87.41	1.5
PD	PD73	bin56	MAG715	333	1914407	42509	7689	5749	87.38	2.5
PD	PD5	bin6	MAG3052	228	1157673	21626	7186	5078	87.36	6.18
PD	PD3	bin10	MAG2847	321	1856949	49465	8957	5785	87.34	4.73
PD	PD58	bin25	MAG1671	64	1746989	194243	44932	27297	87.31	0.73
PD	PD29	bin55	MAG249	143	2068936	87064	24490	14468	87.25	0
PD	PD5	bin46	MAG42	71	1826282	121970	67142	25722	87.25	0
PD	PD81	bin25	MAG2338	637	2811338	30132	8097	4413	87.23	4.61
PD	PD43	bin16	MAG390	343	2746174	41707	11299	8006	87.21	1.97
PD	PD87	bin23	MAG1169	85	2154838	161142	39569	25351	87.2	1.86

PD	PD25	bin62	MAG2479	164	2681251	80509	26911	16349	87.19	3.71
PD	PD40	bin19	MAG942	314	1369625	19906	5132	4362	87.19	1.16
PD	PD11	bin27	MAG915	481	2172775	28581	5683	4517	87.13	1.51
PD	PD14	bin35	MAG2693	484	1475157	12995	3614	3048	87.11	1.2
PD	PD56	bin13	MAG1555	226	2554589	63595	18508	11303	87.1	1.81
PD	PD51	bin29	MAG655	598	1911184	31561	4571	3196	87.08	9.43
PD	PD39	bin24	MAG3071	463	2843937	35892	7739	6142	87.07	0
PD	PD1	bin19	MAG2194	43	2007105	282503	115737	46677	87.02	0.12
PD	PD70	bin10	MAG1901	305	2886051	63726	13997	9462	87	1.75
PD	PD26	bin23	MAG1413	188	1741115	38004	14670	9261	86.92	1.42
PD	PD59	bin49	MAG3174	586	2343374	16941	4727	3999	86.92	2.4
PD	PD70	bin11	MAG1954	105	1851603	99533	26525	17634	86.91	0.67
PD	PD98	bin40	MAG3265	197	1685673	40943	11564	8557	86.9	2.01
PD	PD8	bin59	MAG114	483	1963120	32084	4771	4064	86.89	5.05
PD	PD29	bin58	MAG2308	200	1179136	36104	7962	5896	86.8	2.36
PD	PD68	bin71	MAG245	269	950522	16990	4417	3534	86.8	1.12
PD	PD50	bin29	MAG2865	81	1863939	134660	35144	23012	86.69	0
PD	PD60	bin21	MAG2440	440	2599674	26016	7772	5908	86.63	6.9
PD	PD35	bin27	MAG3247	318	2559881	61184	16567	8050	86.6	1.23
PD	PD29	bin74	MAG3043	46	1754994	153243	59921	38152	86.58	0.67
PD	PD46	bin29	MAG2344	569	1776812	16631	4083	3123	86.58	4.91
PD	PD78	bin24	MAG549	960	3898726	29174	5426	4061	86.52	1.74
PD	PD85	bin8	MAG402	893	2537851	23424	3388	2842	86.49	4.09
PD	PD8	bin23	MAG1323	303	2028200	33851	8694	6694	86.47	0
PD	PD59	bin30	MAG158	516	1706557	23873	4034	3307	86.42	3.4
PD	PD18	bin44	MAG2994	99	2369850	96299	40811	23938	86.39	0.23
PD	PD56	bin42	MAG2945	336	1826357	27177	7452	5436	86.35	0.9
PD	PD62	bin6	MAG1716	320	1785484	26091	6752	5580	86.33	2.69
PD	PD67	bin20	MAG980	288	1829938	45178	9219	6354	86.33	1.26
PD	PD13	bin11	MAG1156	489	1642209	30494	4088	3358	86.32	4.22
PD	PD27	bin33	MAG1355	356	1854837	48491	6970	5210	86.3	0.12
PD	PD71	bin34	MAG1016	662	2215871	15054	3878	3347	86.29	1.84
PD	PD62	bin66	MAG312	672	1712211	19024	2959	2548	86.17	5.68

PD	PD23	bin30	MAG3315	259	1872217	37839	10908	7229	86.1	2.86
PD	PD37	bin26	MAG1165	578	2339231	17239	4669	4047	86.06	3.93
PD	PD78	bin28	MAG1890	296	2409646	59476	12922	8141	86.05	3.69
PD	PD25	bin19	MAG1391	436	2162223	40226	6705	4959	86.04	0.94
PD	PD97	bin65	MAG594	520	1986004	24381	4872	3819	86.04	1.19
PD	PD26	bin26	MAG2201	412	2181916	37554	6622	5296	86.02	1.58
PD	PD53	bin45	MAG1166	388	2024349	23940	6580	5217	85.98	1.72
PD	PD66	bin15	MAG474	215	2229040	51082	16079	10368	85.97	2.85
PD	PD22	bin19	MAG1141	40	1074529	132576	54494	26863	85.96	2.25
PD	PD68	bin65	MAG2332	92	3889496	192561	83694	42277	85.96	1.23
PD	PD24	bin35	MAG2507	41	1776877	199002	85910	43338	85.91	0
PD	PD92	bin51	MAG3387	44	2608963	270805	124541	59295	85.91	0
PD	PD20	bin21	MAG1870	523	1663586	27398	3915	3181	85.87	1.09
PD	PD14	bin4	MAG3075	601	1873351	18665	3781	3117	85.84	1.69
PD	PD10	bin29	MAG2832	253	1067253	18236	5044	4218	85.82	0.67
PD	PD62	bin63	MAG701	475	2390994	23318	6531	5034	85.71	1.08
PD	PD76	bin6	MAG443	776	2332505	21881	3637	3006	85.71	2.27
PD	PD56	bin26	MAG2534	643	2362611	22603	4692	3674	85.69	0.17
PD	PD34	bin42	MAG160	171	1072343	32278	7955	6271	85.67	1.17
PD	PD29	bin24	MAG582	572	1818838	85437	4084	3180	85.66	8.4
PD	PD100	bin12	MAG299	74	1710214	103678	43842	23111	85.64	0.95
PD	PD39	bin52	MAG2892	707	2142030	23070	3495	3030	85.53	0.51
PD	PD56	bin7	MAG2675	396	2106878	29059	6806	5320	85.51	1.68
PD	PD62	bin22	MAG3309	783	2168404	12676	3349	2769	85.51	5.33
PD	PD58	bin11	MAG462	863	2802871	22792	3811	3248	85.5	4.26
PD	PD32	bin77	MAG1186	307	2103244	37353	8819	6851	85.49	1.03
PD	PD34	bin32	MAG3121	316	2014364	40390	8979	6375	85.49	2.01
PD	PD48	bin37	MAG250	614	2141541	25818	4391	3488	85.48	1.12
PD	PD57	bin59	MAG2901	215	2147349	48669	14476	9988	85.48	1.48
PD	PD3	bin25	MAG252	784	2319604	27492	3441	2959	85.47	1.03
PD	PD78	bin17	MAG2493	458	1550700	17388	3907	3386	85.47	1.18
PD	PD32	bin31	MAG1781	660	2605247	32961	5605	3947	85.46	3.04
PD	PD58	bin78	MAG1162	579	2276294	30290	5888	3931	85.4	1.29

PD	PD40	bin55	MAG1320	278	2314343	52896	12609	8325	85.3	1.86
PD	PD51	bin15	MAG2845	477	2211297	44955	5807	4636	85.27	4.27
PD	PD66	bin10	MAG2486	483	2213161	18446	5434	4582	85.27	3.11
PD	PD21	bin21	MAG2749	525	2513737	31271	5754	4788	85.25	0.36
PD	PD32	bin4	MAG1464	752	1999533	13783	3166	2659	85.25	2.42
PD	PD62	bin64	MAG930	173	2053921	67813	22112	11872	85.24	2.21
PD	PD13	bin37	MAG544	147	2273973	97302	24936	15469	85.23	1.01
PD	PD64	bin42	MAG1888	694	2328376	15382	4157	3355	85.23	2.69
PD	PD71	bin2	MAG3463	454	2891164	74507	9099	6368	85.21	6.63
PD	PD12	bin6	MAG1998	529	1782781	18342	4270	3370	85.2	0.75
PD	PD46	bin74	MAG756	485	2081882	17565	5079	4293	85.19	3.63
PD	PD87	bin66	MAG469	591	3436406	77497	12252	5815	85.19	1.73
PD	PD92	bin29	MAG2579	395	1599910	15296	4950	4050	85.14	1.65
PD	PD43	bin53	MAG2231	535	1894474	30351	4675	3541	85.08	5.47
PD	PD47	bin56	MAG2049	431	1649764	16202	4654	3828	85.08	1.98
PD	PD24	bin57	MAG1164	474	2109784	24889	5408	4451	85.02	2.99
PD	PD21	bin17	MAG39	855	1953356	10235	2489	2285	85.01	2.46
PD	PD76	bin60	MAG867	415	2224906	28045	6787	5361	84.99	3.48
PD	PD40	bin34	MAG697	664	2415174	22456	4211	3637	84.97	3.78
PD	PD70	bin16	MAG2927	451	1824609	28375	5230	4046	84.97	1.51
PD	PD9	bin73	MAG2778	513	2081572	28453	5098	4058	84.94	0.88
PD	PD10	bin6	MAG1514	681	2518700	36024	4777	3699	84.93	4.18
PD	PD22	bin58	MAG1665	467	1618452	19851	4212	3466	84.89	2.68
PD	PD64	bin18	MAG3486	637	1961176	16960	3777	3079	84.88	2.98
PD	PD70	bin48	MAG767	381	1966900	27141	7270	5162	84.86	2.43
PD	PD83	bin13	MAG522	768	2021850	16729	2996	2633	84.86	1.19
PD	PD73	bin80	MAG860	267	1097219	21704	4759	4109	84.83	4.49
PD	PD64	bin19	MAG2455	55	2482059	173066	103118	45128	84.8	0.88
PD	PD57	bin21	MAG2915	203	1734575	61863	13454	8545	84.75	1.54
PD	PD25	bin12	MAG2027	254	2248312	39632	12915	8852	84.7	1.07
PD	PD3	bin33	MAG1654	759	2109761	13660	3264	2780	84.65	4.26
PD	PD75	bin36	MAG2511	741	2895595	21842	4252	3908	84.64	1.35
PD	PD64	bin40	MAG2505	382	2829914	45304	9969	7408	84.6	2.15

PD	PD10	bin44	MAG3376	64	2419663	117310	61754	37807	84.54	1.45
PD	PD7	bin34	MAG3352	177	1611300	31738	12452	9103	84.48	0.67
PD	PD76	bin52	MAG24	113	2211133	139932	34932	19568	84.45	4.46
PD	PD39	bin11	MAG301	536	3029686	50379	7821	5652	84.39	0.16
PD	PD2	bin25	MAG1300	350	1728042	33526	6208	4937	84.38	2.57
PD	PD40	bin29	MAG1249	290	2137139	43561	9714	7369	84.36	0.34
PD	PD57	bin10	MAG2953	145	1802201	82100	19915	12429	84.36	0.34
PD	PD9	bin53	MAG182	542	2179047	30133	4741	4020	84.35	1.01
PD	PD45	bin22	MAG578	463	2530181	30079	7145	5465	84.34	0.64
PD	PD61	bin18	MAG298	67	2169318	318282	51270	32378	84.34	0.38
PD	PD67	bin16	MAG167	61	1671708	129082	45089	27405	84.3	0.34
PD	PD12	bin44	MAG849	530	1774858	56841	4194	3349	84.27	2.42
PD	PD3	bin1	MAG2385	479	2253245	31227	5549	4704	84.26	3.69
PD	PD51	bin21	MAG527	620	1716729	14660	3286	2769	84.26	0.69
PD	PD48	bin76	MAG2369	1228	3380815	19495	3276	2753	84.24	2.01
PD	PD22	bin47	MAG1988	422	1845480	21254	5422	4373	84.21	0.99
PD	PD5	bin18	MAG3216	1183	4153825	27623	4028	3511	84.21	9.97
PD	PD41	bin45	MAG993	386	1849640	46098	6055	4792	84.2	5.07
PD	PD64	bin64	MAG178	299	1732853	42177	7372	5795	84.15	3.53
PD	PD9	bin46	MAG199	530	2299128	19583	5033	4338	84.12	0.75
PD	PD29	bin82	MAG1836	280	3138730	124805	32736	11210	84.01	0.74
PD	PD100	bin18	MAG1001	38	1839957	320582	87231	48420	83.89	0
PD	PD34	bin77	MAG2551	79	2447291	139982	60991	30978	83.89	0
PD	PD92	bin11	MAG3495	424	2418068	33489	7409	5703	83.89	1.55
PD	PD62	bin61	MAG1843	567	1875147	16464	3899	3307	83.85	3.23
PD	PD1	bin6	MAG1247	436	1687617	30870	4980	3871	83.84	3.14
PD	PD34	bin52	MAG1629	404	1758099	30670	5540	4352	83.84	3.23
PD	PD13	bin8	MAG2094	765	2134941	11882	3238	2791	83.82	1.06
PD	PD8	bin36	MAG3460	479	1651612	20484	4371	3448	83.82	3.42
PD	PD45	bin7	MAG885	227	1758792	40962	11273	7748	83.79	3.02
PD	PD41	bin72	MAG521	242	1629134	42771	10166	6732	83.75	7.87
PD	PD9	bin69	MAG2031	332	1676572	28804	6503	5050	83.75	3.48
PD	PD47	bin37	MAG1700	440	1738931	22527	4530	3952	83.66	3.23

PD	PD9	bin9	MAG841	220	1623268	52455	10690	7378	83.63	2.29
PD	PD28	bin2	MAG877	289	1957628	46846	9824	6774	83.6	4.06
PD	PD6	bin27	MAG2186	773	2552134	15108	3869	3302	83.59	1.67
PD	PD92	bin44	MAG2413	193	1990131	62787	15526	10312	83.56	0.56
PD	PD29	bin51	MAG1736	127	2253273	128899	33335	17742	83.45	0
PD	PD64	bin30	MAG2956	87	2168926	113488	41305	24930	83.45	1.01
PD	PD13	bin63	MAG3191	355	1863604	25392	6889	5250	83.44	4.42
PD	PD34	bin65	MAG2530	1209	3719906	19175	3606	3077	83.43	1.34
PD	PD4	bin30	MAG350	455	2658108	45898	7627	5842	83.39	2.28
PD	PD91	bin51	MAG1822	112	2793148	156189	57318	24939	83.39	0.37
PD	PD2	bin60	MAG187	356	2178745	36528	9003	6120	83.33	4.22
PD	PD83	bin18	MAG2220	77	2471942	121649	54718	32103	83.33	0
PD	PD46	bin20	MAG726	442	2711093	45582	9603	6134	83.31	0.65
PD	PD8	bin3	MAG970	491	2447386	42919	6039	4984	83.3	6.98
PD	PD22	bin16	MAG734	745	2048867	12071	3321	2750	83.28	1.18
PD	PD20	bin50	MAG574	640	2019228	16977	3889	3155	83.24	1.53
PD	PD45	bin23	MAG2734	166	4238367	221224	88294	25532	83.23	0.19
PD	PD44	bin34	MAG910	286	1659621	43249	7559	5803	83.22	0.67
PD	PD28	bin42	MAG2676	427	1988775	33077	5636	4658	83.2	2.4
PD	PD45	bin66	MAG1909	379	1772457	28370	5714	4677	83.2	3.92
PD	PD82	bin20	MAG2013	936	2478892	12162	3093	2648	83.17	2.18
PD	PD12	bin60	MAG3135	117	1130443	48781	15219	9662	83.15	3.37
PD	PD57	bin7	MAG376	1001	2254294	10523	2497	2252	83.14	5.19
PD	PD75	bin26	MAG3343	759	2174882	14274	3352	2865	83.14	4.66
PD	PD43	bin23	MAG2148	513	1958694	17863	4315	3818	83.13	1.34
PD	PD24	bin25	MAG2942	534	3068462	49348	8007	5746	83.1	3.33
PD	PD29	bin47	MAG1625	526	2368783	24370	5360	4503	83	4.58
PD	PD37	bin15	MAG699	553	1667565	16268	3565	3015	82.96	3.4
PD	PD48	bin25	MAG2293	532	6811325	93425	20088	12803	82.93	9.48
PD	PD2	bin40	MAG1970	406	1986226	20029	6011	4892	82.91	1.93
PD	PD35	bin42	MAG2051	420	3177667	69825	19533	7566	82.84	3
PD	PD62	bin8	MAG2061	334	2139623	40400	8174	6406	82.81	2.55
PD	PD98	bin41	MAG3386	365	1649509	21943	6140	4519	82.81	2.43

PD	PD82	bin13	MAG1067	406	1667553	23646	4692	4107	82.8	2.72
PD	PD77	bin12	MAG3188	1321	3085464	11653	2633	2336	82.79	4.03
PD	PD91	bin11	MAG444	1067	3534401	17604	3958	3312	82.79	2.57
PD	PD73	bin52	MAG1064	96	1511512	148462	35311	15745	82.78	1.97
PD	PD34	bin45	MAG1415	705	1602931	10548	2482	2274	82.76	5.68
PD	PD32	bin76	MAG379	643	2301736	21737	4293	3580	82.74	1.01
PD	PD48	bin69	MAG2064	309	2245658	65220	15172	7268	82.72	2.01
PD	PD71	bin57	MAG1845	932	2196468	20632	2636	2357	82.72	4.42
PD	PD29	bin43	MAG3189	456	2149879	49163	6142	4715	82.7	4.1
PD	PD25	bin9	MAG2211	221	2102235	50182	12004	9512	82.55	0.73
PD	PD47	bin9	MAG1844	31	1816205	218216	113949	58587	82.55	0.67
PD	PD2	bin55	MAG2319	459	2363316	36738	9363	5149	82.53	5.06
PD	PD32	bin27	MAG2062	362	1721757	30806	5850	4756	82.53	5.61
PD	PD58	bin37	MAG3179	565	2871087	30888	6210	5082	82.42	2.48
PD	PD68	bin8	MAG2260	177	1920573	62713	16895	10851	82.38	0.68
PD	PD25	bin7	MAG430	444	1842305	32289	4874	4149	82.34	6.4
PD	PD69	bin33	MAG2340	259	2020318	60921	15068	7800	82.3	1.85
PD	PD34	bin20	MAG333	153	2911816	93320	31300	19031	82.26	1.04
PD	PD9	bin25	MAG2496	412	1891935	29326	5158	4592	82.22	4.75
PD	PD99	bin8	MAG3347	461	1706647	18723	4181	3702	82.18	2.08
PD	PD17	bin18	MAG2391	848	3208236	22870	4320	3783	82.17	1.36
PD	PD5	bin14	MAG1809	222	1566131	36836	9891	7055	82.17	3.36
PD	PD58	bin16	MAG3161	600	1944850	38359	3853	3241	82.16	2.67
PD	PD12	bin40	MAG1752	313	1957831	39618	9249	6255	82.12	1.17
PD	PD62	bin57	MAG318	365	1618878	31758	5233	4435	82.09	1.7
PD	PD26	bin14	MAG2147	409	1610526	46010	5644	3938	82.05	4.77
PD	PD44	bin11	MAG146	635	2502394	21330	4761	3941	81.97	2.16
PD	PD12	bin66	MAG1726	532	2022575	24619	4576	3802	81.95	0.67
PD	PD68	bin23	MAG3271	451	2129022	24010	5850	4721	81.9	0
PD	PD16	bin13	MAG1552	67	1736929	93063	42693	25924	81.88	1.34
PD	PD29	bin44	MAG2484	473	1880567	18445	4688	3976	81.88	0.96
PD	PD91	bin22	MAG2954	261	2209285	67121	12905	8465	81.88	0.72
PD	PD73	bin41	MAG1546	529	1569492	13387	3472	2967	81.82	1.76

PD	PD20	bin3	MAG2552	324	1776998	31499	7328	5485	81.68	2
PD	PD27	bin16	MAG603	537	1634546	28130	3857	3044	81.67	0.89
PD	PD1	bin9	MAG2408	503	2261037	23186	5432	4495	81.66	7.66
PD	PD99	bin39	MAG567	74	1834681	107640	39785	24793	81.63	1.58
PD	PD22	bin38	MAG2873	708	2193934	15790	3871	3099	81.62	1.39
PD	PD86	bin27	MAG1919	443	2094065	38594	5662	4727	81.58	1.18
PD	PD68	bin60	MAG2092	666	1874573	15829	3332	2815	81.49	1.72
PD	PD21	bin27	MAG1095	1264	3685067	42666	3621	2915	81.47	4.29
PD	PD12	bin24	MAG3396	341	1626855	20296	5562	4771	81.42	1.42
PD	PD23	bin3	MAG1997	177	2598810	97484	26047	14683	81.41	0
PD	PD70	bin6	MAG1339	273	2763393	63419	14439	10122	81.35	2.7
PD	PD9	bin23	MAG2575	409	2189146	23430	6585	5352	81.32	3.04
PD	PD8	bin6	MAG890	150	2679513	78687	33732	17863	81.31	1.86
PD	PD100	bin36	MAG1573	308	1867044	26305	8151	6062	81.21	6.21
PD	PD92	bin30	MAG2539	46	1766775	325657	85026	38408	81.21	0
PD	PD64	bin92	MAG1849	390	1639272	24486	5188	4203	81.2	1.61
PD	PD8	bin60	MAG1112	912	2373305	11739	3032	2602	81.17	1.96
PD	PD51	bin24	MAG3097	588	2564480	32581	5249	4361	81.1	4.11
PD	PD42	bin13	MAG3280	407	1980238	43012	7207	4865	81.04	3.22
PD	PD14	bin27	MAG2141	136	3289258	140895	47160	24186	81.03	0
PD	PD19	bin24	MAG175	89	2618236	136321	50552	29418	81.03	0
PD	PD23	bin42	MAG2585	456	2000033	29149	5301	4386	81.02	6.99
PD	PD25	bin21	MAG11	169	1717928	57874	14788	10165	80.98	0.34
PD	PD31	bin2	MAG1470	859	2476519	14111	3314	2883	80.97	1.51
PD	PD56	bin19	MAG2634	848	2632874	23676	3525	3105	80.91	2.18
PD	PD1	bin34	MAG3136	44	2568619	275355	102784	58378	80.9	0
PD	PD53	bin13	MAG300	141	2145708	70345	23954	15218	80.88	0
PD	PD61	bin6	MAG558	260	1806249	37923	10947	6947	80.87	0.75
PD	PD70	bin50	MAG65	137	1655361	80802	20091	12083	80.87	0
PD	PD98	bin20	MAG3240	585	2767269	35793	5643	4730	80.87	1.67
PD	PD41	bin44	MAG621	27	1334994	181029	105704	49444	80.86	1.4
PD	PD94	bin17	MAG3296	45	1331712	115707	54138	29594	80.86	1.4
PD	PD41	bin32	MAG2528	673	1610705	11846	2702	2393	80.83	4.81

PD	PD61	bin60	MAG1702	493	1325973	14339	3136	2690	80.83	0.35
PD	PD12	bin64	MAG686	362	1828668	25015	6428	5052	80.82	1.53
PD	PD41	bin15	MAG3306	673	1707643	15648	2878	2537	80.82	8.49
PD	PD100	bin38	MAG154	655	2826451	22062	5014	4315	80.8	1.73
PD	PD97	bin71	MAG1261	364	1595829	22344	5126	4384	80.8	4.32
PD	PD37	bin25	MAG3450	714	2205700	19791	3571	3089	80.75	0.32
PD	PD56	bin23	MAG3484	223	2539809	66148	16638	11389	80.72	1.45
PD	PD94	bin33	MAG3283	588	2409930	27520	5096	4099	80.7	0.89
PD	PD14	bin20	MAG3141	381	2168118	37461	6250	5691	80.65	2.72
PD	PD57	bin85	MAG3195	75	1903249	97364	45400	25377	80.65	1.34
PD	PD87	bin31	MAG3178	366	2103612	34585	7888	5748	80.65	0.61
PD	PD44	bin4	MAG1485	379	2211527	42317	7533	5835	80.59	0.95
PD	PD68	bin20	MAG959	674	1646629	12054	2750	2443	80.59	2.31
PD	PD3	bin56	MAG2780	119	3803766	219175	50967	31964	80.52	0
PD	PD77	bin64	MAG1444	96	4064950	150135	69660	42343	80.52	0
PD	PD61	bin25	MAG1361	526	2282683	26497	5212	4340	80.47	4.47
PD	PD82	bin28	MAG2242	249	4946494	143889	33840	19865	80.45	1.56
PD	PD12	bin42	MAG776	450	2310776	65688	6178	5135	80.44	1.01
PD	PD13	bin30	MAG408	47	1314695	109267	66359	27972	80.36	0.89
PD	PD68	bin45	MAG454	312	1119471	15790	3979	3588	80.34	0.56
PD	PD75	bin12	MAG2457	500	1627681	15866	4164	3255	80.29	4.04
PD	PD1	bin1	MAG3365	25	1553521	162523	80801	62141	80.27	0
PD	PD58	bin20	MAG664	112	1915046	71934	25694	17099	80.2	0.67
PD	PD12	bin37	MAG2628	67	1390266	139801	110417	20750	80.16	1.05
PD	PD64	bin16	MAG1771	53	1390460	129951	57960	26235	80.16	1.05
PD	PD77	bin4	MAG1763	275	2735642	58601	16519	9948	80.06	2.05
PD	PD47	bin10	MAG2082	192	1954120	55229	16415	10178	80.03	9.17
PD	PD53	bin43	MAG2114	248	1778596	29385	8366	7172	80.02	1.02
PD	PD78	bin4	MAG2481	516	1923625	25080	4395	3728	80.02	1.27
PD	PD100	bin44	MAG224	164	2021834	64346	20391	12328	79.98	0
PD	PD87	bin32	MAG753	446	1877203	25488	4930	4209	79.98	3.36
PD	PD35	bin17	MAG62	236	2212472	94548	20337	9375	79.97	1.79
PD	PD32	bin12	MAG450	545	1799027	14423	4116	3301	79.93	3.7

PD	PD3	bin16	MAG1158	584	2300869	22577	4929	3940	79.84	2.86
PD	PD67	bin9	MAG3300	206	2016630	61900	18736	9789	79.84	0
PD	PD12	bin22	MAG2759	87	3869740	195310	71045	44480	79.81	3.45
PD	PD46	bin77	MAG1192	878	2046142	9331	2589	2330	79.81	2.49
PD	PD4	bin13	MAG639	788	2070198	17361	3048	2627	79.74	3.47
PD	PD83	bin30	MAG801	702	1932636	16543	3198	2753	79.71	1.98
PD	PD40	bin21	MAG2176	800	2075358	11736	2995	2594	79.68	1.15
PD	PD57	bin55	MAG1383	637	1946292	17552	3597	3055	79.67	1.43
PD	PD95	bin27	MAG354	68	3952399	264503	93226	58124	79.66	0
PD	PD87	bin64	MAG309	425	2510299	25503	6739	5907	79.65	1.38
PD	PD82	bin31	MAG121	732	2167618	45637	3612	2961	79.62	3.75
PD	PD51	bin42	MAG2175	284	3041799	126580	51573	10711	79.6	1.63
PD	PD42	bin2	MAG127	454	2517969	34549	6720	5546	79.57	0.63
PD	PD13	bin55	MAG1194	274	2420307	64658	12920	8833	79.55	2.35
PD	PD20	bin22	MAG322	515	1530638	16535	3432	2972	79.55	0.67
PD	PD44	bin17	MAG943	587	1743729	17924	3472	2971	79.5	1.59
PD	PD70	bin37	MAG1054	602	1976683	16104	3733	3284	79.5	2.48
PD	PD95	bin6	MAG1041	406	2086011	35511	6390	5138	79.41	0.6
PD	PD41	bin28	MAG2054	574	1479723	17589	2822	2578	79.36	3.18
PD	PD46	bin4	MAG2799	521	1474467	20240	3323	2830	79.24	2.47
PD	PD22	bin39	MAG762	58	821297	72967	29579	14160	79.21	0.16
PD	PD6	bin33	MAG2453	378	2019103	27935	6790	5342	79.16	2.04
PD	PD12	bin35	MAG615	669	1659248	13875	2857	2480	79.13	0.23
PD	PD12	bin39	MAG1897	392	1638993	25948	5120	4181	79	2.9
PD	PD69	bin37	MAG124	152	2251372	116651	49324	14812	78.9	1.9
PD	PD77	bin36	MAG2003	617	2544288	19669	5184	4124	78.9	2.52
PD	PD92	bin54	MAG1338	613	4426670	58479	11440	7221	78.83	2.84
PD	PD13	bin23	MAG2029	451	1038275	7751	2553	2302	78.82	4.04
PD	PD32	bin46	MAG2489	608	1794921	19335	3623	2952	78.82	1.41
PD	PD22	bin5	MAG698	35	1325795	208209	64219	37880	78.79	0.89
PD	PD47	bin47	MAG33	54	1612630	123403	58346	29864	78.79	1.34
PD	PD14	bin26	MAG2041	347	1785582	25477	6724	5146	78.74	3.71
PD	PD22	bin4	MAG3269	400	1365439	16762	3731	3414	78.73	1.08

PD	PD32	bin75	MAG932	716	1710457	11510	2686	2389	78.72	3.66
PD	PD97	bin31	MAG1911	46	1519444	129274	58336	33031	78.68	0.67
PD	PD44	bin19	MAG1664	212	2318922	105839	33142	10938	78.65	0.58
PD	PD14	bin15	MAG67	488	2373716	33380	6230	4864	78.63	4.86
PD	PD3	bin22	MAG2435	59	1964573	241761	98967	33298	78.52	0
PD	PD64	bin75	MAG532	64	1945160	360711	126847	30393	78.52	1.01
PD	PD68	bin13	MAG835	43	1823889	281954	113679	42416	78.52	0
PD	PD97	bin55	MAG1749	63	1828232	250490	122719	29020	78.52	0.34
PD	PD50	bin59	MAG2546	898	2103649	11732	2630	2343	78.5	2.65
PD	PD37	bin54	MAG1854	140	5864522	247043	69018	41889	78.45	0
PD	PD24	bin39	MAG1586	624	2036941	24892	4300	3264	78.3	2.08
PD	PD67	bin11	MAG3398	454	1540612	24075	4269	3393	78.3	0.16
PD	PD29	bin71	MAG1068	556	1630145	18928	3559	2932	78.25	5.03
PD	PD2	bin41	MAG1134	732	1930123	12954	3067	2637	78.22	1.82
PD	PD39	bin6	MAG881	611	1590746	20642	2959	2604	78.21	1.67
PD	PD91	bin30	MAG2472	193	3522475	114591	31342	18251	78.21	0.53
PD	PD12	bin30	MAG846	57	1421879	118992	43292	24945	78.19	0
PD	PD71	bin20	MAG2330	582	4692737	157427	16870	8063	78.17	5.21
PD	PD50	bin50	MAG276	25	1255018	269496	97024	50201	78.12	0.22
PD	PD99	bin25	MAG2466	207	1487503	43773	9015	7186	77.99	3.62
PD	PD37	bin46	MAG977	1269	2659605	11716	2234	2096	77.98	1.5
PD	PD97	bin1	MAG1827	634	1832594	13849	3337	2891	77.98	2.91
PD	PD32	bin50	MAG2581	156	1782765	64284	18428	11428	77.97	0.67
PD	PD12	bin72	MAG1816	571	1676408	19878	3266	2936	77.95	0.56
PD	PD70	bin1	MAG1418	490	2722324	33791	7329	5556	77.93	4.01
PD	PD22	bin17	MAG2202	56	1381339	119320	32948	24667	77.9	4.92
PD	PD2	bin29	MAG1229	642	1443437	9383	2534	2248	77.89	1.86
PD	PD97	bin56	MAG363	160	2879825	130769	43118	17999	77.86	0.56
PD	PD23	bin34	MAG2347	62	1691179	167409	43775	27277	77.85	0
PD	PD44	bin27	MAG821	78	1887312	95826	46710	24196	77.85	0.63
PD	PD36	bin40	MAG3008	492	1975919	18415	4624	4016	77.81	1.57
PD	PD78	bin16	MAG2510	498	2030722	26840	4755	4078	77.81	0.83
PD	PD91	bin45	MAG3169	699	1618318	9263	2509	2315	77.77	3.87

PD	PD22	bin12	MAG3334	182	897876	32372	5975	4933	77.76	0.21
PD	PD41	bin60	MAG1642	156	2648161	111212	25272	16975	77.76	0
PD	PD6	bin14	MAG1696	579	2400223	36382	4788	4145	77.76	3.16
PD	PD10	bin33	MAG2355	616	1313902	8549	2394	2133	77.73	1.16
PD	PD16	bin51	MAG2838	543	1890561	14442	3860	3482	77.71	1.34
PD	PD57	bin60	MAG2973	147	1802316	84109	25448	12261	77.71	1.7
PD	PD6	bin21	MAG586	1641	4022094	11327	2792	2451	77.7	2.85
PD	PD47	bin59	MAG2647	156	1987270	89264	19801	12739	77.68	0.89
PD	PD97	bin67	MAG2412	902	2387447	12632	3110	2647	77.67	2.45
PD	PD58	bin80	MAG2527	439	1735881	19510	4558	3954	77.66	1.79
PD	PD18	bin11	MAG2716	149	2400248	111411	47932	16109	77.63	0.67
PD	PD66	bin5	MAG862	219	2814954	67006	23234	12854	77.62	0.02
PD	PD20	bin4	MAG2863	324	2474571	55046	12419	7638	77.59	0
PD	PD20	bin53	MAG1994	1107	2645565	10631	2742	2390	77.56	9.97
PD	PD16	bin37	MAG3332	392	1591204	22088	4793	4059	77.53	2.52
PD	PD82	bin60	MAG1918	469	1821455	21349	4669	3884	77.52	2.03
PD	PD6	bin28	MAG31	451	1618607	13920	4098	3589	77.48	0
PD	PD72	bin20	MAG316	800	3213831	21821	4747	4017	77.46	1.44
PD	PD22	bin37	MAG2473	29	1172480	156342	90562	40430	77.45	0.22
PD	PD64	bin35	MAG1793	52	1737605	134249	46572	33415	77.42	0
PD	PD97	bin77	MAG3452	360	1675108	31658	5766	4653	77.42	1.12
PD	PD20	bin33	MAG1200	722	1883557	11455	3133	2609	77.37	0.25
PD	PD73	bin59	MAG539	29	1230251	147272	83153	42422	77.37	2.45
PD	PD25	bin14	MAG1449	98	2606417	118967	40525	26596	77.35	2.19
PD	PD59	bin41	MAG1862	303	3622585	58646	15217	11956	77.35	2.51
PD	PD29	bin61	MAG1630	937	2742391	23146	3399	2927	77.33	1.61
PD	PD68	bin46	MAG1659	622	1328712	10559	2343	2136	77.33	0.06
PD	PD29	bin79	MAG1964	252	1936161	42626	10373	7683	77.31	0
PD	PD29	bin31	MAG34	666	1691356	11890	2883	2540	77.31	2.06
PD	PD61	bin57	MAG3243	464	2102951	18087	5161	4532	77.26	1.7
PD	PD37	bin24	MAG642	1046	2888867	16415	3189	2762	77.24	1.19
PD	PD64	bin62	MAG1941	638	1920374	18861	3809	3010	77.24	3.49
PD	PD25	bin22	MAG2317	270	2407668	70700	13874	8917	76.91	0.5

PD	PD61	bin17	MAG679	50	1627589	204412	71810	32552	76.85	0.67
PD	PD91	bin52	MAG2731	654	1299051	8748	2097	1986	76.82	1.51
PD	PD41	bin68	MAG3298	106	1636364	85979	27525	15437	76.78	2.57
PD	PD2	bin27	MAG2143	827	2164498	19511	2915	2617	76.72	6.88
PD	PD59	bin22	MAG1009	745	1606134	10876	2360	2156	76.63	2.64
PD	PD40	bin47	MAG2050	839	1965069	11341	2717	2342	76.52	9.47
PD	PD50	bin27	MAG1979	31	1526049	192182	86316	49227	76.51	0
PD	PD99	bin31	MAG1094	438	1672644	36591	4791	3819	76.49	2.1
PD	PD81	bin27	MAG1828	1118	2716919	12883	2723	2430	76.38	1.71
PD	PD28	bin21	MAG2458	127	2155726	114531	47374	16974	76.36	2.25
PD	PD97	bin38	MAG2285	207	3443777	166466	49422	16637	76.33	1.79
PD	PD37	bin34	MAG2401	50	1879639	248732	108959	37593	76.29	0.67
PD	PD4	bin34	MAG2911	399	1507344	14512	4270	3778	76.29	3.64
PD	PD76	bin46	MAG3454	661	2300179	14337	3937	3480	76.27	4.19
PD	PD8	bin4	MAG1737	104	2934699	187180	44856	28218	76.21	0.34
PD	PD20	bin36	MAG106	411	1732269	24282	4786	4215	76.07	9.64
PD	PD39	bin37	MAG2227	598	1625727	13480	3116	2719	76.07	2.01
PD	PD10	bin42	MAG1939	1047	3056352	17089	3678	2919	76.06	6.75
PD	PD91	bin16	MAG2492	493	1684278	34504	4254	3416	76.06	1.01
PD	PD81	bin21	MAG552	688	2335470	16011	3813	3395	75.99	0.94
PD	PD62	bin21	MAG2674	318	1500437	22556	5540	4718	75.97	2.86
PD	PD50	bin20	MAG3211	585	2072833	19294	4678	3543	75.96	4.33
PD	PD2	bin17	MAG155	153	1745436	109744	16053	11408	75.94	2.53
PD	PD75	bin29	MAG207	579	1713832	15920	3375	2960	75.91	2.14
PD	PD61	bin34	MAG979	800	2097924	12513	3102	2622	75.89	1.68
PD	PD42	bin22	MAG2367	690	2025405	18398	3402	2935	75.86	5.17
PD	PD64	bin12	MAG2615	588	1810012	14679	3696	3078	75.69	1.68
PD	PD58	bin19	MAG283	439	1379537	11552	3515	3142	75.58	3.97
PD	PD97	bin14	MAG2735	702	1832042	13967	3077	2610	75.57	3.54
PD	PD24	bin12	MAG2874	585	1424310	13514	2806	2435	75.56	1.31
PD	PD9	bin64	MAG2299	195	1472495	39264	10678	7551	75.55	2.46
PD	PD2	bin32	MAG3273	24	1525303	190963	129933	63554	75.5	0
PD	PD35	bin32	MAG1298	1159	2559238	10818	2398	2208	75.49	2.51

PD	PD46	bin53	MAG888	490	2232280	25330	5338	4556	75.38	0.92
PD	PD57	bin62	MAG1412	344	1906827	28111	6286	5543	75.26	0.89
PD	PD60	bin19	MAG2680	937	2160137	10469	2531	2305	75.25	1.2
PD	PD100	bin39	MAG70	429	2740937	26968	7841	6389	75.24	2.76
PD	PD25	bin37	MAG1280	45	1605882	200324	78939	35686	75.21	3.13
PD	PD64	bin14	MAG803	242	1522138	56908	8381	6290	75.1	2.73
PD	PD68	bin16	MAG2781	105	1441188	68753	22598	13726	75.1	2.71
PD	PD57	bin19	MAG2363	736	1874561	14308	2884	2547	75.06	1.03
PD	PD86	bin7	MAG1451	693	1708309	11045	2826	2465	75.03	3.98
PD	PD95	bin33	MAG3310	578	1572084	19025	3308	2720	74.96	1.21
PD	PD22	bin6	MAG41	235	2521405	76056	17099	10729	74.95	0.87
PD	PD18	bin20	MAG2587	771	1626901	9319	2261	2110	74.92	2.07
PD	PD13	bin4	MAG1049	556	1776574	10485	3462	3195	74.83	0.84
PD	PD71	bin21	MAG1745	526	2142709	23674	4797	4074	74.79	3.81
PD	PD19	bin37	MAG1992	43	1537315	222345	66608	35752	74.77	0.67
PD	PD99	bin38	MAG3502	313	1587746	115956	8154	5073	74.76	6.37
PD	PD20	bin34	MAG323	1149	2454420	10751	2310	2136	74.75	2.61
PD	PD10	bin24	MAG2020	610	1487388	12859	2786	2438	74.68	1.72
PD	PD18	bin16	MAG1840	1234	4324436	21916	3872	3504	74.58	2.52
PD	PD69	bin45	MAG1098	839	1852944	11056	2440	2209	74.56	2.41
PD	PD87	bin10	MAG1675	599	2029122	21504	4702	3388	74.56	6.3
PD	PD24	bin18	MAG193	211	2045746	51618	12685	9695	74.46	3.57
PD	PD37	bin5	MAG2222	979	2281309	10784	2550	2330	74.46	1.14
PD	PD70	bin52	MAG1892	203	1538980	39341	11902	7581	74.43	0.34
PD	PD17	bin28	MAG2009	581	2305608	19427	4418	3968	74.42	4.49
PD	PD91	bin23	MAG3344	632	1521127	12736	2735	2407	74.33	3.59
PD	PD45	bin31	MAG1791	178	1947602	54426	16098	10942	74.27	0
PD	PD56	bin43	MAG501	737	1904852	18709	3060	2585	74.27	0.06
PD	PD20	bin40	MAG22	350	1345888	18670	4437	3845	74.2	0.89
PD	PD12	bin45	MAG1253	357	1917012	25513	6482	5370	74.17	0.16
PD	PD64	bin44	MAG1819	28	1466234	174740	97720	52366	74.16	0
PD	PD87	bin65	MAG1606	36	1519118	228486	56084	42198	74.16	0
PD	PD91	bin37	MAG780	101	4375588	209777	80883	43323	74.14	0.86

PD	PD31	bin42	MAG1365	204	1904618	40674	13273	9336	74.11	0
PD	PD91	bin32	MAG189	605	1373663	10895	2612	2271	74.1	0.76
PD	PD69	bin46	MAG3033	646	1611826	10463	2741	2495	74.06	3.65
PD	PD7	bin2	MAG1940	970	2568622	12773	3022	2648	74.04	0
PD	PD46	bin38	MAG3207	786	1868575	15592	2614	2377	73.97	0.91
PD	PD62	bin25	MAG2925	923	2292767	14935	2793	2484	73.94	2.8
PD	PD32	bin82	MAG1532	192	2245825	112860	17587	11697	73.84	1.75
PD	PD47	bin54	MAG2216	60	1578463	244356	106003	26308	73.83	0.34
PD	PD82	bin7	MAG194	298	2172938	38542	9574	7292	73.73	1.79
PD	PD13	bin38	MAG2616	225	1106802	27156	6055	4919	73.64	2.06
PD	PD92	bin61	MAG3408	599	2196394	18574	4104	3667	73.6	7.12
PD	PD62	bin60	MAG960	182	1987195	61641	16473	10919	73.57	0
PD	PD60	bin2	MAG1020	31	1513455	253832	130068	48821	73.49	0
PD	PD86	bin16	MAG2977	784	1851902	32068	2634	2362	73.45	3.3
PD	PD72	bin44	MAG956	104	1582369	164429	56525	15215	73.42	0.63
PD	PD5	bin38	MAG1526	662	2037175	11997	3371	3077	73.4	1.75
PD	PD57	bin41	MAG1789	55	1344994	134509	33090	24454	73.39	1.61
PD	PD37	bin44	MAG1694	111	2057393	95859	33359	18535	73.15	0.67
PD	PD35	bin19	MAG1077	546	1746966	15101	3513	3200	73.11	1.01
PD	PD25	bin47	MAG484	323	1571470	22308	6001	4865	73.09	1.56
PD	PD60	bin4	MAG3286	718	1588881	10832	2425	2213	72.96	2.72
PD	PD97	bin54	MAG1443	397	4871982	93022	20386	12272	72.92	1.72
PD	PD59	bin58	MAG3245	535	1252557	9976	2594	2341	72.86	0.63
PD	PD48	bin40	MAG2404	733	1526394	8373	2203	2082	72.83	3.43
PD	PD9	bin47	MAG389	782	1902489	20650	2777	2433	72.79	4.06
PD	PD19	bin25	MAG2619	1411	4669608	23167	3557	3309	72.72	1.8
PD	PD41	bin51	MAG2719	628	2055403	17411	3579	3273	72.72	1.97
PD	PD34	bin2	MAG2568	178	1780424	43572	13971	10002	72.68	4.46
PD	PD60	bin28	MAG2294	533	1680272	15290	3745	3152	72.54	1.46
PD	PD82	bin11	MAG921	802	1632078	9674	2184	2035	72.53	4.04
PD	PD1	bin33	MAG1096	284	2284167	88917	15665	8043	72.44	1.14
PD	PD64	bin36	MAG3063	585	1739667	10539	3167	2974	72.44	0.51
PD	PD94	bin73	MAG2467	644	1955996	19274	3185	3037	72.39	5.88

PD	PD35	bin48	MAG2099	691	1964829	37065	3356	2843	72.37	2.07
PD	PD12	bin18	MAG3238	597	1444710	10393	2681	2420	72.32	4.82
PD	PD25	bin54	MAG3080	736	1608051	8355	2398	2185	72.28	2.81
PD	PD5	bin44	MAG1946	885	2159830	14057	2703	2440	72.26	1.64
PD	PD35	bin1	MAG2044	657	1384393	8041	2274	2107	72	3.19
PD	PD39	bin8	MAG1335	891	1801526	8580	2168	2022	72	1.08
PD	PD48	bin45	MAG2263	295	1942228	40935	9793	6584	71.93	3.51
PD	PD1	bin37	MAG347	971	1634656	6118	1670	1683	71.83	2.45
PD	PD45	bin8	MAG1386	25	1447138	243517	107082	57886	71.81	0
PD	PD48	bin16	MAG831	1099	4676684	65878	5174	4255	71.8	8.77
PD	PD10	bin17	MAG1797	396	1471348	13068	4095	3716	71.72	2.84
PD	PD75	bin38	MAG1597	376	4646754	301503	25018	12358	71.71	1.72
PD	PD8	bin67	MAG1705	596	1499879	19906	2848	2517	71.7	3.83
PD	PD20	bin47	MAG3093	1319	2662845	9674	2130	2019	71.67	2.29
PD	PD47	bin30	MAG92	617	1687100	15457	3254	2734	71.66	1.77
PD	PD37	bin42	MAG345	206	2694168	139783	24715	13078	71.6	0.02
PD	PD14	bin22	MAG240	602	2825297	23360	6144	4693	71.55	0.66
PD	PD48	bin61	MAG289	485	1731949	18812	4319	3571	71.54	0.42
PD	PD62	bin69	MAG2283	562	1486267	13903	3133	2645	71.49	1.03
PD	PD46	bin75	MAG2753	30	1416016	159144	70848	47201	71.48	0.06
PD	PD39	bin45	MAG1523	233	1206044	68480	6176	5176	71.41	1.01
PD	PD92	bin50	MAG2426	801	1742058	15518	2397	2175	71.41	3.16
PD	PD32	bin37	MAG1452	386	1938089	24205	6104	5021	71.38	3.57
PD	PD72	bin5	MAG84	511	1511709	12423	3501	2958	71.36	1.4
PD	PD20	bin28	MAG897	420	1507711	32504	5520	3590	71.31	7.31
PD	PD7	bin5	MAG2125	589	1295122	10152	2456	2199	71.31	0.7
PD	PD43	bin31	MAG3177	893	1964559	9781	2388	2200	71.24	2.4
PD	PD83	bin40	MAG988	540	1426347	19396	2921	2641	71.22	5.27
PD	PD9	bin30	MAG115	608	1336877	9347	2430	2199	71.09	1.09
PD	PD59	bin3	MAG3037	803	1604399	9289	2128	1998	71.06	2.32
PD	PD37	bin32	MAG1596	719	1708102	10623	2690	2376	71.05	3.21
PD	PD58	bin17	MAG1609	1160	3416814	15170	3049	2946	71.04	5.59
PD	PD31	bin61	MAG3108	401	1705759	25743	4854	4254	70.99	2.16

PD	PD64	bin25	MAG1260	658	1733914	14864	2998	2635	70.95	1.46
PD	PD71	bin22	MAG2989	415	1436262	17333	4216	3461	70.89	2.71
PD	PD70	bin55	MAG1035	401	1441622	13840	4190	3595	70.83	3.59
PD	PD28	bin29	MAG1995	28	1361795	374583	121591	48636	70.81	0
PD	PD53	bin15	MAG1871	31	1539311	251326	94443	49655	70.81	0
PD	PD64	bin26	MAG3430	968	1983625	10857	2249	2049	70.79	7.12
PD	PD16	bin64	MAG3501	422	1610855	39134	4459	3817	70.71	2.53
PD	PD31	bin12	MAG3426	122	1915789	54844	25632	15703	70.69	0
PD	PD39	bin3	MAG1357	734	1822569	21296	2750	2483	70.53	3.53
PD	PD57	bin24	MAG1139	510	1199543	10315	2623	2352	70.52	2.35
PD	PD72	bin14	MAG3488	567	1500042	11281	2999	2646	70.51	1.01
PD	PD68	bin14	MAG423	415	1615113	16470	4453	3892	70.47	1.12
PD	PD40	bin32	MAG2145	841	2270021	12274	2958	2699	70.46	3.34
PD	PD75	bin10	MAG2987	1018	1869822	10221	1896	1837	70.45	3.96
PD	PD68	bin62	MAG3364	67	1286896	100294	27741	19207	70.4	0.67
PD	PD5	bin31	MAG3436	1337	2898997	11841	2354	2168	70.29	3.87
PD	PD39	bin28	MAG2589	884	1850766	9072	2290	2094	70.26	4.15
PD	PD25	bin36	MAG2139	568	1605398	15380	3175	2826	70.23	1.74
PD	PD42	bin9	MAG695	522	2479293	55919	8607	4750	70.18	0.99
PD	PD72	bin54	MAG1302	33	1592686	171866	96766	48263	70.18	0
PD	PD61	bin10	MAG844	999	2041204	13436	2162	2043	70.14	0.83
PD	PD73	bin31	MAG1030	811	1746718	10753	2321	2154	70.14	4.79
PD	PD76	bin40	MAG3439	612	1949437	13684	3562	3185	70.14	0.38
PD	PD13	bin22	MAG1519	344	2415439	44991	9194	7022	70.12	1.44
PD	PD64	bin57	MAG369	599	1536405	27304	2933	2565	70.12	5.11
PD	PD97	bin64	MAG3143	186	2262377	67950	13484	12163	70.1	1.35
PD	PD51	bin20	MAG1424	504	1827197	22279	5421	3625	70.09	4.54
PD	PD94	bin51	MAG496	1440	3020616	8022	2292	2098	70.07	2.96
PD	PD10	bin20	MAG1387	209	1338931	56688	9422	6406	70.02	0.67
PD	PD9	bin74	MAG426	338	1506063	23332	5316	4456	70	4.31
PD	PD57	bin44	MAG1830	81	1504804	80064	29764	18578	69.97	0.13
PD	PD58	bin7	MAG1958	763	1548868	9377	2238	2030	69.76	1.62
PD	PD69	bin12	MAG2035	1296	2388581	13900	1875	1843	69.72	3.04

PD	PD97	bin68	MAG612	881	1671757	8625	1985	1898	69.63	6.93
PD	PD26	bin5	MAG1048	545	1967976	18676	4218	3611	69.56	1.51
PD	PD9	bin58	MAG1252	577	1978925	74463	4539	3430	69.5	2.98
PD	PD9	bin65	MAG2610	36	1649271	235688	127674	45813	69.46	0
PD	PD73	bin65	MAG1342	716	1627026	10563	2511	2272	69.35	2.19
PD	PD32	bin3	MAG3345	412	1870188	57152	8821	4539	69.34	7.83
PD	PD87	bin39	MAG536	190	1809737	44516	14750	9525	69.3	3.51
PD	PD41	bin40	MAG2658	83	1663608	138180	35858	20043	69.24	0
PD	PD25	bin5	MAG205	789	1673372	10836	2286	2121	69.22	1.28
PD	PD64	bin56	MAG3223	823	1800861	11250	2356	2188	69	3.08
PD	PD41	bin47	MAG55	698	1486948	9647	2308	2130	68.97	2.8
PD	PD56	bin27	MAG2070	374	1574613	24656	5547	4210	68.97	0
PD	PD44	bin57	MAG2810	207	1277762	61823	9060	6173	68.96	2.69
PD	PD48	bin26	MAG509	234	2176643	41807	12983	9302	68.91	1.79
PD	PD53	bin25	MAG2608	735	2858439	16853	4372	3889	68.89	1.72
PD	PD73	bin37	MAG2756	624	1550849	17306	2773	2485	68.7	3.01
PD	PD44	bin2	MAG1908	670	1409855	9814	2209	2104	68.67	1.56
PD	PD12	bin63	MAG3406	594	1375048	9766	2541	2315	68.65	1.45
PD	PD70	bin18	MAG847	471	1644551	14004	4107	3492	68.64	0.79
PD	PD21	bin24	MAG1281	1118	4993154	40078	6308	4466	68.61	7.76
PD	PD37	bin33	MAG1433	56	2264803	364974	231604	40443	68.55	0.81
PD	PD77	bin21	MAG2817	77	2926774	128529	57647	38010	68.45	0
PD	PD91	bin49	MAG3209	462	1617619	17399	3772	3501	68.45	0
PD	PD6	bin20	MAG1935	314	1934809	29074	8168	6162	68.43	0.97
PD	PD87	bin4	MAG3003	296	2053398	28087	10113	6937	68.42	0
PD	PD11	bin4	MAG359	736	2208272	18557	3350	3000	68.32	1.53
PD	PD67	bin17	MAG367	461	1829770	23927	4513	3969	68.3	3
PD	PD100	bin19	MAG866	108	2147898	166992	53428	19888	68.24	0.63
PD	PD28	bin56	MAG1394	956	2072122	10865	2444	2167	68.21	7.34
PD	PD3	bin7	MAG2517	1214	2132256	8557	1769	1756	68.2	1.71
PD	PD58	bin13	MAG601	429	1295251	13341	3192	3019	68.1	0.81
PD	PD57	bin56	MAG3446	432	1955302	26719	5326	4526	68.08	1.27
PD	PD66	bin14	MAG1707	472	1869406	18967	4442	3961	67.99	4.26

PD	PD59	bin57	MAG1408	851	1858321	10916	2365	2184	67.94	3.63
PD	PD64	bin50	MAG268	105	1594503	70698	21072	15186	67.88	0
PD	PD58	bin81	MAG896	83	2635996	147890	53336	31759	67.86	9.7
PD	PD8	bin40	MAG355	198	1910502	60152	14740	9649	67.81	0.89
PD	PD46	bin63	MAG243	875	2929710	19965	3647	3348	67.76	5.5
PD	PD81	bin23	MAG2480	395	1845940	30903	5398	4673	67.73	3.45
PD	PD24	bin61	MAG2015	502	1500034	13923	3460	2988	67.68	1.73
PD	PD57	bin66	MAG1283	679	1446687	12985	2290	2131	67.66	4.07
PD	PD48	bin21	MAG3293	225	1694700	25054	8506	7532	67.54	2.02
PD	PD3	bin34	MAG3073	708	1929291	20146	3005	2725	67.53	3.48
PD	PD10	bin49	MAG784	258	1837698	39234	8147	7123	67.24	5.17
PD	PD32	bin17	MAG1920	742	1445597	7047	2112	1948	67.23	1.38
PD	PD62	bin46	MAG2063	491	3614304	54878	11777	7361	66.99	8.77
PD	PD12	bin68	MAG101	677	1211080	8131	1832	1789	66.98	4.81
PD	PD29	bin57	MAG1643	750	1578536	8615	2288	2105	66.97	2.45
PD	PD95	bin7	MAG23	1340	3073123	13613	2489	2293	66.93	2.24
PD	PD85	bin22	MAG15	151	2380952	71183	26483	15768	66.77	1.95
PD	PD58	bin35	MAG1491	834	1982077	9519	2541	2377	66.71	8.97
PD	PD44	bin50	MAG1295	940	1741838	10887	1921	1853	66.63	3.67
PD	PD41	bin41	MAG774	684	1381654	7099	2224	2020	66.59	2.45
PD	PD75	bin16	MAG1619	616	2186223	15208	3929	3549	66.43	3.89
PD	PD62	bin31	MAG1507	768	1922016	17559	2812	2503	66.41	0
PD	PD28	bin22	MAG3479	66	1332898	107139	30973	20195	66.23	2.68
PD	PD94	bin18	MAG1118	76	1556730	128839	42418	20483	66.22	0
PD	PD61	bin29	MAG206	683	1343623	9756	2099	1967	66.21	5.97
PD	PD46	bin22	MAG2661	282	1446924	28497	5902	5131	66.2	0
PD	PD12	bin43	MAG139	418	1461586	26831	3778	3497	66.18	1.48
PD	PD9	bin56	MAG825	1009	1927584	10159	2009	1910	66.04	1.32
PD	PD28	bin52	MAG2699	924	1802976	7046	2110	1951	65.96	1.7
PD	PD21	bin13	MAG833	1815	3118849	7552	1740	1718	65.95	6.22
PD	PD22	bin56	MAG1326	647	1198510	10106	1901	1852	65.9	2.55
PD	PD24	bin6	MAG1540	473	5935525	115209	15452	12549	65.86	9.48
PD	PD82	bin14	MAG1358	439	1863740	54714	6479	4245	65.79	1.75

PD	PD43	bin25	MAG1536	835	1536116	7393	1868	1840	65.65	4.24
PD	PD34	bin46	MAG1554	912	2486192	11890	2812	2726	65.58	6.15
PD	PD98	bin42	MAG1715	615	1279316	6873	2231	2080	65.57	1.66
PD	PD57	bin57	MAG813	1006	1876822	8599	1918	1866	65.52	1.4
PD	PD27	bin8	MAG3081	32	1285363	331109	81448	40168	65.51	0.63
PD	PD17	bin26	MAG29	888	1829949	9076	2176	2061	65.46	3.53
PD	PD44	bin25	MAG2389	229	1689836	54428	11541	7379	65.45	0.97
PD	PD25	bin55	MAG692	604	1347913	12957	2408	2232	65.33	3.04
PD	PD25	bin24	MAG1686	25	1819085	297014	121533	72763	65.32	0
PD	PD77	bin51	MAG3111	348	1674012	28772	5465	4810	65.29	2.09
PD	PD72	bin41	MAG2476	537	1134558	7899	2357	2113	65.28	2.03
PD	PD31	bin56	MAG2474	984	2015784	10305	2220	2049	65.22	0.25
PD	PD64	bin87	MAG2883	515	1518418	13196	3887	2948	65.2	7.1
PD	PD28	bin30	MAG157	76	1346842	136992	30749	17722	65.18	3.57
PD	PD43	bin36	MAG1559	209	1438642	30778	8939	6883	65.18	2.68
PD	PD100	bin8	MAG3438	676	3026144	39399	6630	4477	65.14	3.05
PD	PD39	bin26	MAG325	165	2609399	72977	27816	15815	65	0
PD	PD53	bin4	MAG3466	677	1361401	9418	2126	2011	64.88	5.38
PD	PD35	bin12	MAG188	768	1429773	7539	1933	1862	64.83	1.02
PD	PD87	bin34	MAG676	303	1483549	32273	8356	4896	64.78	3.83
PD	PD76	bin38	MAG1471	504	1045200	8119	2255	2074	64.72	1.84
PD	PD76	bin16	MAG1881	684	1549429	10837	2610	2265	64.71	5.75
PD	PD56	bin17	MAG2127	79	1538427	68063	38786	19474	64.68	8.04
PD	PD34	bin21	MAG493	488	2279339	30125	5403	4671	64.66	1.72
PD	PD29	bin75	MAG3098	126	1621628	74411	18458	12870	64.64	0
PD	PD23	bin10	MAG850	787	2062260	12525	2834	2620	64.61	2.85
PD	PD73	bin53	MAG2713	378	938758	9312	2902	2483	64.59	2.29
PD	PD66	bin22	MAG2812	958	3903877	35880	5645	4075	64.58	0
PD	PD56	bin36	MAG3168	844	1609067	10543	2001	1906	64.57	1.89
PD	PD62	bin40	MAG1421	499	1714187	20536	4577	3435	64.53	0.89
PD	PD36	bin2	MAG2346	721	1363327	7592	2006	1891	64.38	0.34
PD	PD62	bin37	MAG2304	662	1824243	25631	3312	2756	64.36	9.78
PD	PD87	bin72	MAG2694	748	2974743	29871	6210	3977	64.31	6.88

PD	PD94	bin8	MAG159	467	1101977	8739	2626	2360	64.18	0
PD	PD48	bin27	MAG819	1131	1986331	8484	1795	1756	64.13	3.97
PD	PD83	bin3	MAG1991	877	1830247	12117	2226	2087	64.08	0.73
PD	PD67	bin36	MAG3311	760	2000358	10169	2688	2632	63.82	3.34
PD	PD12	bin38	MAG678	210	1554536	36122	9569	7403	63.8	1.23
PD	PD78	bin7	MAG3503	914	1992194	11773	2349	2180	63.64	5.63
PD	PD13	bin13	MAG2156	679	1591731	13310	2593	2344	63.41	4.96
PD	PD32	bin74	MAG2982	662	1532712	15337	2488	2315	63.36	2.71
PD	PD9	bin72	MAG1926	23	1253704	222332	130247	54509	63.29	0
PD	PD37	bin47	MAG3325	798	1510153	7136	1988	1892	63.23	5.33
PD	PD100	bin23	MAG2909	531	2602007	40395	8171	4900	63.16	5.26
PD	PD25	bin64	MAG997	94	1168280	73355	19627	12429	63.13	0.69
PD	PD78	bin41	MAG262	393	1187896	14675	3763	3023	63.13	1.59
PD	PD50	bin25	MAG630	562	1141560	9552	2182	2031	63.1	1.34
PD	PD22	bin22	MAG1218	664	1219359	7256	1905	1836	63.03	4.55
PD	PD10	bin40	MAG3412	1157	1988074	5665	1741	1718	63.02	8.12
PD	PD19	bin30	MAG3253	321	910431	10711	2996	2836	63.02	0.02
PD	PD50	bin10	MAG1531	535	1160068	11674	2357	2168	62.96	0.6
PD	PD60	bin22	MAG2586	151	4768779	158488	59245	31581	62.93	0
PD	PD47	bin22	MAG1505	83	1602494	76660	34839	19307	62.87	0.86
PD	PD11	bin21	MAG1634	1764	3274034	10222	1945	1856	62.83	2.64
PD	PD73	bin15	MAG4	730	1338701	5603	1957	1834	62.8	2.91
PD	PD78	bin12	MAG1032	1572	3219806	11749	2230	2048	62.7	9.35
PD	PD28	bin7	MAG719	528	1605260	13613	3259	3040	62.55	1.58
PD	PD51	bin3	MAG326	599	1050679	9932	1828	1754	62.37	1.95
PD	PD69	bin8	MAG2478	1058	2302366	62609	2247	2176	62.33	2.19
PD	PD81	bin31	MAG568	126	2578722	173761	77836	20466	62.32	0.06
PD	PD16	bin23	MAG1398	456	1341683	13589	3042	2942	62.27	1.4
PD	PD16	bin27	MAG1330	1284	2739834	9673	2304	2134	62.24	1.99
PD	PD48	bin79	MAG2430	419	1908433	26373	5698	4555	62.21	1.82
PD	PD31	bin46	MAG1352	414	2139910	28562	7025	5169	62.07	5.17
PD	PD18	bin37	MAG3367	805	1409676	7952	1800	1751	61.95	2.78
PD	PD44	bin10	MAG1929	782	1432012	7876	1887	1831	61.9	1.09

PD	PD18	bin45	MAG126	358	1609608	20422	5218	4496	61.68	2.01
PD	PD26	bin15	MAG2625	618	2198118	19129	3931	3557	61.61	8.93
PD	PD9	bin71	MAG1152	79	2328993	110505	55522	29481	61.53	0
PD	PD18	bin29	MAG1810	670	2092561	15626	3309	3123	61.48	8.53
PD	PD10	bin23	MAG190	705	1479530	12360	2182	2099	61.45	2.84
PD	PD77	bin56	MAG749	546	2211041	16142	4541	4050	61.43	0.34
PD	PD32	bin20	MAG3157	282	949742	16301	3611	3368	61.4	1.75
PD	PD8	bin65	MAG602	587	1472356	11600	2960	2508	61.4	4.37
PD	PD27	bin4	MAG3255	317	1852293	59820	9534	5843	61.38	2.59
PD	PD71	bin38	MAG338	647	3019532	25247	5216	4667	61.38	2.53
PD	PD20	bin29	MAG2271	553	1704606	14667	3267	3082	61.35	2.22
PD	PD67	bin33	MAG3489	1000	1927053	13746	2055	1927	61.35	5.6
PD	PD19	bin15	MAG1023	806	2065949	12050	2823	2563	61.31	1.92
PD	PD50	bin31	MAG2168	1078	1953249	8687	1854	1812	61.28	2.21
PD	PD66	bin35	MAG8	561	1408964	15124	2804	2512	61.12	1.79
PD	PD9	bin43	MAG1977	367	1366954	19337	3952	3725	61.12	1.41
PD	PD21	bin2	MAG2204	124	681405	21824	7779	5495	61.11	2.56
PD	PD47	bin38	MAG2946	885	4381982	30294	5851	4951	61	6.9
PD	PD48	bin44	MAG2403	808	1475734	7520	1939	1826	60.98	2.84
PD	PD68	bin24	MAG328	831	1327150	5235	1604	1597	60.97	6.03
PD	PD32	bin47	MAG2816	448	1389204	12326	3281	3101	60.95	2.13
PD	PD71	bin25	MAG2037	1530	2680524	7161	1836	1752	60.93	8.78
PD	PD91	bin12	MAG305	428	1545339	15186	4145	3611	60.88	1.79
PD	PD44	bin55	MAG985	215	1138923	16422	5929	5297	60.87	0.34
PD	PD27	bin23	MAG1017	446	1570092	22541	4823	3520	60.8	7.82
PD	PD2	bin52	MAG2097	293	2096785	76108	16230	7156	60.7	6.7
PD	PD46	bin60	MAG236	354	1743511	30206	7163	4925	60.63	1.06
PD	PD28	bin62	MAG1714	664	1466744	12507	2523	2209	60.56	3.73
PD	PD47	bin25	MAG3150	335	1739739	30640	6208	5193	60.53	0
PD	PD77	bin14	MAG1427	1219	3043985	14045	2509	2497	60.46	9.92
PD	PD43	bin58	MAG2448	134	1987269	74848	33187	14830	60.44	1.29
PD	PD70	bin36	MAG836	266	1146808	30896	4835	4311	60.37	1.12
PD	PD25	bin1	MAG3221	283	1613736	19693	6406	5702	60.34	0

PD	PD94	bin57	MAG3021	641	2858770	28894	5157	4460	60.33	4.55
PD	PD20	bin41	MAG2165	1107	1846757	6620	1709	1668	60.32	2.86
PD	PD48	bin20	MAG853	410	1479652	10638	3863	3609	60.25	1.68
PD	PD28	bin31	MAG1655	419	1303833	9902	3295	3112	60.1	1.01
PD	PD73	bin7	MAG1450	730	1712152	20432	2574	2345	60.07	1.58
PD	PD22	bin1	MAG295	543	1032057	7208	2010	1901	60.06	1.15
PD	PD25	bin65	MAG2184	1345	2448434	9330	1862	1820	60.03	2.43
PD	PD12	bin62	MAG3059	1071	2001670	7880	1933	1869	59.91	1.56
PD	PD56	bin38	MAG3299	353	1057288	12715	3149	2995	59.87	0
PD	PD24	bin27	MAG2999	59	2509953	156224	78896	42542	59.83	0
PD	PD76	bin12	MAG1318	561	1505925	11245	2700	2684	59.83	5.9
PD	PD76	bin23	MAG1399	393	1202680	14871	3246	3060	59.78	0.67
PD	PD94	bin43	MAG1014	766	1336351	6752	1803	1745	59.65	5.79
PD	PD50	bin51	MAG3193	597	1120851	5827	1957	1877	59.64	1.23
PD	PD39	bin30	MAG1689	725	1303091	6983	1815	1797	59.59	6
PD	PD68	bin18	MAG1561	263	1992212	39686	9955	7575	59.58	0
PD	PD11	bin17	MAG735	495	1785845	21784	4794	3608	59.53	2.63
PD	PD64	bin41	MAG2614	743	2253054	13203	3190	3032	59.53	2.69
PD	PD82	bin19	MAG1722	100	2277213	186741	63248	22772	59.53	0.67
PD	PD56	bin49	MAG1410	162	1583565	64314	20631	9775	59.48	0
PD	PD40	bin35	MAG3032	777	2328243	17203	3309	2996	59.44	0
PD	PD58	bin15	MAG1435	532	1559936	9387	3050	2932	59.4	1.92
PD	PD1	bin13	MAG3027	1095	1948850	7884	1816	1780	59.39	0.81
PD	PD18	bin41	MAG3508	1044	2144422	11534	2250	2054	59.26	6.06
PD	PD81	bin11	MAG1493	519	1760077	22259	3672	3391	59.26	0.63
PD	PD36	bin22	MAG1239	21	1367163	321720	138510	65103	59.23	0
PD	PD27	bin20	MAG2583	817	2239670	11340	2845	2741	59.04	2.4
PD	PD86	bin10	MAG2091	746	1375215	5292	1941	1843	59.04	2.08
PD	PD99	bin36	MAG210	381	1659978	29729	5043	4357	58.93	1.72
PD	PD43	bin27	MAG3433	440	1303186	12510	3182	2962	58.87	5.17
PD	PD73	bin21	MAG1496	937	1706488	7836	1855	1821	58.79	2.5
PD	PD2	bin39	MAG3382	67	860774	40658	17070	12847	58.56	0
PD	PD22	bin45	MAG863	477	1397224	13832	3097	2929	58.39	5.81

PD	PD77	bin41	MAG631	797	1389512	9068	1705	1743	58.35	1.02
PD	PD6	bin29	MAG1782	619	1181660	9440	2003	1909	58.32	2.01
PD	PD71	bin47	MAG349	1296	2496528	9272	2035	1926	58.27	1.47
PD	PD10	bin27	MAG3144	596	1081686	5771	1913	1815	58.18	3.66
PD	PD81	bin18	MAG2903	525	1037499	8208	2047	1976	58.18	0.99
PD	PD61	bin32	MAG570	411	1791588	25680	5162	4359	58.04	5.36
PD	PD67	bin24	MAG675	597	1083211	6912	1937	1814	57.89	2.55
PD	PD98	bin3	MAG786	124	1020304	38111	12432	8228	57.89	0
PD	PD25	bin78	MAG1623	529	1080675	11735	2304	2043	57.84	6.16
PD	PD51	bin38	MAG3260	395	1198492	14660	3124	3034	57.75	0.34
PD	PD16	bin49	MAG789	739	1329203	8979	1828	1799	57.69	0
PD	PD48	bin12	MAG3445	238	1765790	63050	11710	7419	57.53	0
PD	PD18	bin7	MAG186	1145	2422850	9664	2307	2116	57.51	2.91
PD	PD62	bin5	MAG1110	275	1366687	22775	7227	4970	57.5	1.79
PD	PD95	bin20	MAG2429	215	2244638	76947	19675	10440	57.46	0.55
PD	PD62	bin19	MAG2537	518	1127352	8898	2348	2176	57.35	1.23
PD	PD44	bin12	MAG3274	142	1890344	95311	24441	13312	57.28	2.97
PD	PD32	bin72	MAG44	562	1346152	9241	2565	2395	57.17	2.03
PD	PD37	bin31	MAG2433	250	2158711	47961	13066	8635	57.16	0
PD	PD46	bin81	MAG2021	1399	2247016	10036	1586	1606	57.09	8.86
PD	PD19	bin34	MAG83	750	1355354	11762	1871	1807	57.07	3.08
PD	PD1	bin5	MAG1085	73	3324246	438270	108383	45538	57.05	9.48
PD	PD21	bin10	MAG2269	41	1518881	255502	100142	37046	56.9	0
PD	PD6	bin3	MAG2224	410	1329269	21261	3531	3242	56.86	1.78
PD	PD70	bin8	MAG580	670	1230398	6538	1918	1836	56.83	0.81
PD	PD29	bin9	MAG694	700	1262535	5927	1842	1804	56.79	1.06
PD	PD64	bin7	MAG1769	630	1137842	6736	1875	1806	56.76	1.34
PD	PD35	bin40	MAG2120	449	1489637	20450	3520	3318	56.72	0.63
PD	PD24	bin45	MAG2596	201	1857278	36014	13663	9240	56.61	0
PD	PD2	bin38	MAG2192	981	1644694	9322	1650	1677	56.55	4.33
PD	PD12	bin13	MAG3381	457	1206058	11177	2707	2639	56.51	4.91
PD	PD64	bin46	MAG346	756	1289275	7217	1727	1705	56.41	0.71
PD	PD8	bin66	MAG259	166	1892350	70759	15533	11400	56.41	0

PD	PD85	bin23	MAG1045	455	1741208	11794	4071	3827	56.41	9.82
PD	PD47	bin12	MAG3472	800	2294408	16405	2973	2868	56.36	8.77
PD	PD34	bin64	MAG1479	1042	1632603	5356	1554	1567	56.3	4.15
PD	PD14	bin45	MAG1195	287	1341038	13743	5319	4673	56.27	1.21
PD	PD58	bin54	MAG1566	353	2100133	140611	58271	5949	56.25	9.01
PD	PD10	bin16	MAG1742	942	1550453	5905	1667	1646	56.18	5.62
PD	PD11	bin29	MAG327	455	1492078	15924	3630	3279	56.14	0.88
PD	PD82	bin51	MAG1767	129	1867771	122059	28327	14479	56.14	1.75
PD	PD20	bin43	MAG215	739	1277022	6152	1783	1728	56.11	5.89
PD	PD28	bin33	MAG1243	791	3101815	21440	4628	3921	56.11	8.93
PD	PD6	bin31	MAG1783	888	1558015	6155	1821	1755	56.07	6.75
PD	PD5	bin4	MAG597	310	1323356	29129	4621	4269	55.97	0.67
PD	PD36	bin6	MAG2449	924	1383051	4489	1481	1497	55.95	9.73
PD	PD47	bin16	MAG504	524	1335988	9804	2797	2550	55.95	0
PD	PD34	bin61	MAG2677	615	1359496	15501	2405	2211	55.89	0.73
PD	PD45	bin46	MAG3429	1048	1704798	6445	1631	1627	55.89	3.65
PD	PD34	bin9	MAG212	829	1615170	9174	2092	1948	55.87	1.34
PD	PD50	bin13	MAG2986	255	901966	25902	3880	3537	55.82	0.53
PD	PD40	bin57	MAG1851	1469	2756195	12119	1919	1876	55.81	6.08
PD	PD23	bin11	MAG1366	142	1370363	43956	12730	9650	55.71	2.68
PD	PD95	bin14	MAG901	739	1344345	6312	1888	1819	55.71	2.18
PD	PD11	bin14	MAG3264	57	1378806	108341	42515	24190	55.7	0
PD	PD12	bin67	MAG2280	395	1230771	14636	3396	3116	55.67	2.08
PD	PD26	bin11	MAG1309	412	1283200	10064	3290	3115	55.6	0.16
PD	PD9	bin20	MAG2379	336	1085496	25017	3711	3231	55.55	1.8
PD	PD81	bin34	MAG1808	888	3562822	15909	4378	4012	55.52	1.72
PD	PD3	bin23	MAG1127	503	2418881	27520	5876	4809	55.5	1.72
PD	PD40	bin6	MAG2410	670	1366042	7653	2198	2039	55.5	1.01
PD	PD77	bin18	MAG2651	624	1106111	8336	1799	1773	55.49	1.5
PD	PD64	bin1	MAG1792	83	1881524	165526	40946	22669	55.46	0.95
PD	PD97	bin24	MAG3128	614	1157966	6295	1946	1886	55.44	1.02
PD	PD28	bin51	MAG3507	470	1584193	19015	3645	3371	55.34	0
PD	PD77	bin5	MAG3288	1232	2064460	6366	1689	1676	55.33	2.97

PD	PD99	bin28	MAG668	536	1525886	10203	3210	2847	55.33	0
PD	PD9	bin21	MAG432	724	1381704	7541	1981	1908	55.28	1.79
PD	PD26	bin13	MAG419	731	1347527	6912	1950	1843	55.27	1.02
PD	PD99	bin30	MAG2107	234	2490405	143232	18404	10643	55.26	3.51
PD	PD16	bin39	MAG248	711	1251737	8276	1831	1761	55.23	8.26
PD	PD77	bin3	MAG1873	1209	2226618	10691	1917	1842	55.23	0.38
PD	PD23	bin44	MAG2976	917	1594799	6469	1768	1739	55.19	0.68
PD	PD13	bin29	MAG1026	734	1222002	6427	1708	1665	55.15	0.84
PD	PD1	bin32	MAG2133	977	1729840	7338	1809	1771	55.14	1.68
PD	PD59	bin6	MAG2578	840	1450928	7194	1750	1727	55.14	1.9
PD	PD24	bin42	MAG425	286	846766	9328	3081	2961	55.11	0
PD	PD85	bin36	MAG1773	62	1964168	135259	44933	31680	55.08	0.37
PD	PD92	bin2	MAG185	450	1619728	17688	3899	3599	54.91	1.27
PD	PD31	bin22	MAG2894	1270	1823474	4303	1400	1436	54.86	9.13
PD	PD6	bin12	MAG1256	218	2055022	74682	13771	9427	54.79	1.79
PD	PD48	bin5	MAG1287	502	827202	5748	1676	1648	54.76	1.02
PD	PD50	bin19	MAG1607	752	1158239	4840	1536	1540	54.52	4.5
PD	PD75	bin8	MAG2851	82	1776023	83834	40580	21659	54.39	0
PD	PD87	bin50	MAG3456	247	2708983	200500	27834	10968	54.39	3.51
PD	PD51	bin6	MAG684	21	1160805	181067	78322	55276	54.38	1.77
PD	PD48	bin74	MAG1670	1261	2186567	12860	1787	1734	54.35	9.68
PD	PD77	bin65	MAG1592	569	1131006	8948	2077	1988	54.34	1.65
PD	PD34	bin44	MAG1738	307	775297	7956	2607	2525	54.15	1.28
PD	PD46	bin24	MAG78	844	1673563	6141	2100	1983	54.11	2.96
PD	PD76	bin24	MAG2264	395	1859864	19756	5607	4709	54.09	1.79
PD	PD57	bin35	MAG3312	576	1009992	6620	1819	1753	54.03	1.45
PD	PD21	bin16	MAG2348	935	5391775	48393	7664	5767	53.95	6.9
PD	PD51	bin41	MAG972	573	1335798	16356	2551	2331	53.88	0
PD	PD77	bin52	MAG2640	496	1981016	20410	4673	3994	53.83	3.51
PD	PD16	bin60	MAG1264	603	1134199	6698	1931	1881	53.8	0.68
PD	PD85	bin14	MAG1251	272	3505330	153716	33164	12887	53.79	1.72
PD	PD34	bin63	MAG116	174	1957222	51497	16249	11248	53.76	1.72
PD	PD21	bin28	MAG2128	148	2295039	240951	83333	15507	53.72	1.86

PD	PD2	bin63	MAG3035	191	1058247	32567	6857	5541	53.49	0.67
PD	PD64	bin82	MAG1074	48	1157288	161160	70414	24110	53.46	1.24
PD	PD32	bin43	MAG740	542	946201	7223	1752	1746	53.06	2.41
PD	PD69	bin55	MAG1115	359	1760299	18471	6169	4903	53.04	0
PD	PD87	bin16	MAG1081	474	3183801	75903	11618	6717	53.03	6.33
PD	PD91	bin9	MAG2309	1358	2025507	5866	1482	1492	53.03	9.31
PD	PD51	bin11	MAG2135	551	1048737	8231	1937	1903	53.01	2.38
PD	PD25	bin43	MAG1528	591	1508506	27014	2948	2552	53	1.79
PD	PD41	bin69	MAG2428	889	1512714	5646	1745	1702	52.96	4.41
PD	PD45	bin36	MAG1633	391	1451208	37426	5745	3712	52.83	9.57
PD	PD14	bin18	MAG429	1208	2346386	6882	2043	1942	52.81	6.38
PD	PD51	bin8	MAG130	80	1309879	76250	28460	16373	52.8	0.67
PD	PD85	bin34	MAG1076	194	1440538	89924	17678	7425	52.7	2.27
PD	PD13	bin62	MAG3064	1040	1748759	8612	1696	1681	52.69	2.26
PD	PD24	bin44	MAG1006	49	916295	129140	34551	18700	52.68	1.79
PD	PD41	bin39	MAG1284	94	316560	12911	3847	3368	52.65	0
PD	PD1	bin22	MAG2933	1505	2996845	9022	2103	1991	52.63	0.16
PD	PD75	bin21	MAG2353	92	915381	112386	41635	9950	52.63	1.75
PD	PD82	bin35	MAG1481	327	1371792	18660	5142	4195	52.63	1.75
PD	PD56	bin34	MAG2456	33	1192491	158388	69411	36136	52.61	1.98
PD	PD72	bin13	MAG128	200	1080994	28251	6078	5405	52.6	1.34
PD	PD20	bin48	MAG1228	729	2200102	25043	3261	3018	52.59	1.72
PD	PD17	bin21	MAG2706	460	823343	6200	1832	1790	52.56	1.12
PD	PD12	bin11	MAG2024	662	1182246	7036	1855	1786	52.5	1.77
PD	PD14	bin43	MAG2459	285	1764291	24299	6416	6190	52.48	2.01
PD	PD45	bin16	MAG91	170	2266270	117235	24955	13331	52.44	0.34
PD	PD3	bin32	MAG2327	602	1013813	8253	1689	1684	52.4	0.31
PD	PD82	bin21	MAG1059	913	1541486	5366	1721	1688	52.37	4.75
PD	PD9	bin32	MAG2002	643	1384224	12575	2377	2153	52.31	2.11
PD	PD7	bin31	MAG1550	622	1036694	5854	1687	1667	52.22	3.14
PD	PD67	bin23	MAG266	537	895567	8777	1659	1668	52.19	3.23
PD	PD42	bin19	MAG2149	289	1847983	38860	9417	6394	52.11	1.75
PD	PD91	bin13	MAG1581	590	1096143	8969	1951	1858	52.05	5.59

PD	PD40	bin28	MAG3007	957	1653423	7303	1760	1728	51.67	1.88
PD	PD46	bin58	MAG996	1383	2177564	11210	1540	1575	51.64	5.87
PD	PD48	bin43	MAG3249	801	1339303	6863	1687	1672	51.63	1.15
PD	PD37	bin6	MAG940	991	1619515	6962	1624	1634	51.59	2.48
PD	PD9	bin27	MAG1416	827	1369780	6588	1674	1656	51.53	3.86
PD	PD50	bin34	MAG427	551	986582	5990	1874	1791	51.23	1.34
PD	PD1	bin27	MAG3124	1153	1735901	7904	1459	1506	51.14	5.89
PD	PD58	bin43	MAG1648	720	1134532	4633	1560	1576	51.14	2.57
PD	PD28	bin17	MAG1278	553	1016764	11870	1943	1839	51.13	0.33
PD	PD48	bin83	MAG2124	538	1066452	5853	2175	1982	51.03	0.73
PD	PD45	bin58	MAG2437	723	1245461	7491	1756	1723	50.96	0.96
PD	PD29	bin50	MAG1734	242	1325780	64051	8292	5478	50.88	7.02
PD	PD77	bin9	MAG1916	536	1130454	13880	2245	2109	50.88	0.98
PD	PD81	bin37	MAG1378	419	1393995	17567	4736	3327	50.79	0.08
PD	PD92	bin21	MAG3373	95	3183615	222499	100005	33512	50.76	0
PD	PD71	bin18	MAG2088	276	1348623	14177	5275	4886	50.72	0
PD	PD98	bin27	MAG1368	670	1839675	19237	2791	2746	50.71	2.85
PD	PD56	bin9	MAG882	573	1103914	9245	1999	1927	50.68	2.36
PD	PD100	bin34	MAG577	768	1389509	8329	1864	1809	50.67	1.36
PD	PD98	bin14	MAG2514	1030	1714754	6960	1661	1665	50.62	6.83
PD	PD86	bin36	MAG2001	1023	2051345	22695	2071	2005	50.61	6.35
PD	PD41	bin21	MAG3002	533	1399928	11850	2704	2627	50.59	1.12
PD	PD82	bin4	MAG1678	533	908903	5250	1753	1705	50.59	1.85
PD	PD66	bin32	MAG290	587	1029417	6045	1825	1754	50.58	0.67
PD	PD32	bin48	MAG1286	1057	2524472	9161	2389	2388	50.43	7.02
PD	PD99	bin19	MAG3226	1022	1648038	4255	1663	1613	50.43	9.25
PD	PD37	bin39	MAG1124	311	873393	8105	2898	2808	50.36	0.73
PD	PD83	bin34	MAG447	645	1003928	5780	1542	1556	50.35	2.44
PD	PD72	bin43	MAG1145	1316	1853745	4646	1352	1409	50.29	8.62
PD	PD95	bin36	MAG1806	678	1922902	11571	2913	2836	50.26	3.08
PD	PD98	bin24	MAG1731	638	1264591	10212	2123	1982	50.23	1.12
PD	PD28	bin43	MAG802	1043	1562945	4425	1484	1499	50.08	6.25
PD	PD21	bin32	MAG1028	102	1793614	75611	28726	17584	50.05	0

PD	PD39	bin38	MAG238	570	1063605	8351	1940	1866	50	5.17
PD	PD46	bin39	MAG1334	1179	2060194	7218	1801	1747	50	0.16
Con	Con11	bin5	MAG2372	97	3334544	215340	75503	34377	100	0
Con	Con11	bin26	MAG2604	37	2264702	216014	135084	61208	100	0.38
Con	Con17	bin6	MAG3251	46	2245704	183625	85229	48820	100	0.81
Con	Con19	bin44	MAG957	47	2224942	168066	90232	47339	100	1.61
Con	Con2	bin43	MAG1490	12	1262446	653438	653438	105204	100	0
Con	Con2	bin32	MAG411	23	1911739	375814	160149	83119	100	0
Con	Con20	bin13	MAG1288	54	2272259	224807	83229	42079	100	0.81
Con	Con24	bin51	MAG1071	25	1821277	275901	117723	72851	100	0
Con	Con24	bin56	MAG1956	76	2233486	196992	50298	29388	100	1.61
Con	Con26	bin19	MAG1254	52	2220455	146063	81832	42701	100	8.06
Con	Con27	bin35	MAG3292	31	2145691	235495	166304	69216	100	0.23
Con	Con29	bin7	MAG1463	36	2287445	371488	92148	63540	100	0.47
Con	Con30	bin20	MAG1206	39	2483018	282097	128835	63667	100	0.45
Con	Con32	bin24	MAG2383	19	2193735	380980	198373	115460	100	0
Con	Con38	bin65	MAG181	156	2087600	70174	20387	13382	100	3.23
Con	Con40	bin11	MAG1961	46	2122320	288192	105481	46137	100	0.23
Con	Con40	bin6	MAG2182	93	2143182	136689	49352	23045	100	0.81
Con	Con40	bin36	MAG339	223	2157756	59524	13487	9676	100	0.94
Con	Con44	bin23	MAG2931	41	1990489	202309	64863	48549	100	0
Con	Con44	bin14	MAG3025	43	2052376	238017	138042	47730	100	0
Con	Con48	bin21	MAG1043	40	2192104	352804	231197	54803	100	0.45
Con	Con48	bin41	MAG2446	50	2224061	154597	79416	44481	100	0.81
Con	Con52	bin8	MAG2243	103	2208239	80186	34697	21439	100	0
Con	Con8	bin19	MAG1677	29	1816508	279995	141216	62638	100	0
Con	Con8	bin35	MAG871	116	2683343	165912	38838	23132	100	0
Con	Con21	bin27	MAG3362	63	4493514	294789	136983	71326	99.96	0.46
Con	Con6	bin26	MAG3355	88	4574033	300247	115238	51978	99.96	0.08
Con	Con39	bin13	MAG3383	50	2129309	220083	90030	42586	99.77	0.38
Con	Con34	bin34	MAG3425	56	2163109	234568	102342	38627	99.7	1.4
Con	Con6	bin7	MAG3494	189	2129245	129547	20581	11266	99.68	0.63
Con	Con2	bin61	MAG1420	67	4794779	315452	133489	71564	99.67	0.68

Con	Con19	bin38	MAG2864	65	2801282	343485	143357	43097	99.63	0
Con	Con19	bin30	MAG465	42	1896149	209427	95982	45146	99.62	1.26
Con	Con1	bin1	MAG66	229	2881347	59360	21668	12582	99.55	0.67
Con	Con9	bin19	MAG263	43	2097727	633279	126046	48784	99.55	2.2
Con	Con31	bin13	MAG2859	64	2813638	267435	114221	43963	99.53	0
Con	Con1	bin18	MAG517	89	3338292	135900	60279	37509	99.52	2.82
Con	Con27	bin37	MAG3048	56	2794862	531057	92617	49908	99.52	0
Con	Con35	bin39	MAG1945	59	2649025	261361	66321	44899	99.52	1.22
Con	Con35	bin16	MAG3140	172	3535080	98761	45533	20553	99.52	2.95
Con	Con4	bin26	MAG1538	76	2670301	152305	50540	35136	99.52	0.4
Con	Con32	bin36	MAG1906	76	3017888	284358	59140	39709	99.44	0.24
Con	Con28	bin9	MAG2609	84	2614485	125219	51066	31125	99.42	0
Con	Con22	bin27	MAG1910	50	2437433	217736	95130	48749	99.37	0
Con	Con30	bin28	MAG3254	96	2548533	97411	39114	26547	99.37	0
Con	Con31	bin29	MAG3339	420	3994867	92488	17848	9512	99.37	0.16
Con	Con44	bin18	MAG2161	73	3059825	231684	65221	41915	99.37	0
Con	Con52	bin10	MAG2567	122	2687190	132999	36722	22026	99.37	0
Con	Con1	bin17	MAG2132	418	3974867	70852	20778	9509	99.33	4.7
Con	Con27	bin22	MAG3173	42	1981882	196472	118141	47188	99.33	1.01
Con	Con28	bin15	MAG2889	77	2870063	156940	55153	37274	99.33	2.35
Con	Con31	bin42	MAG2623	57	2198461	602197	163923	38569	99.33	1.01
Con	Con38	bin34	MAG2685	41	2742579	301636	131535	66892	99.33	0
Con	Con38	bin45	MAG652	208	3142431	104600	30158	15108	99.33	0.67
Con	Con45	bin35	MAG2167	38	2025667	195564	115967	53307	99.33	0.69
Con	Con45	bin20	MAG2978	118	2586728	89582	33324	21921	99.33	2.68
Con	Con48	bin24	MAG909	232	3292158	134864	37014	14190	99.33	0.84
Con	Con6	bin18	MAG575	38	1925632	257465	113772	50675	99.33	1.01
Con	Con22	bin17	MAG640	80	2320984	143996	62402	29012	99.32	0
Con	Con27	bin53	MAG177	178	3084961	97014	30095	17331	99.3	1.17
Con	Con4	bin59	MAG2642	155	2881779	118963	30934	18592	99.3	1.17
Con	Con39	bin12	MAG1040	110	2685689	107851	40624	24415	99.29	0.59
Con	Con12	bin22	MAG998	69	2865669	165926	67875	41531	99.24	0
Con	Con48	bin38	MAG2776	137	2390587	227960	50193	17450	99.24	1.14

Con	Con4	bin56	MAG3210	72	4042004	367403	90230	56139	99.22	0
Con	Con6	bin22	MAG540	62	1803810	250596	127507	29094	99.22	0.82
Con	Con6	bin9	MAG689	56	2862057	229123	71342	51108	99.21	1.21
Con	Con26	bin21	MAG2969	166	3048075	139845	36241	18362	99.19	2.69
Con	Con30	bin1	MAG69	62	2217171	195639	79771	35761	99.19	0.89
Con	Con40	bin37	MAG2275	19	1669717	383580	176383	87880	99.19	0.44
Con	Con49	bin31	MAG2823	40	2070197	201346	98066	51755	99.18	0
Con	Con52	bin12	MAG3130	71	1967019	144762	45747	27704	99.18	0
Con	Con6	bin33	MAG468	93	2156570	125898	51697	23189	99.18	0
Con	Con12	bin33	MAG1788	54	3004567	264032	114572	55640	99.13	0.16
Con	Con6	bin5	MAG2312	124	3067523	172690	51799	24738	99.12	0
Con	Con48	bin34	MAG2790	58	2494240	242919	61303	43004	99.11	0.72
Con	Con22	bin21	MAG3057	87	2777749	180451	52839	31928	99.06	0
Con	Con24	bin42	MAG431	111	2972137	180034	47416	26776	99.06	0
Con	Con27	bin49	MAG3239	101	2659861	254071	47924	26335	99.06	0
Con	Con40	bin13	MAG1521	87	2437251	198735	50602	28014	99.06	0.47
Con	Con8	bin24	MAG1117	102	2634934	254824	44814	25833	99.06	0
Con	Con19	bin15	MAG3005	186	2580717	83704	20058	13875	99.04	0.48
Con	Con31	bin35	MAG1097	192	3743702	349245	51976	19498	99.03	0.36
Con	Con52	bin17	MAG1680	100	2142928	111960	48974	21429	99.03	1.58
Con	Con20	bin20	MAG2802	56	2152894	139133	67903	38445	99	1.61
Con	Con12	bin49	MAG1511	90	2713340	131991	49102	30148	98.99	2.01
Con	Con34	bin20	MAG163	227	2601273	75423	19357	11459	98.97	0.02
Con	Con17	bin50	MAG2257	61	2988951	193396	75991	48999	98.95	0.63
Con	Con32	bin38	MAG2019	61	2911163	274038	68822	47724	98.95	0.63
Con	Con34	bin31	MAG3305	109	2435576	133927	36472	22345	98.95	0.64
Con	Con8	bin15	MAG3287	47	2977522	367087	84811	63352	98.95	0.79
Con	Con22	bin25	MAG2584	39	2916424	337182	152002	74780	98.83	1.46
Con	Con25	bin32	MAG2119	45	1904700	330395	76017	42327	98.83	0
Con	Con29	bin20	MAG677	134	2249247	142856	53425	16785	98.83	2.76
Con	Con35	bin4	MAG2126	272	3849873	198641	56866	14154	98.79	0.86
Con	Con46	bin4	MAG515	110	2097724	211810	88231	19070	98.79	0.68
Con	Con12	bin26	MAG2169	266	5547096	105227	30337	20854	98.77	0.63

Con	Con28	bin27	MAG1593	257	4431857	110928	25077	17245	98.75	0.49
Con	Con11	bin3	MAG1839	222	3429270	124175	31944	15447	98.73	1.58
Con	Con22	bin10	MAG1279	205	4787950	171217	67546	23356	98.73	1.11
Con	Con22	bin12	MAG211	43	2778876	253575	97373	64625	98.73	0
Con	Con26	bin16	MAG955	141	2225472	85846	28630	15783	98.69	1.21
Con	Con24	bin54	MAG506	97	2407955	100472	37464	24824	98.68	2.95
Con	Con1	bin29	MAG3246	56	2247094	324373	83752	40127	98.66	0.34
Con	Con1	bin47	MAG817	57	1991416	232961	87054	34937	98.66	0
Con	Con10	bin34	MAG440	36	1901538	447150	149068	52821	98.66	0
Con	Con24	bin7	MAG2300	83	2345390	186468	52330	28258	98.66	0.67
Con	Con24	bin55	MAG2331	68	2627896	136351	57938	38646	98.66	0
Con	Con34	bin17	MAG1969	139	2115003	172883	31551	15216	98.66	0.34
Con	Con35	bin9	MAG1814	28	2638598	313827	131359	94236	98.66	0
Con	Con38	bin54	MAG809	45	2289681	206120	91390	50882	98.66	0.34
Con	Con39	bin48	MAG140	23	1994868	272538	141077	86733	98.66	1.01
Con	Con40	bin34	MAG170	85	2504275	120177	48815	29462	98.66	0.67
Con	Con44	bin45	MAG1754	133	2347788	95720	48045	17653	98.66	0.67
Con	Con45	bin18	MAG2199	32	2644627	450792	124910	82645	98.66	0
Con	Con48	bin27	MAG893	41	2063237	198328	112613	50323	98.66	1.01
Con	Con50	bin41	MAG2110	126	2324907	196069	30513	18452	98.66	0.67
Con	Con28	bin12	MAG590	315	2749450	44981	12710	8728	98.64	0.68
Con	Con30	bin22	MAG168	49	2140999	256086	84520	43694	98.64	0
Con	Con39	bin11	MAG2697	389	2362679	37274	8101	6074	98.64	0.93
Con	Con40	bin18	MAG2454	314	2659838	40112	12795	8471	98.64	1.36
Con	Con52	bin21	MAG152	327	2592107	48707	11110	7927	98.64	1.02
Con	Con1	bin64	MAG1520	74	2592634	225274	86152	35036	98.63	0
Con	Con5	bin43	MAG2266	110	2635097	156065	42869	23955	98.63	0
Con	Con39	bin51	MAG1308	156	2188471	85802	25880	14029	98.6	0.23
Con	Con49	bin23	MAG2850	154	2913468	98289	31767	18919	98.6	1.17
Con	Con28	bin26	MAG2180	275	4045047	110940	28101	14709	98.56	6.94
Con	Con28	bin6	MAG274	101	2948848	169752	55220	29197	98.54	0
Con	Con9	bin2	MAG2095	82	2735373	214352	107897	33358	98.5	0.75
Con	Con3	bin3	MAG27	149	2219960	127545	35220	14899	98.48	3.31

Con	Con5	bin9	MAG3103	100	4590386	188800	86447	45904	98.47	0
Con	Con51	bin5	MAG2624	273	3525600	220455	39424	12914	98.45	0.72
Con	Con32	bin35	MAG2392	104	2244260	105203	35909	21579	98.43	0
Con	Con32	bin4	MAG1608	93	2747149	93815	48190	29539	98.42	0.95
Con	Con27	bin33	MAG2278	102	1979882	88549	28094	19411	98.39	1.48
Con	Con54	bin5	MAG3335	28	1751978	191592	83157	62571	98.39	0
Con	Con22	bin15	MAG285	321	3398019	70065	18514	10586	98.32	1.34
Con	Con34	bin7	MAG2783	97	1899978	160582	31180	19587	98.32	0.67
Con	Con40	bin2	MAG1533	49	1929300	164743	68724	39373	98.32	1.01
Con	Con24	bin28	MAG3359	78	2919758	210073	92621	37433	98.31	1.05
Con	Con38	bin8	MAG3401	138	3546791	112343	45146	25701	98.31	1.86
Con	Con6	bin2	MAG2993	66	3291019	221104	82159	49864	98.31	0.34
Con	Con34	bin29	MAG1196	207	1842665	49318	12315	8902	98.25	1.35
Con	Con6	bin14	MAG3237	132	2821032	103220	37430	21371	98.25	0.58
Con	Con8	bin30	MAG1786	110	2310374	249895	38579	21003	98.14	0.62
Con	Con29	bin3	MAG2291	26	2205845	531405	158908	84840	98.12	0
Con	Con31	bin32	MAG3434	95	2292619	115652	51824	24133	98.12	0.62
Con	Con4	bin14	MAG2811	220	2643200	165501	21923	12015	98.11	0
Con	Con10	bin47	MAG1370	167	3011544	118740	31682	18033	98.07	1.57
Con	Con22	bin29	MAG1960	118	3246022	101984	40492	27509	98.07	1.52
Con	Con45	bin41	MAG1582	70	2769573	294550	86768	39565	98.07	1.76
Con	Con11	bin19	MAG2258	156	3121649	147187	36647	20011	97.99	5.85
Con	Con24	bin8	MAG3151	52	2008116	146516	60705	38618	97.99	0.17
Con	Con27	bin46	MAG1556	64	2136888	107725	52206	33389	97.99	0
Con	Con3	bin41	MAG105	102	2205364	104623	34357	21621	97.99	1.34
Con	Con3	bin12	MAG1717	43	2604438	503689	132474	60568	97.99	0
Con	Con32	bin46	MAG3316	78	2950651	253410	80403	37829	97.99	1.34
Con	Con45	bin54	MAG3017	98	2754086	109171	48643	28103	97.99	0.67
Con	Con30	bin45	MAG396	125	2422582	114270	37658	19381	97.97	0.67
Con	Con2	bin7	MAG314	69	2684187	369793	92601	38901	97.96	0
Con	Con25	bin35	MAG2387	40	2720946	378387	116102	68024	97.96	0
Con	Con31	bin11	MAG2842	43	2530417	201739	74867	58847	97.96	0
Con	Con45	bin7	MAG1936	47	2757459	370464	111352	58669	97.96	0

Con	Con53	bin1	MAG32	42	2671938	404759	96449	63618	97.96	0
Con	Con54	bin32	MAG503	415	2246916	40136	8566	5414	97.91	6.29
Con	Con40	bin44	MAG1513	176	2251579	112768	25617	12793	97.84	1.38
Con	Con35	bin12	MAG1369	133	3074994	221371	43690	23120	97.8	1.45
Con	Con31	bin30	MAG2543	324	1948461	29863	8740	6014	97.77	3.83
Con	Con39	bin33	MAG1149	94	4232566	425721	147713	45027	97.77	0.19
Con	Con53	bin10	MAG3391	846	3065177	29525	4730	3623	97.75	2.36
Con	Con9	bin5	MAG2752	53	2232727	195296	99881	42127	97.75	0.23
Con	Con21	bin16	MAG2739	165	2303264	77423	25942	13959	97.71	1.11
Con	Con10	bin9	MAG2653	108	3098131	375427	83904	28686	97.68	0.63
Con	Con6	bin31	MAG3099	224	2177334	60481	15362	9720	97.65	2.27
Con	Con20	bin28	MAG1271	191	3787180	230724	34821	19828	97.63	0.59
Con	Con49	bin61	MAG302	57	2423840	166158	67091	42524	97.6	1.2
Con	Con53	bin5	MAG1621	100	5163637	354168	90550	51636	97.6	0.45
Con	Con6	bin17	MAG3481	69	2313288	152355	53126	33526	97.6	0
Con	Con1	bin32	MAG2949	78	3867654	257877	88857	49585	97.58	0
Con	Con45	bin27	MAG119	145	3237098	129410	51267	22325	97.58	0.16
Con	Con49	bin54	MAG1310	173	2050559	61898	16691	11853	97.58	1.21
Con	Con17	bin64	MAG2213	30	2040550	332366	144665	68018	97.54	0.67
Con	Con6	bin35	MAG2397	281	2410758	44146	11834	8579	97.51	0.68
Con	Con2	bin68	MAG1923	132	2826628	105291	30769	21414	97.49	0.58
Con	Con20	bin27	MAG589	57	2072519	179749	61884	36360	97.47	1.04
Con	Con26	bin17	MAG2695	146	3159615	106112	30314	21641	97.47	0
Con	Con5	bin2	MAG1305	82	3612034	170303	70188	44049	97.4	0
Con	Con17	bin67	MAG1498	17	1893900	317113	186204	111406	97.32	0.67
Con	Con21	bin39	MAG1465	11	1978711	739673	494852	179883	97.32	0
Con	Con27	bin48	MAG3167	17	1954643	651783	414035	114979	97.32	0.67
Con	Con3	bin67	MAG226	47	2603655	272678	76212	55397	97.32	0
Con	Con38	bin30	MAG13	25	1948220	276818	112050	77929	97.32	0
Con	Con40	bin24	MAG2796	197	2271877	55810	17382	11532	97.32	1.01
Con	Con40	bin17	MAG3129	13	1846244	387350	210537	142019	97.32	0
Con	Con49	bin18	MAG2522	49	2553849	309104	84119	52119	97.32	0
Con	Con8	bin5	MAG3317	70	2740204	140725	61328	39146	97.32	0

Con	Con17	bin45	MAG2701	311	2476883	45322	11597	7964	97.28	0.68
Con	Con46	bin13	MAG2663	295	4449094	84956	23451	15082	97.26	0.73
Con	Con10	bin20	MAG3181	179	2997842	117254	30355	16748	97.2	1.17
Con	Con51	bin36	MAG1248	127	2076217	104673	37443	16348	97.2	2.87
Con	Con17	bin22	MAG475	151	2303468	89971	35857	15255	97.19	0.29
Con	Con53	bin23	MAG2104	86	4526280	242903	102787	52631	97.12	0.51
Con	Con51	bin31	MAG2198	271	4553037	98910	33510	16801	97.09	0.97
Con	Con31	bin15	MAG203	90	2571425	118678	48131	28571	97.08	0.58
Con	Con8	bin17	MAG2072	190	2132731	123430	25219	11225	97	0.73
Con	Con32	bin5	MAG1007	189	6532842	179163	53348	34565	96.98	9.54
Con	Con3	bin68	MAG3036	79	2990769	221976	60939	37858	96.96	1.18
Con	Con38	bin63	MAG409	72	2661898	322835	64131	36971	96.86	1.07
Con	Con39	bin42	MAG1933	89	2973951	148842	77373	33415	96.86	0
Con	Con6	bin20	MAG2782	90	3157504	135926	52751	35083	96.86	0.85
Con	Con31	bin33	MAG269	111	6290393	241672	106987	56670	96.84	1.92
Con	Con25	bin14	MAG2801	389	1986553	41423	7061	5107	96.81	3.21
Con	Con18	bin12	MAG2416	40	2688809	374030	158395	67220	96.77	0.87
Con	Con3	bin65	MAG142	97	2090093	125817	34198	21547	96.77	0.81
Con	Con20	bin5	MAG2007	350	2460165	55540	9838	7029	96.76	1.34
Con	Con19	bin11	MAG2329	43	1677609	105151	70076	39014	96.74	0.54
Con	Con1	bin4	MAG1151	96	2640277	174927	58780	27503	96.64	1.01
Con	Con3	bin25	MAG3422	19	1910586	271559	158257	100557	96.64	0
Con	Con35	bin45	MAG3031	93	2011194	147457	38461	21626	96.64	0
Con	Con11	bin1	MAG3403	507	2860970	51592	7655	5643	96.63	2.25
Con	Con51	bin49	MAG2298	78	2163561	266367	88460	27738	96.63	1.44
Con	Con21	bin14	MAG2434	142	2096321	78007	21322	14763	96.6	0
Con	Con25	bin37	MAG987	142	3284259	145844	54327	23129	96.6	0
Con	Con49	bin42	MAG1957	134	2126268	68508	28246	15868	96.6	0
Con	Con27	bin44	MAG2303	217	3146174	83253	25995	14498	96.52	0.65
Con	Con4	bin27	MAG3014	158	2771661	83655	25553	17542	96.52	0.63
Con	Con26	bin5	MAG2497	80	2366551	167867	41951	29582	96.5	0
Con	Con39	bin26	MAG3020	178	2858639	98553	20584	16060	96.5	1.17
Con	Con2	bin19	MAG3499	319	2407707	74276	12340	7548	96.46	3.36

Con	Con48	bin14	MAG1823	304	2001728	34607	11425	6585	96.45	3.59	
Con	Con17	bin46	MAG3148	156	2064117	93068	22369	13232	96.36	1.18	
Con	Con32	bin10	MAG2843	68	2797535	161090	57515	41140	96.36	0.19	
Con	Con6	bin39	MAG3424	215	2449334	223287	29404	11392	96.36	0.23	
Con	Con21	bin41	MAG2361	458	2233593	58921	6342	4877	96.31	2.04	
Con	Con8	bin22	MAG85	100	6021021	427083	130547	60210	96.28	0.37	
Con	Con35	bin36	MAG711	301	2334688	38409	10674	7756	96.26	1.02	
Con	Con35	bin5	MAG2279	119	2311407	91063	30823	19424	96.2	1.34	
Con	Con49	bin20	MAG1480	38	2110898	184485	105456	55550	96.15	0	
Con	Con27	bin14	MAG3231	123	3203111	137444	47946	26042	96.14	0.85	
Con	Con2	bin1	MAG1948	158	2646121	118310	31090	16748	96.08	0.99	
Con	Con6	bin27	MAG3453	115	2162892	141117	42786	18808	96.07	0.73	
Con	Con49	bin58	MAG2302	261	1905068	60664	13570	7299	96.05	1.93	
Con	Con26	bin15	MAG3156	48	2116294	265441	126438	44089	95.97	0.67	
Con	Con32	bin40	MAG1847	64	2503542	111894	58429	39118	95.97	0	
Con	Con35	bin47	MAG2244	133	2582943	94521	29935	19421	95.97	0	
Con	Con38	bin18	MAG221	112	1956489	182971	29851	17469	95.97	0	
Con	Con4	bin52	MAG1950	340	2489494	53529	10986	7322	95.97	1.85	
Con	Con4	bin57	MAG542	280	2432834	44669	13061	8689	95.97	0.17	
Con	Con45	bin17	MAG141	53	2371537	171234	68441	44746	95.97	0	
Con	Con48	bin22	MAG463	13	1905645	479682	206691	146588	95.97	0	
Con	Con48	bin15	MAG635	105	2526418	118761	40055	24061	95.97	1.34	
Con	Con9	bin7	MAG2131	48	2102163	124671	81757	43795	95.97	0.81	
Con	Con35	bin41	MAG672	106	2058071	155695	33188	19416	95.93	0.23	
Con	Con10	bin19	MAG2233	125	3983917	225497	106860	31871	95.91	0	
Con	Con49	bin77	MAG1138	158	2644939	97336	29181	16740	95.89	3.3	
Con	Con40	bin62	MAG968	77	2727183	171823	64211	35418	95.88	1.86	
Con	Con18	bin6	MAG1329	207	4233804	164417	64857	20453	95.86	3.49	
Con	Con18	bin23	MAG3176	201	3693343	151712	30291	18375	95.85	0.08	
Con	Con1	bin39	MAG2282	98	2653247	172955	56012	27074	95.79	1.42	
Con	Con5	bin41	MAG1898	241	2986864	81694	28966	12394	95.76	9.75	
Con	Con8	bin25	MAG1136	68	2555775	261526	61732	37585	95.75	2.58	
Con	Con32	bin26	MAG2073	105	3525392	149538	58795	33575	95.72	0	

Con	Con18	bin7	MAG3192	480	2517844	58858	8685	5246	95.71	5.85
Con	Con18	bin24	MAG1486	98	1969395	131179	37944	20096	95.7	0
Con	Con3	bin38	MAG1915	52	2526499	140425	70082	48587	95.65	1.22
Con	Con12	bin40	MAG1879	60	2613243	179454	63108	43554	95.64	0
Con	Con17	bin44	MAG3418	96	2236487	101777	37352	23297	95.64	1.34
Con	Con1	bin37	MAG3069	85	2843482	190768	82401	33453	95.63	3.02
Con	Con2	bin31	MAG1306	96	2079592	153450	30955	21662	95.57	2.69
Con	Con35	bin22	MAG858	118	2451870	165075	36851	20779	95.57	0
Con	Con38	bin56	MAG2357	61	2587022	289706	73825	42410	95.57	1.69
Con	Con5	bin24	MAG2159	266	1598751	29136	7570	6010	95.48	0.57
Con	Con1	bin45	MAG2483	138	2577491	116253	28718	18677	95.44	2.14
Con	Con4	bin29	MAG3282	131	2583788	141702	34281	19724	95.39	0
Con	Con4	bin22	MAG2261	180	2293328	70145	20015	12741	95.36	2.01
Con	Con10	bin5	MAG2755	165	1967388	67974	18538	11924	95.3	1.68
Con	Con3	bin5	MAG1617	108	2335619	85674	32154	21626	95.29	1.37
Con	Con28	bin7	MAG282	196	3129887	89659	31439	15969	95.25	0.34
Con	Con46	bin1	MAG233	102	2443290	151127	50824	23954	95.24	0
Con	Con50	bin16	MAG673	90	2672722	150949	55333	29697	95.24	0
Con	Con54	bin11	MAG965	65	2354266	212816	61560	36219	95.24	0
Con	Con51	bin9	MAG545	436	2001129	21243	5896	4590	95.22	1.65
Con	Con18	bin1	MAG1217	1093	3510952	15954	4111	3212	95.17	7.1
Con	Con31	bin24	MAG2529	117	2132337	111314	32247	18225	95.16	0.96
Con	Con50	bin13	MAG2856	299	2814538	58389	14817	9413	95.05	2.89
Con	Con39	bin40	MAG553	195	2657143	75435	18902	13626	95.04	1.37
Con	Con10	bin25	MAG1362	95	2062199	103605	45841	21707	95	0.61
Con	Con17	bin1	MAG2040	455	2840970	59229	8727	6244	94.99	1.63
Con	Con19	bin22	MAG3353	262	2116585	71032	11259	8079	94.97	0
Con	Con48	bin19	MAG2554	159	2520615	196618	38041	15853	94.97	0.89
Con	Con49	bin55	MAG47	112	2523155	186940	41798	22528	94.97	0
Con	Con51	bin17	MAG1255	97	2974333	228158	61756	30663	94.93	1.92
Con	Con53	bin24	MAG442	295	6012651	151945	30482	20382	94.92	1.58
Con	Con19	bin41	MAG1866	402	2816448	67311	14704	7006	94.85	1.96
Con	Con40	bin20	MAG311	163	2045799	143358	34941	12551	94.83	0.34

Con	Con52	bin50	MAG2177	238	3275429	253047	29073	13762	94.81	0.56
Con	Con11	bin9	MAG1658	240	2750051	57784	16252	11459	94.78	1.42
Con	Con38	bin26	MAG1863	240	2182876	60451	18270	9095	94.76	0.4
Con	Con51	bin7	MAG3476	129	2919395	108282	40069	22631	94.75	0.19
Con	Con19	bin29	MAG2979	302	2199927	47368	9694	7285	94.63	2.24
Con	Con22	bin31	MAG3415	98	2554147	118126	41743	26063	94.63	1.34
Con	Con31	bin22	MAG2779	440	4375342	57191	13178	9944	94.63	2.27
Con	Con34	bin36	MAG1107	200	2146199	57444	16831	10731	94.63	0.89
Con	Con1	bin30	MAG1831	136	2785394	155709	56878	20481	94.58	2.78
Con	Con31	bin17	MAG1010	103	2296614	143370	52926	22297	94.56	0
Con	Con39	bin4	MAG1730	100	2253653	174522	33753	22537	94.56	0.06
Con	Con51	bin48	MAG382	198	2706945	74567	19520	13671	94.56	1.59
Con	Con50	bin42	MAG745	64	1968786	142238	60726	30762	94.5	0.34
Con	Con54	bin16	MAG2968	549	3482492	45647	9769	6343	94.41	6.04
Con	Con30	bin9	MAG2878	261	3043931	59912	19377	11663	94.35	0.81
Con	Con18	bin34	MAG385	166	3751744	151864	31835	22601	94.34	0
Con	Con25	bin5	MAG3393	330	1975769	38201	8229	5987	94.26	3.72
Con	Con3	bin49	MAG3051	44	2212559	241030	106711	50285	94.15	0
Con	Con35	bin46	MAG2290	270	3223608	83564	19694	11939	94.15	0
Con	Con49	bin50	MAG1472	56	2418475	285344	95350	43187	94.15	0
Con	Con5	bin47	MAG2875	232	2607575	68010	16385	11240	94.11	1.2
Con	Con18	bin3	MAG3214	589	2954302	40308	6420	5016	94.09	0.79
Con	Con38	bin6	MAG3470	169	2127258	91860	22778	12587	94.09	5.08
Con	Con32	bin8	MAG1744	228	2958890	88632	18564	12978	93.95	0.8
Con	Con5	bin11	MAG2535	311	2520579	47708	11925	8105	93.95	1.42
Con	Con11	bin23	MAG424	121	2958796	122120	51301	24453	93.93	0.79
Con	Con27	bin12	MAG1215	164	2062370	101050	21483	12575	93.87	0.68
Con	Con51	bin62	MAG1972	204	2292878	125401	21531	11240	93.86	2.21
Con	Con40	bin14	MAG1902	745	3441144	37070	6059	4619	93.85	3.25
Con	Con2	bin27	MAG598	32	1104933	215432	120366	34529	93.82	1.12
Con	Con3	bin55	MAG743	158	1720168	57662	18023	10887	93.74	0
Con	Con38	bin3	MAG3405	89	1954738	138240	38778	21963	93.72	0
Con	Con50	bin46	MAG758	100	2434893	154855	42823	24349	93.67	1.74

Con	Con30	bin19	MAG451	79	6526087	403328	149657	82609	93.65	1.27
Con	Con2	bin46	MAG57	481	3897961	108012	20412	8104	93.62	2.67
Con	Con26	bin32	MAG1646	45	1913228	199502	85022	42516	93.55	0
Con	Con40	bin22	MAG2239	35	2681697	195746	129060	76620	93.55	0.54
Con	Con2	bin29	MAG1123	14	1121825	313952	152098	80130	93.54	0
Con	Con24	bin19	MAG1276	81	2796399	131658	64617	34523	93.49	0.13
Con	Con9	bin24	MAG2569	638	3976335	24941	8275	6233	93.48	1.46
Con	Con2	bin34	MAG2829	655	2313430	19800	4275	3532	93.43	0.89
Con	Con17	bin66	MAG2923	43	2072704	367541	103123	48202	93.41	0
Con	Con35	bin24	MAG1226	695	2484684	28789	4770	3575	93.4	2.22
Con	Con8	bin3	MAG2017	150	2265192	90149	32013	15101	93.37	0
Con	Con1	bin57	MAG1943	109	2019661	136271	31975	18529	93.29	0.89
Con	Con10	bin13	MAG2963	18	1254488	301195	148205	69694	93.26	1.28
Con	Con21	bin4	MAG1209	163	2611713	105408	30573	16023	93.26	0
Con	Con35	bin23	MAG1575	10	1278935	544587	482618	127894	93.26	1.28
Con	Con30	bin13	MAG1104	291	2149245	46178	10578	7386	93.25	0.37
Con	Con4	bin58	MAG3258	603	2151040	14831	4255	3567	93.25	1.31
Con	Con17	bin34	MAG145	110	2004054	87532	35935	18219	93.2	0.68
Con	Con9	bin9	MAG1807	514	2440394	34321	5858	4748	93.2	0.84
Con	Con51	bin19	MAG2857	257	3730370	94643	21858	14515	93.19	0.51
Con	Con19	bin25	MAG1458	309	2210356	36239	9849	7153	93.03	1.87
Con	Con20	bin25	MAG261	36	3752261	365851	152660	104229	92.98	0
Con	Con49	bin46	MAG3294	351	3849596	63803	16117	10968	92.94	1.49
Con	Con30	bin32	MAG2558	231	2127659	53314	13690	9211	92.9	0.73
Con	Con4	bin61	MAG609	416	2340152	31125	7361	5625	92.75	0.22
Con	Con11	bin18	MAG3432	45	3071756	311532	108625	68261	92.74	0
Con	Con18	bin21	MAG2981	166	2058386	56453	18649	12400	92.73	0.67
Con	Con3	bin74	MAG438	20	1303606	270185	137325	65180	92.7	3.37
Con	Con39	bin41	MAG53	118	2979753	113270	44600	25252	92.68	1.33
Con	Con34	bin51	MAG161	480	1743051	29441	4929	3631	92.67	0.89
Con	Con22	bin32	MAG1129	481	3085708	41209	8376	6415	92.64	1.55
Con	Con2	bin14	MAG2897	593	2419153	28925	5184	4080	92.58	2.35
Con	Con34	bin55	MAG2920	422	2354392	53674	7976	5579	92.56	2.91

Con	Con29	bin15	MAG1080	150	3849802	126948	45099	25665	92.51	0.48
Con	Con27	bin18	MAG1766	97	2164103	124760	34847	22310	92.45	0.67
Con	Con31	bin36	MAG3113	489	2837940	38845	8976	5804	92.45	2.14
Con	Con39	bin3	MAG2460	49	1308897	90017	37126	26712	92.42	4.49
Con	Con9	bin18	MAG1446	30	1260067	126932	73166	42002	92.42	4.49
Con	Con17	bin56	MAG3026	157	2147351	75999	21644	13677	92.17	1.36
Con	Con50	bin21	MAG2427	357	3001412	51846	14665	8407	92.17	0
Con	Con34	bin57	MAG1508	406	1995878	24497	5998	4916	92.01	2.3
Con	Con28	bin32	MAG3086	382	3480815	72249	13134	9112	91.99	0.63
Con	Con49	bin71	MAG805	252	2146228	45227	12117	8517	91.97	0.35
Con	Con4	bin44	MAG2447	53	2087292	156810	62129	39383	91.95	0
Con	Con8	bin23	MAG799	95	2339753	224924	63830	24629	91.95	0
Con	Con4	bin48	MAG2594	386	4162952	61272	14843	10785	91.89	1.92
Con	Con27	bin1	MAG1072	67	2090344	109503	60168	31199	91.84	0.68
Con	Con48	bin25	MAG88	80	2098062	147823	38110	26226	91.84	0
Con	Con45	bin57	MAG705	93	2848362	102867	49263	30628	91.79	1.45
Con	Con25	bin3	MAG109	42	2849309	260787	81375	67841	91.77	0
Con	Con11	bin31	MAG1502	316	5139567	119104	23085	16264	91.73	1.01
Con	Con39	bin47	MAG1319	201	2182411	101968	20996	10858	91.73	9.86
Con	Con48	bin23	MAG2396	488	2655258	91824	9865	5441	91.64	5.93
Con	Con4	bin25	MAG852	303	2395111	36046	10865	7905	91.59	0.79
Con	Con38	bin2	MAG1307	44	1177304	125295	79587	26757	91.57	1.12
Con	Con31	bin41	MAG1804	105	3096938	203122	49775	29495	91.51	0.94
Con	Con3	bin62	MAG611	231	2786066	118303	21119	12061	91.45	0.19
Con	Con28	bin4	MAG1996	392	4258574	71208	14682	10864	91.4	0.12
Con	Con5	bin29	MAG3152	167	2089716	60680	26789	12513	91.38	0.34
Con	Con39	bin9	MAG857	420	2490591	44324	8143	5930	91.36	1.9
Con	Con38	bin22	MAG1345	50	1197003	126711	46379	23940	91.29	4.49
Con	Con49	bin28	MAG2373	52	1855838	216103	116198	35689	91.28	0.67
Con	Con49	bin1	MAG2043	520	1706589	45145	3971	3282	91.26	5.24
Con	Con24	bin49	MAG497	318	2554343	41807	10736	8033	91.25	1.53
Con	Con3	bin36	MAG2848	129	2207445	116079	28974	17112	91.16	0.34
Con	Con32	bin2	MAG2921	449	1773103	18842	5285	3949	91.1	1.63

Con	Con27	bin21	MAG2036	396	2501046	39391	10865	6316	91.03	1.71
Con	Con12	bin8	MAG2686	43	2049516	233927	85250	47663	91	2.1
Con	Con4	bin9	MAG257	215	1822035	84371	13791	8475	90.99	2.17
Con	Con6	bin4	MAG293	111	2499801	115797	42676	22521	90.97	0.68
Con	Con17	bin5	MAG1639	406	2047285	26987	6205	5043	90.96	1.77
Con	Con5	bin51	MAG3004	209	1872640	50195	13496	8960	90.96	2.61
Con	Con45	bin31	MAG1051	233	1781819	30756	10182	7647	90.94	0.67
Con	Con12	bin15	MAG1981	766	6577833	77143	12844	8587	90.87	4.39
Con	Con45	bin42	MAG1743	125	2092467	147350	28632	16740	90.87	0.04
Con	Con24	bin40	MAG2723	423	1835845	28885	5920	4340	90.82	0.59
Con	Con49	bin27	MAG2170	261	2409175	49763	13191	9231	90.79	2.68
Con	Con24	bin1	MAG254	350	2858994	44716	11121	8169	90.78	1.27
Con	Con3	bin20	MAG1163	196	3690995	126757	31757	18832	90.78	4.86
Con	Con32	bin42	MAG2917	866	2860783	18917	4163	3303	90.77	1.69
Con	Con39	bin10	MAG1701	646	2510571	21298	4822	3886	90.64	1.57
Con	Con18	bin29	MAG3040	65	2500405	155367	58335	38468	90.6	1.01
Con	Con20	bin1	MAG3125	82	2573939	150706	49235	31390	90.6	1.01
Con	Con45	bin55	MAG2914	446	2612129	59851	8357	5857	90.57	1.89
Con	Con49	bin47	MAG1965	391	1845184	24698	5418	4719	90.55	2.68
Con	Con2	bin48	MAG2439	239	2267769	44140	13391	9489	90.48	2.36
Con	Con6	bin25	MAG3304	343	2471747	38440	10236	7206	90.48	0.67
Con	Con49	bin63	MAG2601	334	2557870	41194	9943	7658	90.43	2.85
Con	Con19	bin20	MAG2992	137	2365071	174200	40664	17263	90.35	2.22
Con	Con38	bin52	MAG2305	62	1540705	116179	36594	24850	90.32	0
Con	Con52	bin28	MAG1601	60	2543281	280235	73934	42388	90.32	0
Con	Con1	bin59	MAG1327	239	3647747	87997	26152	15263	90.18	3.97
Con	Con20	bin38	MAG770	272	2300426	36402	12873	8457	90.08	2.11
Con	Con38	bin66	MAG2190	81	2262962	107899	56153	27938	90.06	0.29
Con	Con45	bin25	MAG38	267	1857775	40381	8862	6958	90.04	1.6
Con	Con45	bin52	MAG2718	451	2515753	31428	7041	5578	90.03	3.19
Con	Con34	bin48	MAG2629	274	3145045	61164	17684	11478	90.02	0.34
Con	Con4	bin41	MAG564	11	1893214	605077	184832	172110	89.93	0
Con	Con21	bin3	MAG2181	586	2435189	18597	5655	4156	89.86	0

Con	Con49	bin86	MAG3295	63	1695059	99116	44971	26906	89.81	1.34
Con	Con8	bin29	MAG822	87	2581594	191682	64587	29673	89.8	0
Con	Con18	bin16	MAG3461	628	4212385	119930	11914	6708	89.79	3.1
Con	Con9	bin4	MAG1778	137	2207576	149872	30372	16114	89.77	1.01
Con	Con34	bin16	MAG2315	438	2104567	25493	5697	4805	89.72	0
Con	Con45	bin37	MAG751	90	2405297	115798	42860	26726	89.6	0.89
Con	Con50	bin37	MAG3410	610	3517677	34357	7451	5767	89.54	1.18
Con	Con11	bin27	MAG1974	139	3203409	176915	62565	23046	89.45	0
Con	Con44	bin26	MAG3227	270	2338241	45664	13091	8660	89.43	2.25
Con	Con17	bin7	MAG962	409	1944345	24431	6378	4754	89.33	2.18
Con	Con21	bin24	MAG1932	290	1967157	39084	9508	6783	89.3	2.09
Con	Con10	bin7	MAG2276	150	3134484	117443	34673	20897	89.29	0.37
Con	Con17	bin38	MAG3147	615	3827180	44701	8264	6223	89.26	1.67
Con	Con17	bin3	MAG2866	480	1638339	21353	4213	3413	89.24	1.4
Con	Con30	bin11	MAG663	416	3977139	70127	12094	9560	89.14	0.95
Con	Con5	bin21	MAG816	249	2032042	38039	12290	8161	89.06	0.14
Con	Con4	bin31	MAG889	432	1520536	24828	4756	3520	88.91	4.2
Con	Con2	bin64	MAG810	299	1909556	44753	8033	6386	88.89	0.75
Con	Con35	bin43	MAG2342	117	2254326	98819	28123	19268	88.81	1.02
Con	Con12	bin19	MAG2321	878	4908006	38196	8281	5590	88.8	3.43
Con	Con24	bin10	MAG2153	89	2975981	154870	66550	33438	88.79	1.24
Con	Con6	bin13	MAG511	396	2399128	32723	7539	6058	88.79	1.34
Con	Con19	bin6	MAG166	278	2473062	55467	14309	8896	88.71	4.03
Con	Con4	bin13	MAG2565	588	2172584	35796	5167	3695	88.68	4.03
Con	Con9	bin1	MAG1125	462	2199328	29572	6206	4760	88.68	0
Con	Con44	bin30	MAG458	689	2351624	16578	4198	3413	88.66	2.24
Con	Con5	bin35	MAG1688	114	3203011	109214	41407	28097	88.63	0
Con	Con53	bin3	MAG3498	52	1891153	167317	50558	36368	88.62	0
Con	Con1	bin66	MAG2420	231	1880744	45434	11227	8142	88.59	0.36
Con	Con10	bin51	MAG1865	682	2038380	15659	3528	2989	88.59	1.68
Con	Con48	bin40	MAG133	520	2453428	40239	6821	4718	88.52	1.68
Con	Con12	bin32	MAG247	456	2299443	23366	6731	5043	88.5	2.05
Con	Con28	bin31	MAG1073	509	2147269	31265	5101	4219	88.48	6.17

Con	Con26	bin20	MAG1063	92	2715069	191842	53308	29512	88.44	0.68
Con	Con49	bin43	MAG3001	146	2363489	97813	23877	16188	88.38	0
Con	Con35	bin26	MAG1363	496	2517113	82455	6443	5075	88.03	2.01
Con	Con5	bin12	MAG2069	320	1694311	28734	6658	5295	87.97	1.46
Con	Con21	bin36	MAG3485	292	1760624	51624	8519	6030	87.95	0.84
Con	Con10	bin16	MAG3203	242	1944274	62327	11975	8034	87.86	0.34
Con	Con3	bin18	MAG412	424	2312996	29454	6798	5455	87.8	3.24
Con	Con5	bin52	MAG51	490	1848986	19845	4676	3773	87.79	3.81
Con	Con49	bin36	MAG1857	147	1702979	65962	21860	11585	87.78	1.5
Con	Con32	bin39	MAG788	211	2769943	189947	80121	13128	87.76	5.8
Con	Con17	bin4	MAG3213	347	2354913	42923	9142	6786	87.61	3.43
Con	Con4	bin37	MAG551	328	1943230	46816	8906	5924	87.58	2.33
Con	Con48	bin20	MAG2789	218	1821860	55506	11615	8357	87.52	1.34
Con	Con34	bin18	MAG1460	264	1847390	41495	9422	6998	87.51	3.53
Con	Con31	bin3	MAG720	747	3000882	23533	4815	4017	87.47	1.58
Con	Con30	bin52	MAG983	560	1390838	14396	2815	2484	87.4	6.31
Con	Con5	bin14	MAG922	105	2211689	151391	33156	21064	87.36	0
Con	Con27	bin43	MAG3085	125	2030532	105217	30153	16244	87.35	1.02
Con	Con51	bin30	MAG869	214	2023914	58220	13501	9458	87.35	1.05
Con	Con39	bin39	MAG2926	438	1840891	22831	5866	4203	87.33	4.72
Con	Con50	bin15	MAG1796	609	2056526	15304	4167	3377	87.03	2.24
Con	Con51	bin33	MAG944	84	2817519	188516	54609	33542	86.8	0.5
Con	Con10	bin59	MAG656	790	2471805	32395	3843	3129	86.71	2.09
Con	Con53	bin14	MAG54	123	3181391	219468	48635	25865	86.63	0.13
Con	Con22	bin24	MAG1820	32	1935729	458304	106185	60492	86.58	1.34
Con	Con35	bin40	MAG563	88	2237162	275178	84350	25422	86.58	0
Con	Con24	bin43	MAG256	155	1995384	72536	20205	12873	86.56	1.75
Con	Con22	bin18	MAG229	537	2291600	50581	6795	4267	86.47	2.56
Con	Con54	bin20	MAG2548	675	1888476	16065	3308	2798	86.43	3.58
Con	Con27	bin27	MAG1982	503	1840543	33242	4793	3659	86.4	2.9
Con	Con4	bin5	MAG1356	457	3212006	74636	15020	7028	86.13	0.86
Con	Con51	bin35	MAG2772	578	1542483	15733	3132	2669	86.13	1.8
Con	Con24	bin29	MAG2284	624	1827185	17086	3619	2928	86.05	5.3

Con	Con40	bin49	MAG278	1088	4069326	30678	5013	3740	86.02	4.15
Con	Con19	bin34	MAG2103	39	1791283	183327	83911	45930	85.91	0.67
Con	Con39	bin6	MAG2936	574	2608435	26456	5627	4544	85.89	6.75
Con	Con34	bin1	MAG2916	223	2574032	84847	23884	11543	85.7	1.75
Con	Con25	bin33	MAG1757	498	2048784	21954	4913	4114	85.68	1.85
Con	Con50	bin28	MAG561	501	1791043	29465	4812	3575	85.45	2.06
Con	Con49	bin8	MAG1590	95	1117220	96345	21819	11760	85.39	3.53
Con	Con2	bin60	MAG2157	409	2385619	41825	7624	5833	85.32	2.62
Con	Con2	bin3	MAG3222	118	2645250	94303	36899	22417	85.3	0.79
Con	Con24	bin44	MAG2707	1115	3392829	22904	4191	3043	85.24	5.99
Con	Con11	bin21	MAG353	601	1965702	19471	3934	3271	85.22	3.83
Con	Con21	bin34	MAG886	492	2284749	28878	5867	4644	85.17	2.64
Con	Con4	bin34	MAG1274	398	2335847	31790	7746	5869	85.01	1.72
Con	Con35	bin42	MAG1557	362	2139323	39611	7896	5910	84.9	3.91
Con	Con44	bin40	MAG1543	243	1722805	59621	11206	7090	84.87	0.45
Con	Con44	bin43	MAG868	594	1841135	22240	3806	3100	84.86	1.08
Con	Con4	bin60	MAG2323	838	2579602	20548	3624	3078	84.85	2.2
Con	Con49	bin74	MAG505	125	1729790	73250	32686	13838	84.83	4.36
Con	Con49	bin87	MAG344	253	2027751	47744	11708	8015	84.81	0.68
Con	Con30	bin50	MAG1128	565	2824339	43148	6231	4999	84.79	3.18
Con	Con40	bin26	MAG2470	386	2266606	42458	7517	5872	84.66	2.24
Con	Con19	bin51	MAG1755	363	1601875	48609	6316	4413	84.58	0.69
Con	Con27	bin20	MAG3028	897	4283248	36420	5663	4775	84.52	3.54
Con	Con40	bin12	MAG949	315	2070811	53927	9110	6574	84.36	2.45
Con	Con44	bin24	MAG1267	103	2156454	181776	86654	20936	84.26	0.47
Con	Con38	bin39	MAG2106	741	2180328	14451	3476	2942	84.21	3.86
Con	Con39	bin21	MAG2232	416	1588925	16952	4464	3820	84.2	3.58
Con	Con4	bin62	MAG3428	547	1956906	14697	4345	3578	84.09	2.81
Con	Con25	bin10	MAG1768	82	2404553	176744	89366	29324	84	0.79
Con	Con26	bin13	MAG2023	48	1790091	250163	95787	37294	83.89	0
Con	Con50	bin29	MAG1825	99	2173225	140565	50718	21952	83.89	0.67
Con	Con27	bin10	MAG364	731	1926662	10098	3131	2636	83.83	2.45
Con	Con8	bin12	MAG3324	184	1950154	51614	14488	10599	83.81	2.52

Con	Con25	bin20	MAG1886	85	2289017	138327	41509	26930	83.8	0.84
Con	Con50	bin45	MAG1708	94	2685592	170834	48683	28570	83.73	0.32
Con	Con5	bin38	MAG461	593	1617829	14613	3179	2728	83.72	3.1
Con	Con2	bin37	MAG2690	40	1717249	148158	70371	42931	83.56	0
Con	Con20	bin8	MAG3106	592	2022146	17981	4109	3416	83.48	1.66
Con	Con35	bin15	MAG2622	557	2303719	32046	5639	4136	83.39	0.23
Con	Con10	bin46	MAG1723	553	1504901	14410	3153	2721	83.34	2.07
Con	Con2	bin35	MAG1811	420	2141927	51342	8981	5100	83.32	4.46
Con	Con31	bin23	MAG1468	781	2056473	14085	3041	2633	83.31	0.58
Con	Con32	bin28	MAG1790	506	1550102	13682	3638	3063	83.29	2.33
Con	Con48	bin2	MAG3159	309	1771695	27834	7731	5734	83.17	0.12
Con	Con40	bin42	MAG2671	348	2320437	66572	15572	6668	83.15	2.68
Con	Con21	bin35	MAG2006	685	2653611	16992	4522	3874	83.11	2.82
Con	Con40	bin8	MAG64	383	2053246	25739	6694	5361	82.96	2.95
Con	Con51	bin57	MAG73	753	2168431	16980	3304	2880	82.93	3.58
Con	Con17	bin32	MAG569	30	1621152	206573	95784	54038	82.89	0
Con	Con25	bin1	MAG990	81	1931231	139834	43901	23842	82.77	0.67
Con	Con4	bin10	MAG2407	465	1990955	30504	5137	4282	82.71	2.38
Con	Con4	bin6	MAG1484	41	2077687	189168	84344	50675	82.46	0
Con	Con2	bin11	MAG2411	139	2234969	98104	26009	16079	82.31	4.46
Con	Con40	bin63	MAG2641	368	2093560	28301	7101	5689	82.31	0.93
Con	Con35	bin25	MAG410	929	3932428	19361	4911	4233	82.25	1.25
Con	Con34	bin23	MAG3372	515	1949886	18343	4702	3786	82.23	3.3
Con	Con27	bin6	MAG2039	626	2218379	32606	4850	3544	82.17	1.29
Con	Con31	bin1	MAG48	318	1759805	28713	7938	5534	82.09	0.57
Con	Con6	bin8	MAG2356	846	2336522	16786	3198	2762	81.74	2.6
Con	Con35	bin38	MAG1813	304	1638458	33452	7077	5390	81.72	2.33
Con	Con20	bin26	MAG1237	325	2346479	51950	10491	7220	81.62	9.94
Con	Con34	bin3	MAG2333	463	2305624	22391	6071	4980	81.51	3.1
Con	Con51	bin13	MAG3127	102	3907407	200160	74588	38308	81.38	2.59
Con	Con35	bin18	MAG3204	600	1811802	34325	3823	3020	81.32	2.6
Con	Con44	bin41	MAG2736	76	2101769	131961	46246	27655	81.29	0
Con	Con38	bin42	MAG135	67	2021297	122062	46561	30169	81.22	0.63

Con	Con44	bin16	MAG228	180	2756208	94480	36284	15312	81.21	1.34
Con	Con1	bin25	MAG830	610	2450819	21689	4624	4018	81.13	3.69
Con	Con52	bin6	MAG1150	324	1664234	27341	6555	5137	80.92	0.73
Con	Con52	bin32	MAG2545	34	1654174	179872	99724	48652	80.87	0
Con	Con38	bin9	MAG1177	250	2820340	76303	24244	11281	80.8	2.17
Con	Con22	bin7	MAG2741	760	2053455	20967	2996	2702	80.77	2.59
Con	Con32	bin11	MAG1199	129	3523884	157413	41723	27317	80.7	3.51
Con	Con49	bin70	MAG653	132	1749900	158785	24950	13257	80.65	0
Con	Con21	bin1	MAG728	423	2322284	30589	6776	5490	80.54	0
Con	Con4	bin47	MAG1690	837	2355461	15500	3170	2814	80.49	0.24
Con	Con53	bin4	MAG1402	734	2815978	20106	4435	3836	80.46	5.38
Con	Con19	bin1	MAG165	654	1849004	28430	3343	2827	80.29	4.03
Con	Con53	bin20	MAG82	662	1910088	14342	3561	2885	80.22	4.46
Con	Con1	bin44	MAG2191	446	1897906	29718	5441	4255	80.07	3.91
Con	Con39	bin16	MAG3491	203	2610737	83357	22328	12861	80.02	0
Con	Con45	bin49	MAG1148	405	2253427	43194	8651	5564	79.98	0.37
Con	Con1	bin9	MAG3420	729	1740278	10736	2746	2387	79.82	3.22
Con	Con39	bin7	MAG1181	789	2107766	13219	3084	2671	79.65	2.97
Con	Con9	bin13	MAG3402	1213	2960351	16733	2762	2441	79.62	2.43
Con	Con25	bin26	MAG2725	574	2893015	38270	5835	5040	79.5	6.98
Con	Con49	bin85	MAG3281	98	2102811	126771	41306	21457	79.38	0.68
Con	Con45	bin28	MAG2318	840	2325688	12635	3140	2769	79.15	1.36
Con	Con49	bin52	MAG348	922	2079591	9511	2453	2256	79.13	3.5
Con	Con32	bin27	MAG1905	471	1759781	19148	4252	3736	78.99	1.75
Con	Con32	bin45	MAG2918	571	1661384	12340	3568	2910	78.92	2.04
Con	Con32	bin25	MAG3447	121	3312325	336666	96915	27375	78.92	0.36
Con	Con44	bin13	MAG2301	463	1336337	11409	3382	2886	78.88	0
Con	Con38	bin51	MAG2743	56	1595578	150999	61763	28492	78.79	1.34
Con	Con5	bin22	MAG2592	1214	2433464	8784	2135	2005	78.73	4.68
Con	Con25	bin6	MAG2659	537	2010472	18884	4277	3744	78.65	3.06
Con	Con3	bin50	MAG2631	57	1859834	166418	58669	32629	78.65	0.29
Con	Con20	bin48	MAG1050	992	2196721	10725	2339	2214	78.62	3.46
Con	Con19	bin55	MAG2436	471	1620200	12804	3775	3440	78.53	2.57

Con	Con6	bin38	MAG1159	699	2368116	28363	3817	3388	78.5	4.19
Con	Con27	bin36	MAG3506	81	2086851	114660	42516	25764	78.36	1.85
Con	Con39	bin32	MAG2400	994	2960984	28011	3746	2979	78.26	1.02
Con	Con30	bin43	MAG1351	88	2952021	163468	88561	33546	78.23	0
Con	Con45	bin51	MAG2431	164	2010503	54977	16600	12259	78.2	0
Con	Con38	bin48	MAG2004	518	2373953	27961	4984	4583	77.96	4.28
Con	Con48	bin11	MAG1207	62	1881643	129254	57352	30349	77.85	0.63
Con	Con50	bin18	MAG1488	593	1943918	17735	3564	3278	77.58	0
Con	Con25	bin8	MAG2272	535	1705945	16631	3421	3189	77.53	3.09
Con	Con34	bin8	MAG435	584	2318591	26707	6248	3970	77.53	0.08
Con	Con52	bin5	MAG781	638	1603866	10271	2759	2514	77.48	2.57
Con	Con49	bin6	MAG1562	249	1567829	48326	10574	6297	77.24	1.87
Con	Con49	bin66	MAG2705	786	1753583	8809	2382	2231	77.2	4.08
Con	Con20	bin12	MAG1741	279	2223868	83876	10596	7971	77.05	3.8
Con	Con52	bin40	MAG3012	729	1930674	14170	3093	2648	77	4.03
Con	Con32	bin37	MAG2919	32	1929835	268757	109126	60307	76.96	0.67
Con	Con24	bin2	MAG1467	484	2273698	35911	5571	4698	76.81	0.95
Con	Con38	bin24	MAG771	55	1299623	137011	60797	23630	76.76	2.24
Con	Con5	bin25	MAG1653	1130	3036470	13763	3131	2687	76.74	1.52
Con	Con34	bin10	MAG3153	587	1554624	18394	2962	2648	76.72	2.24
Con	Con53	bin21	MAG418	458	2018076	27735	5557	4406	76.57	1.41
Con	Con40	bin4	MAG1213	436	1613588	21924	4967	3701	76.56	1.36
Con	Con38	bin16	MAG2593	310	2469713	66851	11284	7967	76.54	1.49
Con	Con51	bin43	MAG2643	293	2043219	33592	8527	6973	76.43	0.69
Con	Con51	bin60	MAG920	164	2166826	80608	24589	13212	76.41	0.89
Con	Con46	bin15	MAG2205	166	1598800	187050	19809	9631	76.25	0.37
Con	Con44	bin3	MAG713	436	1401689	33740	4311	3215	76.18	0.58
Con	Con10	bin8	MAG1944	342	3542279	168154	26667	10358	76.16	1.05
Con	Con21	bin40	MAG2482	804	1699034	9775	2283	2113	76.13	5.83
Con	Con34	bin6	MAG2368	55	1572888	120625	46638	28598	76.11	1.48
Con	Con19	bin26	MAG3440	646	1663483	12162	2962	2575	76.02	2.35
Con	Con45	bin1	MAG2907	835	2837970	12986	3873	3399	75.94	2.82
Con	Con27	bin13	MAG1698	528	2300283	37246	5052	4357	75.75	2.08

Con	Con6	bin28	MAG445	229	2077544	105020	17802	9072	75.52	5.36
Con	Con17	bin28	MAG3443	70	2196372	161117	53741	31377	75.5	0
Con	Con5	bin30	MAG1419	1053	2348022	11924	2436	2230	75.43	2.37
Con	Con53	bin27	MAG310	285	2376326	60154	12128	8338	75.37	0.79
Con	Con35	bin6	MAG2195	606	2226330	21596	4289	3674	75.35	1.46
Con	Con34	bin19	MAG436	44	1511211	105733	54209	34346	75.21	2.82
Con	Con26	bin1	MAG2080	151	1820712	110910	30368	12058	75.17	0
Con	Con40	bin46	MAG1183	183	1763683	57607	14918	9638	75.05	2.05
Con	Con49	bin5	MAG3090	890	1830878	7359	2238	2057	75.04	3.71
Con	Con1	bin63	MAG2837	646	2322167	102573	4857	3595	75	5.17
Con	Con32	bin34	MAG324	138	2419272	124331	37687	17531	75	6.36
Con	Con52	bin11	MAG1461	448	2224345	46729	8391	4965	75	8.04
Con	Con27	bin54	MAG1605	897	2457538	13056	3218	2740	74.92	2.97
Con	Con40	bin54	MAG2364	66	2132706	150396	48040	32314	74.88	0
Con	Con49	bin89	MAG1475	400	1737689	28844	5018	4344	74.88	3.32
Con	Con49	bin40	MAG3252	152	1699946	99325	25806	11184	74.87	1.5
Con	Con20	bin3	MAG651	432	2038450	30494	5710	4719	74.84	0.73
Con	Con39	bin14	MAG1160	649	1477337	8612	2615	2276	74.76	1.85
Con	Con10	bin21	MAG1087	488	1547334	19239	3813	3171	74.74	3.16
Con	Con44	bin37	MAG2639	65	2621831	232821	101704	40336	74.68	0
Con	Con12	bin45	MAG351	69	1906802	134675	47430	27635	74.67	0
Con	Con22	bin5	MAG3437	670	1623596	18175	2645	2423	74.66	5.11
Con	Con6	bin1	MAG1990	101	1985788	141087	30823	19661	74.46	3.57
Con	Con1	bin21	MAG3029	927	1988556	8977	2363	2145	74.31	3.91
Con	Con5	bin45	MAG3091	67	2527421	159197	50026	37723	74.31	0
Con	Con17	bin43	MAG3087	527	1337012	8478	2914	2537	74.19	2.73
Con	Con25	bin29	MAG2265	448	2155144	48026	9544	4811	74.16	9.39
Con	Con3	bin35	MAG3105	148	2660328	123636	27081	17975	74.14	0
Con	Con17	bin52	MAG2409	1194	2316335	11008	2058	1940	74.08	4.21
Con	Con17	bin24	MAG361	142	1432054	39325	12936	10085	73.99	1.32
Con	Con46	bin5	MAG2908	877	2512287	19351	3556	2865	73.81	5.12
Con	Con3	bin52	MAG2637	619	1717033	14226	3183	2774	73.75	2.21
Con	Con48	bin31	MAG3392	287	2337301	53475	11795	8144	73.73	8.93

Con	Con3	bin4	MAG2984	166	2165754	70207	18674	13047	73.71	0.74
Con	Con21	bin5	MAG1364	746	3100175	45873	4796	4156	73.68	4.07
Con	Con53	bin8	MAG75	1260	2873322	14059	2552	2280	73.63	1.56
Con	Con12	bin16	MAG2544	62	2348509	251783	94249	37879	73.62	1.58
Con	Con27	bin32	MAG2827	40	1448494	145396	64431	36212	73.54	0.67
Con	Con9	bin3	MAG2869	194	1527911	48266	10368	7876	73.54	1.79
Con	Con19	bin45	MAG1246	567	1971869	17564	3914	3478	73.33	5.08
Con	Con49	bin9	MAG648	44	1187814	106254	61538	26996	73.09	1.79
Con	Con31	bin12	MAG132	568	1751915	18140	3811	3084	73.05	3.45
Con	Con54	bin9	MAG2532	1564	3899942	25938	2717	2494	72.98	2.03
Con	Con10	bin45	MAG2122	383	1714047	19702	5053	4475	72.95	1.36
Con	Con3	bin19	MAG2542	1129	2591192	13109	2509	2295	72.77	5.05
Con	Con24	bin35	MAG911	726	1629177	17629	2474	2244	72.67	2.21
Con	Con44	bin4	MAG787	97	1686749	111621	37973	17389	72.65	1.54
Con	Con8	bin20	MAG1354	1110	3977338	16075	3940	3583	72.63	3.32
Con	Con9	bin22	MAG2238	655	1625914	13939	2743	2482	72.55	1.74
Con	Con28	bin2	MAG2740	762	2568064	31403	3801	3370	72.54	0
Con	Con6	bin37	MAG1721	251	1634011	33815	7573	6510	72.48	0
Con	Con2	bin58	MAG966	516	2407521	20866	6100	4666	72.41	5.17
Con	Con34	bin14	MAG1225	1342	2871637	10369	2281	2140	72.32	5.11
Con	Con49	bin7	MAG1440	268	1175188	23911	5227	4385	72.24	3.37
Con	Con10	bin68	MAG2606	432	2283578	26162	6781	5286	72.15	2.27
Con	Con30	bin16	MAG2710	481	1361137	10063	2965	2830	72.14	1.73
Con	Con50	bin14	MAG2794	536	1600445	11034	3179	2986	72.06	1.08
Con	Con9	bin8	MAG2852	48	3170598	426420	100950	66054	72.03	0.12
Con	Con4	bin16	MAG2775	869	2047685	10638	2634	2356	72.01	1.81
Con	Con6	bin34	MAG874	697	1723840	10719	2737	2473	71.83	0.32
Con	Con27	bin60	MAG952	891	1870619	9521	2259	2099	71.77	1.02
Con	Con50	bin7	MAG908	441	1836635	27642	5717	4165	71.62	3.08
Con	Con24	bin37	MAG3196	1770	3744511	17139	2266	2116	71.61	3.8
Con	Con12	bin38	MAG2932	155	3071353	114253	29948	19815	71.55	3.92
Con	Con18	bin30	MAG725	140	2444119	98471	31802	17458	71.12	0.02
Con	Con9	bin16	MAG637	664	1424363	10157	2323	2145	70.9	3.55

Con	Con53	bin29	MAG362	587	4529722	58279	10385	7717	70.69	1.72
Con	Con35	bin29	MAG1130	130	2436160	131158	38573	18740	70.34	1.8
Con	Con27	bin50	MAG768	371	1851608	22981	6440	4991	70.23	1.79
Con	Con53	bin18	MAG118	80	2190777	119085	49272	27385	70.07	0
Con	Con25	bin13	MAG2549	510	1770363	26550	3957	3471	70.02	2.85
Con	Con11	bin13	MAG494	277	1655407	45594	10628	5976	69.88	1.58
Con	Con31	bin40	MAG2599	1272	2890359	10936	2496	2272	69.88	2.95
Con	Con54	bin26	MAG1953	755	1796886	11250	2677	2380	69.83	1.01
Con	Con12	bin10	MAG2964	851	2086575	9833	2745	2452	69.54	0.68
Con	Con29	bin11	MAG3068	566	1407839	11226	2986	2487	69.43	1.53
Con	Con51	bin11	MAG1829	1197	2310303	10169	2039	1930	69.38	1.32
Con	Con40	bin45	MAG61	226	2768722	97144	16521	12251	69.14	0
Con	Con32	bin31	MAG2377	794	1502180	11743	1955	1892	69.01	7.17
Con	Con38	bin27	MAG1202	83	2161057	110957	37684	26037	68.97	0
Con	Con11	bin2	MAG537	274	2511982	41949	11793	9168	68.89	0.63
Con	Con49	bin60	MAG1693	159	1683141	44000	14312	10586	68.75	0
Con	Con4	bin55	MAG514	153	1406167	43404	13343	9191	68.28	1.07
Con	Con38	bin40	MAG358	217	1275299	28030	7099	5877	68.26	5.94
Con	Con50	bin6	MAG1676	406	1214696	13227	3587	2992	68.25	8.04
Con	Con51	bin2	MAG3270	137	1825578	74153	17675	13325	68.21	0
Con	Con32	bin43	MAG1853	408	1740651	47760	5915	4266	68.1	0.32
Con	Con2	bin8	MAG137	702	1554104	10005	2385	2214	67.8	2.48
Con	Con5	bin26	MAG2990	66	2570659	157030	59128	38949	67.59	0.86
Con	Con28	bin11	MAG557	408	1594465	24373	5047	3908	67.42	3.57
Con	Con17	bin18	MAG3202	76	1771351	115335	38189	23307	67.35	0.44
Con	Con44	bin49	MAG1527	481	1762243	21394	4113	3664	67.32	0.58
Con	Con40	bin52	MAG1003	112	2356154	128853	29652	21037	67.24	0
Con	Con4	bin33	MAG917	461	1249504	19685	3136	2710	67.13	0
Con	Con29	bin17	MAG1434	528	1190682	14530	2479	2255	66.92	0.79
Con	Con32	bin14	MAG691	149	1971404	130535	32466	13231	66.88	0.67
Con	Con48	bin1	MAG2247	695	1694859	18748	2914	2439	66.58	7.07
Con	Con48	bin35	MAG2516	332	1743058	55636	6413	5250	66.49	0.79
Con	Con12	bin18	MAG2289	152	2136803	81125	21818	14058	66.48	0.65

Con	Con51	bin61	MAG1703	214	2721966	93362	23814	12719	66.48	4.11
Con	Con44	bin46	MAG2806	154	2049834	128375	24503	13311	66.46	0.11
Con	Con49	bin41	MAG1070	113	2270312	90546	36123	20091	66.38	3.45
Con	Con40	bin3	MAG1431	252	2550626	58041	13072	10122	66.18	0.69
Con	Con20	bin37	MAG837	524	1039701	8601	2148	1984	66.17	2.01
Con	Con49	bin79	MAG1695	66	2114309	224999	56911	32035	65.83	0.19
Con	Con50	bin8	MAG989	630	1283399	7076	2189	2037	65.62	1.41
Con	Con40	bin9	MAG2996	244	2139382	49797	11905	8768	65.54	0
Con	Con24	bin5	MAG341	830	2006973	19819	2693	2418	65.52	1.72
Con	Con39	bin31	MAG495	834	1618650	9461	2053	1941	65.15	1.4
Con	Con10	bin39	MAG2310	1513	2832004	11264	1940	1872	65.06	2.21
Con	Con30	bin48	MAG811	1159	3605442	15901	3575	3111	65.06	0.97
Con	Con48	bin26	MAG1978	804	1563168	6742	2059	1944	65.04	2.38
Con	Con51	bin54	MAG3182	117	2705845	208417	50191	23127	65	3.45
Con	Con40	bin21	MAG1925	56	2152845	156111	55918	38444	64.97	0
Con	Con34	bin32	MAG1058	54	1544584	183166	63660	28603	64.77	0
Con	Con12	bin48	MAG2055	252	1833434	38400	10372	7276	64.73	3.45
Con	Con35	bin14	MAG397	758	1501372	7969	2111	1981	64.72	2.29
Con	Con31	bin34	MAG718	1067	3307632	50970	3635	3100	64.66	0
Con	Con10	bin56	MAG1442	334	644677	6768	2065	1930	64.54	1.17
Con	Con31	bin14	MAG794	962	1678044	7886	1791	1744	64.52	3.08
Con	Con27	bin39	MAG428	553	1268809	11771	2533	2294	64.5	0.89
Con	Con3	bin9	MAG143	448	1758699	19365	4355	3926	64.42	7.76
Con	Con26	bin4	MAG1093	821	1703053	10750	2195	2074	63.48	1.24
Con	Con5	bin13	MAG3132	174	4747740	315274	50831	27286	63.43	0
Con	Con26	bin6	MAG529	699	1761450	11864	2520	2520	63.2	4.25
Con	Con12	bin24	MAG1002	214	1517646	42362	8167	7092	63.02	1.34
Con	Con19	bin52	MAG2941	1441	3744978	14103	2931	2599	62.93	0.86
Con	Con17	bin26	MAG2523	439	1217315	10546	2982	2773	62.85	2.36
Con	Con19	bin14	MAG1167	666	1590337	15540	2721	2388	62.63	2.91
Con	Con9	bin26	MAG904	422	1278946	19391	3384	3031	62.56	0.4
Con	Con34	bin21	MAG1331	892	1575434	6718	1860	1766	62.55	1.92
Con	Con30	bin29	MAG622	583	1360659	20525	2471	2334	62.5	2.32

Con	Con28	bin16	MAG991	360	1179311	22034	3493	3276	62.49	0.55
Con	Con2	bin65	MAG3233	669	1323062	9991	2114	1978	62.33	2.1
Con	Con53	bin9	MAG2774	871	3439075	19999	4337	3948	62.25	0.95
Con	Con3	bin31	MAG1396	561	1070682	14853	2013	1909	62.14	1.09
Con	Con19	bin43	MAG1039	743	1796176	20095	2634	2417	62.07	1.72
Con	Con17	bin37	MAG614	480	1938930	54585	5676	4039	61.49	2.03
Con	Con48	bin17	MAG632	85	1556252	126635	66864	18309	61.4	0
Con	Con1	bin67	MAG1987	919	1960148	11771	2297	2133	61.25	3.46
Con	Con31	bin28	MAG818	636	1529600	14618	2811	2405	61.21	0.86
Con	Con6	bin21	MAG1021	449	1335821	11455	3186	2975	61.1	0.85
Con	Con25	bin16	MAG629	1592	2894171	7064	1929	1818	61.08	9.95
Con	Con10	bin38	MAG1525	1015	1936738	10517	2050	1908	61.04	3.57
Con	Con34	bin38	MAG2221	179	1115279	27256	7050	6231	60.93	0.67
Con	Con1	bin42	MAG1647	648	1392305	15026	2308	2149	60.83	0
Con	Con10	bin18	MAG284	190	1639601	37937	12712	8629	60.83	1.79
Con	Con10	bin29	MAG1973	386	1589350	20399	4932	4117	60.71	3.57
Con	Con4	bin20	MAG3119	381	1147123	11007	3195	3011	60.71	2.28
Con	Con26	bin9	MAG1171	783	1395284	5765	1815	1782	59.96	0.94
Con	Con34	bin37	MAG3205	738	1511274	6574	2259	2048	59.7	1.45
Con	Con8	bin26	MAG2229	860	1672213	11101	2076	1944	59.6	2.47
Con	Con49	bin35	MAG2375	111	1239271	117289	27257	11165	59.55	1.46
Con	Con17	bin60	MAG1147	769	1504226	7788	2047	1956	59.34	0.69
Con	Con32	bin29	MAG1779	1038	2579619	8315	2706	2485	59.07	2.03
Con	Con28	bin29	MAG2399	381	1026228	19106	3318	2694	58.94	1.85
Con	Con11	bin17	MAG17	273	1629492	40567	6902	5969	58.53	0
Con	Con20	bin14	MAG3163	634	1242563	10413	1999	1960	58.49	0.67
Con	Con51	bin41	MAG3409	524	1675678	13724	3365	3198	58.36	0
Con	Con1	bin41	MAG1618	736	2230552	14746	3151	3031	58.25	4.3
Con	Con50	bin17	MAG36	65	1439574	150016	56635	22147	58.23	0
Con	Con48	bin43	MAG357	630	1039050	5532	1640	1649	58.15	7.68
Con	Con8	bin2	MAG891	459	1061560	8563	2622	2313	57.98	1.28
Con	Con34	bin46	MAG1301	703	1374851	11509	2105	1956	57.88	5.65
Con	Con12	bin25	MAG1157	587	3028710	43362	6322	5160	57.76	3.45

Con	Con5	bin7	MAG1031	1643	4095002	37332	2833	2492	57.76	1.72
Con	Con32	bin20	MAG3267	305	1618592	26955	6118	5307	57.75	7.95
Con	Con5	bin23	MAG2193	982	1710870	5070	1771	1742	57.51	6.58
Con	Con2	bin21	MAG937	345	2752058	105972	25119	7977	57.5	8.93
Con	Con39	bin44	MAG1740	272	1633870	35338	7353	6007	57.14	1.79
Con	Con19	bin5	MAG1142	314	1126938	19342	4044	3589	57.02	0.46
Con	Con19	bin12	MAG336	206	888267	17680	4811	4312	56.77	0
Con	Con45	bin9	MAG1856	599	1357241	11866	2526	2266	56.75	3.13
Con	Con4	bin42	MAG785	315	1624936	30929	6194	5159	56.61	1.79
Con	Con28	bin19	MAG1188	1325	2407795	9188	1873	1817	56.54	1.67
Con	Con18	bin9	MAG315	1148	2003767	8316	1764	1745	56.46	6.32
Con	Con53	bin26	MAG422	505	973529	7031	1994	1928	56.33	0.22
Con	Con9	bin10	MAG1750	1010	1795615	6450	1821	1778	56.15	4.11
Con	Con22	bin23	MAG2769	408	2002768	24428	5838	4909	56.14	0
Con	Con50	bin43	MAG783	69	1870171	220173	66070	27104	56.12	0
Con	Con8	bin41	MAG332	239	1609705	25441	8150	6735	56.06	6.9
Con	Con49	bin29	MAG657	26	1284419	126733	92159	49401	56.04	0
Con	Con28	bin5	MAG448	739	1344731	6930	1896	1820	56.02	2.43
Con	Con40	bin28	MAG709	591	1908211	17427	3532	3229	55.96	0.88
Con	Con49	bin51	MAG2745	68	2014594	223602	41887	29626	55.93	0
Con	Con31	bin6	MAG646	826	4343453	103282	9366	5258	55.75	0.63
Con	Con18	bin5	MAG2582	895	1796357	9202	2150	2007	55.62	2.57
Con	Con2	bin53	MAG3234	634	1172129	7915	1920	1849	55.39	0
Con	Con34	bin25	MAG1008	284	1133049	13600	4308	3990	55.39	1.01
Con	Con21	bin37	MAG798	665	1243919	8359	1928	1871	55.32	1.9
Con	Con46	bin3	MAG3259	140	1254671	53034	13517	8962	55.15	1.12
Con	Con24	bin12	MAG2884	374	1345004	24437	3940	3596	55.09	1.73
Con	Con49	bin14	MAG93	424	1130179	7940	2690	2666	54.83	0
Con	Con48	bin30	MAG3464	139	1510220	42533	15579	10865	54.82	0
Con	Con17	bin31	MAG2922	70	2275571	178476	42417	32508	54.66	0
Con	Con26	bin26	MAG3497	998	2175660	11544	2380	2180	54.49	1.29
Con	Con12	bin12	MAG150	314	1356024	26008	4885	4319	54.47	1.72
Con	Con3	bin13	MAG1292	1097	1569296	6560	1334	1431	54.24	7.48

Con	Con26	bin27	MAG2760	1092	1634718	5679	1452	1497	54.03	7.14
Con	Con11	bin10	MAG103	26	1237619	185102	142210	47601	53.92	0.92
Con	Con34	bin60	MAG110	533	1085687	11210	2160	2037	53.88	0.66
Con	Con29	bin19	MAG2655	356	5035386	251121	32384	14144	53.79	8.62
Con	Con28	bin25	MAG2253	592	1037446	7309	1770	1752	53.67	0.9
Con	Con17	bin59	MAG2662	618	1122791	9455	1836	1817	53.61	1.72
Con	Con22	bin6	MAG520	1276	2207967	7434	1740	1730	53.15	7.24
Con	Con52	bin19	MAG3061	718	1516049	10090	2317	2111	53.13	0.63
Con	Con1	bin2	MAG724	733	1251381	8957	1726	1707	53.12	0.34
Con	Con46	bin8	MAG40	671	1478326	10347	2390	2203	53.09	1.42
Con	Con17	bin41	MAG929	790	2508305	18183	3411	3175	52.98	0
Con	Con51	bin21	MAG1887	225	884145	17844	4462	3930	52.82	0.69
Con	Con44	bin51	MAG2000	1045	2092985	8880	2197	2003	52.64	1.52
Con	Con4	bin15	MAG2121	806	1638891	18440	2093	2033	52.6	1.31
Con	Con40	bin32	MAG98	188	2182713	127456	42840	11610	52.46	3.62
Con	Con48	bin4	MAG1541	468	1151638	35578	2793	2461	52.39	9.53
Con	Con2	bin42	MAG2393	817	2333181	14444	2969	2856	52.26	7.02
Con	Con20	bin31	MAG2868	740	1178099	5658	1591	1592	52.25	2.01
Con	Con20	bin22	MAG636	1089	1885932	6816	1746	1732	52.23	1.3
Con	Con34	bin53	MAG1838	538	1066692	12583	2072	1983	52.2	0.22
Con	Con38	bin1	MAG913	555	1035112	5541	1975	1865	52.09	1.43
Con	Con52	bin25	MAG197	141	1447869	51236	17360	10269	51.79	0
Con	Con30	bin33	MAG9	245	652391	30664	3062	2663	51.78	0.3
Con	Con12	bin21	MAG1805	826	1603251	7543	1997	1941	51.75	2.14
Con	Con21	bin20	MAG900	843	1281828	3999	1499	1521	51.43	4.03
Con	Con8	bin39	MAG3172	606	1429232	13838	2649	2358	51.42	3.04
Con	Con17	bin10	MAG3482	919	1800838	7155	2116	1960	51.23	0
Con	Con2	bin69	MAG1563	1023	1738822	6741	1738	1700	51.12	4.11
Con	Con34	bin9	MAG702	473	972684	26288	2134	2056	51.11	0
Con	Con48	bin8	MAG666	364	1889041	41058	6447	5190	50.88	0.88
Con	Con26	bin11	MAG2441	560	1609335	10395	3063	2874	50.83	0.27
Con	Con6	bin24	MAG320	1050	1606338	5170	1504	1530	50.42	9.91
Con	Con34	bin13	MAG2626	489	882793	8583	1869	1805	50.39	0

Con	Con49	bin45	MAG1756	282	1831993	28075	7116	6496	50.34	1.72
Con	Con24	bin31	MAG3154	322	4125485	118732	19804	12812	50	0
