# Supplementary material for: Gut microbial community of patients with Parkinson’s disease analyzed using metagenome-assembled genomes
Source: Neural Regen Res. 2025 Sep 29;21(8):3815–23. doi: 10.4103/NRR.NRR-D-25-00420 (PMC13460800; doi:10.4103/NRR.NRR-D-25-00420)
Supplement: Supplementary file 2 [file NRR-21-3815_Suppl2.pdf]

Additional Table 2 Taxonomy labels of MAGs annotated by GTDB-TK

Group	Bin Id	Bin	MAG Id	Domain	Phylum	Class	Order	Family	Genus	Species
PD	PD29	bin19	MAG270	d__Bacteria	p__Bacillota_A	c__Clostridia	o__Christensenellales	f__CAG-74	g__Limiplasma	s__Limiplasma sp900550955
PD	PD97	bin3	MAG1132	d__Bacteria	p__Bacillota_A	c__Clostridia	o__Christensenellales	f__CAG-74	g__Limiplasma	s__Limiplasma merdipullorum
PD	PD2	bin57	MAG2381	d__Bacteria	p__Bacillota_A	c__Clostridia	o__Christensenellales	f__CAG-74	g__Limiplasma	s__Limiplasma merdipullorum
PD	PD70	bin10	MAG1901	d__Bacteria	p__Bacillota_A	c__Clostridia	o__Christensenellales	f__CAG-74	g__Limiplasma	s__Limiplasma merdipullorum
PD	PD64	bin40	MAG2505	d__Bacteria	p__Bacillota_A	c__Clostridia	o__Christensenellales	f__CAG-74	g__Limiplasma	s__Limiplasma merdipullorum
PD	PD22	bin38	MAG2873	d__Bacteria	p__Bacillota_A	c__Clostridia	o__Christensenellales	f__CAG-74	g__Limiplasma	s__Limiplasma merdipullorum
PD	PD58	bin54	MAG1566	d__Bacteria	p__Bacillota_A	c__Clostridia	o__Christensenellales	f__CAG-74	g__Limiplasma	s__Limiplasma merdipullorum
PD	PD29	bin46	MAG2057	d__Bacteria	p__Bacillota_A	c__Clostridia	o__Christensenellales	f__CAG-138	g__Phil1	s__Phil1 sp001940855
PD	PD41	bin20	MAG1934	d__Bacteria	p__Bacillota_A	c__Clostridia	o__Christensenellales	f__CAG-138	g__Phil1	s__Phil1 sp001940855
PD	PD58	bin42	MAG543	d__Bacteria	p__Bacillota_A	c__Clostridia	o__Christensenellales	f__CAG-138	g__Phil1	s__Phil1 sp001940855
PD	PD12	bin34	MAG368	d__Bacteria	p__Bacillota_A	c__Clostridia	o__Christensenellales	f__CAG-138	g__Phil1	s__Phil1 sp001940855
PD	PD64	bin35	MAG1793	d__Bacteria	p__Bacillota_A	c__Clostridia	o__Christensenellales	f__CAG-138	g__Phil1	s__Phil1 sp001940855
PD	PD7	bin31	MAG1550	d__Bacteria	p__Bacillota_A	c__Clostridia	o__Christensenellales	f__CAG-138	g__Phil1	s__Phil1 sp001940855
PD	PD94	bin49	MAG3266	d__Bacteria	p__Bacillota_A	c__Clostridia	o__Christensenellales	f__CAG-138	g__Pelethousia	s__Pelethousia gallinarum
PD	PD57	bin59	MAG2901	d__Bacteria	p__Bacillota_A	c__Clostridia	o__Christensenellales	f__CAG-138	g__Pelethousia	s__Pelethousia gallinarum
PD	PD46	bin83	MAG669	d__Bacteria	p__Bacillota_A	c__Clostridia	o__Christensenellales	f__CAG-138	g__PeH17	s__PeH17 sp000435055
PD	PD56	bin52	MAG899	d__Bacteria	p__Bacillota_A	c__Clostridia	o__Christensenellales	f__CAG-138	g__PeH17	s__PeH17 sp000435055
PD	PD58	bin5	MAG3358	d__Bacteria	p__Bacillota_A	c__Clostridia	o__Christensenellales	f__CAG-138	g__PeH17	s__PeH17 sp000435055
PD	PD94	bin5	MAG1837	d__Bacteria	p__Bacillota_A	c__Clostridia	o__Christensenellales	f__CAG-138	g__PeH17	s__PeH17 sp000435055
PD	PD98	bin32	MAG1896	d__Bacteria	p__Bacillota_A	c__Clostridia	o__Christensenellales	f__CAG-138	g__PeH17	s__PeH17 sp000435055
PD	PD25	bin20	MAG925	d__Bacteria	p__Bacillota_A	c__Clostridia	o__Christensenellales	f__CAG-138	g__PeH17	s__PeH17 sp000435055
PD	PD73	bin39	MAG464	d__Bacteria	p__Bacillota_A	c__Clostridia	o__Christensenellales	f__CAG-138	g__PeH17	s__PeH17 sp000435055
PD	PD41	bin27	MAG3228	d__Bacteria	p__Bacillota_A	c__Clostridia	o__Christensenellales	f__CAG-138	g__PeH17	s__PeH17 sp000435055
PD	PD7	bin40	MAG3102	d__Bacteria	p__Bacillota_A	c__Clostridia	o__Christensenellales	f__CAG-138	g__PeH17	s__PeH17 sp000435055
PD	PD70	bin2	MAG2750	d__Bacteria	p__Bacillota_A	c__Clostridia	o__Christensenellales	f__CAG-138	g__PeH17	s__PeH17 sp000435055
PD	PD57	bin25	MAG1787	d__Bacteria	p__Bacillota_A	c__Clostridia	o__Christensenellales	f__CAG-138	g__PeH17	s__PeH17 sp000435055
PD	PD72	bin16	MAG782	d__Bacteria	p__Bacillota_A	c__Clostridia	o__Christensenellales	f__CAG-138	g__PeH17	s__PeH17 sp000435055
PD	PD2	bin29	MAG1229	d__Bacteria	p__Bacillota_A	c__Clostridia	o__Christensenellales	f__CAG-138	g__PeH17	s__PeH17 sp000435055
PD	PD22	bin56	MAG1326	d__Bacteria	p__Bacillota_A	c__Clostridia	o__Christensenellales	f__CAG-138	g__PeH17	s__PeH17 sp000435055
PD	PD64	bin7	MAG1769	d__Bacteria	p__Bacillota_A	c__Clostridia	o__Christensenellales	f__CAG-138	g__PeH17	s__PeH17 sp000435055
PD	PD22	bin36	MAG242	d__Bacteria	p__Bacillota_A	c__Clostridia	o__Christensenellales	f__CAG-74	g__SFHK01	s__SFHK01 sp004556395
PD	PD57	bin3	MAG2140	d__Bacteria	p__Bacillota_A	c__Clostridia	o__Christensenellales	f__CAG-138	g__UBA1685	s__UBA1685 sp002320595
PD	PD64	bin42	MAG1888	d__Bacteria	p__Bacillota_A	c__Clostridia	o__Christensenellales	f__CAG-74	g__Faecivicinus	s__Faecivicinus sp934663595
PD	PD14	bin15	MAG67	d__Bacteria	p__Bacillota_A	c__Clostridia	o__Christensenellales	f__CAG-74	g__Ventricola	s__Ventricola sp900542445
PD	PD43	bin39	MAG1545	d__Bacteria	p__Bacillota_A	c__Clostridia	o__Christensenellales	f__Christensenellaceae	g__Christensenella	s__Christensenella hongkongensis
PD	PD97	bin67	MAG2412	d__Bacteria	p__Bacillota_A	c__Clostridia	o__Christensenellales	f__Christensenellaceae	g__Christensenella	s__Christensenella hongkongensis
PD	PD56	bin61	MAG377	d__Bacteria	p__Bacillota_A	c__Clostridia	o__Christensenellales	f__CAG-74	g__Fimadaptatus	s__Fimadaptatus sp900553645
PD	PD73	bin78	MAG3444	d__Bacteria	p__Bacillota_A	c__Clostridia	o__Christensenellales	f__CAG-74	g__Fimadaptatus	s__Fimadaptatus sp900553645
PD	PD57	bin57	MAG813	d__Bacteria	p__Bacillota_A	c__Clostridia	o__Christensenellales	f__CAG-74	g__Fimadaptatus	s__Fimadaptatus sp900553645
PD	PD12	bin54	MAG227	d__Bacteria	p__Bacillota_A	c__Clostridia	o__Christensenellales	f__CAG-74	g__UBA11524	s__UBA11524 sp000437595
PD	PD2	bin1	MAG1842	d__Bacteria	p__Bacillota_A	c__Clostridia	o__Christensenellales	f__CAG-74	g__UBA11524	s__UBA11524 sp000437595
PD	PD27	bin10	MAG3419	d__Bacteria	p__Bacillota_A	c__Clostridia	o__Christensenellales	f__CAG-74	g__UBA11524	s__UBA11524 sp000437595
PD	PD28	bin8	MAG1197	d__Bacteria	p__Bacillota_A	c__Clostridia	o__Christensenellales	f__CAG-74	g__UBA11524	s__UBA11524 sp000437595
PD	PD32	bin33	MAG387	d__Bacteria	p__Bacillota_A	c__Clostridia	o__Christensenellales	f__CAG-74	g__UBA11524	s__UBA11524 sp000437595
PD	PD66	bin7	MAG222	d__Bacteria	p__Bacillota_A	c__Clostridia	o__Christensenellales	f__CAG-74	g__UBA11524	s__UBA11524 sp000437595
PD	PD97	bin73	MAG945	d__Bacteria	p__Bacillota_A	c__Clostridia	o__Christensenellales	f__CAG-74	g__UBA11524	s__UBA11524 sp000437595
PD	PD13	bin65	MAG3220	d__Bacteria	p__Bacillota_A	c__Clostridia	o__Christensenellales	f__CAG-74	g__UBA11524	s__UBA11524 sp000437595
PD	PD14	bin10	MAG1332	d__Bacteria	p__Bacillota_A	c__Clostridia	o__Christensenellales	f__CAG-74	g__UBA11524	s__UBA11524 sp000437595
PD	PD46	bin1	MAG797	d__Bacteria	p__Bacillota_A	c__Clostridia	o__Christensenellales	f__CAG-74	g__UBA11524	s__UBA11524 sp000437595

PD	PD62	bin16	MAG3139	d__Bacteria	p__Bacillota_A	c__Clostridia	o__Christensenellales	f__CAG-74	g__UBA11524	s__UBA11524 sp000437595
PD	PD25	bin34	MAG1651	d__Bacteria	p__Bacillota_A	c__Clostridia	o__Christensenellales	f__CAG-74	g__UBA11524	s__UBA11524 sp000437595
PD	PD71	bin42	MAG624	d__Bacteria	p__Bacillota_A	c__Clostridia	o__Christensenellales	f__CAG-74	g__UBA11524	s__UBA11524 sp000437595
PD	PD69	bin21	MAG708	d__Bacteria	p__Bacillota_A	c__Clostridia	o__Christensenellales	f__CAG-74	g__UBA11524	s__UBA11524 sp000437595
PD	PD70	bin13	MAG3145	d__Bacteria	p__Bacillota_A	c__Clostridia	o__Christensenellales	f__CAG-74	g__UBA11524	s__UBA11524 sp000437595
PD	PD94	bin28	MAG1660	d__Bacteria	p__Bacillota_A	c__Clostridia	o__Christensenellales	f__CAG-74	g__UBA11524	s__UBA11524 sp000437595
PD	PD9	bin41	MAG471	d__Bacteria	p__Bacillota_A	c__Clostridia	o__Christensenellales	f__CAG-74	g__UBA11524	s__UBA11524 sp000437595
PD	PD5	bin38	MAG1526	d__Bacteria	p__Bacillota_A	c__Clostridia	o__Christensenellales	f__CAG-74	g__UBA11524	s__UBA11524 sp000437595
PD	PD61	bin10	MAG844	d__Bacteria	p__Bacillota_A	c__Clostridia	o__Christensenellales	f__CAG-74	g__UBA11524	s__UBA11524 sp000437595
PD	PD31	bin56	MAG2474	d__Bacteria	p__Bacillota_A	c__Clostridia	o__Christensenellales	f__CAG-74	g__UBA11524	s__UBA11524 sp000437595
PD	PD41	bin21	MAG3002	d__Bacteria	p__Bacillota_A	c__Clostridia	o__Christensenellales	f__CAG-74	g__UBA11524	s__UBA11524 sp000437595
PD	PD64	bin36	MAG3063	d__Bacteria	p__Bacillota_A	c__Clostridia	o__Christensenellales	f__CAG-74	g__Egerieenecus	s__Egerieenecus merdigallinarum
PD	PD2	bin35	MAG2316	d__Bacteria	p__Bacillota_A	c__Clostridia	o__Christensenellales	f__CAG-138	g__SFEL01	s__SFEL01 sp004557245
PD	PD29	bin15	MAG1537	d__Bacteria	p__Bacillota_A	c__Clostridia	o__Christensenellales	f__CAG-138	g__SFEL01	s__SFEL01 sp004557245
PD	PD72	bin34	MAG2488	d__Bacteria	p__Bacillota_A	c__Clostridia	o__Christensenellales	f__CAG-138	g__SFEL01	s__SFEL01 sp004557245
PD	PD41	bin48	MAG1285	d__Bacteria	p__Bacillota_A	c__Clostridia	o__Christensenellales	f__CAG-138	g__SFEL01	s__SFEL01 sp004557245
PD	PD34	bin45	MAG1415	d__Bacteria	p__Bacillota_A	c__Clostridia	o__Christensenellales	f__CAG-138	g__SFEL01	s__SFEL01 sp004557245
PD	PD7	bin5	MAG2125	d__Bacteria	p__Bacillota_A	c__Clostridia	o__Christensenellales	f__CAG-138	g__SFEL01	s__SFEL01 sp004557245
PD	PD22	bin22	MAG1218	d__Bacteria	p__Bacillota_A	c__Clostridia	o__Christensenellales	f__CAG-138	g__SFEL01	s__SFEL01 sp004557245
PD	PD59	bin57	MAG1408	d__Bacteria	p__Bacillota_A	c__Clostridia	o__Christensenellales	f__Christensenellaceae	g__Christensenella	s__Christensenella intestinihominis
PD	PD50	bin19	MAG1607	d__Bacteria	p__Bacillota_A	c__Clostridia	o__Christensenellales	f__CAG-74	g__SFMI01	s__SFMI01 sp004556155
PD	PD70	bin5	MAG164	d__Bacteria	p__Bacillota_A	c__Clostridia	o__Christensenellales	f__CAG-74	g__Limiplasma	s__Limiplasma sp004556545
PD	PD72	bin9	MAG1395	d__Bacteria	p__Bacillota_A	c__Clostridia	o__Christensenellales	f__CAG-74	g__Ventricola	s__Ventricola sp900542395
PD	PD76	bin14	MAG1474	d__Bacteria	p__Bacillota_A	c__Clostridia	o__Christensenellales	f__CAG-74	g__Ventricola	s__Ventricola sp900542395
PD	PD39	bin52	MAG2892	d__Bacteria	p__Bacillota_A	c__Clostridia	o__Christensenellales	f__CAG-74	g__Ventricola	s__Ventricola sp900542395
PD	PD43	bin23	MAG2148	d__Bacteria	p__Bacillota_A	c__Clostridia	o__Christensenellales	f__CAG-74	g__Ventricola	s__Ventricola sp900542395
PD	PD25	bin5	MAG205	d__Bacteria	p__Bacillota_A	c__Clostridia	o__Christensenellales	f__CAG-74	g__Ventricola	s__Ventricola sp900542395
PD	PD73	bin21	MAG1496	d__Bacteria	p__Bacillota_A	c__Clostridia	o__Christensenellales	f__CAG-74	g__Ventricola	s__Ventricola sp900542395
PD	PD51	bin35	MAG2287	d__Bacteria	p__Bacillota_A	c__Clostridia	o__Christensenellales	f__CAG-74	g__Limiplasma	s__Limiplasma sp900548145
PD	PD57	bin50	MAG169	d__Bacteria	p__Bacillota_A	c__Clostridia	o__Christensenellales	f__CAG-74	g__Limiplasma	s__Limiplasma sp900548145
PD	PD12	bin42	MAG776	d__Bacteria	p__Bacillota_A	c__Clostridia	o__Christensenellales	f__CAG-74	g__Limiplasma	s__Limiplasma sp900548145
PD	PD98	bin4	MAG1428	d__Bacteria	p__Bacillota_A	c__Clostridia	o__Christensenellales	f__CAG-74	g__Ventricola	s__Ventricola sp900548125
PD	PD56	bin57	MAG2833	d__Bacteria	p__Bacillota_A	c__Clostridia	o__Christensenellales	f__CAG-74	g__Ventricola	s__Ventricola sp900548125
PD	PD16	bin26	MAG2928	d__Bacteria	p__Bacillota_A	c__Clostridia	o__Christensenellales	f__QALW01	g__QALW01	s__QALW01 sp900552055
PD	PD56	bin27	MAG2070	d__Bacteria	p__Bacillota_A	c__Clostridia	o__Clostridiales	f__Clostridiaceae	g__Clostridium	s__Clostridium saudiense
PD	PD76	bin6	MAG443	d__Bacteria	p__Bacillota_A	c__Clostridia	o__Clostridiales	f__Clostridiaceae	g__Clostridium	s__Clostridium sp900540255
PD	PD19	bin24	MAG175	d__Bacteria	p__Bacillota_A	c__Clostridia	o__Clostridiales	f__Clostridiaceae	g__Clostridium	s__Clostridium sp900540255
PD	PD20	bin33	MAG1200	d__Bacteria	p__Bacillota_A	c__Clostridia	o__Clostridiales	f__Clostridiaceae	g__Clostridium	s__Clostridium sp900540255
PD	PD44	bin8	MAG3225	d__Bacteria	p__Bacillota_A	c__Clostridia	o__Clostridiales	f__Clostridiaceae	g__Clostridium	s__Clostridium sp001916075
PD	PD12	bin29	MAG2966	d__Bacteria	p__Bacillota_A	c__Clostridia	o__Lachnospirales	f__Lachnospiraceae	g__CAG-632	s__CAG-632 sp000431515
PD	PD12	bin66	MAG1726	d__Bacteria	p__Bacillota_A	c__Clostridia	o__Lachnospirales	f__Lachnospiraceae	g__Eubacterium_G	s__Eubacterium_G sp000432355
PD	PD75	bin20	MAG420	d__Bacteria	p__Bacillota_A	c__Clostridia	o__Lachnospirales	f__Lachnospiraceae	g__Schaedlerella	s__Schaedlerella sp900066545
PD	PD40	bin38	MAG184	d__Bacteria	p__Bacillota_A	c__Clostridia	o__Lachnospirales	f__Lachnospiraceae	g__Faecalimonas	s__Faecalimonas phoceensis
PD	PD20	bin29	MAG2271	d__Bacteria	p__Bacillota_A	c__Clostridia	o__Lachnospirales	f__Lachnospiraceae	g__Faecalimonas	s__Faecalimonas phoceensis
PD	PD62	bin46	MAG2063	d__Bacteria	p__Bacillota_A	c__Clostridia	o__Lachnospirales	f__Lachnospiraceae	g__Roseburia	s__Roseburia sp019411365
PD	PD92	bin37	MAG2722	d__Bacteria	p__Bacillota_A	c__Clostridia	o__Lachnospirales	f__Lachnospiraceae	g__Lachnoclostridium_B	s__Lachnoclostridium_B monacensis
PD	PD46	bin18	MAG2241	d__Bacteria	p__Bacillota_A	c__Clostridia	o__Lachnospirales	f__Lachnospiraceae	g__Roseburia	s__Roseburia intestinalis
PD	PD32	bin62	MAG1259	d__Bacteria	p__Bacillota_A	c__Clostridia	o__Lachnospirales	f__Lachnospiraceae	g__Roseburia	s__Roseburia intestinalis
PD	PD18	bin6	MAG541	d__Bacteria	p__Bacillota_A	c__Clostridia	o__Lachnospirales	f__Lachnospiraceae	g__Roseburia	s__Roseburia intestinalis
PD	PD29	bin11	MAG414	d__Bacteria	p__Bacillota_A	c__Clostridia	o__Lachnospirales	f__Lachnospiraceae	g__Roseburia	s__Roseburia intestinalis
PD	PD71	bin58	MAG2627	d__Bacteria	p__Bacillota_A	c__Clostridia	o__Lachnospirales	f__Lachnospiraceae	g__Roseburia	s__Roseburia intestinalis

PD	PD43	bin16	MAG390	d__Bacteria	p__Bacillota_A	c__Clostridia	o__Lachnospirales	f__Lachnospiraceae	g__Roseburia	s__Roseburia intestinalis
PD	PD24	bin25	MAG2942	d__Bacteria	p__Bacillota_A	c__Clostridia	o__Lachnospirales	f__Lachnospiraceae	g__Roseburia	s__Roseburia intestinalis
PD	PD77	bin4	MAG1763	d__Bacteria	p__Bacillota_A	c__Clostridia	o__Lachnospirales	f__Lachnospiraceae	g__Roseburia	s__Roseburia intestinalis
PD	PD19	bin15	MAG1023	d__Bacteria	p__Bacillota_A	c__Clostridia	o__Lachnospirales	f__Lachnospiraceae	g__Roseburia	s__Roseburia intestinalis
PD	PD1	bin31	MAG2965	d__Bacteria	p__Bacillota_A	c__Clostridia	o__Lachnospirales	f__Lachnospiraceae	g__Hungatella	s__Hungatella effluvii
PD	PD81	bin13	MAG766	d__Bacteria	p__Bacillota_A	c__Clostridia	o__Lachnospirales	f__Lachnospiraceae	g__Anaerobutyricum	s__Anaerobutyricum soehngenii
PD	PD40	bin48	MAG1799	d__Bacteria	p__Bacillota_A	c__Clostridia	o__Lachnospirales	f__Lachnospiraceae	g__Anaerobutyricum	s__Anaerobutyricum soehngenii
PD	PD98	bin35	MAG2974	d__Bacteria	p__Bacillota_A	c__Clostridia	o__Lachnospirales	f__Lachnospiraceae	g__Anaerobutyricum	s__Anaerobutyricum soehngenii
PD	PD87	bin63	MAG395	d__Bacteria	p__Bacillota_A	c__Clostridia	o__Lachnospirales	f__Lachnospiraceae	g__Anaerobutyricum	s__Anaerobutyricum soehngenii
PD	PD48	bin69	MAG2064	d__Bacteria	p__Bacillota_A	c__Clostridia	o__Lachnospirales	f__Lachnospiraceae	g__Anaerobutyricum	s__Anaerobutyricum soehngenii
PD	PD42	bin13	MAG3280	d__Bacteria	p__Bacillota_A	c__Clostridia	o__Lachnospirales	f__Lachnospiraceae	g__Anaerobutyricum	s__Anaerobutyricum soehngenii
PD	PD50	bin20	MAG3211	d__Bacteria	p__Bacillota_A	c__Clostridia	o__Lachnospirales	f__Lachnospiraceae	g__Anaerobutyricum	s__Anaerobutyricum soehngenii
PD	PD21	bin5	MAG2078	d__Bacteria	p__Bacillota_A	c__Clostridia	o__Lachnospirales	f__Lachnospiraceae	g__Clostridium_Q	s__Clostridium_Q sp000435655
PD	PD98	bin51	MAG380	d__Bacteria	p__Bacillota_A	c__Clostridia	o__Lachnospirales	f__Lachnospiraceae	g__Anaerostipes	s__Anaerostipes hadrus_A
PD	PD69	bin33	MAG2340	d__Bacteria	p__Bacillota_A	c__Clostridia	o__Lachnospirales	f__Lachnospiraceae	g__Anaerostipes	s__Anaerostipes hadrus_A
PD	PD9	bin67	MAG2129	d__Bacteria	p__Bacillota_A	c__Clostridia	o__Lachnospirales	f__Lachnospiraceae	g__Agathobacter	s__Agathobacter rectalis
PD	PD31	bin11	MAG2603	d__Bacteria	p__Bacillota_A	c__Clostridia	o__Lachnospirales	f__Lachnospiraceae	g__Agathobacter	s__Agathobacter rectalis
PD	PD47	bin14	MAG1867	d__Bacteria	p__Bacillota_A	c__Clostridia	o__Lachnospirales	f__Lachnospiraceae	g__Agathobacter	s__Agathobacter rectalis
PD	PD5	bin27	MAG2553	d__Bacteria	p__Bacillota_A	c__Clostridia	o__Lachnospirales	f__Lachnospiraceae	g__Agathobacter	s__Agathobacter rectalis
PD	PD71	bin24	MAG2708	d__Bacteria	p__Bacillota_A	c__Clostridia	o__Lachnospirales	f__Lachnospiraceae	g__Agathobacter	s__Agathobacter rectalis
PD	PD77	bin40	MAG1047	d__Bacteria	p__Bacillota_A	c__Clostridia	o__Lachnospirales	f__Lachnospiraceae	g__Agathobacter	s__Agathobacter rectalis
PD	PD85	bin6	MAG1392	d__Bacteria	p__Bacillota_A	c__Clostridia	o__Lachnospirales	f__Lachnospiraceae	g__Agathobacter	s__Agathobacter rectalis
PD	PD8	bin5	MAG872	d__Bacteria	p__Bacillota_A	c__Clostridia	o__Lachnospirales	f__Lachnospiraceae	g__Agathobacter	s__Agathobacter rectalis
PD	PD99	bin16	MAG2766	d__Bacteria	p__Bacillota_A	c__Clostridia	o__Lachnospirales	f__Lachnospiraceae	g__Agathobacter	s__Agathobacter rectalis
PD	PD35	bin5	MAG2196	d__Bacteria	p__Bacillota_A	c__Clostridia	o__Lachnospirales	f__Lachnospiraceae	g__Agathobacter	s__Agathobacter rectalis
PD	PD91	bin3	MAG654	d__Bacteria	p__Bacillota_A	c__Clostridia	o__Lachnospirales	f__Lachnospiraceae	g__Agathobacter	s__Agathobacter rectalis
PD	PD98	bin47	MAG7	d__Bacteria	p__Bacillota_A	c__Clostridia	o__Lachnospirales	f__Lachnospiraceae	g__Agathobacter	s__Agathobacter rectalis
PD	PD13	bin54	MAG2306	d__Bacteria	p__Bacillota_A	c__Clostridia	o__Lachnospirales	f__Lachnospiraceae	g__Agathobacter	s__Agathobacter rectalis
PD	PD40	bin49	MAG1872	d__Bacteria	p__Bacillota_A	c__Clostridia	o__Lachnospirales	f__Lachnospiraceae	g__Agathobacter	s__Agathobacter rectalis
PD	PD69	bin25	MAG2450	d__Bacteria	p__Bacillota_A	c__Clostridia	o__Lachnospirales	f__Lachnospiraceae	g__Agathobacter	s__Agathobacter rectalis
PD	PD20	bin23	MAG195	d__Bacteria	p__Bacillota_A	c__Clostridia	o__Lachnospirales	f__Lachnospiraceae	g__Agathobacter	s__Agathobacter rectalis
PD	PD68	bin57	MAG1777	d__Bacteria	p__Bacillota_A	c__Clostridia	o__Lachnospirales	f__Lachnospiraceae	g__Agathobacter	s__Agathobacter rectalis
PD	PD25	bin6	MAG123	d__Bacteria	p__Bacillota_A	c__Clostridia	o__Lachnospirales	f__Lachnospiraceae	g__Agathobacter	s__Agathobacter rectalis
PD	PD19	bin13	MAG2160	d__Bacteria	p__Bacillota_A	c__Clostridia	o__Lachnospirales	f__Lachnospiraceae	g__Agathobacter	s__Agathobacter rectalis
PD	PD53	bin34	MAG1012	d__Bacteria	p__Bacillota_A	c__Clostridia	o__Lachnospirales	f__Lachnospiraceae	g__Agathobacter	s__Agathobacter rectalis
PD	PD82	bin53	MAG946	d__Bacteria	p__Bacillota_A	c__Clostridia	o__Lachnospirales	f__Lachnospiraceae	g__Agathobacter	s__Agathobacter rectalis
PD	PD58	bin12	MAG2102	d__Bacteria	p__Bacillota_A	c__Clostridia	o__Lachnospirales	f__Lachnospiraceae	g__Agathobacter	s__Agathobacter rectalis
PD	PD44	bin59	MAG2109	d__Bacteria	p__Bacillota_A	c__Clostridia	o__Lachnospirales	f__Lachnospiraceae	g__Agathobacter	s__Agathobacter rectalis
PD	PD59	bin18	MAG3067	d__Bacteria	p__Bacillota_A	c__Clostridia	o__Lachnospirales	f__Lachnospiraceae	g__Agathobacter	s__Agathobacter rectalis
PD	PD43	bin52	MAG971	d__Bacteria	p__Bacillota_A	c__Clostridia	o__Lachnospirales	f__Lachnospiraceae	g__Agathobacter	s__Agathobacter rectalis
PD	PD70	bin48	MAG767	d__Bacteria	p__Bacillota_A	c__Clostridia	o__Lachnospirales	f__Lachnospiraceae	g__Agathobacter	s__Agathobacter rectalis
PD	PD2	bin40	MAG1970	d__Bacteria	p__Bacillota_A	c__Clostridia	o__Lachnospirales	f__Lachnospiraceae	g__Agathobacter	s__Agathobacter rectalis
PD	PD83	bin30	MAG801	d__Bacteria	p__Bacillota_A	c__Clostridia	o__Lachnospirales	f__Lachnospiraceae	g__Agathobacter	s__Agathobacter rectalis
PD	PD57	bin55	MAG1383	d__Bacteria	p__Bacillota_A	c__Clostridia	o__Lachnospirales	f__Lachnospiraceae	g__Agathobacter	s__Agathobacter rectalis
PD	PD37	bin32	MAG1596	d__Bacteria	p__Bacillota_A	c__Clostridia	o__Lachnospirales	f__Lachnospiraceae	g__Agathobacter	s__Agathobacter rectalis
PD	PD73	bin31	MAG1030	d__Bacteria	p__Bacillota_A	c__Clostridia	o__Lachnospirales	f__Lachnospiraceae	g__Agathobacter	s__Agathobacter rectalis
PD	PD97	bin68	MAG612	d__Bacteria	p__Bacillota_A	c__Clostridia	o__Lachnospirales	f__Lachnospiraceae	g__Agathobacter	s__Agathobacter rectalis
PD	PD34	bin64	MAG1479	d__Bacteria	p__Bacillota_A	c__Clostridia	o__Lachnospirales	f__Lachnospiraceae	g__Agathobacter	s__Agathobacter rectalis
PD	PD64	bin50	MAG268	d__Bacteria	p__Bacillota_A	c__Clostridia	o__Lachnospirales	f__Lachnospiraceae	g__Blautia_A	s__Blautia_A sp900066145
PD	PD28	bin50	MAG2163	d__Bacteria	p__Bacillota_A	c__Clostridia	o__Lachnospirales	f__Lachnospiraceae	g__Acetatifactor	s__Acetatifactor intestinalis
PD	PD47	bin13	MAG2334	d__Bacteria	p__Bacillota_A	c__Clostridia	o__Lachnospirales	f__Lachnospiraceae	g__Acetatifactor	s__Acetatifactor intestinalis

PD	PD19	bin27	MAG291	d__Bacteria	p__Bacillota_A	c__Clostridia	o__Lachnospirales	f__Lachnospiraceae	g__Acetatifactor	s__Acetatifactor intestinalis
PD	PD5	bin24	MAG3333	d__Bacteria	p__Bacillota_A	c__Clostridia	o__Lachnospirales	f__Lachnospiraceae	g__Acetatifactor	s__Acetatifactor intestinalis
PD	PD12	bin28	MAG742	d__Bacteria	p__Bacillota_A	c__Clostridia	o__Lachnospirales	f__Lachnospiraceae	g__Acetatifactor	s__Acetatifactor intestinalis
PD	PD24	bin13	MAG149	d__Bacteria	p__Bacillota_A	c__Clostridia	o__Lachnospirales	f__Lachnospiraceae	g__Acetatifactor	s__Acetatifactor intestinalis
PD	PD10	bin44	MAG3376	d__Bacteria	p__Bacillota_A	c__Clostridia	o__Lachnospirales	f__Lachnospiraceae	g__Acetatifactor	s__Acetatifactor intestinalis
PD	PD61	bin25	MAG1361	d__Bacteria	p__Bacillota_A	c__Clostridia	o__Lachnospirales	f__Lachnospiraceae	g__Acetatifactor	s__Acetatifactor intestinalis
PD	PD31	bin61	MAG3108	d__Bacteria	p__Bacillota_A	c__Clostridia	o__Lachnospirales	f__Lachnospiraceae	g__Acetatifactor	s__Acetatifactor intestinalis
PD	PD14	bin45	MAG1195	d__Bacteria	p__Bacillota_A	c__Clostridia	o__Lachnospirales	f__Lachnospiraceae	g__Acetatifactor	s__Acetatifactor intestinalis
PD	PD45	bin10	MAG1322	d__Bacteria	p__Bacillota_A	c__Clostridia	o__Lachnospirales	f__Lachnospiraceae	g__Roseburia	s__Roseburia inulinivorans
PD	PD3	bin13	MAG3018	d__Bacteria	p__Bacillota_A	c__Clostridia	o__Lachnospirales	f__Lachnospiraceae	g__Roseburia	s__Roseburia inulinivorans
PD	PD71	bin36	MAG604	d__Bacteria	p__Bacillota_A	c__Clostridia	o__Lachnospirales	f__Lachnospiraceae	g__Roseburia	s__Roseburia inulinivorans
PD	PD28	bin37	MAG660	d__Bacteria	p__Bacillota_A	c__Clostridia	o__Lachnospirales	f__Lachnospiraceae	g__Roseburia	s__Roseburia inulinivorans
PD	PD23	bin43	MAG1027	d__Bacteria	p__Bacillota_A	c__Clostridia	o__Lachnospirales	f__Lachnospiraceae	g__Roseburia	s__Roseburia inulinivorans
PD	PD64	bin2	MAG1499	d__Bacteria	p__Bacillota_A	c__Clostridia	o__Lachnospirales	f__Lachnospiraceae	g__Roseburia	s__Roseburia inulinivorans
PD	PD32	bin35	MAG2618	d__Bacteria	p__Bacillota_A	c__Clostridia	o__Lachnospirales	f__Lachnospiraceae	g__Roseburia	s__Roseburia inulinivorans
PD	PD78	bin14	MAG525	d__Bacteria	p__Bacillota_A	c__Clostridia	o__Lachnospirales	f__Lachnospiraceae	g__Roseburia	s__Roseburia inulinivorans
PD	PD5	bin18	MAG3216	d__Bacteria	p__Bacillota_A	c__Clostridia	o__Lachnospirales	f__Lachnospiraceae	g__Roseburia	s__Roseburia inulinivorans
PD	PD87	bin4	MAG3003	d__Bacteria	p__Bacillota_A	c__Clostridia	o__Lachnospirales	f__Lachnospiraceae	g__Eubacterium_I	s__Eubacterium_I ramulus_A
PD	PD75	bin16	MAG1619	d__Bacteria	p__Bacillota_A	c__Clostridia	o__Lachnospirales	f__Lachnospiraceae	g__Eubacterium_I	s__Eubacterium_I ramulus_A
PD	PD9	bin59	MAG2388	d__Bacteria	p__Bacillota_A	c__Clostridia	o__Lachnospirales	f__Lachnospiraceae	g__Roseburia	s__Roseburia hominis
PD	PD64	bin32	MAG731	d__Bacteria	p__Bacillota_A	c__Clostridia	o__Lachnospirales	f__Lachnospiraceae	g__Roseburia	s__Roseburia hominis
PD	PD48	bin55	MAG2700	d__Bacteria	p__Bacillota_A	c__Clostridia	o__Lachnospirales	f__Lachnospiraceae	g__Roseburia	s__Roseburia hominis
PD	PD28	bin41	MAG321	d__Bacteria	p__Bacillota_A	c__Clostridia	o__Lachnospirales	f__Lachnospiraceae	g__Roseburia	s__Roseburia hominis
PD	PD68	bin31	MAG2274	d__Bacteria	p__Bacillota_A	c__Clostridia	o__Lachnospirales	f__Lachnospiraceae	g__Roseburia	s__Roseburia hominis
PD	PD39	bin40	MAG1494	d__Bacteria	p__Bacillota_A	c__Clostridia	o__Lachnospirales	f__Lachnospiraceae	g__Roseburia	s__Roseburia hominis
PD	PD61	bin58	MAG737	d__Bacteria	p__Bacillota_A	c__Clostridia	o__Lachnospirales	f__Lachnospiraceae	g__Roseburia	s__Roseburia hominis
PD	PD97	bin51	MAG3474	d__Bacteria	p__Bacillota_A	c__Clostridia	o__Lachnospirales	f__Lachnospiraceae	g__Roseburia	s__Roseburia hominis
PD	PD24	bin43	MAG1509	d__Bacteria	p__Bacillota_A	c__Clostridia	o__Lachnospirales	f__Lachnospiraceae	g__Roseburia	s__Roseburia hominis
PD	PD12	bin33	MAG2008	d__Bacteria	p__Bacillota_A	c__Clostridia	o__Lachnospirales	f__Lachnospiraceae	g__Roseburia	s__Roseburia hominis
PD	PD29	bin47	MAG1625	d__Bacteria	p__Bacillota_A	c__Clostridia	o__Lachnospirales	f__Lachnospiraceae	g__Roseburia	s__Roseburia hominis
PD	PD58	bin35	MAG1491	d__Bacteria	p__Bacillota_A	c__Clostridia	o__Lachnospirales	f__Lachnospiraceae	g__Roseburia	s__Roseburia hominis
PD	PD14	bin18	MAG429	d__Bacteria	p__Bacillota_A	c__Clostridia	o__Lachnospirales	f__Lachnospiraceae	g__Roseburia	s__Roseburia hominis
PD	PD100	bin25	MAG220	d__Bacteria	p__Bacillota_A	c__Clostridia	o__Lachnospirales	f__Lachnospiraceae	g__Dorea_A	s__Dorea_A longicatena_B
PD	PD4	bin22	MAG3458	d__Bacteria	p__Bacillota_A	c__Clostridia	o__Lachnospirales	f__Lachnospiraceae	g__Dorea_A	s__Dorea_A longicatena_B
PD	PD58	bin62	MAG2721	d__Bacteria	p__Bacillota_A	c__Clostridia	o__Lachnospirales	f__Lachnospiraceae	g__Dorea_A	s__Dorea_A longicatena_B
PD	PD35	bin45	MAG902	d__Bacteria	p__Bacillota_A	c__Clostridia	o__Lachnospirales	f__Lachnospiraceae	g__Dorea_A	s__Dorea_A longicatena_B
PD	PD81	bin8	MAG237	d__Bacteria	p__Bacillota_A	c__Clostridia	o__Lachnospirales	f__Lachnospiraceae	g__Dorea_A	s__Dorea_A longicatena_B
PD	PD44	bin33	MAG879	d__Bacteria	p__Bacillota_A	c__Clostridia	o__Lachnospirales	f__Lachnospiraceae	g__Dorea_A	s__Dorea_A longicatena_B
PD	PD25	bin12	MAG2027	d__Bacteria	p__Bacillota_A	c__Clostridia	o__Lachnospirales	f__Lachnospiraceae	g__Dorea_A	s__Dorea_A longicatena_B
PD	PD95	bin6	MAG1041	d__Bacteria	p__Bacillota_A	c__Clostridia	o__Lachnospirales	f__Lachnospiraceae	g__Dorea_A	s__Dorea_A longicatena_B
PD	PD99	bin38	MAG3502	d__Bacteria	p__Bacillota_A	c__Clostridia	o__Lachnospirales	f__Lachnospiraceae	g__Dorea_A	s__Dorea_A longicatena_B
PD	PD98	bin3	MAG786	d__Bacteria	p__Bacillota_A	c__Clostridia	o__Lachnospirales	f__Lachnospiraceae	g__Dorea_A	s__Dorea_A longicatena_B
PD	PD47	bin12	MAG3472	d__Bacteria	p__Bacillota_A	c__Clostridia	o__Lachnospirales	f__Lachnospiraceae	g__Dorea_A	s__Dorea_A longicatena_B
PD	PD75	bin8	MAG2851	d__Bacteria	p__Bacillota_A	c__Clostridia	o__Lachnospirales	f__Lachnospiraceae	g__Dorea_A	s__Dorea_A longicatena_B
PD	PD75	bin21	MAG2353	d__Bacteria	p__Bacillota_A	c__Clostridia	o__Lachnospirales	f__Lachnospiraceae	g__Dorea_A	s__Dorea_A longicatena_B
PD	PD75	bin26	MAG3343	d__Bacteria	p__Bacillota_A	c__Clostridia	o__Lachnospirales	f__Anaerotignaceae	g__Metalachnospira	s__Unclassified
PD	PD95	bin1	MAG3371	d__Bacteria	p__Bacillota_A	c__Clostridia	o__Lachnospirales	f__Lachnospiraceae	g__Mediterraneibacter	s__Mediterraneibacter torques
PD	PD53	bin45	MAG1166	d__Bacteria	p__Bacillota_A	c__Clostridia	o__Lachnospirales	f__Lachnospiraceae	g__Mediterraneibacter	s__Mediterraneibacter torques
PD	PD75	bin29	MAG207	d__Bacteria	p__Bacillota_A	c__Clostridia	o__Lachnospirales	f__Lachnospiraceae	g__Mediterraneibacter	s__Mediterraneibacter torques
PD	PD60	bin28	MAG2294	d__Bacteria	p__Bacillota_A	c__Clostridia	o__Lachnospirales	f__Lachnospiraceae	g__Mediterraneibacter	s__Mediterraneibacter torques
PD	PD73	bin37	MAG2756	d__Bacteria	p__Bacillota_A	c__Clostridia	o__Lachnospirales	f__Lachnospiraceae	g__Mediterraneibacter	s__Mediterraneibacter torques

PD	PD44	bin2	MAG1908	d__Bacteria	p__Bacillota_A	c__Clostridia	o__Lachnospirales	f__Lachnospiraceae	g__Mediterraneibacter	s__Mediterraneibacter torques
PD	PD76	bin16	MAG1881	d__Bacteria	p__Bacillota_A	c__Clostridia	o__Lachnospirales	f__Lachnospiraceae	g__Mediterraneibacter	s__Mediterraneibacter torques
PD	PD97	bin24	MAG3128	d__Bacteria	p__Bacillota_A	c__Clostridia	o__Lachnospirales	f__Lachnospiraceae	g__Mediterraneibacter	s__Mediterraneibacter torques
PD	PD16	bin60	MAG1264	d__Bacteria	p__Bacillota_A	c__Clostridia	o__Lachnospirales	f__Lachnospiraceae	g__Mediterraneibacter	s__Mediterraneibacter torques
PD	PD13	bin32	MAG939	d__Bacteria	p__Bacillota_A	c__Clostridia	o__Lachnospirales	f__Lachnospiraceae	g__Anaerobutyricum	s__Anaerobutyricum hallii
PD	PD34	bin73	MAG1549	d__Bacteria	p__Bacillota_A	c__Clostridia	o__Lachnospirales	f__Lachnospiraceae	g__Anaerobutyricum	s__Anaerobutyricum hallii
PD	PD39	bin1	MAG1025	d__Bacteria	p__Bacillota_A	c__Clostridia	o__Lachnospirales	f__Lachnospiraceae	g__Anaerobutyricum	s__Anaerobutyricum hallii
PD	PD51	bin4	MAG90	d__Bacteria	p__Bacillota_A	c__Clostridia	o__Lachnospirales	f__Lachnospiraceae	g__Anaerobutyricum	s__Anaerobutyricum hallii
PD	PD64	bin83	MAG2060	d__Bacteria	p__Bacillota_A	c__Clostridia	o__Lachnospirales	f__Lachnospiraceae	g__Anaerobutyricum	s__Anaerobutyricum hallii
PD	PD77	bin31	MAG231	d__Bacteria	p__Bacillota_A	c__Clostridia	o__Lachnospirales	f__Lachnospiraceae	g__Anaerobutyricum	s__Anaerobutyricum hallii
PD	PD57	bin81	MAG793	d__Bacteria	p__Bacillota_A	c__Clostridia	o__Lachnospirales	f__Lachnospiraceae	g__Anaerobutyricum	s__Anaerobutyricum hallii
PD	PD69	bin49	MAG3039	d__Bacteria	p__Bacillota_A	c__Clostridia	o__Lachnospirales	f__Lachnospiraceae	g__Anaerobutyricum	s__Anaerobutyricum hallii
PD	PD66	bin31	MAG2613	d__Bacteria	p__Bacillota_A	c__Clostridia	o__Lachnospirales	f__Lachnospiraceae	g__Anaerobutyricum	s__Anaerobutyricum hallii
PD	PD16	bin31	MAG1669	d__Bacteria	p__Bacillota_A	c__Clostridia	o__Lachnospirales	f__Lachnospiraceae	g__Anaerobutyricum	s__Anaerobutyricum hallii
PD	PD72	bin18	MAG1235	d__Bacteria	p__Bacillota_A	c__Clostridia	o__Lachnospirales	f__Lachnospiraceae	g__Anaerobutyricum	s__Anaerobutyricum hallii
PD	PD87	bin66	MAG469	d__Bacteria	p__Bacillota_A	c__Clostridia	o__Lachnospirales	f__Lachnospiraceae	g__Anaerobutyricum	s__Anaerobutyricum hallii
PD	PD10	bin42	MAG1939	d__Bacteria	p__Bacillota_A	c__Clostridia	o__Lachnospirales	f__Lachnospiraceae	g__Anaerobutyricum	s__Anaerobutyricum hallii
PD	PD37	bin44	MAG1694	d__Bacteria	p__Bacillota_A	c__Clostridia	o__Lachnospirales	f__Lachnospiraceae	g__Anaerobutyricum	s__Anaerobutyricum hallii
PD	PD48	bin16	MAG831	d__Bacteria	p__Bacillota_A	c__Clostridia	o__Lachnospirales	f__Lachnospiraceae	g__Anaerobutyricum	s__Anaerobutyricum hallii
PD	PD82	bin51	MAG1767	d__Bacteria	p__Bacillota_A	c__Clostridia	o__Lachnospirales	f__Lachnospiraceae	g__Anaerobutyricum	s__Anaerobutyricum hallii
PD	PD99	bin30	MAG2107	d__Bacteria	p__Bacillota_A	c__Clostridia	o__Lachnospirales	f__Lachnospiraceae	g__Anaerobutyricum	s__Anaerobutyricum hallii
PD	PD26	bin19	MAG2398	d__Bacteria	p__Bacillota_A	c__Clostridia	o__Lachnospirales	f__Lachnospiraceae	g__Lachnospira	s__Lachnospira sp900545725
PD	PD35	bin44	MAG2158	d__Bacteria	p__Bacillota_A	c__Clostridia	o__Lachnospirales	f__Lachnospiraceae	g__Eubacterium_G	s__Eubacterium_G ventriosum
PD	PD45	bin28	MAG1735	d__Bacteria	p__Bacillota_A	c__Clostridia	o__Lachnospirales	f__Lachnospiraceae	g__Eubacterium_G	s__Eubacterium_G ventriosum
PD	PD61	bin4	MAG2561	d__Bacteria	p__Bacillota_A	c__Clostridia	o__Lachnospirales	f__Lachnospiraceae	g__Eubacterium_G	s__Eubacterium_G ventriosum
PD	PD78	bin1	MAG2891	d__Bacteria	p__Bacillota_A	c__Clostridia	o__Lachnospirales	f__Lachnospiraceae	g__Eubacterium_G	s__Eubacterium_G ventriosum
PD	PD82	bin39	MAG2698	d__Bacteria	p__Bacillota_A	c__Clostridia	o__Lachnospirales	f__Lachnospiraceae	g__Eubacterium_G	s__Eubacterium_G ventriosum
PD	PD81	bin17	MAG840	d__Bacteria	p__Bacillota_A	c__Clostridia	o__Lachnospirales	f__Lachnospiraceae	g__Eubacterium_G	s__Eubacterium_G ventriosum
PD	PD44	bin30	MAG2018	d__Bacteria	p__Bacillota_A	c__Clostridia	o__Lachnospirales	f__Lachnospiraceae	g__Eubacterium_G	s__Eubacterium_G ventriosum
PD	PD70	bin16	MAG2927	d__Bacteria	p__Bacillota_A	c__Clostridia	o__Lachnospirales	f__Lachnospiraceae	g__Eubacterium_G	s__Eubacterium_G ventriosum
PD	PD34	bin46	MAG1554	d__Bacteria	p__Bacillota_A	c__Clostridia	o__Lachnospirales	f__Lachnospiraceae	g__Eubacterium_G	s__Eubacterium_G ventriosum
PD	PD62	bin19	MAG2537	d__Bacteria	p__Bacillota_A	c__Clostridia	o__Lachnospirales	f__Lachnospiraceae	g__Eubacterium_G	s__Eubacterium_G ventriosum
PD	PD95	bin14	MAG901	d__Bacteria	p__Bacillota_A	c__Clostridia	o__Lachnospirales	f__Lachnospiraceae	g__Eubacterium_G	s__Eubacterium_G ventriosum
PD	PD40	bin6	MAG2410	d__Bacteria	p__Bacillota_A	c__Clostridia	o__Lachnospirales	f__Lachnospiraceae	g__Eubacterium_G	s__Eubacterium_G ventriosum
PD	PD98	bin27	MAG1368	d__Bacteria	p__Bacillota_A	c__Clostridia	o__Lachnospirales	f__Lachnospiraceae	g__Eubacterium_G	s__Eubacterium_G ventriosum
PD	PD4	bin14	MAG1602	d__Bacteria	p__Bacillota_A	c__Clostridia	o__Lachnospirales	f__Lachnospiraceae	g__Eubacterium_G	s__Eubacterium_G sp000435815
PD	PD77	bin14	MAG1427	d__Bacteria	p__Bacillota_A	c__Clostridia	o__Lachnospirales	f__Lachnospiraceae	g__Eubacterium_G	s__Eubacterium_G sp000435815
PD	PD43	bin30	MAG2337	d__Bacteria	p__Bacillota_A	c__Clostridia	o__Lachnospirales	f__Lachnospiraceae	g__Anaerostipes	s__Anaerostipes hadrus
PD	PD44	bin37	MAG1400	d__Bacteria	p__Bacillota_A	c__Clostridia	o__Lachnospirales	f__Lachnospiraceae	g__Anaerostipes	s__Anaerostipes hadrus
PD	PD46	bin31	MAG1190	d__Bacteria	p__Bacillota_A	c__Clostridia	o__Lachnospirales	f__Lachnospiraceae	g__Anaerostipes	s__Anaerostipes hadrus
PD	PD48	bin34	MAG281	d__Bacteria	p__Bacillota_A	c__Clostridia	o__Lachnospirales	f__Lachnospiraceae	g__Anaerostipes	s__Anaerostipes hadrus
PD	PD70	bin24	MAG1092	d__Bacteria	p__Bacillota_A	c__Clostridia	o__Lachnospirales	f__Lachnospiraceae	g__Anaerostipes	s__Anaerostipes hadrus
PD	PD75	bin24	MAG1269	d__Bacteria	p__Bacillota_A	c__Clostridia	o__Lachnospirales	f__Lachnospiraceae	g__Anaerostipes	s__Anaerostipes hadrus
PD	PD76	bin10	MAG559	d__Bacteria	p__Bacillota_A	c__Clostridia	o__Lachnospirales	f__Lachnospiraceae	g__Anaerostipes	s__Anaerostipes hadrus
PD	PD10	bin26	MAG610	d__Bacteria	p__Bacillota_A	c__Clostridia	o__Lachnospirales	f__Lachnospiraceae	g__Anaerostipes	s__Anaerostipes hadrus
PD	PD77	bin20	MAG3307	d__Bacteria	p__Bacillota_A	c__Clostridia	o__Lachnospirales	f__Lachnospiraceae	g__Anaerostipes	s__Anaerostipes hadrus
PD	PD82	bin46	MAG1065	d__Bacteria	p__Bacillota_A	c__Clostridia	o__Lachnospirales	f__Lachnospiraceae	g__Anaerostipes	s__Anaerostipes hadrus
PD	PD37	bin8	MAG2326	d__Bacteria	p__Bacillota_A	c__Clostridia	o__Lachnospirales	f__Lachnospiraceae	g__Anaerostipes	s__Anaerostipes hadrus
PD	PD73	bin73	MAG1553	d__Bacteria	p__Bacillota_A	c__Clostridia	o__Lachnospirales	f__Lachnospiraceae	g__Anaerostipes	s__Anaerostipes hadrus
PD	PD67	bin12	MAG2684	d__Bacteria	p__Bacillota_A	c__Clostridia	o__Lachnospirales	f__Lachnospiraceae	g__Anaerostipes	s__Anaerostipes hadrus
PD	PD95	bin21	MAG172	d__Bacteria	p__Bacillota_A	c__Clostridia	o__Lachnospirales	f__Lachnospiraceae	g__Anaerostipes	s__Anaerostipes hadrus

PD	PD4	bin43	MAG2471	d__Bacteria	p__Bacillota_A	c__Clostridia	o__Lachnospirales	f__Lachnospiraceae	g__Anaerostipes	s__Anaerostipes hadrus
PD	PD42	bin27	MAG3116	d__Bacteria	p__Bacillota_A	c__Clostridia	o__Lachnospirales	f__Lachnospiraceae	g__Anaerostipes	s__Anaerostipes hadrus
PD	PD40	bin54	MAG3199	d__Bacteria	p__Bacillota_A	c__Clostridia	o__Lachnospirales	f__Lachnospiraceae	g__Anaerostipes	s__Anaerostipes hadrus
PD	PD72	bin29	MAG1571	d__Bacteria	p__Bacillota_A	c__Clostridia	o__Lachnospirales	f__Lachnospiraceae	g__Anaerostipes	s__Anaerostipes hadrus
PD	PD64	bin79	MAG2273	d__Bacteria	p__Bacillota_A	c__Clostridia	o__Lachnospirales	f__Lachnospiraceae	g__Anaerostipes	s__Anaerostipes hadrus
PD	PD50	bin62	MAG1315	d__Bacteria	p__Bacillota_A	c__Clostridia	o__Lachnospirales	f__Lachnospiraceae	g__Anaerostipes	s__Anaerostipes hadrus
PD	PD57	bin23	MAG2870	d__Bacteria	p__Bacillota_A	c__Clostridia	o__Lachnospirales	f__Lachnospiraceae	g__Anaerostipes	s__Anaerostipes hadrus
PD	PD47	bin52	MAG95	d__Bacteria	p__Bacillota_A	c__Clostridia	o__Lachnospirales	f__Lachnospiraceae	g__Anaerostipes	s__Anaerostipes hadrus
PD	PD8	bin59	MAG114	d__Bacteria	p__Bacillota_A	c__Clostridia	o__Lachnospirales	f__Lachnospiraceae	g__Anaerostipes	s__Anaerostipes hadrus
PD	PD56	bin7	MAG2675	d__Bacteria	p__Bacillota_A	c__Clostridia	o__Lachnospirales	f__Lachnospiraceae	g__Anaerostipes	s__Anaerostipes hadrus
PD	PD13	bin37	MAG544	d__Bacteria	p__Bacillota_A	c__Clostridia	o__Lachnospirales	f__Lachnospiraceae	g__Anaerostipes	s__Anaerostipes hadrus
PD	PD29	bin43	MAG3189	d__Bacteria	p__Bacillota_A	c__Clostridia	o__Lachnospirales	f__Lachnospiraceae	g__Anaerostipes	s__Anaerostipes hadrus
PD	PD58	bin16	MAG3161	d__Bacteria	p__Bacillota_A	c__Clostridia	o__Lachnospirales	f__Lachnospiraceae	g__Anaerostipes	s__Anaerostipes hadrus
PD	PD87	bin32	MAG753	d__Bacteria	p__Bacillota_A	c__Clostridia	o__Lachnospirales	f__Lachnospiraceae	g__Anaerostipes	s__Anaerostipes hadrus
PD	PD97	bin77	MAG3452	d__Bacteria	p__Bacillota_A	c__Clostridia	o__Lachnospirales	f__Lachnospiraceae	g__Anaerostipes	s__Anaerostipes hadrus
PD	PD100	bin23	MAG2909	d__Bacteria	p__Bacillota_A	c__Clostridia	o__Lachnospirales	f__Lachnospiraceae	g__Anaerostipes	s__Anaerostipes hadrus
PD	PD94	bin57	MAG3021	d__Bacteria	p__Bacillota_A	c__Clostridia	o__Lachnospirales	f__Lachnospiraceae	g__Anaerostipes	s__Anaerostipes hadrus
PD	PD32	bin72	MAG44	d__Bacteria	p__Bacillota_A	c__Clostridia	o__Lachnospirales	f__Lachnospiraceae	g__Anaerostipes	s__Anaerostipes hadrus
PD	PD2	bin63	MAG3035	d__Bacteria	p__Bacillota_A	c__Clostridia	o__Lachnospirales	f__Lachnospiraceae	g__Anaerostipes	s__Anaerostipes hadrus
PD	PD46	bin54	MAG619	d__Bacteria	p__Bacillota_A	c__Clostridia	o__Lachnospirales	f__Lachnospiraceae	g__Coprococcus	s__Coprococcus eutactus_A
PD	PD47	bin36	MAG914	d__Bacteria	p__Bacillota_A	c__Clostridia	o__Lachnospirales	f__Lachnospiraceae	g__Coprococcus	s__Coprococcus eutactus_A
PD	PD5	bin30	MAG1000	d__Bacteria	p__Bacillota_A	c__Clostridia	o__Lachnospirales	f__Lachnospiraceae	g__Coprococcus	s__Coprococcus eutactus_A
PD	PD62	bin27	MAG2171	d__Bacteria	p__Bacillota_A	c__Clostridia	o__Lachnospirales	f__Lachnospiraceae	g__Coprococcus	s__Coprococcus eutactus_A
PD	PD10	bin52	MAG1459	d__Bacteria	p__Bacillota_A	c__Clostridia	o__Lachnospirales	f__Lachnospiraceae	g__Coprococcus	s__Coprococcus eutactus_A
PD	PD61	bin34	MAG979	d__Bacteria	p__Bacillota_A	c__Clostridia	o__Lachnospirales	f__Lachnospiraceae	g__Coprococcus	s__Coprococcus eutactus_A
PD	PD81	bin5	MAG1304	d__Bacteria	p__Bacillota_A	c__Clostridia	o__Lachnospirales	f__Lachnospiraceae	g__Anaerostipes	s__Anaerostipes caccae
PD	PD94	bin46	MAG2688	d__Bacteria	p__Bacillota_A	c__Clostridia	o__Lachnospirales	f__Lachnospiraceae	g__Coprococcus	s__Coprococcus sp900066115
PD	PD9	bin45	MAG3158	d__Bacteria	p__Bacillota_A	c__Clostridia	o__Lachnospirales	f__Lachnospiraceae	g__Coprococcus	s__Coprococcus sp900066115
PD	PD70	bin59	MAG2995	d__Bacteria	p__Bacillota_A	c__Clostridia	o__Lachnospirales	f__Lachnospiraceae	g__Coprococcus	s__Coprococcus sp900066115
PD	PD36	bin40	MAG3008	d__Bacteria	p__Bacillota_A	c__Clostridia	o__Lachnospirales	f__Lachnospiraceae	g__Coprococcus	s__Coprococcus sp900066115
PD	PD78	bin34	MAG3327	d__Bacteria	p__Bacillota_A	c__Clostridia	o__Lachnospirales	f__Lachnospiraceae	g__Hominisplanchenecus	s__Hominisplanchenecus faecis
PD	PD66	bin15	MAG474	d__Bacteria	p__Bacillota_A	c__Clostridia	o__Lachnospirales	f__Lachnospiraceae	g__Hominisplanchenecus	s__Hominisplanchenecus faecis
PD	PD62	bin8	MAG2061	d__Bacteria	p__Bacillota_A	c__Clostridia	o__Lachnospirales	f__Lachnospiraceae	g__Hominisplanchenecus	s__Hominisplanchenecus faecis
PD	PD12	bin45	MAG1253	d__Bacteria	p__Bacillota_A	c__Clostridia	o__Lachnospirales	f__Lachnospiraceae	g__Hominisplanchenecus	s__Hominisplanchenecus faecis
PD	PD57	bin56	MAG3446	d__Bacteria	p__Bacillota_A	c__Clostridia	o__Lachnospirales	f__Lachnospiraceae	g__Hominisplanchenecus	s__Hominisplanchenecus faecis
PD	PD3	bin34	MAG3073	d__Bacteria	p__Bacillota_A	c__Clostridia	o__Lachnospirales	f__Lachnospiraceae	g__Hominisplanchenecus	s__Hominisplanchenecus faecis
PD	PD92	bin2	MAG185	d__Bacteria	p__Bacillota_A	c__Clostridia	o__Lachnospirales	f__Lachnospiraceae	g__Hominisplanchenecus	s__Hominisplanchenecus faecis
PD	PD26	bin5	MAG1048	d__Bacteria	p__Bacillota_A	c__Clostridia	o__Lachnospirales	f__Lachnospiraceae	g__Butyribacter	s__Butyribacter hominis
PD	PD28	bin7	MAG719	d__Bacteria	p__Bacillota_A	c__Clostridia	o__Lachnospirales	f__Lachnospiraceae	g__Butyribacter	s__Butyribacter hominis
PD	PD85	bin16	MAG489	d__Bacteria	p__Bacillota_A	c__Clostridia	o__Lachnospirales	f__Lachnospiraceae	g__Blautia_A	s__Blautia_A wexlerae
PD	PD19	bin8	MAG480	d__Bacteria	p__Bacillota_A	c__Clostridia	o__Lachnospirales	f__Lachnospiraceae	g__Blautia_A	s__Blautia_A wexlerae
PD	PD29	bin77	MAG1121	d__Bacteria	p__Bacillota_A	c__Clostridia	o__Lachnospirales	f__Lachnospiraceae	g__Blautia_A	s__Blautia_A wexlerae
PD	PD13	bin27	MAG219	d__Bacteria	p__Bacillota_A	c__Clostridia	o__Lachnospirales	f__Lachnospiraceae	g__Blautia_A	s__Blautia_A wexlerae
PD	PD75	bin50	MAG2228	d__Bacteria	p__Bacillota_A	c__Clostridia	o__Lachnospirales	f__Lachnospiraceae	g__Blautia_A	s__Blautia_A wexlerae
PD	PD21	bin4	MAG2853	d__Bacteria	p__Bacillota_A	c__Clostridia	o__Lachnospirales	f__Lachnospiraceae	g__Blautia_A	s__Blautia_A wexlerae
PD	PD36	bin44	MAG3319	d__Bacteria	p__Bacillota_A	c__Clostridia	o__Lachnospirales	f__Lachnospiraceae	g__Blautia_A	s__Blautia_A wexlerae
PD	PD72	bin7	MAG1324	d__Bacteria	p__Bacillota_A	c__Clostridia	o__Lachnospirales	f__Lachnospiraceae	g__Blautia_A	s__Blautia_A wexlerae
PD	PD58	bin78	MAG1162	d__Bacteria	p__Bacillota_A	c__Clostridia	o__Lachnospirales	f__Lachnospiraceae	g__Blautia_A	s__Blautia_A wexlerae
PD	PD39	bin11	MAG301	d__Bacteria	p__Bacillota_A	c__Clostridia	o__Lachnospirales	f__Lachnospiraceae	g__Blautia_A	s__Blautia_A wexlerae
PD	PD46	bin20	MAG726	d__Bacteria	p__Bacillota_A	c__Clostridia	o__Lachnospirales	f__Lachnospiraceae	g__Blautia_A	s__Blautia_A wexlerae
PD	PD8	bin3	MAG970	d__Bacteria	p__Bacillota_A	c__Clostridia	o__Lachnospirales	f__Lachnospiraceae	g__Blautia_A	s__Blautia_A wexlerae

PD	PD51	bin24	MAG3097	d__Bacteria	p__Bacillota_A	c__Clostridia	o__Lachnospirales	f__Lachnospiraceae	g__Blautia_A	s__Blautia_A wexlerae
PD	PD66	bin5	MAG862	d__Bacteria	p__Bacillota_A	c__Clostridia	o__Lachnospirales	f__Lachnospiraceae	g__Blautia_A	s__Blautia_A wexlerae
PD	PD87	bin10	MAG1675	d__Bacteria	p__Bacillota_A	c__Clostridia	o__Lachnospirales	f__Lachnospiraceae	g__Blautia_A	s__Blautia_A wexlerae
PD	PD42	bin9	MAG695	d__Bacteria	p__Bacillota_A	c__Clostridia	o__Lachnospirales	f__Lachnospiraceae	g__Blautia_A	s__Blautia_A wexlerae
PD	PD48	bin12	MAG3445	d__Bacteria	p__Bacillota_A	c__Clostridia	o__Lachnospirales	f__Lachnospiraceae	g__Blautia_A	s__Blautia_A wexlerae
PD	PD14	bin43	MAG2459	d__Bacteria	p__Bacillota_A	c__Clostridia	o__Lachnospirales	f__Lachnospiraceae	g__Blautia_A	s__Blautia_A wexlerae
PD	PD81	bin37	MAG1378	d__Bacteria	p__Bacillota_A	c__Clostridia	o__Lachnospirales	f__Lachnospiraceae	g__Blautia_A	s__Blautia_A wexlerae
PD	PD97	bin21	MAG2207	d__Bacteria	p__Bacillota_A	c__Clostridia	o__Lachnospirales	f__Lachnospiraceae	g__Massilistercora	s__Massilistercora sp902406105
PD	PD23	bin24	MAG1371	d__Bacteria	p__Bacillota_A	c__Clostridia	o__Lachnospirales	f__Lachnospiraceae	g__Clostridium_AP	s__Clostridium_AP sp000509125
PD	PD37	bin24	MAG642	d__Bacteria	p__Bacillota_A	c__Clostridia	o__Lachnospirales	f__Lachnospiraceae	g__Clostridium_AP	s__Clostridium_AP sp000509125
PD	PD81	bin27	MAG1828	d__Bacteria	p__Bacillota_A	c__Clostridia	o__Lachnospirales	f__Lachnospiraceae	g__Clostridium_AP	s__Clostridium_AP sp000509125
PD	PD9	bin61	MAG581	d__Bacteria	p__Bacillota_A	c__Clostridia	o__Lachnospirales	f__Lachnospiraceae	g__Agathobacter	s__Agathobacter faecis
PD	PD78	bin32	MAG1921	d__Bacteria	p__Bacillota_A	c__Clostridia	o__Lachnospirales	f__Lachnospiraceae	g__Agathobacter	s__Agathobacter faecis
PD	PD81	bin30	MAG374	d__Bacteria	p__Bacillota_A	c__Clostridia	o__Lachnospirales	f__Lachnospiraceae	g__Agathobacter	s__Agathobacter faecis
PD	PD45	bin35	MAG3187	d__Bacteria	p__Bacillota_A	c__Clostridia	o__Lachnospirales	f__Lachnospiraceae	g__Agathobacter	s__Agathobacter faecis
PD	PD23	bin17	MAG1088	d__Bacteria	p__Bacillota_A	c__Clostridia	o__Lachnospirales	f__Lachnospiraceae	g__Agathobacter	s__Agathobacter faecis
PD	PD69	bin38	MAG1885	d__Bacteria	p__Bacillota_A	c__Clostridia	o__Lachnospirales	f__Lachnospiraceae	g__Agathobacter	s__Agathobacter faecis
PD	PD91	bin7	MAG3261	d__Bacteria	p__Bacillota_A	c__Clostridia	o__Lachnospirales	f__Lachnospiraceae	g__Agathobacter	s__Agathobacter faecis
PD	PD35	bin10	MAG775	d__Bacteria	p__Bacillota_A	c__Clostridia	o__Lachnospirales	f__Lachnospiraceae	g__Agathobacter	s__Agathobacter faecis
PD	PD68	bin32	MAG1600	d__Bacteria	p__Bacillota_A	c__Clostridia	o__Lachnospirales	f__Lachnospiraceae	g__Agathobacter	s__Agathobacter faecis
PD	PD36	bin5	MAG2646	d__Bacteria	p__Bacillota_A	c__Clostridia	o__Lachnospirales	f__Lachnospiraceae	g__Agathobacter	s__Agathobacter faecis
PD	PD28	bin23	MAG1455	d__Bacteria	p__Bacillota_A	c__Clostridia	o__Lachnospirales	f__Lachnospiraceae	g__Agathobacter	s__Agathobacter faecis
PD	PD62	bin11	MAG3120	d__Bacteria	p__Bacillota_A	c__Clostridia	o__Lachnospirales	f__Lachnospiraceae	g__Agathobacter	s__Agathobacter faecis
PD	PD87	bin23	MAG1169	d__Bacteria	p__Bacillota_A	c__Clostridia	o__Lachnospirales	f__Lachnospiraceae	g__Agathobacter	s__Agathobacter faecis
PD	PD40	bin55	MAG1320	d__Bacteria	p__Bacillota_A	c__Clostridia	o__Lachnospirales	f__Lachnospiraceae	g__Agathobacter	s__Agathobacter faecis
PD	PD56	bin23	MAG3484	d__Bacteria	p__Bacillota_A	c__Clostridia	o__Lachnospirales	f__Lachnospiraceae	g__Agathobacter	s__Agathobacter faecis
PD	PD17	bin28	MAG2009	d__Bacteria	p__Bacillota_A	c__Clostridia	o__Lachnospirales	f__Lachnospiraceae	g__Agathobacter	s__Agathobacter faecis
PD	PD31	bin46	MAG1352	d__Bacteria	p__Bacillota_A	c__Clostridia	o__Lachnospirales	f__Lachnospiraceae	g__Agathobacter	s__Agathobacter faecis
PD	PD12	bin12	MAG2784	d__Bacteria	p__Bacillota_A	c__Clostridia	o__Lachnospirales	f__Lachnospiraceae	g__Blautia_A	s__Blautia_A obeum
PD	PD98	bin11	MAG2501	d__Bacteria	p__Bacillota_A	c__Clostridia	o__Lachnospirales	f__Lachnospiraceae	g__Blautia_A	s__Blautia_A obeum
PD	PD97	bin64	MAG3143	d__Bacteria	p__Bacillota_A	c__Clostridia	o__Lachnospirales	f__Lachnospiraceae	g__Blautia_A	s__Blautia_A obeum
PD	PD2	bin52	MAG2097	d__Bacteria	p__Bacillota_A	c__Clostridia	o__Lachnospirales	f__Lachnospiraceae	g__Blautia_A	s__Blautia_A obeum
PD	PD64	bin25	MAG1260	d__Bacteria	p__Bacillota_A	c__Clostridia	o__Lachnospirales	f__Lachnospiraceae	g__Lachnospira	s__Lachnospira sp003451515
PD	PD92	bin61	MAG3408	d__Bacteria	p__Bacillota_A	c__Clostridia	o__Lachnospirales	f__Lachnospiraceae	g__Merdimonas	s__Merdimonas faecis
PD	PD76	bin32	MAG1818	d__Bacteria	p__Bacillota_A	c__Clostridia	o__Lachnospirales	f__Lachnospiraceae	g__Blautia	s__Blautia stercoris
PD	PD2	bin55	MAG2319	d__Bacteria	p__Bacillota_A	c__Clostridia	o__Lachnospirales	f__Lachnospiraceae	g__Blautia	s__Blautia stercoris
PD	PD32	bin82	MAG1532	d__Bacteria	p__Bacillota_A	c__Clostridia	o__Lachnospirales	f__Lachnospiraceae	g__RUG115	s__RUG115 sp900066395
PD	PD19	bin30	MAG3253	d__Bacteria	p__Bacillota_A	c__Clostridia	o__Lachnospirales	f__Lachnospiraceae	g__UBA4285	s__Unclassified
PD	PD71	bin21	MAG1745	d__Bacteria	p__Bacillota_A	c__Clostridia	o__Lachnospirales	f__Lachnospiraceae	g__Ruminococcus_B	s__Ruminococcus_B gnavus
PD	PD21	bin10	MAG2269	d__Bacteria	p__Bacillota_A	c__Clostridia	o__Lachnospirales	f__Lachnospiraceae	g__Ruminococcus_B	s__Ruminococcus_B gnavus
PD	PD94	bin12	MAG556	d__Bacteria	p__Bacillota_A	c__Clostridia	o__Lachnospirales	f__Lachnospiraceae	g__Massilistercora	s__Massilistercora timonensis
PD	PD3	bin1	MAG2385	d__Bacteria	p__Bacillota_A	c__Clostridia	o__Lachnospirales	f__Lachnospiraceae	g__Lachnospira	s__Lachnospira sp000437735
PD	PD18	bin8	MAG340	d__Bacteria	p__Bacillota_A	c__Clostridia	o__Lachnospirales	f__Lachnospiraceae	g__Fusicatenibacter	s__Fusicatenibacter saccharivorans
PD	PD42	bin12	MAG670	d__Bacteria	p__Bacillota_A	c__Clostridia	o__Lachnospirales	f__Lachnospiraceae	g__Fusicatenibacter	s__Fusicatenibacter saccharivorans
PD	PD83	bin26	MAG2461	d__Bacteria	p__Bacillota_A	c__Clostridia	o__Lachnospirales	f__Lachnospiraceae	g__Fusicatenibacter	s__Fusicatenibacter saccharivorans
PD	PD75	bin9	MAG179	d__Bacteria	p__Bacillota_A	c__Clostridia	o__Lachnospirales	f__Lachnospiraceae	g__Fusicatenibacter	s__Fusicatenibacter saccharivorans
PD	PD81	bin40	MAG3394	d__Bacteria	p__Bacillota_A	c__Clostridia	o__Lachnospirales	f__Lachnospiraceae	g__Fusicatenibacter	s__Fusicatenibacter saccharivorans
PD	PD37	bin14	MAG1852	d__Bacteria	p__Bacillota_A	c__Clostridia	o__Lachnospirales	f__Lachnospiraceae	g__Fusicatenibacter	s__Fusicatenibacter saccharivorans
PD	PD1	bin30	MAG1812	d__Bacteria	p__Bacillota_A	c__Clostridia	o__Lachnospirales	f__Lachnospiraceae	g__Fusicatenibacter	s__Fusicatenibacter saccharivorans
PD	PD31	bin15	MAG2818	d__Bacteria	p__Bacillota_A	c__Clostridia	o__Lachnospirales	f__Lachnospiraceae	g__Fusicatenibacter	s__Fusicatenibacter saccharivorans
PD	PD78	bin33	MAG50	d__Bacteria	p__Bacillota_A	c__Clostridia	o__Lachnospirales	f__Lachnospiraceae	g__Fusicatenibacter	s__Fusicatenibacter saccharivorans

PD	PD71	bin12	MAG331	d__Bacteria	p__Bacillota_A	c__Clostridia	o__Lachnospirales	f__Lachnospiraceae	g__Fusicatenibacter	s__Fusicatenibacter saccharivorans
PD	PD57	bin87	MAG2849	d__Bacteria	p__Bacillota_A	c__Clostridia	o__Lachnospirales	f__Lachnospiraceae	g__Fusicatenibacter	s__Fusicatenibacter saccharivorans
PD	PD45	bin29	MAG2555	d__Bacteria	p__Bacillota_A	c__Clostridia	o__Lachnospirales	f__Lachnospiraceae	g__Fusicatenibacter	s__Fusicatenibacter saccharivorans
PD	PD62	bin56	MAG3078	d__Bacteria	p__Bacillota_A	c__Clostridia	o__Lachnospirales	f__Lachnospiraceae	g__Fusicatenibacter	s__Fusicatenibacter saccharivorans
PD	PD29	bin48	MAG3363	d__Bacteria	p__Bacillota_A	c__Clostridia	o__Lachnospirales	f__Lachnospiraceae	g__Fusicatenibacter	s__Fusicatenibacter saccharivorans
PD	PD58	bin63	MAG1291	d__Bacteria	p__Bacillota_A	c__Clostridia	o__Lachnospirales	f__Lachnospiraceae	g__Fusicatenibacter	s__Fusicatenibacter saccharivorans
PD	PD82	bin43	MAG3224	d__Bacteria	p__Bacillota_A	c__Clostridia	o__Lachnospirales	f__Lachnospiraceae	g__Fusicatenibacter	s__Fusicatenibacter saccharivorans
PD	PD77	bin28	MAG453	d__Bacteria	p__Bacillota_A	c__Clostridia	o__Lachnospirales	f__Lachnospiraceae	g__Fusicatenibacter	s__Fusicatenibacter saccharivorans
PD	PD9	bin54	MAG2219	d__Bacteria	p__Bacillota_A	c__Clostridia	o__Lachnospirales	f__Lachnospiraceae	g__Fusicatenibacter	s__Fusicatenibacter saccharivorans
PD	PD26	bin26	MAG2201	d__Bacteria	p__Bacillota_A	c__Clostridia	o__Lachnospirales	f__Lachnospiraceae	g__Fusicatenibacter	s__Fusicatenibacter saccharivorans
PD	PD66	bin10	MAG2486	d__Bacteria	p__Bacillota_A	c__Clostridia	o__Lachnospirales	f__Lachnospiraceae	g__Fusicatenibacter	s__Fusicatenibacter saccharivorans
PD	PD76	bin60	MAG867	d__Bacteria	p__Bacillota_A	c__Clostridia	o__Lachnospirales	f__Lachnospiraceae	g__Fusicatenibacter	s__Fusicatenibacter saccharivorans
PD	PD2	bin60	MAG187	d__Bacteria	p__Bacillota_A	c__Clostridia	o__Lachnospirales	f__Lachnospiraceae	g__Fusicatenibacter	s__Fusicatenibacter saccharivorans
PD	PD44	bin4	MAG1485	d__Bacteria	p__Bacillota_A	c__Clostridia	o__Lachnospirales	f__Lachnospiraceae	g__Fusicatenibacter	s__Fusicatenibacter saccharivorans
PD	PD69	bin37	MAG124	d__Bacteria	p__Bacillota_A	c__Clostridia	o__Lachnospirales	f__Lachnospiraceae	g__Fusicatenibacter	s__Fusicatenibacter saccharivorans
PD	PD40	bin32	MAG2145	d__Bacteria	p__Bacillota_A	c__Clostridia	o__Lachnospirales	f__Lachnospiraceae	g__Fusicatenibacter	s__Fusicatenibacter saccharivorans
PD	PD12	bin43	MAG139	d__Bacteria	p__Bacillota_A	c__Clostridia	o__Lachnospirales	f__Lachnospiraceae	g__Fusicatenibacter	s__Fusicatenibacter saccharivorans
PD	PD32	bin47	MAG2816	d__Bacteria	p__Bacillota_A	c__Clostridia	o__Lachnospirales	f__Lachnospiraceae	g__Fusicatenibacter	s__Fusicatenibacter saccharivorans
PD	PD35	bin40	MAG2120	d__Bacteria	p__Bacillota_A	c__Clostridia	o__Lachnospirales	f__Lachnospiraceae	g__Fusicatenibacter	s__Fusicatenibacter saccharivorans
PD	PD47	bin16	MAG504	d__Bacteria	p__Bacillota_A	c__Clostridia	o__Lachnospirales	f__Lachnospiraceae	g__Fusicatenibacter	s__Fusicatenibacter saccharivorans
PD	PD64	bin1	MAG1792	d__Bacteria	p__Bacillota_A	c__Clostridia	o__Lachnospirales	f__Lachnospiraceae	g__Fusicatenibacter	s__Fusicatenibacter saccharivorans
PD	PD44	bin43	MAG760	d__Bacteria	p__Bacillota_A	c__Clostridia	o__Lachnospirales	f__Lachnospiraceae	g__Schaedlerella	s__Schaedlerella glycyrrhizinilytica_A
PD	PD4	bin12	MAG1372	d__Bacteria	p__Bacillota_A	c__Clostridia	o__Lachnospirales	f__Lachnospiraceae	g__Schaedlerella	s__Schaedlerella glycyrrhizinilytica_A
PD	PD83	bin22	MAG1627	d__Bacteria	p__Bacillota_A	c__Clostridia	o__Lachnospirales	f__Lachnospiraceae	g__Schaedlerella	s__Schaedlerella glycyrrhizinilytica_A
PD	PD86	bin27	MAG1919	d__Bacteria	p__Bacillota_A	c__Clostridia	o__Lachnospirales	f__Lachnospiraceae	g__Schaedlerella	s__Schaedlerella glycyrrhizinilytica_A
PD	PD99	bin28	MAG668	d__Bacteria	p__Bacillota_A	c__Clostridia	o__Lachnospirales	f__Lachnospiraceae	g__Schaedlerella	s__Schaedlerella glycyrrhizinilytica_A
PD	PD91	bin16	MAG2492	d__Bacteria	p__Bacillota_A	c__Clostridia	o__Lachnospirales	f__CAG-274	g__CAG-274	s__CAG-274 sp900545305
PD	PD77	bin9	MAG1916	d__Bacteria	p__Bacillota_A	c__Clostridia	o__Lachnospirales	f__CAG-274	g__CAG-274	s__CAG-274 sp900545305
PD	PD95	bin28	MAG2138	d__Bacteria	p__Bacillota_A	c__Clostridia	o__Lachnospirales	f__Lachnospiraceae	g__Blautia	s__Blautia hansenii
PD	PD81	bin25	MAG2338	d__Bacteria	p__Bacillota_A	c__Clostridia	o__Lachnospirales	f__Lachnospiraceae	g__Blautia	s__Blautia hansenii
PD	PD10	bin48	MAG1949	d__Bacteria	p__Bacillota_A	c__Clostridia	o__Lachnospirales	f__Lachnospiraceae	g__Blautia_A	s__Blautia_A sp900120195
PD	PD66	bin14	MAG1707	d__Bacteria	p__Bacillota_A	c__Clostridia	o__Lachnospirales	f__Lachnospiraceae	g__Blautia_A	s__Blautia_A sp900120195
PD	PD21	bin32	MAG1028	d__Bacteria	p__Bacillota_A	c__Clostridia	o__Lachnospirales	f__Lachnospiraceae	g__Bariatricus	s__Bariatricus comes
PD	PD39	bin19	MAG80	d__Bacteria	p__Bacillota_A	c__Clostridia	o__Lachnospirales	f__Lachnospiraceae	g__Agathobacter	s__Agathobacter sp900543445
PD	PD68	bin4	MAG3457	d__Bacteria	p__Bacillota_A	c__Clostridia	o__Lachnospirales	f__Lachnospiraceae	g__Agathobacter	s__Agathobacter sp900543445
PD	PD3	bin45	MAG773	d__Bacteria	p__Bacillota_A	c__Clostridia	o__Lachnospirales	f__Lachnospiraceae	g__Agathobacter	s__Agathobacter sp900543445
PD	PD57	bin13	MAG2633	d__Bacteria	p__Bacillota_A	c__Clostridia	o__Lachnospirales	f__Lachnospiraceae	g__Agathobacter	s__Agathobacter sp900543445
PD	PD19	bin9	MAG1492	d__Bacteria	p__Bacillota_A	c__Clostridia	o__Lachnospirales	f__Lachnospiraceae	g__CAG-56	s__CAG-56 sp905200565
PD	PD24	bin21	MAG1710	d__Bacteria	p__Bacillota_A	c__Clostridia	o__Lachnospirales	f__Lachnospiraceae	g__Wujia	s__Wujia chipingensis
PD	PD29	bin63	MAG2580	d__Bacteria	p__Bacillota_A	c__Clostridia	o__Lachnospirales	f__Lachnospiraceae	g__Wujia	s__Wujia chipingensis
PD	PD32	bin81	MAG755	d__Bacteria	p__Bacillota_A	c__Clostridia	o__Lachnospirales	f__Lachnospiraceae	g__Wujia	s__Wujia chipingensis
PD	PD57	bin53	MAG1219	d__Bacteria	p__Bacillota_A	c__Clostridia	o__Lachnospirales	f__Lachnospiraceae	g__Wujia	s__Wujia chipingensis
PD	PD64	bin8	MAG3357	d__Bacteria	p__Bacillota_A	c__Clostridia	o__Lachnospirales	f__Lachnospiraceae	g__Wujia	s__Wujia chipingensis
PD	PD3	bin27	MAG1289	d__Bacteria	p__Bacillota_A	c__Clostridia	o__Lachnospirales	f__Lachnospiraceae	g__Wujia	s__Wujia chipingensis
PD	PD94	bin39	MAG3056	d__Bacteria	p__Bacillota_A	c__Clostridia	o__Lachnospirales	f__Lachnospiraceae	g__Wujia	s__Wujia chipingensis
PD	PD87	bin6	MAG1137	d__Bacteria	p__Bacillota_A	c__Clostridia	o__Lachnospirales	f__Lachnospiraceae	g__Wujia	s__Wujia chipingensis
PD	PD34	bin38	MAG974	d__Bacteria	p__Bacillota_A	c__Clostridia	o__Lachnospirales	f__Lachnospiraceae	g__Wujia	s__Wujia chipingensis
PD	PD28	bin2	MAG877	d__Bacteria	p__Bacillota_A	c__Clostridia	o__Lachnospirales	f__Lachnospiraceae	g__Wujia	s__Wujia chipingensis
PD	PD61	bin6	MAG558	d__Bacteria	p__Bacillota_A	c__Clostridia	o__Lachnospirales	f__Lachnospiraceae	g__Wujia	s__Wujia chipingensis
PD	PD26	bin18	MAG1004	d__Bacteria	p__Bacillota_A	c__Clostridia	o__Lachnospirales	f__Lachnospiraceae	g__Lachnospira	s__Lachnospira sp000436475
PD	PD3	bin31	MAG1850	d__Bacteria	p__Bacillota_A	c__Clostridia	o__Lachnospirales	f__Lachnospiraceae	g__Lachnospira	s__Lachnospira sp000436475

PD	PD62	bin22	MAG3309	d__Bacteria	p__Bacillota_A	c__Clostridia	o__Lachnospirales	f__Lachnospiraceae	g__Lachnospira	s__Lachnospira sp000436475
PD	PD9	bin53	MAG182	d__Bacteria	p__Bacillota_A	c__Clostridia	o__Lachnospirales	f__Lachnospiraceae	g__Lachnospira	s__Lachnospira sp000436475
PD	PD32	bin76	MAG379	d__Bacteria	p__Bacillota_A	c__Clostridia	o__Lachnospirales	f__Lachnospiraceae	g__Lachnospira	s__Lachnospira sp000436475
PD	PD25	bin9	MAG2211	d__Bacteria	p__Bacillota_A	c__Clostridia	o__Lachnospirales	f__Lachnospiraceae	g__Lachnospira	s__Lachnospira sp000436475
PD	PD83	bin3	MAG1991	d__Bacteria	p__Bacillota_A	c__Clostridia	o__Lachnospirales	f__Lachnospiraceae	g__Lachnospira	s__Lachnospira sp000436475
PD	PD47	bin25	MAG3150	d__Bacteria	p__Bacillota_A	c__Clostridia	o__Lachnospirales	f__Lachnospiraceae	g__Lachnospira	s__Lachnospira sp000436475
PD	PD44	bin36	MAG923	d__Bacteria	p__Bacillota_A	c__Clostridia	o__Lachnospirales	f__Lachnospiraceae	g__Blautia_A	s__Blautia_A sp003471165
PD	PD1	bin33	MAG1096	d__Bacteria	p__Bacillota_A	c__Clostridia	o__Lachnospirales	f__Lachnospiraceae	g__Blautia_A	s__Blautia_A sp003471165
PD	PD37	bin42	MAG345	d__Bacteria	p__Bacillota_A	c__Clostridia	o__Lachnospirales	f__Lachnospiraceae	g__Blautia_A	s__Blautia_A sp003471165
PD	PD95	bin20	MAG2429	d__Bacteria	p__Bacillota_A	c__Clostridia	o__Lachnospirales	f__Lachnospiraceae	g__Blautia_A	s__Blautia_A sp003471165
PD	PD42	bin19	MAG2149	d__Bacteria	p__Bacillota_A	c__Clostridia	o__Lachnospirales	f__Lachnospiraceae	g__Blautia_A	s__Blautia_A sp003471165
PD	PD6	bin21	MAG586	d__Bacteria	p__Bacillota_A	c__Clostridia	o__Lachnospirales	f__Lachnospiraceae	g__Enterocloster	s__Enterocloster bolteae
PD	PD21	bin13	MAG833	d__Bacteria	p__Bacillota_A	c__Clostridia	o__Lachnospirales	f__Lachnospiraceae	g__Enterocloster	s__Enterocloster bolteae
PD	PD11	bin21	MAG1634	d__Bacteria	p__Bacillota_A	c__Clostridia	o__Lachnospirales	f__Lachnospiraceae	g__Enterocloster	s__Enterocloster bolteae
PD	PD1	bin22	MAG2933	d__Bacteria	p__Bacillota_A	c__Clostridia	o__Lachnospirales	f__Lachnospiraceae	g__Enterocloster	s__Enterocloster bolteae
PD	PD45	bin49	MAG1384	d__Bacteria	p__Bacillota_A	c__Clostridia	o__Lachnospirales	f__Lachnospiraceae	g__Eisenbergiella	s__Eisenbergiella sp900066775
PD	PD76	bin43	MAG2617	d__Bacteria	p__Bacillota_A	c__Clostridia	o__Lachnospirales	f__Lachnospiraceae	g__Eisenbergiella	s__Eisenbergiella sp900066775
PD	PD75	bin5	MAG2491	d__Bacteria	p__Bacillota_A	c__Clostridia	o__Lachnospirales	f__Lachnospiraceae	g__Eisenbergiella	s__Eisenbergiella sp900066775
PD	PD28	bin4	MAG1113	d__Bacteria	p__Bacillota_A	c__Clostridia	o__Lachnospirales	f__Lachnospiraceae	g__Eisenbergiella	s__Eisenbergiella sp900066775
PD	PD60	bin21	MAG2440	d__Bacteria	p__Bacillota_A	c__Clostridia	o__Lachnospirales	f__Lachnospiraceae	g__Eisenbergiella	s__Eisenbergiella sp900066775
PD	PD71	bin34	MAG1016	d__Bacteria	p__Bacillota_A	c__Clostridia	o__Lachnospirales	f__Lachnospiraceae	g__Eisenbergiella	s__Eisenbergiella sp900066775
PD	PD44	bin11	MAG146	d__Bacteria	p__Bacillota_A	c__Clostridia	o__Lachnospirales	f__Lachnospiraceae	g__Eisenbergiella	s__Eisenbergiella sp900066775
PD	PD45	bin30	MAG658	d__Bacteria	p__Bacillota_A	c__Clostridia	o__Lachnospirales	f__Lachnospiraceae	g__CAG-882	s__CAG-882 sp003486385
PD	PD62	bin7	MAG478	d__Bacteria	p__Bacillota_A	c__Clostridia	o__Lachnospirales	f__Lachnospiraceae	g__CAG-882	s__CAG-882 sp003486385
PD	PD32	bin31	MAG1781	d__Bacteria	p__Bacillota_A	c__Clostridia	o__Lachnospirales	f__Lachnospiraceae	g__CAG-882	s__CAG-882 sp003486385
PD	PD28	bin52	MAG2699	d__Bacteria	p__Bacillota_A	c__Clostridia	o__Lachnospirales	f__Lachnospiraceae	g__CAG-882	s__CAG-882 sp003486385
PD	PD61	bin1	MAG1346	d__Bacteria	p__Bacillota_A	c__Clostridia	o__Lachnospirales	f__Lachnospiraceae	g__Mediterraneibacter	s__Mediterraneibacter faecis
PD	PD68	bin15	MAG3268	d__Bacteria	p__Bacillota_A	c__Clostridia	o__Lachnospirales	f__Lachnospiraceae	g__Mediterraneibacter	s__Mediterraneibacter faecis
PD	PD99	bin39	MAG567	d__Bacteria	p__Bacillota_A	c__Clostridia	o__Lachnospirales	f__Lachnospiraceae	g__Mediterraneibacter	s__Mediterraneibacter faecis
PD	PD44	bin19	MAG1664	d__Bacteria	p__Bacillota_A	c__Clostridia	o__Lachnospirales	f__Lachnospiraceae	g__Mediterraneibacter	s__Mediterraneibacter faecis
PD	PD41	bin51	MAG2719	d__Bacteria	p__Bacillota_A	c__Clostridia	o__Lachnospirales	f__Lachnospiraceae	g__Mediterraneibacter	s__Mediterraneibacter faecis
PD	PD6	bin3	MAG2224	d__Bacteria	p__Bacillota_A	c__Clostridia	o__Lachnospirales	f__Lachnospiraceae	g__Mediterraneibacter	s__Mediterraneibacter faecis
PD	PD18	bin5	MAG2841	d__Bacteria	p__Bacillota_A	c__Clostridia	o__Lachnospirales	f__Lachnospiraceae	g__CAG-317	s__CAG-317 sp000433535
PD	PD18	bin55	MAG3510	d__Bacteria	p__Bacillota_A	c__Clostridia	o__Lachnospirales	f__Lachnospiraceae	g__Ventrisoma	s__Ventrisoma faecale
PD	PD71	bin39	MAG1109	d__Bacteria	p__Bacillota_A	c__Clostridia	o__Lachnospirales	f__Lachnospiraceae	g__Lachnospira	s__Lachnospira eligens_A
PD	PD1	bin7	MAG1584	d__Bacteria	p__Bacillota_A	c__Clostridia	o__Lachnospirales	f__Lachnospiraceae	g__Lachnospira	s__Lachnospira eligens_A
PD	PD18	bin46	MAG681	d__Bacteria	p__Bacillota_A	c__Clostridia	o__Lachnospirales	f__Lachnospiraceae	g__Lachnospira	s__Lachnospira eligens_A
PD	PD72	bin22	MAG2939	d__Bacteria	p__Bacillota_A	c__Clostridia	o__Lachnospirales	f__Lachnospiraceae	g__Lachnospira	s__Lachnospira eligens_A
PD	PD46	bin52	MAG628	d__Bacteria	p__Bacillota_A	c__Clostridia	o__Lachnospirales	f__Lachnospiraceae	g__Lachnospira	s__Lachnospira eligens_A
PD	PD3	bin8	MAG1638	d__Bacteria	p__Bacillota_A	c__Clostridia	o__Lachnospirales	f__Lachnospiraceae	g__Lachnospira	s__Lachnospira eligens_A
PD	PD11	bin20	MAG388	d__Bacteria	p__Bacillota_A	c__Clostridia	o__Lachnospirales	f__Lachnospiraceae	g__Lachnospira	s__Lachnospira eligens_A
PD	PD29	bin8	MAG512	d__Bacteria	p__Bacillota_A	c__Clostridia	o__Lachnospirales	f__Lachnospiraceae	g__Lachnospira	s__Lachnospira eligens_A
PD	PD41	bin26	MAG87	d__Bacteria	p__Bacillota_A	c__Clostridia	o__Lachnospirales	f__Lachnospiraceae	g__Lachnospira	s__Lachnospira eligens_A
PD	PD25	bin62	MAG2479	d__Bacteria	p__Bacillota_A	c__Clostridia	o__Lachnospirales	f__Lachnospiraceae	g__Lachnospira	s__Lachnospira eligens_A
PD	PD24	bin57	MAG1164	d__Bacteria	p__Bacillota_A	c__Clostridia	o__Lachnospirales	f__Lachnospiraceae	g__Lachnospira	s__Lachnospira eligens_A
PD	PD39	bin28	MAG2589	d__Bacteria	p__Bacillota_A	c__Clostridia	o__Lachnospirales	f__Lachnospiraceae	g__Lachnospira	s__Lachnospira eligens_A
PD	PD100	bin34	MAG577	d__Bacteria	p__Bacillota_A	c__Clostridia	o__Lachnospirales	f__Lachnospiraceae	g__Lachnospira	s__Lachnospira eligens_A
PD	PD87	bin28	MAG1640	d__Bacteria	p__Bacillota_A	c__Clostridia	o__Lachnospirales	f__Lachnospiraceae	g__Butyribacter	s__Butyribacter sp003529475
PD	PD9	bin23	MAG2575	d__Bacteria	p__Bacillota_A	c__Clostridia	o__Lachnospirales	f__Lachnospiraceae	g__Butyribacter	s__Butyribacter sp003529475
PD	PD82	bin60	MAG1918	d__Bacteria	p__Bacillota_A	c__Clostridia	o__Lachnospirales	f__Lachnospiraceae	g__Butyribacter	s__Butyribacter sp003529475
PD	PD13	bin4	MAG1049	d__Bacteria	p__Bacillota_A	c__Clostridia	o__Lachnospirales	f__Lachnospiraceae	g__Butyribacter	s__Butyribacter sp003529475

PD	PD70	bin18	MAG847	d__Bacteria	p__Bacillota_A	c__Clostridia	o__Lachnospirales	f__Lachnospiraceae	g__Butyribacter	s__Butyribacter sp003529475
PD	PD48	bin20	MAG853	d__Bacteria	p__Bacillota_A	c__Clostridia	o__Lachnospirales	f__Lachnospiraceae	g__Butyribacter	s__Butyribacter sp003529475
PD	PD28	bin31	MAG1655	d__Bacteria	p__Bacillota_A	c__Clostridia	o__Lachnospirales	f__Lachnospiraceae	g__Butyribacter	s__Butyribacter sp003529475
PD	PD40	bin29	MAG1249	d__Bacteria	p__Bacillota_A	c__Clostridia	o__Lachnospirales	f__Lachnospiraceae	g__Clostridium_Q	s__Clostridium_Q fessum
PD	PD78	bin4	MAG2481	d__Bacteria	p__Bacillota_A	c__Clostridia	o__Lachnospirales	f__Lachnospiraceae	g__Clostridium_Q	s__Clostridium_Q fessum
PD	PD91	bin49	MAG3209	d__Bacteria	p__Bacillota_A	c__Clostridia	o__Lachnospirales	f__Lachnospiraceae	g__Clostridium_Q	s__Clostridium_Q fessum
PD	PD18	bin22	MAG2179	d__Bacteria	p__Bacillota_A	c__Clostridia	o__Lachnospirales	f__Lachnospiraceae	g__Oliverpabstia	s__Oliverpabstia sp002361775
PD	PD67	bin40	MAG308	d__Bacteria	p__Bacillota_A	c__Clostridia	o__Lachnospirales	f__Lachnospiraceae	g__UBA11774	s__UBA11774 sp003507655
PD	PD34	bin17	MAG2360	d__Bacteria	p__Bacillota_A	c__Clostridia	o__Lachnospirales	f__Lachnospiraceae	g__UBA11774	s__UBA11774 sp003507655
PD	PD12	bin20	MAG2144	d__Bacteria	p__Bacillota_A	c__Clostridia	o__Lachnospirales	f__Lachnospiraceae	g__UBA11774	s__UBA11774 sp003507655
PD	PD2	bin25	MAG1300	d__Bacteria	p__Bacillota_A	c__Clostridia	o__Lachnospirales	f__Lachnospiraceae	g__UBA11774	s__UBA11774 sp003507655
PD	PD22	bin47	MAG1988	d__Bacteria	p__Bacillota_A	c__Clostridia	o__Lachnospirales	f__Lachnospiraceae	g__UBA11774	s__UBA11774 sp003507655
PD	PD97	bin71	MAG1261	d__Bacteria	p__Bacillota_A	c__Clostridia	o__Lachnospirales	f__Lachnospiraceae	g__UBA11774	s__UBA11774 sp003507655
PD	PD14	bin26	MAG2041	d__Bacteria	p__Bacillota_A	c__Clostridia	o__Lachnospirales	f__Lachnospiraceae	g__UBA11774	s__UBA11774 sp003507655
PD	PD25	bin47	MAG484	d__Bacteria	p__Bacillota_A	c__Clostridia	o__Lachnospirales	f__Lachnospiraceae	g__UBA11774	s__UBA11774 sp003507655
PD	PD71	bin56	MAG757	d__Bacteria	p__Bacillota_A	c__Clostridia	o__Lachnospirales	f__Lachnospiraceae	g__Clostridium_Q	s__Clostridium_Q saccharolyticum_A
PD	PD94	bin14	MAG3354	d__Bacteria	p__Bacillota_A	c__Clostridia	o__Lachnospirales	f__Lachnospiraceae	g__Dorea_A	s__Dorea_A longicatena
PD	PD87	bin24	MAG277	d__Bacteria	p__Bacillota_A	c__Clostridia	o__Lachnospirales	f__Lachnospiraceae	g__Dorea_A	s__Dorea_A longicatena
PD	PD29	bin50	MAG1734	d__Bacteria	p__Bacillota_A	c__Clostridia	o__Lachnospirales	f__Lachnospiraceae	g__Dorea_A	s__Dorea_A longicatena
PD	PD44	bin27	MAG821	d__Bacteria	p__Bacillota_A	c__Clostridia	o__Lachnospirales	f__Lachnospiraceae	g__Oliverpabstia	s__Oliverpabstia intestinalis
PD	PD70	bin1	MAG1418	d__Bacteria	p__Bacillota_A	c__Clostridia	o__Lachnospirales	f__Lachnospiraceae	g__Blautia_A	s__Blautia_A sp900066335
PD	PD46	bin51	MAG3201	d__Bacteria	p__Bacillota_A	c__Clostridia	o__Lachnospirales	f__CAG-274	g__CAG-274	s__CAG-274 sp000432155
PD	PD44	bin34	MAG910	d__Bacteria	p__Bacillota_A	c__Clostridia	o__Lachnospirales	f__CAG-274	g__CAG-274	s__CAG-274 sp000432155
PD	PD37	bin34	MAG2401	d__Bacteria	p__Bacillota_A	c__Clostridia	o__Lachnospirales	f__CAG-274	g__CAG-274	s__CAG-274 sp000432155
PD	PD24	bin61	MAG2015	d__Bacteria	p__Bacillota_A	c__Clostridia	o__Lachnospirales	f__CAG-274	g__CAG-274	s__CAG-274 sp000432155
PD	PD19	bin18	MAG2046	d__Bacteria	p__Bacillota_A	c__Clostridia	o__Lachnospirales	f__Lachnospiraceae	g__Extibacter	s__Extibacter hylemonae
PD	PD64	bin19	MAG2455	d__Bacteria	p__Bacillota_A	c__Clostridia	o__Lachnospirales	f__Lachnospiraceae	g__Lachnoclostridium_B	s__Lachnoclostridium_B faecipullorum
PD	PD42	bin2	MAG127	d__Bacteria	p__Bacillota_A	c__Clostridia	o__Lachnospirales	f__Lachnospiraceae	g__Enterocloster	s__Enterocloster sp900541315
PD	PD11	bin4	MAG359	d__Bacteria	p__Bacillota_A	c__Clostridia	o__Lachnospirales	f__Lachnospiraceae	g__Enterocloster	s__Enterocloster sp900541315
PD	PD62	bin31	MAG1507	d__Bacteria	p__Bacillota_A	c__Clostridia	o__Lachnospirales	f__Lachnospiraceae	g__Enterocloster	s__Enterocloster sp900541315
PD	PD10	bin19	MAG554	d__Bacteria	p__Bacillota_A	c__Clostridia	o__Lachnospirales	f__Lachnospiraceae	g__Dorea	s__Dorea formicigenerans
PD	PD87	bin50	MAG3456	d__Bacteria	p__Bacillota_A	c__Clostridia	o__Lachnospirales	f__Lachnospiraceae	g__Dorea	s__Dorea formicigenerans
PD	PD32	bin48	MAG1286	d__Bacteria	p__Bacillota_A	c__Clostridia	o__Lachnospirales	f__Lachnospiraceae	g__Dorea	s__Dorea formicigenerans
PD	PD76	bin15	MAG2366	d__Bacteria	p__Bacillota_A	c__Clostridia	o__Lachnospirales	f__Lachnospiraceae	g__Pararoseburia	s__Pararoseburia lenta
PD	PD40	bin33	MAG2214	d__Bacteria	p__Bacillota_A	c__Clostridia	o__Lachnospirales	f__Lachnospiraceae	g__Pararoseburia	s__Pararoseburia lenta
PD	PD48	bin48	MAG1855	d__Bacteria	p__Bacillota_A	c__Clostridia	o__Lachnospirales	f__Lachnospiraceae	g__Pararoseburia	s__Pararoseburia lenta
PD	PD34	bin67	MAG1257	d__Bacteria	p__Bacillota_A	c__Clostridia	o__Lachnospirales	f__Lachnospiraceae	g__Pararoseburia	s__Pararoseburia lenta
PD	PD66	bin39	MAG2150	d__Bacteria	p__Bacillota_A	c__Clostridia	o__Lachnospirales	f__Lachnospiraceae	g__Pararoseburia	s__Pararoseburia lenta
PD	PD11	bin29	MAG327	d__Bacteria	p__Bacillota_A	c__Clostridia	o__Lachnospirales	f__Lachnospiraceae	g__Pararoseburia	s__Pararoseburia lenta
PD	PD87	bin61	MAG1373	d__Bacteria	p__Bacillota_A	c__Clostridia	o__Lachnospirales	f__Lachnospiraceae	g__CAG-95	s__CAG-95 sp000438155
PD	PD28	bin55	MAG3475	d__Bacteria	p__Bacillota_A	c__Clostridia	o__Lachnospirales	f__Lachnospiraceae	g__COE1	s__COE1 sp001916965
PD	PD25	bin63	MAG3346	d__Bacteria	p__Bacillota_A	c__Clostridia	o__Lachnospirales	f__Lachnospiraceae	g__Blautia_A	s__Blautia_A sp000436615
PD	PD28	bin21	MAG2458	d__Bacteria	p__Bacillota_A	c__Clostridia	o__Lachnospirales	f__Lachnospiraceae	g__Blautia_A	s__Blautia_A sp000436615
PD	PD87	bin39	MAG536	d__Bacteria	p__Bacillota_A	c__Clostridia	o__Lachnospirales	f__Lachnospiraceae	g__Blautia_A	s__Blautia_A sp000436615
PD	PD97	bin70	MAG1417	d__Bacteria	p__Bacillota_A	c__Clostridia	o__Lachnospirales	f__Lachnospiraceae	g__Copromonas	s__Copromonas sp000435795
PD	PD71	bin54	MAG2111	d__Bacteria	p__Bacillota_A	c__Clostridia	o__Lachnospirales	f__Lachnospiraceae	g__Copromonas	s__Copromonas sp000435795
PD	PD11	bin27	MAG915	d__Bacteria	p__Bacillota_A	c__Clostridia	o__Lachnospirales	f__Anaerotignaceae	g__Anaerotignum	s__Anaerotignum sp001304995
PD	PD97	bin48	MAG2065	d__Bacteria	p__Bacillota_A	c__Clostridia	o__Lachnospirales	f__Lachnospiraceae	g__Blautia_A	s__Blautia_A schinkii
PD	PD36	bin29	MAG878	d__Bacteria	p__Bacillota_A	c__Clostridia	o__Lachnospirales	f__Lachnospiraceae	g__Eubacterium_F	s__Eubacterium_F sp003491505
PD	PD28	bin42	MAG2676	d__Bacteria	p__Bacillota_A	c__Clostridia	o__Lachnospirales	f__Lachnospiraceae	g__Eubacterium_F	s__Eubacterium_F sp003491505
PD	PD68	bin23	MAG3271	d__Bacteria	p__Bacillota_A	c__Clostridia	o__Lachnospirales	f__Lachnospiraceae	g__Eubacterium_F	s__Eubacterium_F sp003491505

PD	PD94	bin33	MAG3283	d__Bacteria	p__Bacillota_A	c__Clostridia	o__Lachnospirales	f__Lachnospiraceae	g__Eubacterium_F	s__Eubacterium_F sp003491505
PD	PD32	bin74	MAG2982	d__Bacteria	p__Bacillota_A	c__Clostridia	o__Lachnospirales	f__Lachnospiraceae	g__Eubacterium_F	s__Eubacterium_F sp003491505
PD	PD9	bin21	MAG432	d__Bacteria	p__Bacillota_A	c__Clostridia	o__Lachnospirales	f__Lachnospiraceae	g__Eubacterium_F	s__Eubacterium_F sp003491505
PD	PD44	bin47	MAG1591	d__Bacteria	p__Bacillota_A	c__Clostridia	o__Lachnospirales	f__Lachnospiraceae	g__Blautia_A	s__Blautia_A massiliensis
PD	PD78	bin28	MAG1890	d__Bacteria	p__Bacillota_A	c__Clostridia	o__Lachnospirales	f__Lachnospiraceae	g__Blautia_A	s__Blautia_A massiliensis
PD	PD83	bin18	MAG2220	d__Bacteria	p__Bacillota_A	c__Clostridia	o__Lachnospirales	f__Lachnospiraceae	g__Blautia_A	s__Blautia_A massiliensis
PD	PD100	bin19	MAG866	d__Bacteria	p__Bacillota_A	c__Clostridia	o__Lachnospirales	f__Lachnospiraceae	g__Blautia_A	s__Blautia_A massiliensis
PD	PD73	bin7	MAG1450	d__Bacteria	p__Bacillota_A	c__Clostridia	o__Lachnospirales	f__Lachnospiraceae	g__Blautia_A	s__Blautia_A massiliensis
PD	PD82	bin19	MAG1722	d__Bacteria	p__Bacillota_A	c__Clostridia	o__Lachnospirales	f__Lachnospiraceae	g__Blautia_A	s__Blautia_A massiliensis
PD	PD87	bin11	MAG335	d__Bacteria	p__Bacillota_A	c__Clostridia	o__Lachnospirales	f__Lachnospiraceae	g__Coprococcus	s__Coprococcus eutactus
PD	PD17	bin14	MAG1846	d__Bacteria	p__Bacillota_A	c__Clostridia	o__Lachnospirales	f__Lachnospiraceae	g__Coprococcus	s__Coprococcus eutactus
PD	PD91	bin9	MAG2309	d__Bacteria	p__Bacillota_A	c__Clostridia	o__Lachnospirales	f__Lachnospiraceae	g__Eubacterium_I	s__Eubacterium_I ramulus
PD	PD77	bin52	MAG2640	d__Bacteria	p__Bacillota_A	c__Clostridia	o__Lachnospirales	f__Lachnospiraceae	g__Butyribacter	s__Butyribacter intestini
PD	PD28	bin51	MAG3507	d__Bacteria	p__Bacillota_A	c__Clostridia	o__Lachnospirales	f__Lachnospiraceae	g__Hominisplanchenecus	s__Hominisplanchenecus sp905214675
PD	PD36	bin41	MAG279	d__Bacteria	p__Bacillota_A	c__Clostridia	o__Lachnospirales	f__Lachnospiraceae	g__Blautia_A	s__Blautia_A faecis
PD	PD10	bin46	MAG1784	d__Bacteria	p__Bacillota_A	c__Clostridia	o__Lachnospirales	f__Lachnospiraceae	g__Blautia_A	s__Blautia_A faecis
PD	PD46	bin53	MAG888	d__Bacteria	p__Bacillota_A	c__Clostridia	o__Lachnospirales	f__Lachnospiraceae	g__Blautia_A	s__Blautia_A faecis
PD	PD81	bin11	MAG1493	d__Bacteria	p__Bacillota_A	c__Clostridia	o__Lachnospirales	f__Lachnospiraceae	g__Blautia_A	s__Blautia_A faecis
PD	PD27	bin20	MAG2583	d__Bacteria	p__Bacillota_A	c__Clostridia	o__Lachnospirales	f__Lachnospiraceae	g__Blautia_A	s__Blautia_A faecis
PD	PD95	bin36	MAG1806	d__Bacteria	p__Bacillota_A	c__Clostridia	o__Lachnospirales	f__Lachnospiraceae	g__Blautia_A	s__Blautia_A faecis
PD	PD87	bin26	MAG3089	d__Bacteria	p__Bacillota_A	c__Clostridia	o__Lachnospirales	f__Lachnospiraceae	g__Lachnoclostridium_B	s__Lachnoclostridium_B sp900066555
PD	PD19	bin36	MAG905	d__Bacteria	p__Bacillota_A	c__Clostridia	o__Lachnospirales	f__Lachnospiraceae	g__Blautia_A	s__Blautia_A sp021771755
PD	PD26	bin1	MAG202	d__Bacteria	p__Bacillota_A	c__Clostridia	o__Lachnospirales	f__Lachnospiraceae	g__Lachnospira	s__Lachnospira sp900316325
PD	PD97	bin25	MAG2703	d__Bacteria	p__Bacillota_A	c__Clostridia	o__Lachnospirales	f__Lachnospiraceae	g__Coprococcus_A	s__Coprococcus_A catus_A
PD	PD34	bin61	MAG2677	d__Bacteria	p__Bacillota_A	c__Clostridia	o__Lachnospirales	f__Lachnospiraceae	g__Coprococcus_A	s__Coprococcus_A catus_A
PD	PD98	bin24	MAG1731	d__Bacteria	p__Bacillota_A	c__Clostridia	o__Lachnospirales	f__Lachnospiraceae	g__Coprococcus_A	s__Coprococcus_A catus_A
PD	PD59	bin51	MAG3451	d__Bacteria	p__Bacillota_A	c__Clostridia	o__Lachnospirales	f__Lachnospiraceae	g__Eisenbergiella	s__Eisenbergiella porci
PD	PD9	bin60	MAG1388	d__Bacteria	p__Bacillota_A	c__Clostridia	o__Lachnospirales	f__Lachnospiraceae	g__Mediterraneibacter	s__Mediterraneibacter lactaris
PD	PD4	bin8	MAG2991	d__Bacteria	p__Bacillota_A	c__Clostridia	o__Lachnospirales	f__Lachnospiraceae	g__Mediterraneibacter	s__Mediterraneibacter lactaris
PD	PD81	bin23	MAG2480	d__Bacteria	p__Bacillota_A	c__Clostridia	o__Lachnospirales	f__Lachnospiraceae	g__Mediterraneibacter	s__Mediterraneibacter lactaris
PD	PD46	bin22	MAG2661	d__Bacteria	p__Bacillota_A	c__Clostridia	o__Lachnospirales	f__Lachnospiraceae	g__Mediterraneibacter	s__Mediterraneibacter lactaris
PD	PD71	bin38	MAG338	d__Bacteria	p__Bacillota_A	c__Clostridia	o__Lachnospirales	f__Lachnospiraceae	g__Enterocloster	s__Enterocloster aldenensis
PD	PD18	bin29	MAG1810	d__Bacteria	p__Bacillota_A	c__Clostridia	o__Lachnospirales	f__Lachnospiraceae	g__Sellimonas	s__Sellimonas avistercoris
PD	PD60	bin30	MAG938	d__Bacteria	p__Bacillota_A	c__Clostridia	o__Lachnospirales	f__Lachnospiraceae	g__Acetatifactor	s__Acetatifactor sp900066565
PD	PD68	bin6	MAG1477	d__Bacteria	p__Bacillota_A	c__Clostridia	o__Lachnospirales	f__Lachnospiraceae	g__Acetatifactor	s__Acetatifactor sp900066565
PD	PD3	bin6	MAG3492	d__Bacteria	p__Bacillota_A	c__Clostridia	o__Lachnospirales	f__Lachnospiraceae	g__Acetatifactor	s__Acetatifactor sp900066565
PD	PD48	bin67	MAG1798	d__Bacteria	p__Bacillota_A	c__Clostridia	o__Lachnospirales	f__Lachnospiraceae	g__Acetatifactor	s__Acetatifactor sp900066565
PD	PD45	bin22	MAG578	d__Bacteria	p__Bacillota_A	c__Clostridia	o__Lachnospirales	f__Lachnospiraceae	g__Acetatifactor	s__Acetatifactor sp900066565
PD	PD92	bin11	MAG3495	d__Bacteria	p__Bacillota_A	c__Clostridia	o__Lachnospirales	f__Lachnospiraceae	g__Acetatifactor	s__Acetatifactor sp900066565
PD	PD6	bin14	MAG1696	d__Bacteria	p__Bacillota_A	c__Clostridia	o__Lachnospirales	f__Lachnospiraceae	g__Acetatifactor	s__Acetatifactor sp900066565
PD	PD17	bin26	MAG29	d__Bacteria	p__Bacillota_A	c__Clostridia	o__Lachnospirales	f__Lachnospiraceae	g__Acetatifactor	s__Acetatifactor sp900066565
PD	PD81	bin44	MAG2550	d__Bacteria	p__Bacillota_A	c__Clostridia	o__Lachnospirales	f__Lachnospiraceae	g__Blautia	s__Blautia sp000432195
PD	PD73	bin53	MAG2713	d__Bacteria	p__Bacillota_A	c__Clostridia	o__Lachnospirales	f__Lachnospiraceae	g__UBA4285	s__UBA4285 sp900545225
PD	PD7	bin13	MAG2197	d__Bacteria	p__Bacillota_A	c__Clostridia	o__Lachnospirales	f__Lachnospiraceae	g__Eisenbergiella	s__Eisenbergiella tayi
PD	PD19	bin6	MAG1719	d__Bacteria	p__Bacillota_A	c__Clostridia	o__Lachnospirales	f__Lachnospiraceae	g__Eisenbergiella	s__Eisenbergiella tayi
PD	PD20	bin31	MAG1503	d__Bacteria	p__Bacillota_A	c__Clostridia	o__Lachnospirales	f__Lachnospiraceae	g__Lachnospira	s__Lachnospira rogosae
PD	PD77	bin62	MAG1718	d__Bacteria	p__Bacillota_A	c__Clostridia	o__Lachnospirales	f__Lachnospiraceae	g__Lachnospira	s__Lachnospira rogosae
PD	PD60	bin15	MAG3320	d__Bacteria	p__Bacillota_A	c__Clostridia	o__Lachnospirales	f__Lachnospiraceae	g__Lachnospira	s__Lachnospira rogosae
PD	PD82	bin31	MAG121	d__Bacteria	p__Bacillota_A	c__Clostridia	o__Lachnospirales	f__Lachnospiraceae	g__Lachnospira	s__Lachnospira rogosae
PD	PD78	bin16	MAG2510	d__Bacteria	p__Bacillota_A	c__Clostridia	o__Lachnospirales	f__Lachnospiraceae	g__Lachnospira	s__Lachnospira rogosae
PD	PD35	bin19	MAG1077	d__Bacteria	p__Bacillota_A	c__Clostridia	o__Lachnospirales	f__Lachnospiraceae	g__Lachnospira	s__Lachnospira rogosae

PD	PD29	bin52	MAG1268	d__Bacteria	p__Bacillota_A	c__Clostridia	o__Lachnospirales	f__Anaerotignaceae	g__Anaerotignum	s__Anaerotignum faecicola
PD	PD77	bin11	MAG35	d__Bacteria	p__Bacillota_A	c__Clostridia	o__Lachnospirales	f__Anaerotignaceae	g__Anaerotignum	s__Anaerotignum faecicola
PD	PD60	bin9	MAG352	d__Bacteria	p__Bacillota_A	c__Clostridia	o__Lachnospirales	f__Anaerotignaceae	g__Anaerotignum	s__Anaerotignum faecicola
PD	PD8	bin38	MAG1753	d__Bacteria	p__Bacillota_A	c__Clostridia	o__Lachnospirales	f__Anaerotignaceae	g__Anaerotignum	s__Anaerotignum faecicola
PD	PD37	bin25	MAG3450	d__Bacteria	p__Bacillota_A	c__Clostridia	o__Lachnospirales	f__Lachnospiraceae	g__Blautia	s__Blautia sp900541955
PD	PD71	bin2	MAG3463	d__Bacteria	p__Bacillota_A	c__Clostridia	o__Lachnospirales	f__Lachnospiraceae	g__Enterocloster	s__Enterocloster sp000431375
PD	PD23	bin10	MAG850	d__Bacteria	p__Bacillota_A	c__Clostridia	o__Lachnospirales	f__Lachnospiraceae	g__Enterocloster	s__Enterocloster sp000431375
PD	PD18	bin13	MAG3351	d__Bacteria	p__Bacillota_A	c__Clostridia	o__Lachnospirales	f__Lachnospiraceae	g__Mediterraneibacter	s__Mediterraneibacter excrementipullorum
PD	PD44	bin25	MAG2389	d__Bacteria	p__Bacillota_A	c__Clostridia	o__Lachnospirales	f__Lachnospiraceae	g__Blautia_A	s__Blautia_A intestinalis
PD	PD1	bin9	MAG2408	d__Bacteria	p__Bacillota_A	c__Clostridia	o__Lachnospirales	f__Lachnospiraceae	g__Copromonas	s__Copromonas sp900066535
PD	PD26	bin11	MAG1309	d__Bacteria	p__Bacillota_A	c__Clostridia	o__Lachnospirales	f__Lachnospiraceae	g__Copromonas	s__Copromonas sp900066535
PD	PD13	bin7	MAG56	d__Bacteria	p__Bacillota_A	c__Clostridia	o__Monoglobales	f__Monoglobaceae	g__UMGS1820	s__UMGS1820 sp900555375
PD	PD11	bin8	MAG1135	d__Bacteria	p__Bacillota_A	c__Clostridia	o__Oscillospirales	f__Ruminococcaceae	g__Faecalibacterium	s__Faecalibacterium prausnitzii_D
PD	PD20	bin11	MAG2256	d__Bacteria	p__Bacillota_A	c__Clostridia	o__Oscillospirales	f__Ruminococcaceae	g__Faecalibacterium	s__Faecalibacterium prausnitzii_D
PD	PD60	bin36	MAG1780	d__Bacteria	p__Bacillota_A	c__Clostridia	o__Oscillospirales	f__Ruminococcaceae	g__Faecalibacterium	s__Faecalibacterium prausnitzii_D
PD	PD92	bin17	MAG3301	d__Bacteria	p__Bacillota_A	c__Clostridia	o__Oscillospirales	f__Ruminococcaceae	g__Faecalibacterium	s__Faecalibacterium prausnitzii_D
PD	PD77	bin19	MAG2709	d__Bacteria	p__Bacillota_A	c__Clostridia	o__Oscillospirales	f__Ruminococcaceae	g__Faecalibacterium	s__Faecalibacterium prausnitzii_D
PD	PD78	bin26	MAG2673	d__Bacteria	p__Bacillota_A	c__Clostridia	o__Oscillospirales	f__Ruminococcaceae	g__Faecalibacterium	s__Faecalibacterium prausnitzii_D
PD	PD97	bin39	MAG2468	d__Bacteria	p__Bacillota_A	c__Clostridia	o__Oscillospirales	f__Ruminococcaceae	g__Faecalibacterium	s__Faecalibacterium prausnitzii_D
PD	PD37	bin50	MAG1883	d__Bacteria	p__Bacillota_A	c__Clostridia	o__Oscillospirales	f__Ruminococcaceae	g__Faecalibacterium	s__Faecalibacterium prausnitzii_D
PD	PD58	bin26	MAG562	d__Bacteria	p__Bacillota_A	c__Clostridia	o__Oscillospirales	f__Ruminococcaceae	g__Faecalibacterium	s__Faecalibacterium prausnitzii_D
PD	PD3	bin40	MAG3413	d__Bacteria	p__Bacillota_A	c__Clostridia	o__Oscillospirales	f__Ruminococcaceae	g__Faecalibacterium	s__Faecalibacterium prausnitzii_D
PD	PD95	bin35	MAG744	d__Bacteria	p__Bacillota_A	c__Clostridia	o__Oscillospirales	f__Ruminococcaceae	g__Faecalibacterium	s__Faecalibacterium prausnitzii_D
PD	PD1	bin1	MAG3365	d__Bacteria	p__Bacillota_A	c__Clostridia	o__Oscillospirales	f__Ruminococcaceae	g__Faecalibacterium	s__Faecalibacterium prausnitzii_D
PD	PD62	bin40	MAG1421	d__Bacteria	p__Bacillota_A	c__Clostridia	o__Oscillospirales	f__Ruminococcaceae	g__Faecalibacterium	s__Faecalibacterium prausnitzii_D
PD	PD66	bin35	MAG8	d__Bacteria	p__Bacillota_A	c__Clostridia	o__Oscillospirales	f__Ruminococcaceae	g__Faecalibacterium	s__Faecalibacterium prausnitzii_D
PD	PD46	bin60	MAG236	d__Bacteria	p__Bacillota_A	c__Clostridia	o__Oscillospirales	f__Ruminococcaceae	g__Faecalibacterium	s__Faecalibacterium prausnitzii_D
PD	PD18	bin20	MAG2587	d__Bacteria	p__Bacillota_A	c__Clostridia	o__Oscillospirales	f__Butyricicoccaceae	g__Agathobaculum	s__Agathobaculum intestinigallinarum
PD	PD59	bin58	MAG3245	d__Bacteria	p__Bacillota_A	c__Clostridia	o__Oscillospirales	f__Acutalibacteraceae	g__UMGS1071	s__UMGS1071 sp900541905
PD	PD72	bin41	MAG2476	d__Bacteria	p__Bacillota_A	c__Clostridia	o__Oscillospirales	f__Acutalibacteraceae	g__UMGS1071	s__UMGS1071 sp900541905
PD	PD94	bin7	MAG1560	d__Bacteria	p__Bacillota_A	c__Clostridia	o__Oscillospirales	f__CAG-382	g__Woodwardibium	s__Woodwardibium gallinarum
PD	PD41	bin56	MAG1176	d__Bacteria	p__Bacillota_A	c__Clostridia	o__Oscillospirales	f__CAG-382	g__UBA1206	s__UBA1206 sp000433115
PD	PD72	bin5	MAG84	d__Bacteria	p__Bacillota_A	c__Clostridia	o__Oscillospirales	f__CAG-382	g__Woodwardibium	s__Woodwardibium sp900760275
PD	PD59	bin3	MAG3037	d__Bacteria	p__Bacillota_A	c__Clostridia	o__Oscillospirales	f__Acutalibacteraceae	g__Acutalibacter	s__Acutalibacter sp900548545
PD	PD71	bin62	MAG2374	d__Bacteria	p__Bacillota_A	c__Clostridia	o__Oscillospirales	f__Ruminococcaceae	g__Faecalibacterium	s__Faecalibacterium prausnitzii
PD	PD8	bin18	MAG1321	d__Bacteria	p__Bacillota_A	c__Clostridia	o__Oscillospirales	f__Ruminococcaceae	g__Faecalibacterium	s__Faecalibacterium prausnitzii
PD	PD72	bin32	MAG2771	d__Bacteria	p__Bacillota_A	c__Clostridia	o__Oscillospirales	f__Ruminococcaceae	g__Faecalibacterium	s__Faecalibacterium prausnitzii
PD	PD53	bin13	MAG300	d__Bacteria	p__Bacillota_A	c__Clostridia	o__Oscillospirales	f__Ruminococcaceae	g__Faecalibacterium	s__Faecalibacterium prausnitzii
PD	PD100	bin44	MAG224	d__Bacteria	p__Bacillota_A	c__Clostridia	o__Oscillospirales	f__Ruminococcaceae	g__Faecalibacterium	s__Faecalibacterium prausnitzii
PD	PD35	bin17	MAG62	d__Bacteria	p__Bacillota_A	c__Clostridia	o__Oscillospirales	f__Ruminococcaceae	g__Faecalibacterium	s__Faecalibacterium prausnitzii
PD	PD45	bin31	MAG1791	d__Bacteria	p__Bacillota_A	c__Clostridia	o__Oscillospirales	f__Ruminococcaceae	g__Faecalibacterium	s__Faecalibacterium prausnitzii
PD	PD31	bin42	MAG1365	d__Bacteria	p__Bacillota_A	c__Clostridia	o__Oscillospirales	f__Ruminococcaceae	g__Faecalibacterium	s__Faecalibacterium prausnitzii
PD	PD82	bin7	MAG194	d__Bacteria	p__Bacillota_A	c__Clostridia	o__Oscillospirales	f__Ruminococcaceae	g__Faecalibacterium	s__Faecalibacterium prausnitzii
PD	PD62	bin60	MAG960	d__Bacteria	p__Bacillota_A	c__Clostridia	o__Oscillospirales	f__Ruminococcaceae	g__Faecalibacterium	s__Faecalibacterium prausnitzii
PD	PD48	bin26	MAG509	d__Bacteria	p__Bacillota_A	c__Clostridia	o__Oscillospirales	f__Ruminococcaceae	g__Faecalibacterium	s__Faecalibacterium prausnitzii
PD	PD28	bin30	MAG157	d__Bacteria	p__Bacillota_A	c__Clostridia	o__Oscillospirales	f__Ruminococcaceae	g__Faecalibacterium	s__Faecalibacterium prausnitzii
PD	PD91	bin12	MAG305	d__Bacteria	p__Bacillota_A	c__Clostridia	o__Oscillospirales	f__Ruminococcaceae	g__Faecalibacterium	s__Faecalibacterium prausnitzii
PD	PD24	bin45	MAG2596	d__Bacteria	p__Bacillota_A	c__Clostridia	o__Oscillospirales	f__Ruminococcaceae	g__Faecalibacterium	s__Faecalibacterium prausnitzii
PD	PD16	bin39	MAG248	d__Bacteria	p__Bacillota_A	c__Clostridia	o__Oscillospirales	f__Ruminococcaceae	g__Faecalibacterium	s__Faecalibacterium prausnitzii
PD	PD53	bin15	MAG1871	d__Bacteria	p__Bacillota_A	c__Clostridia	o__Oscillospirales	f__Oscillospiraceae	g__CAG-103	s__CAG-103 sp900317855
PD	PD75	bin25	MAG1924	d__Bacteria	p__Bacillota_A	c__Clostridia	o__Oscillospirales	f__Ruminococcaceae	g__Faecalibacterium	s__Faecalibacterium duncaniae

PD	PD26	bin7	MAG2858	d__Bacteria	p__Bacillota_A	c__Clostridia	o__Oscillospirales	f__Ruminococcaceae	g__Faecalibacterium	s__Faecalibacterium duncaniae
PD	PD48	bin13	MAG690	d__Bacteria	p__Bacillota_A	c__Clostridia	o__Oscillospirales	f__Ruminococcaceae	g__Faecalibacterium	s__Faecalibacterium duncaniae
PD	PD8	bin52	MAG1962	d__Bacteria	p__Bacillota_A	c__Clostridia	o__Oscillospirales	f__Ruminococcaceae	g__Faecalibacterium	s__Faecalibacterium duncaniae
PD	PD69	bin1	MAG2822	d__Bacteria	p__Bacillota_A	c__Clostridia	o__Oscillospirales	f__Ruminococcaceae	g__Faecalibacterium	s__Faecalibacterium duncaniae
PD	PD50	bin49	MAG2452	d__Bacteria	p__Bacillota_A	c__Clostridia	o__Oscillospirales	f__Ruminococcaceae	g__Faecalibacterium	s__Faecalibacterium duncaniae
PD	PD44	bin9	MAG365	d__Bacteria	p__Bacillota_A	c__Clostridia	o__Oscillospirales	f__Ruminococcaceae	g__Faecalibacterium	s__Faecalibacterium duncaniae
PD	PD46	bin43	MAG3250	d__Bacteria	p__Bacillota_A	c__Clostridia	o__Oscillospirales	f__Ruminococcaceae	g__Faecalibacterium	s__Faecalibacterium duncaniae
PD	PD32	bin34	MAG3155	d__Bacteria	p__Bacillota_A	c__Clostridia	o__Oscillospirales	f__Ruminococcaceae	g__Faecalibacterium	s__Faecalibacterium duncaniae
PD	PD25	bin10	MAG842	d__Bacteria	p__Bacillota_A	c__Clostridia	o__Oscillospirales	f__Ruminococcaceae	g__Faecalibacterium	s__Faecalibacterium duncaniae
PD	PD35	bin22	MAG1381	d__Bacteria	p__Bacillota_A	c__Clostridia	o__Oscillospirales	f__Ruminococcaceae	g__Faecalibacterium	s__Faecalibacterium duncaniae
PD	PD23	bin3	MAG1997	d__Bacteria	p__Bacillota_A	c__Clostridia	o__Oscillospirales	f__Ruminococcaceae	g__Faecalibacterium	s__Faecalibacterium duncaniae
PD	PD47	bin59	MAG2647	d__Bacteria	p__Bacillota_A	c__Clostridia	o__Oscillospirales	f__Ruminococcaceae	g__Faecalibacterium	s__Faecalibacterium duncaniae
PD	PD34	bin2	MAG2568	d__Bacteria	p__Bacillota_A	c__Clostridia	o__Oscillospirales	f__Ruminococcaceae	g__Faecalibacterium	s__Faecalibacterium duncaniae
PD	PD28	bin22	MAG3479	d__Bacteria	p__Bacillota_A	c__Clostridia	o__Oscillospirales	f__Ruminococcaceae	g__Faecalibacterium	s__Faecalibacterium duncaniae
PD	PD64	bin87	MAG2883	d__Bacteria	p__Bacillota_A	c__Clostridia	o__Oscillospirales	f__Ruminococcaceae	g__Faecalibacterium	s__Faecalibacterium duncaniae
PD	PD43	bin36	MAG1559	d__Bacteria	p__Bacillota_A	c__Clostridia	o__Oscillospirales	f__Ruminococcaceae	g__Faecalibacterium	s__Faecalibacterium duncaniae
PD	PD24	bin44	MAG1006	d__Bacteria	p__Bacillota_A	c__Clostridia	o__Oscillospirales	f__Ruminococcaceae	g__Faecalibacterium	s__Faecalibacterium duncaniae
PD	PD57	bin24	MAG1139	d__Bacteria	p__Bacillota_A	c__Clostridia	o__Oscillospirales	f__Oscillospiraceae	g__Vescimonas	s__Vescimonas fastidiosa
PD	PD72	bin14	MAG3488	d__Bacteria	p__Bacillota_A	c__Clostridia	o__Oscillospirales	f__UMGS1783	g__UMGS1783	s__UMGS1783 sp900555065
PD	PD25	bin36	MAG2139	d__Bacteria	p__Bacillota_A	c__Clostridia	o__Oscillospirales	f__Oscillospiraceae	g__Limivicinus	s__Limivicinus sp900547315
PD	PD72	bin54	MAG1302	d__Bacteria	p__Bacillota_A	c__Clostridia	o__Oscillospirales	f__UBA644	g__UBA644	s__UBA644 sp014872655
PD	PD100	bin16	MAG1266	d__Bacteria	p__Bacillota_A	c__Clostridia	o__Oscillospirales	f__Acutalibacteraceae	g__Ruminococcus_E	s__Ruminococcus_E bromii_B
PD	PD23	bin20	MAG961	d__Bacteria	p__Bacillota_A	c__Clostridia	o__Oscillospirales	f__Acutalibacteraceae	g__Ruminococcus_E	s__Ruminococcus_E bromii_B
PD	PD36	bin46	MAG706	d__Bacteria	p__Bacillota_A	c__Clostridia	o__Oscillospirales	f__Acutalibacteraceae	g__Ruminococcus_E	s__Ruminococcus_E bromii_B
PD	PD53	bin2	MAG2030	d__Bacteria	p__Bacillota_A	c__Clostridia	o__Oscillospirales	f__Acutalibacteraceae	g__Ruminococcus_E	s__Ruminococcus_E bromii_B
PD	PD56	bin29	MAG2519	d__Bacteria	p__Bacillota_A	c__Clostridia	o__Oscillospirales	f__Acutalibacteraceae	g__Ruminococcus_E	s__Ruminococcus_E bromii_B
PD	PD66	bin20	MAG45	d__Bacteria	p__Bacillota_A	c__Clostridia	o__Oscillospirales	f__Acutalibacteraceae	g__Ruminococcus_E	s__Ruminococcus_E bromii_B
PD	PD8	bin1	MAG2598	d__Bacteria	p__Bacillota_A	c__Clostridia	o__Oscillospirales	f__Acutalibacteraceae	g__Ruminococcus_E	s__Ruminococcus_E bromii_B
PD	PD92	bin57	MAG2098	d__Bacteria	p__Bacillota_A	c__Clostridia	o__Oscillospirales	f__Acutalibacteraceae	g__Ruminococcus_E	s__Ruminococcus_E bromii_B
PD	PD95	bin10	MAG2576	d__Bacteria	p__Bacillota_A	c__Clostridia	o__Oscillospirales	f__Acutalibacteraceae	g__Ruminococcus_E	s__Ruminococcus_E bromii_B
PD	PD17	bin11	MAG1212	d__Bacteria	p__Bacillota_A	c__Clostridia	o__Oscillospirales	f__Acutalibacteraceae	g__Ruminococcus_E	s__Ruminococcus_E bromii_B
PD	PD81	bin6	MAG2067	d__Bacteria	p__Bacillota_A	c__Clostridia	o__Oscillospirales	f__Acutalibacteraceae	g__Ruminococcus_E	s__Ruminococcus_E bromii_B
PD	PD94	bin65	MAG2345	d__Bacteria	p__Bacillota_A	c__Clostridia	o__Oscillospirales	f__Acutalibacteraceae	g__Ruminococcus_E	s__Ruminococcus_E bromii_B
PD	PD9	bin3	MAG3194	d__Bacteria	p__Bacillota_A	c__Clostridia	o__Oscillospirales	f__Acutalibacteraceae	g__Ruminococcus_E	s__Ruminococcus_E bromii_B
PD	PD70	bin32	MAG1441	d__Bacteria	p__Bacillota_A	c__Clostridia	o__Oscillospirales	f__Acutalibacteraceae	g__Ruminococcus_E	s__Ruminococcus_E bromii_B
PD	PD73	bin24	MAG217	d__Bacteria	p__Bacillota_A	c__Clostridia	o__Oscillospirales	f__Acutalibacteraceae	g__Ruminococcus_E	s__Ruminococcus_E bromii_B
PD	PD14	bin59	MAG2341	d__Bacteria	p__Bacillota_A	c__Clostridia	o__Oscillospirales	f__Acutalibacteraceae	g__Ruminococcus_E	s__Ruminococcus_E bromii_B
PD	PD2	bin42	MAG2689	d__Bacteria	p__Bacillota_A	c__Clostridia	o__Oscillospirales	f__Acutalibacteraceae	g__Ruminococcus_E	s__Ruminococcus_E bromii_B
PD	PD59	bin30	MAG158	d__Bacteria	p__Bacillota_A	c__Clostridia	o__Oscillospirales	f__Acutalibacteraceae	g__Ruminococcus_E	s__Ruminococcus_E bromii_B
PD	PD47	bin9	MAG1844	d__Bacteria	p__Bacillota_A	c__Clostridia	o__Oscillospirales	f__Acutalibacteraceae	g__Ruminococcus_E	s__Ruminococcus_E bromii_B
PD	PD64	bin75	MAG532	d__Bacteria	p__Bacillota_A	c__Clostridia	o__Oscillospirales	f__Acutalibacteraceae	g__Ruminococcus_E	s__Ruminococcus_E bromii_B
PD	PD67	bin24	MAG675	d__Bacteria	p__Bacillota_A	c__Clostridia	o__Oscillospirales	f__Acutalibacteraceae	g__Ruminococcus_E	s__Ruminococcus_E bromii_B
PD	PD78	bin13	MAG790	d__Bacteria	p__Bacillota_A	c__Clostridia	o__Oscillospirales	f__Butyricicoccaceae	g__Agathobaculum	s__Agathobaculum butyriciproducens
PD	PD44	bin3	MAG2571	d__Bacteria	p__Bacillota_A	c__Clostridia	o__Oscillospirales	f__Butyricicoccaceae	g__Agathobaculum	s__Agathobaculum butyriciproducens
PD	PD76	bin39	MAG1061	d__Bacteria	p__Bacillota_A	c__Clostridia	o__Oscillospirales	f__Butyricicoccaceae	g__Agathobaculum	s__Agathobaculum butyriciproducens
PD	PD85	bin9	MAG2944	d__Bacteria	p__Bacillota_A	c__Clostridia	o__Oscillospirales	f__Butyricicoccaceae	g__Agathobaculum	s__Agathobaculum butyriciproducens
PD	PD37	bin2	MAG446	d__Bacteria	p__Bacillota_A	c__Clostridia	o__Oscillospirales	f__Butyricicoccaceae	g__Agathobaculum	s__Agathobaculum butyriciproducens
PD	PD66	bin1	MAG209	d__Bacteria	p__Bacillota_A	c__Clostridia	o__Oscillospirales	f__Butyricicoccaceae	g__Agathobaculum	s__Agathobaculum butyriciproducens
PD	PD19	bin17	MAG2824	d__Bacteria	p__Bacillota_A	c__Clostridia	o__Oscillospirales	f__Butyricicoccaceae	g__Agathobaculum	s__Agathobaculum butyriciproducens
PD	PD42	bin18	MAG3164	d__Bacteria	p__Bacillota_A	c__Clostridia	o__Oscillospirales	f__Butyricicoccaceae	g__Agathobaculum	s__Agathobaculum butyriciproducens
PD	PD62	bin42	MAG239	d__Bacteria	p__Bacillota_A	c__Clostridia	o__Oscillospirales	f__Butyricicoccaceae	g__Agathobaculum	s__Agathobaculum butyriciproducens

PD	PD83	bin36	MAG918	d__Bacteria	p__Bacillota_A	c__Clostridia	o__Oscillospirales	f__Butyricicoccaceae	g__Agathobaculum	s__Agathobaculum butyriciproducens
PD	PD82	bin24	MAG3009	d__Bacteria	p__Bacillota_A	c__Clostridia	o__Oscillospirales	f__Butyricicoccaceae	g__Agathobaculum	s__Agathobaculum butyriciproducens
PD	PD47	bin46	MAG1785	d__Bacteria	p__Bacillota_A	c__Clostridia	o__Oscillospirales	f__Butyricicoccaceae	g__Agathobaculum	s__Agathobaculum butyriciproducens
PD	PD61	bin22	MAG1612	d__Bacteria	p__Bacillota_A	c__Clostridia	o__Oscillospirales	f__Butyricicoccaceae	g__Agathobaculum	s__Agathobaculum butyriciproducens
PD	PD71	bin57	MAG1845	d__Bacteria	p__Bacillota_A	c__Clostridia	o__Oscillospirales	f__Butyricicoccaceae	g__Agathobaculum	s__Agathobaculum butyriciproducens
PD	PD9	bin25	MAG2496	d__Bacteria	p__Bacillota_A	c__Clostridia	o__Oscillospirales	f__Butyricicoccaceae	g__Agathobaculum	s__Agathobaculum butyriciproducens
PD	PD58	bin80	MAG2527	d__Bacteria	p__Bacillota_A	c__Clostridia	o__Oscillospirales	f__Butyricicoccaceae	g__Agathobaculum	s__Agathobaculum butyriciproducens
PD	PD35	bin48	MAG2099	d__Bacteria	p__Bacillota_A	c__Clostridia	o__Oscillospirales	f__Butyricicoccaceae	g__Agathobaculum	s__Agathobaculum butyriciproducens
PD	PD39	bin3	MAG1357	d__Bacteria	p__Bacillota_A	c__Clostridia	o__Oscillospirales	f__Butyricicoccaceae	g__Agathobaculum	s__Agathobaculum butyriciproducens
PD	PD32	bin55	MAG2590	d__Bacteria	p__Bacillota_A	c__Clostridia	o__Oscillospirales	f__Ruminococcaceae	g__Ruminococcus_D	s__Ruminococcus_D sp900539095
PD	PD57	bin44	MAG1830	d__Bacteria	p__Bacillota_A	c__Clostridia	o__Oscillospirales	f__Oscillospiraceae	g__Onthomonas	s__Onthomonas sp003150485
PD	PD73	bin65	MAG1342	d__Bacteria	p__Bacillota_A	c__Clostridia	o__Oscillospirales	f__CAG-272	g__UMGS911	s__UMGS911 sp900545935
PD	PD12	bin11	MAG2024	d__Bacteria	p__Bacillota_A	c__Clostridia	o__Oscillospirales	f__CAG-272	g__UMGS911	s__UMGS911 sp900545935
PD	PD82	bin55	MAG1912	d__Bacteria	p__Bacillota_A	c__Clostridia	o__Oscillospirales	f__Ruminococcaceae	g__Gemmiger	s__Gemmiger qucibialis
PD	PD39	bin43	MAG2913	d__Bacteria	p__Bacillota_A	c__Clostridia	o__Oscillospirales	f__Ruminococcaceae	g__Gemmiger	s__Gemmiger qucibialis
PD	PD67	bin26	MAG1042	d__Bacteria	p__Bacillota_A	c__Clostridia	o__Oscillospirales	f__Ruminococcaceae	g__Gemmiger	s__Gemmiger qucibialis
PD	PD11	bin28	MAG2236	d__Bacteria	p__Bacillota_A	c__Clostridia	o__Oscillospirales	f__Ruminococcaceae	g__Gemmiger	s__Gemmiger qucibialis
PD	PD17	bin15	MAG1598	d__Bacteria	p__Bacillota_A	c__Clostridia	o__Oscillospirales	f__Ruminococcaceae	g__Gemmiger	s__Gemmiger qucibialis
PD	PD45	bin45	MAG148	d__Bacteria	p__Bacillota_A	c__Clostridia	o__Oscillospirales	f__Ruminococcaceae	g__Gemmiger	s__Gemmiger qucibialis
PD	PD6	bin16	MAG2834	d__Bacteria	p__Bacillota_A	c__Clostridia	o__Oscillospirales	f__Ruminococcaceae	g__Gemmiger	s__Gemmiger qucibialis
PD	PD70	bin22	MAG727	d__Bacteria	p__Bacillota_A	c__Clostridia	o__Oscillospirales	f__Ruminococcaceae	g__Gemmiger	s__Gemmiger qucibialis
PD	PD77	bin39	MAG2178	d__Bacteria	p__Bacillota_A	c__Clostridia	o__Oscillospirales	f__Ruminococcaceae	g__Gemmiger	s__Gemmiger qucibialis
PD	PD97	bin58	MAG1649	d__Bacteria	p__Bacillota_A	c__Clostridia	o__Oscillospirales	f__Ruminococcaceae	g__Gemmiger	s__Gemmiger qucibialis
PD	PD32	bin78	MAG486	d__Bacteria	p__Bacillota_A	c__Clostridia	o__Oscillospirales	f__Ruminococcaceae	g__Gemmiger	s__Gemmiger qucibialis
PD	PD69	bin56	MAG171	d__Bacteria	p__Bacillota_A	c__Clostridia	o__Oscillospirales	f__Ruminococcaceae	g__Gemmiger	s__Gemmiger qucibialis
PD	PD53	bin9	MAG112	d__Bacteria	p__Bacillota_A	c__Clostridia	o__Oscillospirales	f__Ruminococcaceae	g__Gemmiger	s__Gemmiger qucibialis
PD	PD86	bin1	MAG2034	d__Bacteria	p__Bacillota_A	c__Clostridia	o__Oscillospirales	f__Ruminococcaceae	g__Gemmiger	s__Gemmiger qucibialis
PD	PD9	bin15	MAG319	d__Bacteria	p__Bacillota_A	c__Clostridia	o__Oscillospirales	f__Ruminococcaceae	g__Gemmiger	s__Gemmiger qucibialis
PD	PD47	bin5	MAG1242	d__Bacteria	p__Bacillota_A	c__Clostridia	o__Oscillospirales	f__Ruminococcaceae	g__Gemmiger	s__Gemmiger qucibialis
PD	PD64	bin94	MAG1106	d__Bacteria	p__Bacillota_A	c__Clostridia	o__Oscillospirales	f__Ruminococcaceae	g__Gemmiger	s__Gemmiger qucibialis
PD	PD22	bin51	MAG2432	d__Bacteria	p__Bacillota_A	c__Clostridia	o__Oscillospirales	f__Ruminococcaceae	g__Gemmiger	s__Gemmiger qucibialis
PD	PD100	bin42	MAG204	d__Bacteria	p__Bacillota_A	c__Clostridia	o__Oscillospirales	f__Ruminococcaceae	g__Gemmiger	s__Gemmiger qucibialis
PD	PD66	bin30	MAG2730	d__Bacteria	p__Bacillota_A	c__Clostridia	o__Oscillospirales	f__Ruminococcaceae	g__Gemmiger	s__Gemmiger qucibialis
PD	PD31	bin21	MAG317	d__Bacteria	p__Bacillota_A	c__Clostridia	o__Oscillospirales	f__Ruminococcaceae	g__Gemmiger	s__Gemmiger qucibialis
PD	PD5	bin49	MAG876	d__Bacteria	p__Bacillota_A	c__Clostridia	o__Oscillospirales	f__Ruminococcaceae	g__Gemmiger	s__Gemmiger qucibialis
PD	PD75	bin30	MAG3171	d__Bacteria	p__Bacillota_A	c__Clostridia	o__Oscillospirales	f__Ruminococcaceae	g__Gemmiger	s__Gemmiger qucibialis
PD	PD40	bin5	MAG1565	d__Bacteria	p__Bacillota_A	c__Clostridia	o__Oscillospirales	f__Ruminococcaceae	g__Gemmiger	s__Gemmiger qucibialis
PD	PD13	bin1	MAG2665	d__Bacteria	p__Bacillota_A	c__Clostridia	o__Oscillospirales	f__Ruminococcaceae	g__Gemmiger	s__Gemmiger qucibialis
PD	PD28	bin47	MAG265	d__Bacteria	p__Bacillota_A	c__Clostridia	o__Oscillospirales	f__Ruminococcaceae	g__Gemmiger	s__Gemmiger qucibialis
PD	PD44	bin31	MAG3242	d__Bacteria	p__Bacillota_A	c__Clostridia	o__Oscillospirales	f__Ruminococcaceae	g__Gemmiger	s__Gemmiger qucibialis
PD	PD62	bin23	MAG1185	d__Bacteria	p__Bacillota_A	c__Clostridia	o__Oscillospirales	f__Ruminococcaceae	g__Gemmiger	s__Gemmiger qucibialis
PD	PD68	bin70	MAG1425	d__Bacteria	p__Bacillota_A	c__Clostridia	o__Oscillospirales	f__Ruminococcaceae	g__Gemmiger	s__Gemmiger qucibialis
PD	PD24	bin56	MAG1353	d__Bacteria	p__Bacillota_A	c__Clostridia	o__Oscillospirales	f__Ruminococcaceae	g__Gemmiger	s__Gemmiger qucibialis
PD	PD94	bin21	MAG3244	d__Bacteria	p__Bacillota_A	c__Clostridia	o__Oscillospirales	f__Ruminococcaceae	g__Gemmiger	s__Gemmiger qucibialis
PD	PD14	bin20	MAG3141	d__Bacteria	p__Bacillota_A	c__Clostridia	o__Oscillospirales	f__Ruminococcaceae	g__Gemmiger	s__Gemmiger qucibialis
PD	PD4	bin13	MAG639	d__Bacteria	p__Bacillota_A	c__Clostridia	o__Oscillospirales	f__Ruminococcaceae	g__Gemmiger	s__Gemmiger qucibialis
PD	PD20	bin34	MAG323	d__Bacteria	p__Bacillota_A	c__Clostridia	o__Oscillospirales	f__Ruminococcaceae	g__Gemmiger	s__Gemmiger qucibialis
PD	PD35	bin12	MAG188	d__Bacteria	p__Bacillota_A	c__Clostridia	o__Oscillospirales	f__Ruminococcaceae	g__Gemmiger	s__Gemmiger qucibialis
PD	PD36	bin2	MAG2346	d__Bacteria	p__Bacillota_A	c__Clostridia	o__Oscillospirales	f__Acutalibacteraceae	g__UMGS1474	s__UMGS1474 sp900547105
PD	PD29	bin44	MAG2484	d__Bacteria	p__Bacillota_A	c__Clostridia	o__Oscillospirales	f__Oscillospiraceae	g__Scatomorpha	s__Scatomorpha intestinigallinarum
PD	PD57	bin19	MAG2363	d__Bacteria	p__Bacillota_A	c__Clostridia	o__Oscillospirales	f__Oscillospiraceae	g__Scatomorpha	s__Scatomorpha intestinigallinarum

PD	PD18	bin4	MAG593	d__Bacteria	p__Bacillota_A	c__Clostridia	o__Oscillospirales	f__Oscillospiraceae	g__CAG-103	s__CAG-103 sp004556965		
PD	PD19	bin12	MAG2747	d__Bacteria	p__Bacillota_A	c__Clostridia	o__Oscillospirales	f__Ruminococcaceae	g__Faecalibacterium	s__Faecalibacterium prausnitzii_I		
PD	PD76	bin24	MAG2264	d__Bacteria	p__Bacillota_A	c__Clostridia	o__Oscillospirales	f__Ruminococcaceae	g__Faecalibacterium	s__Faecalibacterium prausnitzii_I		
PD	PD26	bin15	MAG2625	d__Bacteria	p__Bacillota_A	c__Clostridia	o__Oscillospirales	f__Ruminococcaceae	g__Faecalibacterium	s__Faecalibacterium sp934525735		
PD	PD57	bin26	MAG912	d__Bacteria	p__Bacillota_A	c__Clostridia	o__Oscillospirales	f__Butyricicoccaceae	g__Pseudobutyricicoccus	s__Pseudobutyricicoccus sp003477405		
PD	PD50	bin51	MAG3193	d__Bacteria	p__Bacillota_A	c__Clostridia	o__Oscillospirales	f__Oscillospiraceae	g__CAG-170	s__CAG-170 sp003516765		
PD	PD14	bin19	MAG627	d__Bacteria	p__Bacillota_A	c__Clostridia	o__Oscillospirales	f__Ruminococcaceae	g__Ruminiclostridium_E	s__Ruminiclostridium_E siraeum		
PD	PD24	bin8	MAG3490	d__Bacteria	p__Bacillota_A	c__Clostridia	o__Oscillospirales	f__Ruminococcaceae	g__Ruminiclostridium_E	s__Ruminiclostridium_E siraeum		
PD	PD48	bin31	MAG434	d__Bacteria	p__Bacillota_A	c__Clostridia	o__Oscillospirales	f__Ruminococcaceae	g__Ruminiclostridium_E	s__Ruminiclostridium_E siraeum		
PD	PD32	bin59	MAG2556	d__Bacteria	p__Bacillota_A	c__Clostridia	o__Oscillospirales	f__Ruminococcaceae	g__Ruminiclostridium_E	s__Ruminiclostridium_E siraeum		
PD	PD5	bin29	MAG535	d__Bacteria	p__Bacillota_A	c__Clostridia	o__Oscillospirales	f__Ruminococcaceae	g__Ruminiclostridium_E	s__Ruminiclostridium_E siraeum		
PD	PD87	bin46	MAG136	d__Bacteria	p__Bacillota_A	c__Clostridia	o__Oscillospirales	f__Ruminococcaceae	g__Ruminiclostridium_E	s__Ruminiclostridium_E siraeum		
PD	PD27	bin26	MAG2836	d__Bacteria	p__Bacillota_A	c__Clostridia	o__Oscillospirales	f__Ruminococcaceae	g__Ruminiclostridium_E	s__Ruminiclostridium_E siraeum		
PD	PD46	bin77	MAG1192	d__Bacteria	p__Bacillota_A	c__Clostridia	o__Oscillospirales	f__Ruminococcaceae	g__Ruminiclostridium_E	s__Ruminiclostridium_E siraeum		
PD	PD82	bin30	MAG244	d__Bacteria	p__Bacillota_A	c__Clostridia	o__Oscillospirales	f__Ruminococcaceae	g__Ruminococcus_C	s__Ruminococcus_C callidus		
PD	PD68	bin30	MAG2767	d__Bacteria	p__Bacillota_A	c__Clostridia	o__Oscillospirales	f__Ruminococcaceae	g__Ruminococcus_C	s__Ruminococcus_C callidus		
PD	PD8	bin23	MAG1323	d__Bacteria	p__Bacillota_A	c__Clostridia	o__Oscillospirales	f__Ruminococcaceae	g__Ruminococcus_C	s__Ruminococcus_C callidus		
PD	PD56	bin43	MAG501	d__Bacteria	p__Bacillota_A	c__Clostridia	o__Oscillospirales	f__Ruminococcaceae	g__Ruminococcus_C	s__Ruminococcus_C callidus		
PD	PD13	bin29	MAG1026	d__Bacteria	p__Bacillota_A	c__Clostridia	o__Oscillospirales	f__Ruminococcaceae	g__Ruminococcus_C	s__Ruminococcus_C callidus		
PD	PD70	bin8	MAG580	d__Bacteria	p__Bacillota_A	c__Clostridia	o__Oscillospirales	f__Oscillospiraceae	g__Evtepia	s__Evtepia excrementipullorum		
PD	PD17	bin4	MAG2691	d__Bacteria	p__Bacillota_A	c__Clostridia	o__Oscillospirales	f__Oscillospiraceae	g__ER4	s__ER4 sp000765235		
PD	PD68	bin54	MAG3248	d__Bacteria	p__Bacillota_A	c__Clostridia	o__Oscillospirales	f__Oscillospiraceae	g__ER4	s__ER4 sp000765235		
PD	PD75	bin45	MAG1234	d__Bacteria	p__Bacillota_A	c__Clostridia	o__Oscillospirales	f__Oscillospiraceae	g__ER4	s__ER4 sp000765235		
PD	PD35	bin39	MAG3407	d__Bacteria	p__Bacillota_A	c__Clostridia	o__Oscillospirales	f__Oscillospiraceae	g__ER4	s__ER4 sp000765235		
PD	PD62	bin52	MAG260	d__Bacteria	p__Bacillota_A	c__Clostridia	o__Oscillospirales	f__Oscillospiraceae	g__ER4	s__ER4 sp000765235		
PD	PD39	bin31	MAG1013	d__Bacteria	p__Bacillota_A	c__Clostridia	o__Oscillospirales	f__Oscillospiraceae	g__ER4	s__ER4 sp000765235		
PD	PD97	bin29	MAG806	d__Bacteria	p__Bacillota_A	c__Clostridia	o__Oscillospirales	f__Oscillospiraceae	g__ER4	s__ER4 sp000765235		
PD	PD50	bin23	MAG941	d__Bacteria	p__Bacillota_A	c__Clostridia	o__Oscillospirales	f__Oscillospiraceae	g__ER4	s__ER4 sp000765235		
PD	PD34	bin25	MAG2679	d__Bacteria	p__Bacillota_A	c__Clostridia	o__Oscillospirales	f__Oscillospiraceae	g__ER4	s__ER4 sp000765235		
PD	PD61	bin39	MAG2295	d__Bacteria	p__Bacillota_A	c__Clostridia	o__Oscillospirales	f__Oscillospiraceae	g__ER4	s__ER4 sp000765235		
PD	PD13	bin11	MAG1156	d__Bacteria	p__Bacillota_A	c__Clostridia	o__Oscillospirales	f__Oscillospiraceae	g__ER4	s__ER4 sp000765235		
PD	PD47	bin56	MAG2049	d__Bacteria	p__Bacillota_A	c__Clostridia	o__Oscillospirales	f__Oscillospiraceae	g__ER4	s__ER4 sp000765235		
PD	PD9	bin69	MAG2031	d__Bacteria	p__Bacillota_A	c__Clostridia	o__Oscillospirales	f__Oscillospiraceae	g__ER4	s__ER4 sp000765235		
PD	PD12	bin39	MAG1897	d__Bacteria	p__Bacillota_A	c__Clostridia	o__Oscillospirales	f__Oscillospiraceae	g__ER4	s__ER4 sp000765235		
PD	PD91	bin32	MAG189	d__Bacteria	p__Bacillota_A	c__Clostridia	o__Oscillospirales	f__Oscillospiraceae	g__ER4	s__ER4 sp000765235		
PD	PD70	bin36	MAG836	d__Bacteria	p__Bacillota_A	c__Clostridia	o__Oscillospirales	f__Oscillospiraceae	g__ER4	s__ER4 sp000765235		
PD	PD76	bin23	MAG1399	d__Bacteria	p__Bacillota_A	c__Clostridia	o__Oscillospirales	f__Oscillospiraceae	g__ER4	s__ER4 sp000765235		
PD	PD19	bin16	MAG1432	d__Bacteria	p__Bacillota_A	c__Clostridia	o__Oscillospirales	f__Acutalibacteraceae	g__Eubacterium_R	s__Unclassified		
PD	PD19	bin35	MAG3455	d__Bacteria	p__Bacillota_A	c__Clostridia	o__Oscillospirales	f__Acutalibacteraceae	g__Ruminococcus_E	s__Ruminococcus_E sp003438075		
PD	PD3	bin49	MAG2010	d__Bacteria	p__Bacillota_A	c__Clostridia	o__Oscillospirales	f__Acutalibacteraceae	g__Ruminococcus_E	s__Ruminococcus_E sp003438075		
PD	PD24	bin24	MAG1349	d__Bacteria	p__Bacillota_A	c__Clostridia	o__Oscillospirales	f__Acutalibacteraceae	g__Ruminococcus_E	s__Ruminococcus_E sp003438075		
PD	PD36	bin31	MAG3160	d__Bacteria	p__Bacillota_A	c__Clostridia	o__Oscillospirales	f__Acutalibacteraceae	g__Eubacterium_R	s__Eubacterium_R sp000434995		
PD	PD4	bin2	MAG2890	d__Bacteria	p__Bacillota_A	c__Clostridia	o__Oscillospirales	f__Acutalibacteraceae	g__Eubacterium_R	s__Eubacterium_R sp000434995		
PD	PD47	bin4	MAG330	d__Bacteria	p__Bacillota_A	c__Clostridia	o__Oscillospirales	f__Acutalibacteraceae	g__Eubacterium_R	s__Eubacterium_R sp000434995		
PD	PD61	bin16	MAG2351	d__Bacteria	p__Bacillota_A	c__Clostridia	o__Oscillospirales	f__Acutalibacteraceae	g__Eubacterium_R	s__Eubacterium_R sp000434995		
PD	PD16	bin5	MAG800	d__Bacteria	p__Bacillota_A	c__Clostridia	o__Oscillospirales	f__Acutalibacteraceae	g__Eubacterium_R	s__Eubacterium_R sp000434995		
PD	PD14	bin17	MAG1011	d__Bacteria	p__Bacillota_A	c__Clostridia	o__Oscillospirales	f__Acutalibacteraceae	g__Eubacterium_R	s__Eubacterium_R sp000434995		
PD	PD46	bin4	MAG2799	d__Bacteria	p__Bacillota_A	c__Clostridia	o__Oscillospirales	f__Acutalibacteraceae	g__Eubacterium_R	s__Eubacterium_R sp000434995		
PD	PD50	bin25	MAG630	d__Bacteria	p__Bacillota_A	c__Clostridia	o__Oscillospirales	f__Acutalibacteraceae	g__Eubacterium_R	s__Eubacterium_R sp000434995		
PD	PD44	bin38	MAG3100	d__Bacteria	p__Bacillota_A	c__Clostridia	o__Oscillospirales	f__Acutalibacteraceae	g__Ruminococcus_E	s__Ruminococcus_E sp003526955		
PD	PD77	bin34	MAG1422	d__Bacteria	p__Bacillota_A	c__Clostridia	o__Oscillospirales	f__Acutalibacteraceae	g__Ruminococcus_E	s__Ruminococcus_E sp003526955		

PD	PD29	bin60	MAG2983	d__Bacteria	p__Bacillota_A	c__Clostridia	o__Oscillospirales	f__Acutalibacteraceae	g__Ruminococcus_E	s__Ruminococcus_E sp003526955
PD	PD75	bin31	MAG230	d__Bacteria	p__Bacillota_A	c__Clostridia	o__Oscillospirales	f__Acutalibacteraceae	g__Ruminococcus_E	s__Ruminococcus_E sp003526955
PD	PD32	bin32	MAG2172	d__Bacteria	p__Bacillota_A	c__Clostridia	o__Oscillospirales	f__Acutalibacteraceae	g__Ruminococcus_E	s__Ruminococcus_E sp003526955
PD	PD41	bin9	MAG79	d__Bacteria	p__Bacillota_A	c__Clostridia	o__Oscillospirales	f__Acutalibacteraceae	g__Ruminococcus_E	s__Ruminococcus_E sp003526955
PD	PD22	bin3	MAG2825	d__Bacteria	p__Bacillota_A	c__Clostridia	o__Oscillospirales	f__Acutalibacteraceae	g__Ruminococcus_E	s__Ruminococcus_E sp003526955
PD	PD5	bin9	MAG366	d__Bacteria	p__Bacillota_A	c__Clostridia	o__Oscillospirales	f__Acutalibacteraceae	g__Ruminococcus_E	s__Ruminococcus_E sp003526955
PD	PD14	bin2	MAG3092	d__Bacteria	p__Bacillota_A	c__Clostridia	o__Oscillospirales	f__Acutalibacteraceae	g__Ruminococcus_E	s__Ruminococcus_E sp003526955
PD	PD67	bin16	MAG167	d__Bacteria	p__Bacillota_A	c__Clostridia	o__Oscillospirales	f__Acutalibacteraceae	g__Ruminococcus_E	s__Ruminococcus_E sp003526955
PD	PD45	bin2	MAG3291	d__Bacteria	p__Bacillota_A	c__Clostridia	o__Oscillospirales	f__Acutalibacteraceae	g__UBA1417	s__UBA1417 sp003531055
PD	PD57	bin45	MAG1875	d__Bacteria	p__Bacillota_A	c__Clostridia	o__Oscillospirales	f__Acutalibacteraceae	g__UBA1417	s__UBA1417 sp003531055
PD	PD39	bin41	MAG2485	d__Bacteria	p__Bacillota_A	c__Clostridia	o__Oscillospirales	f__Acutalibacteraceae	g__UBA1417	s__UBA1417 sp003531055
PD	PD34	bin32	MAG3121	d__Bacteria	p__Bacillota_A	c__Clostridia	o__Oscillospirales	f__Acutalibacteraceae	g__UBA1417	s__UBA1417 sp003531055
PD	PD12	bin72	MAG1816	d__Bacteria	p__Bacillota_A	c__Clostridia	o__Oscillospirales	f__Acutalibacteraceae	g__UBA1417	s__UBA1417 sp003531055
PD	PD62	bin30	MAG796	d__Bacteria	p__Bacillota_A	c__Clostridia	o__Oscillospirales	f__Acutalibacteraceae	g__Ruminococcus_E	s__Ruminococcus_E sp934476515
PD	PD37	bin3	MAG2335	d__Bacteria	p__Bacillota_A	c__Clostridia	o__Oscillospirales	f__Acutalibacteraceae	g__Ruminococcus_E	s__Ruminococcus_E sp934476515
PD	PD69	bin7	MAG20	d__Bacteria	p__Bacillota_A	c__Clostridia	o__Oscillospirales	f__Acutalibacteraceae	g__Ruminococcus_E	s__Ruminococcus_E sp934476515
PD	PD82	bin32	MAG296	d__Bacteria	p__Bacillota_A	c__Clostridia	o__Oscillospirales	f__Acutalibacteraceae	g__Ruminococcus_E	s__Ruminococcus_E sp934476515
PD	PD27	bin16	MAG603	d__Bacteria	p__Bacillota_A	c__Clostridia	o__Oscillospirales	f__Acutalibacteraceae	g__Ruminococcus_E	s__Ruminococcus_E sp934476515
PD	PD64	bin15	MAG3024	d__Bacteria	p__Bacillota_A	c__Clostridia	o__Oscillospirales	f__Acutalibacteraceae	g__Eubacterium_R	s__Eubacterium_R sp000436835
PD	PD66	bin4	MAG1221	d__Bacteria	p__Bacillota_A	c__Clostridia	o__Oscillospirales	f__Acutalibacteraceae	g__Eubacterium_R	s__Eubacterium_R sp000436835
PD	PD41	bin62	MAG3462	d__Bacteria	p__Bacillota_A	c__Clostridia	o__Oscillospirales	f__Acutalibacteraceae	g__Eubacterium_R	s__Eubacterium_R sp000436835
PD	PD12	bin52	MAG381	d__Bacteria	p__Bacillota_A	c__Clostridia	o__Oscillospirales	f__Acutalibacteraceae	g__Eubacterium_R	s__Eubacterium_R sp000436835
PD	PD5	bin46	MAG42	d__Bacteria	p__Bacillota_A	c__Clostridia	o__Oscillospirales	f__Acutalibacteraceae	g__Eubacterium_R	s__Eubacterium_R sp000436835
PD	PD24	bin35	MAG2507	d__Bacteria	p__Bacillota_A	c__Clostridia	o__Oscillospirales	f__Acutalibacteraceae	g__Eubacterium_R	s__Eubacterium_R sp000436835
PD	PD100	bin18	MAG1001	d__Bacteria	p__Bacillota_A	c__Clostridia	o__Oscillospirales	f__Acutalibacteraceae	g__Eubacterium_R	s__Eubacterium_R sp000436835
PD	PD68	bin13	MAG835	d__Bacteria	p__Bacillota_A	c__Clostridia	o__Oscillospirales	f__Acutalibacteraceae	g__Eubacterium_R	s__Eubacterium_R sp000436835
PD	PD7	bin23	MAG200	d__Bacteria	p__Bacillota_A	c__Clostridia	o__Oscillospirales	f__Ruminococcaceae	g__Anaerotruncus	s__Anaerotruncus massiliensis
PD	PD95	bin16	MAG2751	d__Bacteria	p__Bacillota_A	c__Clostridia	o__Oscillospirales	f__Butyricicoccaceae	g__Agathobaculum	s__Agathobaculum sp003481705
PD	PD45	bin7	MAG885	d__Bacteria	p__Bacillota_A	c__Clostridia	o__Oscillospirales	f__Butyricicoccaceae	g__Agathobaculum	s__Agathobaculum sp003481705
PD	PD64	bin14	MAG803	d__Bacteria	p__Bacillota_A	c__Clostridia	o__Oscillospirales	f__Butyricicoccaceae	g__Agathobaculum	s__Agathobaculum sp003481705
PD	PD44	bin55	MAG985	d__Bacteria	p__Bacillota_A	c__Clostridia	o__Oscillospirales	f__Butyricicoccaceae	g__Agathobaculum	s__Agathobaculum sp003481705
PD	PD9	bin20	MAG2379	d__Bacteria	p__Bacillota_A	c__Clostridia	o__Oscillospirales	f__Butyricicoccaceae	g__Agathobaculum	s__Agathobaculum sp003481705
PD	PD23	bin18	MAG741	d__Bacteria	p__Bacillota_A	c__Clostridia	o__Oscillospirales	f__Ruminococcaceae	g__Ruminococcus_D	s__Ruminococcus_D bicirculans
PD	PD47	bin48	MAG1265	d__Bacteria	p__Bacillota_A	c__Clostridia	o__Oscillospirales	f__Ruminococcaceae	g__Ruminococcus_D	s__Ruminococcus_D bicirculans
PD	PD11	bin32	MAG334	d__Bacteria	p__Bacillota_A	c__Clostridia	o__Oscillospirales	f__Ruminococcaceae	g__Ruminococcus_D	s__Ruminococcus_D bicirculans
PD	PD57	bin70	MAG1019	d__Bacteria	p__Bacillota_A	c__Clostridia	o__Oscillospirales	f__Ruminococcaceae	g__Ruminococcus_D	s__Ruminococcus_D bicirculans
PD	PD82	bin50	MAG1587	d__Bacteria	p__Bacillota_A	c__Clostridia	o__Oscillospirales	f__Ruminococcaceae	g__Ruminococcus_D	s__Ruminococcus_D bicirculans
PD	PD17	bin23	MAG196	d__Bacteria	p__Bacillota_A	c__Clostridia	o__Oscillospirales	f__Ruminococcaceae	g__Ruminococcus_D	s__Ruminococcus_D bicirculans
PD	PD6	bin32	MAG2744	d__Bacteria	p__Bacillota_A	c__Clostridia	o__Oscillospirales	f__Ruminococcaceae	g__Ruminococcus_D	s__Ruminococcus_D bicirculans
PD	PD97	bin35	MAG3395	d__Bacteria	p__Bacillota_A	c__Clostridia	o__Oscillospirales	f__Ruminococcaceae	g__Ruminococcus_D	s__Ruminococcus_D bicirculans
PD	PD36	bin8	MAG488	d__Bacteria	p__Bacillota_A	c__Clostridia	o__Oscillospirales	f__Ruminococcaceae	g__Ruminococcus_D	s__Ruminococcus_D bicirculans
PD	PD69	bin11	MAG807	d__Bacteria	p__Bacillota_A	c__Clostridia	o__Oscillospirales	f__Ruminococcaceae	g__Ruminococcus_D	s__Ruminococcus_D bicirculans
PD	PD29	bin54	MAG415	d__Bacteria	p__Bacillota_A	c__Clostridia	o__Oscillospirales	f__Ruminococcaceae	g__Ruminococcus_D	s__Ruminococcus_D bicirculans
PD	PD3	bin25	MAG252	d__Bacteria	p__Bacillota_A	c__Clostridia	o__Oscillospirales	f__Ruminococcaceae	g__Ruminococcus_D	s__Ruminococcus_D bicirculans
PD	PD46	bin38	MAG3207	d__Bacteria	p__Bacillota_A	c__Clostridia	o__Oscillospirales	f__Ruminococcaceae	g__Ruminococcus_D	s__Ruminococcus_D bicirculans
PD	PD64	bin56	MAG3223	d__Bacteria	p__Bacillota_A	c__Clostridia	o__Oscillospirales	f__Ruminococcaceae	g__Ruminococcus_D	s__Ruminococcus_D bicirculans
PD	PD29	bin9	MAG694	d__Bacteria	p__Bacillota_A	c__Clostridia	o__Oscillospirales	f__Acutalibacteraceae	g__UBA737	s__Unclassified
PD	PD85	bin23	MAG1045	d__Bacteria	p__Bacillota_A	c__Clostridia	o__Oscillospirales	f__Ruminococcaceae	g__Faecalibacterium	s__Unclassified
PD	PD99	bin19	MAG3226	d__Bacteria	p__Bacillota_A	c__Clostridia	o__Oscillospirales	f__Ruminococcaceae	g__Faecalibacterium	s__Unclassified
PD	PD10	bin16	MAG1742	d__Bacteria	p__Bacillota_A	c__Clostridia	o__Oscillospirales	f__Oscillospiraceae	g__CAG-170	s__CAG-170 sp900549635
PD	PD25	bin72	MAG873	d__Bacteria	p__Bacillota_A	c__Clostridia	o__Oscillospirales	f__Ruminococcaceae	g__Gemmiger	s__Gemmiger sp900539695

PD	PD35	bin4	MAG3278	d__Bacteria	p__Bacillota_A	c__Clostridia	o__Oscillospirales	f__Ruminococcaceae	g__Gemmiger	s__Gemmiger sp900539695
PD	PD8	bin2	MAG3094	d__Bacteria	p__Bacillota_A	c__Clostridia	o__Oscillospirales	f__Ruminococcaceae	g__Gemmiger	s__Gemmiger sp900539695
PD	PD99	bin15	MAG156	d__Bacteria	p__Bacillota_A	c__Clostridia	o__Oscillospirales	f__Ruminococcaceae	g__Gemmiger	s__Gemmiger sp900539695
PD	PD29	bin51	MAG1736	d__Bacteria	p__Bacillota_A	c__Clostridia	o__Oscillospirales	f__Ruminococcaceae	g__Gemmiger	s__Gemmiger sp900539695
PD	PD13	bin63	MAG3191	d__Bacteria	p__Bacillota_A	c__Clostridia	o__Oscillospirales	f__Ruminococcaceae	g__Gemmiger	s__Gemmiger sp900539695
PD	PD12	bin24	MAG3396	d__Bacteria	p__Bacillota_A	c__Clostridia	o__Oscillospirales	f__Ruminococcaceae	g__Gemmiger	s__Gemmiger sp900539695
PD	PD6	bin33	MAG2453	d__Bacteria	p__Bacillota_A	c__Clostridia	o__Oscillospirales	f__Ruminococcaceae	g__Gemmiger	s__Gemmiger sp900539695
PD	PD57	bin60	MAG2973	d__Bacteria	p__Bacillota_A	c__Clostridia	o__Oscillospirales	f__Ruminococcaceae	g__Gemmiger	s__Gemmiger sp900539695
PD	PD58	bin13	MAG601	d__Bacteria	p__Bacillota_A	c__Clostridia	o__Oscillospirales	f__Ruminococcaceae	g__Gemmiger	s__Gemmiger sp900539695
PD	PD6	bin31	MAG1783	d__Bacteria	p__Bacillota_A	c__Clostridia	o__Oscillospirales	f__Ruminococcaceae	g__Faecalibacterium	s__Faecalibacterium sp905215595
PD	PD5	bin4	MAG597	d__Bacteria	p__Bacillota_A	c__Clostridia	o__Oscillospirales	f__Oscillospiraceae	g__Dysosmobacter	s__Dysosmobacter sp001916835
PD	PD98	bin1	MAG3414	d__Bacteria	p__Bacillota_A	c__Clostridia	o__Oscillospirales	f__Oscillospiraceae	g__Faecousia	s__Faecousia sp000434635
PD	PD70	bin37	MAG1054	d__Bacteria	p__Bacillota_A	c__Clostridia	o__Oscillospirales	f__Oscillospiraceae	g__Faecousia	s__Faecousia sp000434635
PD	PD64	bin62	MAG1941	d__Bacteria	p__Bacillota_A	c__Clostridia	o__Oscillospirales	f__Oscillospiraceae	g__Faecousia	s__Faecousia sp000434635
PD	PD57	bin62	MAG1412	d__Bacteria	p__Bacillota_A	c__Clostridia	o__Oscillospirales	f__Oscillospiraceae	g__Faecousia	s__Faecousia sp000434635
PD	PD68	bin14	MAG423	d__Bacteria	p__Bacillota_A	c__Clostridia	o__Oscillospirales	f__Oscillospiraceae	g__Faecousia	s__Faecousia sp000434635
PD	PD25	bin43	MAG1528	d__Bacteria	p__Bacillota_A	c__Clostridia	o__Oscillospirales	f__Ruminococcaceae	g__Gemmiger	s__Gemmiger sp900540775
PD	PD45	bin36	MAG1633	d__Bacteria	p__Bacillota_A	c__Clostridia	o__Oscillospirales	f__Oscillospiraceae	g__CAG-170	s__CAG-170 sp900553545
PD	PD25	bin82	MAG1426	d__Bacteria	p__Bacillota_A	c__Clostridia	o__Oscillospirales	f__Oscillospiraceae	g__Faecousia	s__Faecousia sp000435995
PD	PD98	bin14	MAG2514	d__Bacteria	p__Bacillota_A	c__Clostridia	o__Oscillospirales	f__Oscillospiraceae	g__Faecousia	s__Faecousia sp000435995
PD	PD37	bin6	MAG940	d__Bacteria	p__Bacillota_A	c__Clostridia	o__Oscillospirales	f__Ruminococcaceae	g__Avimicrobium	s__Avimicrobium caecorum
PD	PD45	bin58	MAG2437	d__Bacteria	p__Bacillota_A	c__Clostridia	o__Oscillospirales	f__CAG-382	g__UMGS1387	s__UMGS1387 sp900551295
PD	PD5	bin42	MAG667	d__Bacteria	p__Bacillota_A	c__Clostridia	o__Oscillospirales	f__Ruminococcaceae	g__Faecalibacterium	s__Faecalibacterium prausnitzii_E
PD	PD2	bin8	MAG2223	d__Bacteria	p__Bacillota_A	c__Clostridia	o__Oscillospirales	f__Ruminococcaceae	g__Faecalibacterium	s__Faecalibacterium prausnitzii_E
PD	PD12	bin53	MAG1414	d__Bacteria	p__Bacillota_A	c__Clostridia	o__Oscillospirales	f__Ruminococcaceae	g__Faecalibacterium	s__Faecalibacterium prausnitzii_E
PD	PD62	bin64	MAG930	d__Bacteria	p__Bacillota_A	c__Clostridia	o__Oscillospirales	f__Ruminococcaceae	g__Faecalibacterium	s__Faecalibacterium prausnitzii_E
PD	PD47	bin10	MAG2082	d__Bacteria	p__Bacillota_A	c__Clostridia	o__Oscillospirales	f__Ruminococcaceae	g__Faecalibacterium	s__Faecalibacterium prausnitzii_E
PD	PD67	bin17	MAG367	d__Bacteria	p__Bacillota_A	c__Clostridia	o__Oscillospirales	f__Ruminococcaceae	g__Faecalibacterium	s__Faecalibacterium prausnitzii_E
PD	PD44	bin53	MAG1328	d__Bacteria	p__Bacillota_A	c__Clostridia	o__Oscillospirales	f__Acutalibacteraceae	g__CAG-217	s__CAG-217 sp000436335
PD	PD62	bin15	MAG1860	d__Bacteria	p__Bacillota_A	c__Clostridia	o__Oscillospirales	f__Acutalibacteraceae	g__CAG-217	s__CAG-217 sp000436335
PD	PD68	bin36	MAG2820	d__Bacteria	p__Bacillota_A	c__Clostridia	o__Oscillospirales	f__Acutalibacteraceae	g__CAG-217	s__CAG-217 sp000436335
PD	PD77	bin33	MAG717	d__Bacteria	p__Bacillota_A	c__Clostridia	o__Oscillospirales	f__Acutalibacteraceae	g__CAG-217	s__CAG-217 sp000436335
PD	PD25	bin15	MAG2577	d__Bacteria	p__Bacillota_A	c__Clostridia	o__Oscillospirales	f__Acutalibacteraceae	g__CAG-217	s__CAG-217 sp000436335
PD	PD6	bin2	MAG1938	d__Bacteria	p__Bacillota_A	c__Clostridia	o__Oscillospirales	f__Acutalibacteraceae	g__CAG-217	s__CAG-217 sp000436335
PD	PD75	bin6	MAG865	d__Bacteria	p__Bacillota_A	c__Clostridia	o__Oscillospirales	f__Acutalibacteraceae	g__CAG-217	s__CAG-217 sp000436335
PD	PD94	bin44	MAG2692	d__Bacteria	p__Bacillota_A	c__Clostridia	o__Oscillospirales	f__Acutalibacteraceae	g__CAG-217	s__CAG-217 sp000436335
PD	PD14	bin14	MAG2748	d__Bacteria	p__Bacillota_A	c__Clostridia	o__Oscillospirales	f__Acutalibacteraceae	g__CAG-217	s__CAG-217 sp000436335
PD	PD32	bin6	MAG2997	d__Bacteria	p__Bacillota_A	c__Clostridia	o__Oscillospirales	f__Acutalibacteraceae	g__CAG-217	s__CAG-217 sp000436335
PD	PD57	bin33	MAG1501	d__Bacteria	p__Bacillota_A	c__Clostridia	o__Oscillospirales	f__Acutalibacteraceae	g__CAG-217	s__CAG-217 sp000436335
PD	PD48	bin75	MAG848	d__Bacteria	p__Bacillota_A	c__Clostridia	o__Oscillospirales	f__Acutalibacteraceae	g__CAG-217	s__CAG-217 sp000436335
PD	PD98	bin49	MAG826	d__Bacteria	p__Bacillota_A	c__Clostridia	o__Oscillospirales	f__Acutalibacteraceae	g__CAG-217	s__CAG-217 sp000436335
PD	PD3	bin24	MAG1053	d__Bacteria	p__Bacillota_A	c__Clostridia	o__Oscillospirales	f__Acutalibacteraceae	g__CAG-217	s__CAG-217 sp000436335
PD	PD70	bin28	MAG1482	d__Bacteria	p__Bacillota_A	c__Clostridia	o__Oscillospirales	f__Acutalibacteraceae	g__CAG-217	s__CAG-217 sp000436335
PD	PD10	bin31	MAG739	d__Bacteria	p__Bacillota_A	c__Clostridia	o__Oscillospirales	f__Acutalibacteraceae	g__CAG-217	s__CAG-217 sp000436335
PD	PD73	bin41	MAG1546	d__Bacteria	p__Bacillota_A	c__Clostridia	o__Oscillospirales	f__Acutalibacteraceae	g__CAG-217	s__CAG-217 sp000436335
PD	PD28	bin29	MAG1995	d__Bacteria	p__Bacillota_A	c__Clostridia	o__Oscillospirales	f__Acutalibacteraceae	g__CAG-217	s__CAG-217 sp000436335
PD	PD57	bin11	MAG383	d__Bacteria	p__Bacillota_A	c__Clostridia	o__Oscillospirales	f__Acutalibacteraceae	g__Pseudoruminococcus	s__Pseudoruminococcus massiliensis
PD	PD46	bin45	MAG2288	d__Bacteria	p__Bacillota_A	c__Clostridia	o__Oscillospirales	f__Acutalibacteraceae	g__Pseudoruminococcus	s__Pseudoruminococcus massiliensis
PD	PD39	bin45	MAG1523	d__Bacteria	p__Bacillota_A	c__Clostridia	o__Oscillospirales	f__Acutalibacteraceae	g__Pseudoruminococcus	s__Pseudoruminococcus massiliensis
PD	PD95	bin23	MAG1663	d__Bacteria	p__Bacillota_A	c__Clostridia	o__Oscillospirales	f__Ruminococcaceae	g__Ruthenibacterium	s__Ruthenibacterium lactatiformans
PD	PD40	bin31	MAG769	d__Bacteria	p__Bacillota_A	c__Clostridia	o__Oscillospirales	f__Ruminococcaceae	g__Ruthenibacterium	s__Ruthenibacterium lactatiformans

PD	PD16	bin56	MAG916	d__Bacteria	p__Bacillota_A	c__Clostridia	o__Oscillospirales	f__Ruminococcaceae	g__Ruthenibacterium	s__Ruthenibacterium lactatiformans
PD	PD50	bin28	MAG3493	d__Bacteria	p__Bacillota_A	c__Clostridia	o__Oscillospirales	f__Ruminococcaceae	g__Ruthenibacterium	s__Ruthenibacterium lactatiformans
PD	PD9	bin62	MAG2380	d__Bacteria	p__Bacillota_A	c__Clostridia	o__Oscillospirales	f__Ruminococcaceae	g__Ruthenibacterium	s__Ruthenibacterium lactatiformans
PD	PD98	bin25	MAG3399	d__Bacteria	p__Bacillota_A	c__Clostridia	o__Oscillospirales	f__Ruminococcaceae	g__Ruthenibacterium	s__Ruthenibacterium lactatiformans
PD	PD37	bin21	MAG2134	d__Bacteria	p__Bacillota_A	c__Clostridia	o__Oscillospirales	f__Ruminococcaceae	g__Ruthenibacterium	s__Ruthenibacterium lactatiformans
PD	PD83	bin16	MAG1036	d__Bacteria	p__Bacillota_A	c__Clostridia	o__Oscillospirales	f__Ruminococcaceae	g__Ruthenibacterium	s__Ruthenibacterium lactatiformans
PD	PD48	bin9	MAG1258	d__Bacteria	p__Bacillota_A	c__Clostridia	o__Oscillospirales	f__Ruminococcaceae	g__Ruthenibacterium	s__Ruthenibacterium lactatiformans
PD	PD39	bin24	MAG3071	d__Bacteria	p__Bacillota_A	c__Clostridia	o__Oscillospirales	f__Ruminococcaceae	g__Ruthenibacterium	s__Ruthenibacterium lactatiformans
PD	PD62	bin63	MAG701	d__Bacteria	p__Bacillota_A	c__Clostridia	o__Oscillospirales	f__Ruminococcaceae	g__Ruthenibacterium	s__Ruthenibacterium lactatiformans
PD	PD56	bin26	MAG2534	d__Bacteria	p__Bacillota_A	c__Clostridia	o__Oscillospirales	f__Ruminococcaceae	g__Ruthenibacterium	s__Ruthenibacterium lactatiformans
PD	PD6	bin27	MAG2186	d__Bacteria	p__Bacillota_A	c__Clostridia	o__Oscillospirales	f__Ruminococcaceae	g__Ruthenibacterium	s__Ruthenibacterium lactatiformans
PD	PD82	bin20	MAG2013	d__Bacteria	p__Bacillota_A	c__Clostridia	o__Oscillospirales	f__Ruminococcaceae	g__Ruthenibacterium	s__Ruthenibacterium lactatiformans
PD	PD8	bin60	MAG1112	d__Bacteria	p__Bacillota_A	c__Clostridia	o__Oscillospirales	f__Ruminococcaceae	g__Ruthenibacterium	s__Ruthenibacterium lactatiformans
PD	PD60	bin19	MAG2680	d__Bacteria	p__Bacillota_A	c__Clostridia	o__Oscillospirales	f__Ruminococcaceae	g__Ruthenibacterium	s__Ruthenibacterium lactatiformans
PD	PD45	bin46	MAG3429	d__Bacteria	p__Bacillota_A	c__Clostridia	o__Oscillospirales	f__Ruminococcaceae	g__Ruthenibacterium	s__Ruthenibacterium lactatiformans
PD	PD31	bin22	MAG2894	d__Bacteria	p__Bacillota_A	c__Clostridia	o__Oscillospirales	f__Ruminococcaceae	g__Ruthenibacterium	s__Ruthenibacterium lactatiformans
PD	PD16	bin19	MAG3349	d__Bacteria	p__Bacillota_A	c__Clostridia	o__Oscillospirales	f__Acutalibacteraceae	g__CAG-177	s__CAG-177 sp003538135
PD	PD22	bin29	MAG2621	d__Bacteria	p__Bacillota_A	c__Clostridia	o__Oscillospirales	f__Acutalibacteraceae	g__CAG-177	s__CAG-177 sp003538135
PD	PD27	bin37	MAG954	d__Bacteria	p__Bacillota_A	c__Clostridia	o__Oscillospirales	f__Acutalibacteraceae	g__CAG-177	s__CAG-177 sp003514385
PD	PD70	bin25	MAG2382	d__Bacteria	p__Bacillota_A	c__Clostridia	o__Oscillospirales	f__Acutalibacteraceae	g__CAG-177	s__CAG-177 sp003514385
PD	PD94	bin31	MAG887	d__Bacteria	p__Bacillota_A	c__Clostridia	o__Oscillospirales	f__Acutalibacteraceae	g__CAG-177	s__CAG-177 sp003514385
PD	PD98	bin54	MAG208	d__Bacteria	p__Bacillota_A	c__Clostridia	o__Oscillospirales	f__Acutalibacteraceae	g__CAG-177	s__CAG-177 sp003514385
PD	PD10	bin8	MAG1616	d__Bacteria	p__Bacillota_A	c__Clostridia	o__Oscillospirales	f__Acutalibacteraceae	g__CAG-177	s__CAG-177 sp003514385
PD	PD2	bin9	MAG1069	d__Bacteria	p__Bacillota_A	c__Clostridia	o__Oscillospirales	f__Acutalibacteraceae	g__CAG-177	s__CAG-177 sp003514385
PD	PD34	bin34	MAG201	d__Bacteria	p__Bacillota_A	c__Clostridia	o__Oscillospirales	f__Acutalibacteraceae	g__CAG-177	s__CAG-177 sp003514385
PD	PD41	bin34	MAG2462	d__Bacteria	p__Bacillota_A	c__Clostridia	o__Oscillospirales	f__Acutalibacteraceae	g__CAG-177	s__CAG-177 sp003514385
PD	PD67	bin37	MAG94	d__Bacteria	p__Bacillota_A	c__Clostridia	o__Oscillospirales	f__Acutalibacteraceae	g__CAG-177	s__CAG-177 sp003514385
PD	PD39	bin7	MAG1337	d__Bacteria	p__Bacillota_A	c__Clostridia	o__Oscillospirales	f__Acutalibacteraceae	g__CAG-177	s__CAG-177 sp003514385
PD	PD72	bin49	MAG3377	d__Bacteria	p__Bacillota_A	c__Clostridia	o__Oscillospirales	f__Acutalibacteraceae	g__CAG-177	s__CAG-177 sp003514385
PD	PD32	bin12	MAG450	d__Bacteria	p__Bacillota_A	c__Clostridia	o__Oscillospirales	f__Acutalibacteraceae	g__CAG-177	s__CAG-177 sp003514385
PD	PD50	bin40	MAG2314	d__Bacteria	p__Bacillota_A	c__Clostridia	o__Oscillospirales	f__Acutalibacteraceae	g__CAG-488	s__CAG-488 sp000434055
PD	PD41	bin42	MAG1626	d__Bacteria	p__Bacillota_A	c__Clostridia	o__Oscillospirales	f__Acutalibacteraceae	g__CAG-488	s__CAG-488 sp000434055
PD	PD71	bin60	MAG634	d__Bacteria	p__Bacillota_A	c__Clostridia	o__Oscillospirales	f__Oscillospiraceae	g__Flavonifractor	s__Flavonifractor plautii
PD	PD43	bin32	MAG3297	d__Bacteria	p__Bacillota_A	c__Clostridia	o__Oscillospirales	f__Oscillospiraceae	g__Flavonifractor	s__Flavonifractor plautii
PD	PD100	bin30	MAG2137	d__Bacteria	p__Bacillota_A	c__Clostridia	o__Oscillospirales	f__Oscillospiraceae	g__Flavonifractor	s__Flavonifractor plautii
PD	PD83	bin32	MAG1476	d__Bacteria	p__Bacillota_A	c__Clostridia	o__Oscillospirales	f__Oscillospiraceae	g__Flavonifractor	s__Flavonifractor plautii
PD	PD77	bin29	MAG1632	d__Bacteria	p__Bacillota_A	c__Clostridia	o__Oscillospirales	f__Oscillospiraceae	g__Flavonifractor	s__Flavonifractor plautii
PD	PD78	bin20	MAG96	d__Bacteria	p__Bacillota_A	c__Clostridia	o__Oscillospirales	f__Oscillospiraceae	g__Flavonifractor	s__Flavonifractor plautii
PD	PD27	bin25	MAG566	d__Bacteria	p__Bacillota_A	c__Clostridia	o__Oscillospirales	f__Oscillospiraceae	g__Flavonifractor	s__Flavonifractor plautii
PD	PD21	bin21	MAG2749	d__Bacteria	p__Bacillota_A	c__Clostridia	o__Oscillospirales	f__Oscillospiraceae	g__Flavonifractor	s__Flavonifractor plautii
PD	PD20	bin53	MAG1994	d__Bacteria	p__Bacillota_A	c__Clostridia	o__Oscillospirales	f__Oscillospiraceae	g__Flavonifractor	s__Flavonifractor plautii
PD	PD76	bin46	MAG3454	d__Bacteria	p__Bacillota_A	c__Clostridia	o__Oscillospirales	f__Oscillospiraceae	g__Flavonifractor	s__Flavonifractor plautii
PD	PD35	bin32	MAG1298	d__Bacteria	p__Bacillota_A	c__Clostridia	o__Oscillospirales	f__Oscillospiraceae	g__Flavonifractor	s__Flavonifractor plautii
PD	PD1	bin13	MAG3027	d__Bacteria	p__Bacillota_A	c__Clostridia	o__Oscillospirales	f__Oscillospiraceae	g__Flavonifractor	s__Flavonifractor plautii
PD	PD37	bin31	MAG2433	d__Bacteria	p__Bacillota_A	c__Clostridia	o__Oscillospirales	f__Oscillospiraceae	g__Flavonifractor	s__Flavonifractor plautii
PD	PD29	bin49	MAG3330	d__Bacteria	p__Bacillota_A	c__Clostridia	o__Oscillospirales	f__Ruminococcaceae	g__CAG-115	s__CAG-115 sp003531585
PD	PD9	bin40	MAG2415	d__Bacteria	p__Bacillota_A	c__Clostridia	o__Oscillospirales	f__Ruminococcaceae	g__CAG-115	s__CAG-115 sp003531585
PD	PD29	bin68	MAG3509	d__Bacteria	p__Bacillota_A	c__Clostridia	o__Oscillospirales	f__Ruminococcaceae	g__CAG-353	s__CAG-353 sp900066885
PD	PD92	bin1	MAG617	d__Bacteria	p__Bacillota_A	c__Clostridia	o__Oscillospirales	f__Oscillospiraceae	g__Dysosmobacter	s__Dysosmobacter welbionis
PD	PD37	bin26	MAG1165	d__Bacteria	p__Bacillota_A	c__Clostridia	o__Oscillospirales	f__Oscillospiraceae	g__Dysosmobacter	s__Dysosmobacter welbionis
PD	PD77	bin56	MAG749	d__Bacteria	p__Bacillota_A	c__Clostridia	o__Oscillospirales	f__Oscillospiraceae	g__Dysosmobacter	s__Dysosmobacter welbionis

PD	PD11	bin17	MAG735	d__Bacteria	p__Bacillota_A	c__Clostridia	o__Oscillospirales	f__Oscillospiraceae	g__Dysosmobacter	s__Dysosmobacter welbionis
PD	PD1	bin14	MAG1084	d__Bacteria	p__Bacillota_A	c__Clostridia	o__Oscillospirales	f__Ruminococcaceae	g__Gemmiger	s__Gemmiger formicilis
PD	PD14	bin56	MAG2245	d__Bacteria	p__Bacillota_A	c__Clostridia	o__Oscillospirales	f__Ruminococcaceae	g__Gemmiger	s__Gemmiger formicilis
PD	PD66	bin37	MAG372	d__Bacteria	p__Bacillota_A	c__Clostridia	o__Oscillospirales	f__Ruminococcaceae	g__Gemmiger	s__Gemmiger formicilis
PD	PD13	bin44	MAG3435	d__Bacteria	p__Bacillota_A	c__Clostridia	o__Oscillospirales	f__Ruminococcaceae	g__Gemmiger	s__Gemmiger formicilis
PD	PD23	bin38	MAG2365	d__Bacteria	p__Bacillota_A	c__Clostridia	o__Oscillospirales	f__Ruminococcaceae	g__Gemmiger	s__Gemmiger formicilis
PD	PD9	bin19	MAG393	d__Bacteria	p__Bacillota_A	c__Clostridia	o__Oscillospirales	f__Ruminococcaceae	g__Gemmiger	s__Gemmiger formicilis
PD	PD17	bin32	MAG894	d__Bacteria	p__Bacillota_A	c__Clostridia	o__Oscillospirales	f__Ruminococcaceae	g__Gemmiger	s__Gemmiger formicilis
PD	PD77	bin13	MAG1876	d__Bacteria	p__Bacillota_A	c__Clostridia	o__Oscillospirales	f__Ruminococcaceae	g__Gemmiger	s__Gemmiger formicilis
PD	PD5	bin8	MAG2600	d__Bacteria	p__Bacillota_A	c__Clostridia	o__Oscillospirales	f__Ruminococcaceae	g__Gemmiger	s__Gemmiger formicilis
PD	PD62	bin50	MAG1604	d__Bacteria	p__Bacillota_A	c__Clostridia	o__Oscillospirales	f__Ruminococcaceae	g__Gemmiger	s__Gemmiger formicilis
PD	PD72	bin8	MAG1102	d__Bacteria	p__Bacillota_A	c__Clostridia	o__Oscillospirales	f__Ruminococcaceae	g__Gemmiger	s__Gemmiger formicilis
PD	PD71	bin15	MAG2904	d__Bacteria	p__Bacillota_A	c__Clostridia	o__Oscillospirales	f__Ruminococcaceae	g__Gemmiger	s__Gemmiger formicilis
PD	PD75	bin4	MAG3504	d__Bacteria	p__Bacillota_A	c__Clostridia	o__Oscillospirales	f__Ruminococcaceae	g__Gemmiger	s__Gemmiger formicilis
PD	PD46	bin41	MAG2313	d__Bacteria	p__Bacillota_A	c__Clostridia	o__Oscillospirales	f__Ruminococcaceae	g__Gemmiger	s__Gemmiger formicilis
PD	PD56	bin45	MAG982	d__Bacteria	p__Bacillota_A	c__Clostridia	o__Oscillospirales	f__Ruminococcaceae	g__Gemmiger	s__Gemmiger formicilis
PD	PD98	bin50	MAG3230	d__Bacteria	p__Bacillota_A	c__Clostridia	o__Oscillospirales	f__Ruminococcaceae	g__Gemmiger	s__Gemmiger formicilis
PD	PD68	bin59	MAG2672	d__Bacteria	p__Bacillota_A	c__Clostridia	o__Oscillospirales	f__Ruminococcaceae	g__Gemmiger	s__Gemmiger formicilis
PD	PD34	bin71	MAG391	d__Bacteria	p__Bacillota_A	c__Clostridia	o__Oscillospirales	f__Ruminococcaceae	g__Gemmiger	s__Gemmiger formicilis
PD	PD85	bin19	MAG827	d__Bacteria	p__Bacillota_A	c__Clostridia	o__Oscillospirales	f__Ruminococcaceae	g__Gemmiger	s__Gemmiger formicilis
PD	PD82	bin34	MAG2052	d__Bacteria	p__Bacillota_A	c__Clostridia	o__Oscillospirales	f__Ruminococcaceae	g__Gemmiger	s__Gemmiger formicilis
PD	PD16	bin36	MAG3114	d__Bacteria	p__Bacillota_A	c__Clostridia	o__Oscillospirales	f__Ruminococcaceae	g__Gemmiger	s__Gemmiger formicilis
PD	PD10	bin15	MAG2116	d__Bacteria	p__Bacillota_A	c__Clostridia	o__Oscillospirales	f__Ruminococcaceae	g__Gemmiger	s__Gemmiger formicilis
PD	PD20	bin50	MAG574	d__Bacteria	p__Bacillota_A	c__Clostridia	o__Oscillospirales	f__Ruminococcaceae	g__Gemmiger	s__Gemmiger formicilis
PD	PD76	bin40	MAG3439	d__Bacteria	p__Bacillota_A	c__Clostridia	o__Oscillospirales	f__Ruminococcaceae	g__Gemmiger	s__Gemmiger formicilis
PD	PD51	bin20	MAG1424	d__Bacteria	p__Bacillota_A	c__Clostridia	o__Oscillospirales	f__Ruminococcaceae	g__Gemmiger	s__Gemmiger formicilis
PD	PD6	bin20	MAG1935	d__Bacteria	p__Bacillota_A	c__Clostridia	o__Oscillospirales	f__Ruminococcaceae	g__Gemmiger	s__Gemmiger formicilis
PD	PD8	bin40	MAG355	d__Bacteria	p__Bacillota_A	c__Clostridia	o__Oscillospirales	f__Ruminococcaceae	g__Gemmiger	s__Gemmiger formicilis
PD	PD11	bin26	MAG2081	d__Bacteria	p__Bacillota_A	c__Clostridia	o__Oscillospirales	f__Oscillospiraceae	g__Lawsonibacter	s__Lawsonibacter sp900553135
PD	PD56	bin25	MAG3467	d__Bacteria	p__Bacillota_A	c__Clostridia	o__Oscillospirales	f__Ruminococcaceae	g__Ruthenibacterium	s__Ruthenibacterium merdipullorum
PD	PD72	bin3	MAG483	d__Bacteria	p__Bacillota_A	c__Clostridia	o__Oscillospirales	f__Ruminococcaceae	g__Ruthenibacterium	s__Ruthenibacterium merdipullorum
PD	PD18	bin53	MAG1615	d__Bacteria	p__Bacillota_A	c__Clostridia	o__Oscillospirales	f__Ruminococcaceae	g__Ruthenibacterium	s__Ruthenibacterium merdipullorum
PD	PD36	bin45	MAG3010	d__Bacteria	p__Bacillota_A	c__Clostridia	o__Oscillospirales	f__Ruminococcaceae	g__Ruthenibacterium	s__Ruthenibacterium merdipullorum
PD	PD97	bin65	MAG594	d__Bacteria	p__Bacillota_A	c__Clostridia	o__Oscillospirales	f__Ruminococcaceae	g__Ruthenibacterium	s__Ruthenibacterium merdipullorum
PD	PD60	bin4	MAG3286	d__Bacteria	p__Bacillota_A	c__Clostridia	o__Oscillospirales	f__Ruminococcaceae	g__Ruthenibacterium	s__Ruthenibacterium merdipullorum
PD	PD72	bin30	MAG1282	d__Bacteria	p__Bacillota_A	c__Clostridia	o__Oscillospirales	f__Oscillospiraceae	g__NSJ-51	s__NSJ-51 sp900549795
PD	PD28	bin18	MAG576	d__Bacteria	p__Bacillota_A	c__Clostridia	o__Oscillospirales	f__Ruminococcaceae	g__Ruminococcus_D	s__Ruminococcus_D sp000434695
PD	PD77	bin16	MAG1699	d__Bacteria	p__Bacillota_A	c__Clostridia	o__Oscillospirales	f__Acutalibacteraceae	g__Acutalibacter	s__Acutalibacter stercoravium
PD	PD94	bin72	MAG2307	d__Bacteria	p__Bacillota_A	c__Clostridia	o__Oscillospirales	f__Ruminococcaceae	g__Fournierella	s__Fournierella excrementavium
PD	PD50	bin60	MAG3331	d__Bacteria	p__Bacillota_A	c__Clostridia	o__Oscillospirales	f__CAG-272	g__CAG-841	s__CAG-841 sp000437375
PD	PD25	bin44	MAG2038	d__Bacteria	p__Bacillota_A	c__Clostridia	o__Oscillospirales	f__Acutalibacteraceae	g__UMGS1484	s__UMGS1484 sp900552285
PD	PD68	bin46	MAG1659	d__Bacteria	p__Bacillota_A	c__Clostridia	o__Oscillospirales	f__Acutalibacteraceae	g__UMGS1484	s__UMGS1484 sp900552285
PD	PD41	bin35	MAG3107	d__Bacteria	p__Bacillota_A	c__Clostridia	o__Oscillospirales	f__Oscillospiraceae	g__Vescimonas	s__Vescimonas sp001916855
PD	PD50	bin26	MAG1656	d__Bacteria	p__Bacillota_A	c__Clostridia	o__Oscillospirales	f__Oscillospiraceae	g__Vescimonas	s__Vescimonas sp001916855
PD	PD70	bin11	MAG1954	d__Bacteria	p__Bacillota_A	c__Clostridia	o__Oscillospirales	f__Oscillospiraceae	g__Vescimonas	s__Vescimonas sp001916855
PD	PD98	bin40	MAG3265	d__Bacteria	p__Bacillota_A	c__Clostridia	o__Oscillospirales	f__Oscillospiraceae	g__Vescimonas	s__Vescimonas sp001916855
PD	PD16	bin13	MAG1552	d__Bacteria	p__Bacillota_A	c__Clostridia	o__Oscillospirales	f__Oscillospiraceae	g__Vescimonas	s__Vescimonas sp001916855
PD	PD51	bin8	MAG130	d__Bacteria	p__Bacillota_A	c__Clostridia	o__Oscillospirales	f__Oscillospiraceae	g__Vescimonas	s__Vescimonas sp001916855
PD	PD50	bin9	MAG74	d__Bacteria	p__Bacillota_A	c__Clostridia	o__Oscillospirales	f__Oscillospiraceae	g__Pseudoflavonifractor	s__Pseudoflavonifractor sp014287675
PD	PD58	bin83	MAG71	d__Bacteria	p__Bacillota_A	c__Clostridia	o__Oscillospirales	f__Oscillospiraceae	g__Vescimonas	s__Vescimonas coprocola
PD	PD97	bin28	MAG2797	d__Bacteria	p__Bacillota_A	c__Clostridia	o__Oscillospirales	f__Oscillospiraceae	g__Vescimonas	s__Vescimonas coprocola

PD	PD56	bin62	MAG2654	d__Bacteria	p__Bacillota_A	c__Clostridia	o__Oscillospirales	f__Oscillospiraceae	g__Vescimonas	s__Vescimonas coprocola
PD	PD87	bin53	MAG1795	d__Bacteria	p__Bacillota_A	c__Clostridia	o__Oscillospirales	f__Oscillospiraceae	g__Vescimonas	s__Vescimonas coprocola
PD	PD29	bin74	MAG3043	d__Bacteria	p__Bacillota_A	c__Clostridia	o__Oscillospirales	f__Oscillospiraceae	g__Vescimonas	s__Vescimonas coprocola
PD	PD22	bin58	MAG1665	d__Bacteria	p__Bacillota_A	c__Clostridia	o__Oscillospirales	f__Oscillospiraceae	g__Vescimonas	s__Vescimonas coprocola
PD	PD32	bin27	MAG2062	d__Bacteria	p__Bacillota_A	c__Clostridia	o__Oscillospirales	f__Oscillospiraceae	g__Vescimonas	s__Vescimonas coprocola
PD	PD35	bin1	MAG2044	d__Bacteria	p__Bacillota_A	c__Clostridia	o__Oscillospirales	f__Oscillospiraceae	g__Vescimonas	s__Vescimonas coprocola
PD	PD9	bin30	MAG115	d__Bacteria	p__Bacillota_A	c__Clostridia	o__Oscillospirales	f__Oscillospiraceae	g__Vescimonas	s__Vescimonas coprocola
PD	PD19	bin34	MAG83	d__Bacteria	p__Bacillota_A	c__Clostridia	o__Oscillospirales	f__Oscillospiraceae	g__Vescimonas	s__Vescimonas coprocola
PD	PD50	bin34	MAG427	d__Bacteria	p__Bacillota_A	c__Clostridia	o__Oscillospirales	f__Oscillospiraceae	g__Vescimonas	s__Vescimonas coprocola
PD	PD28	bin17	MAG1278	d__Bacteria	p__Bacillota_A	c__Clostridia	o__Oscillospirales	f__Oscillospiraceae	g__Vescimonas	s__Vescimonas coprocola
PD	PD98	bin26	MAG1517	d__Bacteria	p__Bacillota_A	c__Clostridia	o__Oscillospirales	f__Oscillospiraceae	g__Vescimonas	s__Vescimonas sp003539495
PD	PD25	bin84	MAG1900	d__Bacteria	p__Bacillota_A	c__Clostridia	o__Oscillospirales	f__Oscillospiraceae	g__Vescimonas	s__Vescimonas sp003539495
PD	PD68	bin42	MAG491	d__Bacteria	p__Bacillota_A	c__Clostridia	o__Oscillospirales	f__Oscillospiraceae	g__Vescimonas	s__Vescimonas sp003539495
PD	PD18	bin33	MAG1967	d__Bacteria	p__Bacillota_A	c__Clostridia	o__Oscillospirales	f__Oscillospiraceae	g__Evtepia	s__Evtepia sp934597725
PD	PD70	bin58	MAG176	d__Bacteria	p__Bacillota_A	c__Clostridia	o__Oscillospirales	f__Oscillospiraceae	g__Faecousia	s__Faecousia sp003525905
PD	PD46	bin44	MAG1515	d__Bacteria	p__Bacillota_A	c__Clostridia	o__Oscillospirales	f__Oscillospiraceae	g__Faecousia	s__Faecousia sp003525905
PD	PD60	bin25	MAG994	d__Bacteria	p__Bacillota_A	c__Clostridia	o__Oscillospirales	f__Oscillospiraceae	g__Faecousia	s__Faecousia sp003525905
PD	PD16	bin29	MAG623	d__Bacteria	p__Bacillota_A	c__Clostridia	o__Oscillospirales	f__Oscillospiraceae	g__Faecousia	s__Faecousia sp003525905
PD	PD59	bin53	MAG1542	d__Bacteria	p__Bacillota_A	c__Clostridia	o__Oscillospirales	f__Oscillospiraceae	g__Faecousia	s__Faecousia sp003525905
PD	PD92	bin12	MAG99	d__Bacteria	p__Bacillota_A	c__Clostridia	o__Oscillospirales	f__Oscillospiraceae	g__Faecousia	s__Faecousia sp003525905
PD	PD29	bin55	MAG249	d__Bacteria	p__Bacillota_A	c__Clostridia	o__Oscillospirales	f__Oscillospiraceae	g__Faecousia	s__Faecousia sp003525905
PD	PD41	bin45	MAG993	d__Bacteria	p__Bacillota_A	c__Clostridia	o__Oscillospirales	f__Oscillospiraceae	g__Faecousia	s__Faecousia sp003525905
PD	PD23	bin42	MAG2585	d__Bacteria	p__Bacillota_A	c__Clostridia	o__Oscillospirales	f__Oscillospiraceae	g__Faecousia	s__Faecousia sp003525905
PD	PD97	bin1	MAG1827	d__Bacteria	p__Bacillota_A	c__Clostridia	o__Oscillospirales	f__Oscillospiraceae	g__Faecousia	s__Faecousia sp003525905
PD	PD6	bin28	MAG31	d__Bacteria	p__Bacillota_A	c__Clostridia	o__Oscillospirales	f__Oscillospiraceae	g__Faecousia	s__Faecousia sp003525905
PD	PD48	bin83	MAG2124	d__Bacteria	p__Bacillota_A	c__Clostridia	o__Oscillospirales	f__Oscillospiraceae	g__Faecousia	s__Faecousia sp003525905
PD	PD39	bin38	MAG238	d__Bacteria	p__Bacillota_A	c__Clostridia	o__Oscillospirales	f__Oscillospiraceae	g__Faecousia	s__Faecousia sp003525905
PD	PD57	bin72	MAG1380	d__Bacteria	p__Bacillota_A	c__Clostridia	o__Oscillospirales	f__Oscillospiraceae	g__Vescimonas	s__Vescimonas sp900545585
PD	PD48	bin66	MAG3212	d__Bacteria	p__Bacillota_A	c__Clostridia	o__Oscillospirales	f__Oscillospiraceae	g__Vescimonas	s__Vescimonas sp900545585
PD	PD25	bin74	MAG1824	d__Bacteria	p__Bacillota_A	c__Clostridia	o__Oscillospirales	f__Oscillospiraceae	g__Vescimonas	s__Vescimonas sp900545585
PD	PD47	bin40	MAG180	d__Bacteria	p__Bacillota_A	c__Clostridia	o__Oscillospirales	f__Ruminococcaceae	g__Gemmiger	s__Gemmiger sp900540595
PD	PD12	bin64	MAG686	d__Bacteria	p__Bacillota_A	c__Clostridia	o__Oscillospirales	f__Ruminococcaceae	g__Gemmiger	s__Gemmiger sp900540595
PD	PD32	bin37	MAG1452	d__Bacteria	p__Bacillota_A	c__Clostridia	o__Oscillospirales	f__Ruminococcaceae	g__Gemmiger	s__Gemmiger sp900540595
PD	PD68	bin18	MAG1561	d__Bacteria	p__Bacillota_A	c__Clostridia	o__Oscillospirales	f__Ruminococcaceae	g__Gemmiger	s__Gemmiger sp900540595
PD	PD8	bin66	MAG259	d__Bacteria	p__Bacillota_A	c__Clostridia	o__Oscillospirales	f__Ruminococcaceae	g__Gemmiger	s__Gemmiger sp900540595
PD	PD6	bin12	MAG1256	d__Bacteria	p__Bacillota_A	c__Clostridia	o__Oscillospirales	f__Ruminococcaceae	g__Gemmiger	s__Gemmiger sp900540595
PD	PD45	bin16	MAG91	d__Bacteria	p__Bacillota_A	c__Clostridia	o__Oscillospirales	f__Ruminococcaceae	g__Gemmiger	s__Gemmiger sp900540595
PD	PD92	bin7	MAG3042	d__Bacteria	p__Bacillota_A	c__Clostridia	o__Oscillospirales	f__Oscillospiraceae	g__Lawsonibacter	s__Lawsonibacter sp900545895
PD	PD18	bin57	MAG2108	d__Bacteria	p__Bacillota_A	c__Clostridia	o__Oscillospirales	f__Acutalibacteraceae	g__Acutalibacter	s__Acutalibacter ornithocaccae
PD	PD7	bin12	MAG2792	d__Bacteria	p__Bacillota_A	c__Clostridia	o__Oscillospirales	f__Oscillospiraceae	g__Vescimonas	s__Vescimonas sp000435555
PD	PD58	bin25	MAG1671	d__Bacteria	p__Bacillota_A	c__Clostridia	o__Oscillospirales	f__Oscillospiraceae	g__Vescimonas	s__Vescimonas sp000435555
PD	PD12	bin67	MAG2280	d__Bacteria	p__Bacillota_A	c__Clostridia	o__Oscillospirales	f__Oscillospiraceae	g__Vescimonas	s__Vescimonas sp000435555
PD	PD32	bin83	MAG3236	d__Bacteria	p__Bacillota_A	c__Clostridia	o__Oscillospirales	f__Oscillospiraceae	g__ER4	s__ER4 sp900317525
PD	PD5	bin12	MAG2268	d__Bacteria	p__Bacillota_A	c__Clostridia	o__Oscillospirales	f__Oscillospiraceae	g__ER4	s__ER4 sp900317525
PD	PD25	bin3	MAG712	d__Bacteria	p__Bacillota_A	c__Clostridia	o__Oscillospirales	f__Oscillospiraceae	g__ER4	s__ER4 sp900317525
PD	PD31	bin28	MAG1610	d__Bacteria	p__Bacillota_A	c__Clostridia	o__Oscillospirales	f__Oscillospiraceae	g__ER4	s__ER4 sp900317525
PD	PD41	bin72	MAG521	d__Bacteria	p__Bacillota_A	c__Clostridia	o__Oscillospirales	f__Oscillospiraceae	g__ER4	s__ER4 sp900317525
PD	PD58	bin19	MAG283	d__Bacteria	p__Bacillota_A	c__Clostridia	o__Oscillospirales	f__Oscillospiraceae	g__ER4	s__ER4 sp900317525
PD	PD9	bin43	MAG1977	d__Bacteria	p__Bacillota_A	c__Clostridia	o__Oscillospirales	f__Oscillospiraceae	g__ER4	s__ER4 sp900317525
PD	PD50	bin13	MAG2986	d__Bacteria	p__Bacillota_A	c__Clostridia	o__Oscillospirales	f__Oscillospiraceae	g__ER4	s__ER4 sp900317525
PD	PD24	bin42	MAG425	d__Bacteria	p__Bacillota_A	c__Clostridia	o__Oscillospirales	f__Oscillospiraceae	g__ER4	s__ER4 sp900317525

PD	PD44	bin22	MAG1405	d__Bacteria	p__Bacillota_A	c__Clostridia	o__Oscillospirales	f__Oscillospiraceae	g__CAG-103	s__CAG-103 sp000432375
PD	PD69	bin42	MAG2200	d__Bacteria	p__Bacillota_A	c__Clostridia	o__Oscillospirales	f__Oscillospiraceae	g__CAG-103	s__CAG-103 sp000432375
PD	PD57	bin21	MAG2915	d__Bacteria	p__Bacillota_A	c__Clostridia	o__Oscillospirales	f__Oscillospiraceae	g__CAG-103	s__CAG-103 sp000432375
PD	PD9	bin9	MAG841	d__Bacteria	p__Bacillota_A	c__Clostridia	o__Oscillospirales	f__Oscillospiraceae	g__CAG-103	s__CAG-103 sp000432375
PD	PD92	bin30	MAG2539	d__Bacteria	p__Bacillota_A	c__Clostridia	o__Oscillospirales	f__Oscillospiraceae	g__CAG-103	s__CAG-103 sp000432375
PD	PD10	bin24	MAG2020	d__Bacteria	p__Bacillota_A	c__Clostridia	o__Oscillospirales	f__Oscillospiraceae	g__CAG-103	s__CAG-103 sp000432375
PD	PD70	bin55	MAG1035	d__Bacteria	p__Bacillota_A	c__Clostridia	o__Oscillospirales	f__Oscillospiraceae	g__CAG-103	s__CAG-103 sp000432375
PD	PD36	bin22	MAG1239	d__Bacteria	p__Bacillota_A	c__Clostridia	o__Oscillospirales	f__Oscillospiraceae	g__CAG-103	s__CAG-103 sp000432375
PD	PD72	bin13	MAG128	d__Bacteria	p__Bacillota_A	c__Clostridia	o__Oscillospirales	f__Oscillospiraceae	g__CAG-103	s__CAG-103 sp000432375
PD	PD57	bin14	MAG192	d__Bacteria	p__Bacillota_A	c__Clostridia	o__Oscillospirales	f__Oscillospiraceae	g__F23-B02	s__F23-B02 sp001916715
PD	PD22	bin31	MAG1691	d__Bacteria	p__Bacillota_A	c__Clostridia	o__Oscillospirales	f__Oscillospiraceae	g__F23-B02	s__F23-B02 sp001916715
PD	PD73	bin56	MAG715	d__Bacteria	p__Bacillota_A	c__Clostridia	o__Oscillospirales	f__Oscillospiraceae	g__F23-B02	s__F23-B02 sp001916715
PD	PD64	bin55	MAG1294	d__Bacteria	p__Bacillota_A	c__Clostridia	o__Oscillospirales	f__Oscillospiraceae	g__Vescimonas	s__Vescimonas sp003487665
PD	PD50	bin24	MAG1303	d__Bacteria	p__Bacillota_A	c__Clostridia	o__Oscillospirales	f__Oscillospiraceae	g__Vescimonas	s__Vescimonas sp003487665
PD	PD13	bin48	MAG1682	d__Bacteria	p__Bacillota_A	c__Clostridia	o__Oscillospirales	f__Oscillospiraceae	g__Vescimonas	s__Vescimonas sp003487665
PD	PD57	bin10	MAG2953	d__Bacteria	p__Bacillota_A	c__Clostridia	o__Oscillospirales	f__Oscillospiraceae	g__Vescimonas	s__Vescimonas sp003487665
PD	PD5	bin14	MAG1809	d__Bacteria	p__Bacillota_A	c__Clostridia	o__Oscillospirales	f__Oscillospiraceae	g__Vescimonas	s__Vescimonas sp003487665
PD	PD50	bin38	MAG820	d__Bacteria	p__Bacillota_A	c__Clostridia	o__Oscillospirales	f__Ruminococcaceae	g__Fournierella	s__Fournierella sp900543285
PD	PD94	bin70	MAG1314	d__Bacteria	p__Bacillota_A	c__Clostridia	o__Oscillospirales	f__Oscillospiraceae	g__CAG-170	s__CAG-170 sp900545925
PD	PD8	bin14	MAG2765	d__Bacteria	p__Bacillota_A	c__Clostridia	o__Oscillospirales	f__Oscillospiraceae	g__CAG-170	s__CAG-170 sp900545925
PD	PD56	bin19	MAG2634	d__Bacteria	p__Bacillota_A	c__Clostridia	o__Oscillospirales	f__Oscillospiraceae	g__CAG-170	s__CAG-170 sp900545925
PD	PD97	bin19	MAG1119	d__Bacteria	p__Bacillota_A	c__Clostridia	o__Oscillospirales	f__Oscillospiraceae	g__Evtepia	s__Unclassified
PD	PD68	bin29	MAG3329	d__Bacteria	p__Bacillota_A	c__Clostridia	o__Oscillospirales	f__Oscillospiraceae	g__CAG-170	s__CAG-170 sp000436735
PD	PD17	bin17	MAG3082	d__Bacteria	p__Bacillota_A	c__Clostridia	o__Oscillospirales	f__Acutalibacteraceae	g__Avimonas_A	s__Avimonas_A narfia
PD	PD18	bin3	MAG1089	d__Bacteria	p__Bacillota_A	c__Clostridia	o__Oscillospirales	f__Oscillospiraceae	g__Lawsonibacter	s__Lawsonibacter sp000177015
PD	PD16	bin51	MAG2838	d__Bacteria	p__Bacillota_A	c__Clostridia	o__Oscillospirales	f__Oscillospiraceae	g__Lawsonibacter	s__Lawsonibacter sp000177015
PD	PD6	bin7	MAG2248	d__Bacteria	p__Bacillota_A	c__Clostridia	o__Oscillospirales	f__Oscillospiraceae	g__UBA738	s__UBA738 sp003522945
PD	PD32	bin77	MAG1186	d__Bacteria	p__Bacillota_A	c__Clostridia	o__Oscillospirales	f__Oscillospiraceae	g__UBA738	s__UBA738 sp003522945
PD	PD71	bin30	MAG1589	d__Bacteria	p__Bacillota_A	c__Clostridia	o__Oscillospirales	f__Oscillospiraceae	g__Intestinimonas	s__Intestinimonas massiliensis
PD	PD2	bin50	MAG1692	d__Bacteria	p__Bacillota_A	c__Clostridia	o__Oscillospirales	f__Oscillospiraceae	g__Evtepia	s__Evtepia gabavorous
PD	PD57	bin85	MAG3195	d__Bacteria	p__Bacillota_A	c__Clostridia	o__Oscillospirales	f__Oscillospiraceae	g__Evtepia	s__Evtepia gabavorous
PD	PD58	bin20	MAG664	d__Bacteria	p__Bacillota_A	c__Clostridia	o__Oscillospirales	f__Oscillospiraceae	g__Evtepia	s__Evtepia gabavorous
PD	PD59	bin22	MAG1009	d__Bacteria	p__Bacillota_A	c__Clostridia	o__Oscillospirales	f__Oscillospiraceae	g__Evtepia	s__Evtepia gabavorous
PD	PD48	bin40	MAG2404	d__Bacteria	p__Bacillota_A	c__Clostridia	o__Oscillospirales	f__Oscillospiraceae	g__Evtepia	s__Evtepia gabavorous
PD	PD56	bin36	MAG3168	d__Bacteria	p__Bacillota_A	c__Clostridia	o__Oscillospirales	f__Oscillospiraceae	g__Evtepia	s__Evtepia gabavorous
PD	PD73	bin15	MAG4	d__Bacteria	p__Bacillota_A	c__Clostridia	o__Oscillospirales	f__Oscillospiraceae	g__Evtepia	s__Evtepia gabavorous
PD	PD66	bin32	MAG290	d__Bacteria	p__Bacillota_A	c__Clostridia	o__Oscillospirales	f__Oscillospiraceae	g__Evtepia	s__Evtepia gabavorous
PD	PD18	bin27	MAG1636	d__Bacteria	p__Bacillota_A	c__Clostridia	o__Oscillospirales	f__Acutalibacteraceae	g__Anaeromassilibacillus	s__Anaeromassilibacillus stercoravium
PD	PD59	bin44	MAG526	d__Bacteria	p__Bacillota_A	c__Clostridia	o__Oscillospirales	f__Ruminococcaceae	g__Anaerotruncus	s__Anaerotruncus colihominis
PD	PD64	bin23	MAG662	d__Bacteria	p__Bacillota_A	c__Clostridia	o__Oscillospirales	f__UBA644	g__UBA644	s__UBA644 sp002299265
PD	PD29	bin12	MAG1576	d__Bacteria	p__Bacillota_A	c__Clostridia	o__Oscillospirales	f__Oscillospiraceae	g__Dysosmobacter	s__Dysosmobacter sp021771635
PD	PD2	bin16	MAG1645	d__Bacteria	p__Bacillota_A	c__Clostridia	o__Oscillospirales	f__Ruminococcaceae	g__UBA1394	s__UBA1394 sp900066845
PD	PD36	bin14	MAG3016	d__Bacteria	p__Bacillota_A	c__Clostridia	o__Oscillospirales	f__Acutalibacteraceae	g__Pseudoruminococcus	s__Pseudoruminococcus sp902765855
PD	PD73	bin57	MAG1674	d__Bacteria	p__Bacillota_A	c__Clostridia	o__Oscillospirales	f__Acutalibacteraceae	g__CAG-177	s__CAG-177 sp000431775
PD	PD12	bin6	MAG1998	d__Bacteria	p__Bacillota_A	c__Clostridia	o__Oscillospirales	f__Acutalibacteraceae	g__CAG-177	s__CAG-177 sp000431775
PD	PD35	bin46	MAG2564	d__Bacteria	p__Bacillota_A	c__Clostridia	o__Oscillospirales	f__Ruminococcaceae	g__Faecalibacterium	s__Faecalibacterium sp900539945
PD	PD27	bin33	MAG1355	d__Bacteria	p__Bacillota_A	c__Clostridia	o__Oscillospirales	f__Ruminococcaceae	g__Faecalibacterium	s__Faecalibacterium sp900539945
PD	PD68	bin8	MAG2260	d__Bacteria	p__Bacillota_A	c__Clostridia	o__Oscillospirales	f__Ruminococcaceae	g__Faecalibacterium	s__Faecalibacterium sp900539945
PD	PD24	bin18	MAG193	d__Bacteria	p__Bacillota_A	c__Clostridia	o__Oscillospirales	f__Ruminococcaceae	g__Faecalibacterium	s__Faecalibacterium sp900539945
PD	PD29	bin75	MAG3098	d__Bacteria	p__Bacillota_A	c__Clostridia	o__Oscillospirales	f__Ruminococcaceae	g__Faecalibacterium	s__Faecalibacterium sp900539945
PD	PD61	bin32	MAG570	d__Bacteria	p__Bacillota_A	c__Clostridia	o__Oscillospirales	f__Ruminococcaceae	g__Faecalibacterium	s__Faecalibacterium sp900539945

PD	PD62	bin5	MAG1110	d__Bacteria	p__Bacillota_A	c__Clostridia	o__Oscillospirales	f__Ruminococcaceae	g__Faecalibacterium	s__Faecalibacterium sp900539945
PD	PD23	bin11	MAG1366	d__Bacteria	p__Bacillota_A	c__Clostridia	o__Oscillospirales	f__Ruminococcaceae	g__Faecalibacterium	s__Faecalibacterium sp900539945
PD	PD69	bin55	MAG1115	d__Bacteria	p__Bacillota_A	c__Clostridia	o__Oscillospirales	f__Ruminococcaceae	g__Faecalibacterium	s__Faecalibacterium sp900539945
PD	PD72	bin12	MAG3062	d__Bacteria	p__Bacillota_A	c__Clostridia	o__Oscillospirales	f__Oscillospiraceae	g__CAG-170	s__CAG-170 sp002404795
PD	PD18	bin34	MAG3478	d__Bacteria	p__Bacillota_A	c__Clostridia	o__Oscillospirales	f__Ruminococcaceae	g__Negativibacillus	s__Negativibacillus faecipullorum
PD	PD94	bin52	MAG2762	d__Bacteria	p__Bacillota_A	c__Clostridia	o__Oscillospirales	f__Ruminococcaceae	g__Negativibacillus	s__Negativibacillus faecipullorum
PD	PD12	bin74	MAG3421	d__Bacteria	p__Bacillota_A	c__Clostridia	o__Oscillospirales	f__Oscillospiraceae	g__Faecousia	s__Faecousia sp900546415
PD	PD50	bin29	MAG2865	d__Bacteria	p__Bacillota_A	c__Clostridia	o__Oscillospirales	f__Ruminococcaceae	g__Angelakisella	s__Angelakisella sp900547385
PD	PD25	bin21	MAG11	d__Bacteria	p__Bacillota_A	c__Clostridia	o__Oscillospirales	f__Ruminococcaceae	g__Angelakisella	s__Angelakisella sp900547385
PD	PD67	bin9	MAG3300	d__Bacteria	p__Bacillota_A	c__Clostridia	o__Oscillospirales	f__Ruminococcaceae	g__Angelakisella	s__Angelakisella sp900547385
PD	PD32	bin50	MAG2581	d__Bacteria	p__Bacillota_A	c__Clostridia	o__Oscillospirales	f__Ruminococcaceae	g__Angelakisella	s__Angelakisella sp900547385
PD	PD62	bin69	MAG2283	d__Bacteria	p__Bacillota_A	c__Clostridia	o__Oscillospirales	f__Ruminococcaceae	g__Angelakisella	s__Angelakisella sp900547385
PD	PD41	bin40	MAG2658	d__Bacteria	p__Bacillota_A	c__Clostridia	o__Oscillospirales	f__Ruminococcaceae	g__Angelakisella	s__Angelakisella sp900547385
PD	PD12	bin63	MAG3406	d__Bacteria	p__Bacillota_A	c__Clostridia	o__Oscillospirales	f__Ruminococcaceae	g__Angelakisella	s__Angelakisella sp900547385
PD	PD94	bin18	MAG1118	d__Bacteria	p__Bacillota_A	c__Clostridia	o__Oscillospirales	f__Ruminococcaceae	g__Angelakisella	s__Angelakisella sp900547385
PD	PD6	bin29	MAG1782	d__Bacteria	p__Bacillota_A	c__Clostridia	o__Oscillospirales	f__Ruminococcaceae	g__Angelakisella	s__Angelakisella sp900547385
PD	PD18	bin44	MAG2994	d__Bacteria	p__Bacillota_A	c__Clostridia	o__Oscillospirales	f__Ruminococcaceae	g__Fournierella	s__Fournierella massiliensis
PD	PD23	bin30	MAG3315	d__Bacteria	p__Bacillota_A	c__Clostridia	o__Oscillospirales	f__Ruminococcaceae	g__Faecalibacterium	s__Faecalibacterium longum
PD	PD82	bin13	MAG1067	d__Bacteria	p__Bacillota_A	c__Clostridia	o__Oscillospirales	f__Ruminococcaceae	g__Faecalibacterium	s__Faecalibacterium longum
PD	PD62	bin57	MAG318	d__Bacteria	p__Bacillota_A	c__Clostridia	o__Oscillospirales	f__Ruminococcaceae	g__Faecalibacterium	s__Faecalibacterium longum
PD	PD75	bin12	MAG2457	d__Bacteria	p__Bacillota_A	c__Clostridia	o__Oscillospirales	f__Ruminococcaceae	g__Faecalibacterium	s__Faecalibacterium longum
PD	PD53	bin43	MAG2114	d__Bacteria	p__Bacillota_A	c__Clostridia	o__Oscillospirales	f__Ruminococcaceae	g__Faecalibacterium	s__Faecalibacterium longum
PD	PD44	bin17	MAG943	d__Bacteria	p__Bacillota_A	c__Clostridia	o__Oscillospirales	f__Ruminococcaceae	g__Faecalibacterium	s__Faecalibacterium longum
PD	PD29	bin79	MAG1964	d__Bacteria	p__Bacillota_A	c__Clostridia	o__Oscillospirales	f__Ruminococcaceae	g__Faecalibacterium	s__Faecalibacterium longum
PD	PD40	bin47	MAG2050	d__Bacteria	p__Bacillota_A	c__Clostridia	o__Oscillospirales	f__Ruminococcaceae	g__Faecalibacterium	s__Faecalibacterium longum
PD	PD95	bin33	MAG3310	d__Bacteria	p__Bacillota_A	c__Clostridia	o__Oscillospirales	f__Ruminococcaceae	g__Faecalibacterium	s__Faecalibacterium longum
PD	PD91	bin23	MAG3344	d__Bacteria	p__Bacillota_A	c__Clostridia	o__Oscillospirales	f__Ruminococcaceae	g__Faecalibacterium	s__Faecalibacterium longum
PD	PD37	bin47	MAG3325	d__Bacteria	p__Bacillota_A	c__Clostridia	o__Oscillospirales	f__Ruminococcaceae	g__Faecalibacterium	s__Faecalibacterium longum
PD	PD78	bin41	MAG262	d__Bacteria	p__Bacillota_A	c__Clostridia	o__Oscillospirales	f__Ruminococcaceae	g__Faecalibacterium	s__Faecalibacterium longum
PD	PD28	bin62	MAG1714	d__Bacteria	p__Bacillota_A	c__Clostridia	o__Oscillospirales	f__Ruminococcaceae	g__Faecalibacterium	s__Faecalibacterium longum
PD	PD92	bin51	MAG3387	d__Bacteria	p__Bacillota_A	c__Clostridia	o__Oscillospirales	f__Acutalibacteraceae	g__Clostridium_A	s__Clostridium_A leptum
PD	PD34	bin77	MAG2551	d__Bacteria	p__Bacillota_A	c__Clostridia	o__Oscillospirales	f__Acutalibacteraceae	g__Clostridium_A	s__Clostridium_A leptum
PD	PD48	bin44	MAG2403	d__Bacteria	p__Bacillota_A	c__Clostridia	o__Oscillospirales	f__Acutalibacteraceae	g__Clostridium_A	s__Clostridium_A leptum
PD	PD32	bin43	MAG740	d__Bacteria	p__Bacillota_A	c__Clostridia	o__Oscillospirales	f__Acutalibacteraceae	g__Clostridium_A	s__Clostridium_A leptum
PD	PD19	bin7	MAG2912	d__Bacteria	p__Bacillota_A	c__Clostridia	o__Oscillospirales	f__Ruminococcaceae	g__Ruminococcus_C	s__Ruminococcus_C sp000433635
PD	PD17	bin40	MAG2961	d__Bacteria	p__Bacillota_A	c__Clostridia	o__Oscillospirales	f__Ruminococcaceae	g__Ruminococcus_C	s__Ruminococcus_C sp000433635
PD	PD45	bin38	MAG1826	d__Bacteria	p__Bacillota_A	c__Clostridia	o__Oscillospirales	f__Ruminococcaceae	g__Ruminococcus_C	s__Ruminococcus_C sp000433635
PD	PD92	bin44	MAG2413	d__Bacteria	p__Bacillota_A	c__Clostridia	o__Oscillospirales	f__Ruminococcaceae	g__Ruminococcus_C	s__Ruminococcus_C sp000433635
PD	PD67	bin11	MAG3398	d__Bacteria	p__Bacillota_A	c__Clostridia	o__Oscillospirales	f__Ruminococcaceae	g__Ruminococcus_C	s__Ruminococcus_C sp000433635
PD	PD23	bin34	MAG2347	d__Bacteria	p__Bacillota_A	c__Clostridia	o__Oscillospirales	f__Ruminococcaceae	g__Ruminococcus_C	s__Ruminococcus_C sp000433635
PD	PD10	bin20	MAG1387	d__Bacteria	p__Bacillota_A	c__Clostridia	o__Oscillospirales	f__Ruminococcaceae	g__Ruminococcus_C	s__Ruminococcus_C sp000433635
PD	PD7	bin34	MAG3352	d__Bacteria	p__Bacillota_A	c__Clostridia	o__Oscillospirales	f__Oscillospiraceae	g__Vescimonas	s__Vescimonas sp000435975
PD	PD76	bin52	MAG24	d__Bacteria	p__Bacillota_A	c__Clostridia	o__Oscillospirales	f__Ruminococcaceae	g__Faecalibacterium	s__Faecalibacterium sp900765705
PD	PD51	bin21	MAG527	d__Bacteria	p__Bacillota_A	c__Clostridia	o__Oscillospirales	f__UBA644	g__UBA644	s__UBA644 sp900547165
PD	PD41	bin41	MAG774	d__Bacteria	p__Bacillota_A	c__Clostridia	o__Oscillospirales	f__UBA644	g__UBA644	s__UBA644 sp900547165
PD	PD13	bin8	MAG2094	d__Bacteria	p__Bacillota_A	c__Clostridia	o__Oscillospirales	f__Ruminococcaceae	g__CAG-115	s__CAG-115 sp003507295
PD	PD28	bin56	MAG1394	d__Bacteria	p__Bacillota_A	c__Clostridia	o__Oscillospirales	f__Ruminococcaceae	g__CAG-115	s__CAG-115 sp003507295
PD	PD64	bin30	MAG2956	d__Bacteria	p__Bacillota_A	c__Clostridia	o__Oscillospirales	f__Ruminococcaceae	g__Ruminococcus_C	s__Ruminococcus_C sp000980705
PD	PD48	bin61	MAG289	d__Bacteria	p__Bacillota_A	c__Clostridia	o__Oscillospirales	f__Ruminococcaceae	g__Ruminococcus_C	s__Ruminococcus_C sp000980705
PD	PD37	bin15	MAG699	d__Bacteria	p__Bacillota_A	c__Clostridia	o__Oscillospirales	f__Acutalibacteraceae	g__Eubacterium_R	s__Eubacterium_R sp003526845
PD	PD92	bin9	MAG2728	d__Bacteria	p__Bacillota_A	c__Clostridia	o__Oscillospirales	f__Acutalibacteraceae	g__Fimivicinus	s__Fimivicinus sp900544375

PD	PD29	bin83	MAG2929	d__Bacteria	p__Bacillota_A	c__Clostridia	o__Oscillospirales	f__Acutalibacteraceae	g__Fimivicinus	s__Fimivicinus sp900544375
PD	PD59	bin6	MAG2578	d__Bacteria	p__Bacillota_A	c__Clostridia	o__Oscillospirales	f__Acutalibacteraceae	g__Fimivicinus	s__Fimivicinus sp900544375
PD	PD40	bin36	MAG1761	d__Bacteria	p__Bacillota_A	c__Clostridia	o__Oscillospirales	f__Oscillospiraceae	g__CAG-103	s__CAG-103 sp900543625
PD	PD8	bin34	MAG2636	d__Bacteria	p__Bacillota_A	c__Clostridia	o__Oscillospirales	f__Oscillospiraceae	g__CAG-103	s__CAG-103 sp900543625
PD	PD48	bin23	MAG1401	d__Bacteria	p__Bacillota_A	c__Clostridia	o__Oscillospirales	f__Oscillospiraceae	g__CAG-103	s__CAG-103 sp900543625
PD	PD41	bin11	MAG2096	d__Bacteria	p__Bacillota_A	c__Clostridia	o__Oscillospirales	f__Oscillospiraceae	g__CAG-103	s__CAG-103 sp900543625
PD	PD51	bin38	MAG3260	d__Bacteria	p__Bacillota_A	c__Clostridia	o__Oscillospirales	f__Oscillospiraceae	g__CAG-103	s__CAG-103 sp900543625
PD	PD29	bin32	MAG1650	d__Bacteria	p__Bacillota_A	c__Clostridia	o__Oscillospirales	f__Oscillospiraceae	g__CAG-170	s__CAG-170 sp000432135
PD	PD64	bin9	MAG1046	d__Bacteria	p__Bacillota_A	c__Clostridia	o__Oscillospirales	f__Oscillospiraceae	g__CAG-170	s__CAG-170 sp000432135
PD	PD7	bin6	MAG2053	d__Bacteria	p__Bacillota_A	c__Clostridia	o__Oscillospirales	f__Oscillospiraceae	g__CAG-170	s__CAG-170 sp000432135
PD	PD57	bin2	MAG1774	d__Bacteria	p__Bacillota_A	c__Clostridia	o__Oscillospirales	f__Oscillospiraceae	g__CAG-170	s__CAG-170 sp000432135
PD	PD12	bin40	MAG1752	d__Bacteria	p__Bacillota_A	c__Clostridia	o__Oscillospirales	f__Oscillospiraceae	g__Limivicinus	s__Limivicinus sp003150355
PD	PD100	bin36	MAG1573	d__Bacteria	p__Bacillota_A	c__Clostridia	o__Oscillospirales	f__Oscillospiraceae	g__Lawsonibacter	s__Lawsonibacter asaccharolyticus
PD	PD70	bin50	MAG65	d__Bacteria	p__Bacillota_A	c__Clostridia	o__Oscillospirales	f__Ruminococcaceae	g__Ruminococcus_C	s__Ruminococcus_C sp000437255
PD	PD32	bin46	MAG2489	d__Bacteria	p__Bacillota_A	c__Clostridia	o__Oscillospirales	f__Ruminococcaceae	g__Ruminococcus	s__Ruminococcus sp900540005
PD	PD97	bin53	MAG2506	d__Bacteria	p__Bacillota_A	c__Clostridia	o__Oscillospirales	f__Oscillospiraceae	g__CAG-103	s__CAG-103 sp900757655
PD	PD12	bin9	MAG3374	d__Bacteria	p__Bacillota_A	c__Clostridia	o__Oscillospirales	f__Oscillospiraceae	g__CAG-103	s__CAG-103 sp900757655
PD	PD59	bin42	MAG2951	d__Bacteria	p__Bacillota_A	c__Clostridia	o__Oscillospirales	f__Oscillospiraceae	g__CAG-103	s__CAG-103 sp900757655
PD	PD56	bin42	MAG2945	d__Bacteria	p__Bacillota_A	c__Clostridia	o__Oscillospirales	f__Oscillospiraceae	g__CAG-103	s__CAG-103 sp900757655
PD	PD29	bin71	MAG1068	d__Bacteria	p__Bacillota_A	c__Clostridia	o__Oscillospirales	f__Oscillospiraceae	g__CAG-103	s__CAG-103 sp900757655
PD	PD3	bin22	MAG2435	d__Bacteria	p__Bacillota_A	c__Clostridia	o__Oscillospirales	f__Ruminococcaceae	g__UBA1394	s__UBA1394 sp900538575
PD	PD97	bin55	MAG1749	d__Bacteria	p__Bacillota_A	c__Clostridia	o__Oscillospirales	f__Acutalibacteraceae	g__Ruminococcus_E	s__Ruminococcus_E sp905215855
PD	PD12	bin30	MAG846	d__Bacteria	p__Bacillota_A	c__Clostridia	o__Oscillospirales	f__Acutalibacteraceae	g__Fimenecus	s__Fimenecus sp000432435
PD	PD61	bin17	MAG679	d__Bacteria	p__Bacillota_A	c__Clostridia	o__Oscillospirales	f__Acutalibacteraceae	g__Fimenecus	s__Fimenecus sp000432435
PD	PD50	bin27	MAG1979	d__Bacteria	p__Bacillota_A	c__Clostridia	o__Oscillospirales	f__Acutalibacteraceae	g__Fimenecus	s__Fimenecus sp000432435
PD	PD2	bin32	MAG3273	d__Bacteria	p__Bacillota_A	c__Clostridia	o__Oscillospirales	f__Acutalibacteraceae	g__Fimenecus	s__Fimenecus sp000432435
PD	PD64	bin44	MAG1819	d__Bacteria	p__Bacillota_A	c__Clostridia	o__Oscillospirales	f__Acutalibacteraceae	g__Fimenecus	s__Fimenecus sp000432435
PD	PD87	bin65	MAG1606	d__Bacteria	p__Bacillota_A	c__Clostridia	o__Oscillospirales	f__Acutalibacteraceae	g__Fimenecus	s__Fimenecus sp000432435
PD	PD47	bin54	MAG2216	d__Bacteria	p__Bacillota_A	c__Clostridia	o__Oscillospirales	f__Acutalibacteraceae	g__Fimenecus	s__Fimenecus sp000432435
PD	PD60	bin2	MAG1020	d__Bacteria	p__Bacillota_A	c__Clostridia	o__Oscillospirales	f__Acutalibacteraceae	g__Fimenecus	s__Fimenecus sp000432435
PD	PD45	bin8	MAG1386	d__Bacteria	p__Bacillota_A	c__Clostridia	o__Oscillospirales	f__Acutalibacteraceae	g__Fimenecus	s__Fimenecus sp000432435
PD	PD46	bin75	MAG2753	d__Bacteria	p__Bacillota_A	c__Clostridia	o__Oscillospirales	f__Acutalibacteraceae	g__Fimenecus	s__Fimenecus sp000432435
PD	PD9	bin65	MAG2610	d__Bacteria	p__Bacillota_A	c__Clostridia	o__Oscillospirales	f__Acutalibacteraceae	g__Fimenecus	s__Fimenecus sp000432435
PD	PD82	bin4	MAG1678	d__Bacteria	p__Bacillota_A	c__Clostridia	o__Oscillospirales	f__Acutalibacteraceae	g__Fimenecus	s__Fimenecus sp000432435
PD	PD18	bin11	MAG2716	d__Bacteria	p__Bacillota_A	c__Clostridia	o__Oscillospirales	f__Oscillospiraceae	g__Faecousia	s__Faecousia gallistercoris
PD	PD39	bin37	MAG2227	d__Bacteria	p__Bacillota_A	c__Clostridia	o__Oscillospirales	f__Oscillospiraceae	g__Vescimonas	s__Vescimonas sp900552475
PD	PD35	bin25	MAG3200	d__Bacteria	p__Bacillota_A	c__Clostridia	o__Oscillospirales	f__Acutalibacteraceae	g__UMGS1071	s__UMGS1071 sp900542375
PD	PD78	bin17	MAG2493	d__Bacteria	p__Bacillota_A	c__Clostridia	o__Oscillospirales	f__Acutalibacteraceae	g__UMGS1071	s__UMGS1071 sp900542375
PD	PD20	bin22	MAG322	d__Bacteria	p__Bacillota_A	c__Clostridia	o__Oscillospirales	f__Acutalibacteraceae	g__UMGS1071	s__UMGS1071 sp900542375
PD	PD94	bin8	MAG159	d__Bacteria	p__Bacillota_A	c__Clostridia	o__Oscillospirales	f__Acutalibacteraceae	g__UMGS1071	s__UMGS1071 sp900542375
PD	PD100	bin39	MAG70	d__Bacteria	p__Bacillota_A	c__Clostridia	o__Oscillospirales	f__Oscillospiraceae	g__Intestinimonas	s__Intestinimonas butyriciproducens
PD	PD77	bin65	MAG1592	d__Bacteria	p__Bacillota_A	c__Clostridia	o__Peptostreptococcales	f__Anaerovoracaceae	g__Lentihominibacter	s__Lentihominibacter hominis
PD	PD61	bin60	MAG1702	d__Bacteria	p__Bacillota_A	c__Clostridia	o__Peptostreptococcales	f__Anaerovoracaceae	g__VUNA01	s__VUNA01 sp002299625
PD	PD18	bin47	MAG1091	d__Bacteria	p__Bacillota_A	c__Clostridia	o__Peptostreptococcales	f__Anaerovoracaceae	g__Lentihominibacter	s__Unclassified
PD	PD72	bin20	MAG316	d__Bacteria	p__Bacillota_A	c__Clostridia	o__Peptostreptococcales	f__Anaerovoracaceae	g__Emergencia	s__Emergencia timonensis
PD	PD10	bin23	MAG190	d__Bacteria	p__Bacillota_A	c__Clostridia	o__Peptostreptococcales	f__Anaerovoracaceae	g__Lentihominibacter	s__Lentihominibacter sp900066305
PD	PD1	bin3	MAG1968	d__Bacteria	p__Bacillota_A	c__Clostridia	o__Peptostreptococcales	f__Anaerovoracaceae	g__Fimisoma	s__Fimisoma sp000435715
PD	PD27	bin38	MAG1661	d__Bacteria	p__Bacillota_A	c__Clostridia	o__Peptostreptococcales	f__Anaerovoracaceae	g__Fimisoma	s__Fimisoma sp000435715
PD	PD46	bin29	MAG2344	d__Bacteria	p__Bacillota_A	c__Clostridia	o__Peptostreptococcales	f__Anaerovoracaceae	g__Fimisoma	s__Fimisoma sp000435715
PD	PD97	bin15	MAG1189	d__Bacteria	p__Bacillota_A	c__Clostridia	o__Peptostreptococcales	f__Anaerovoracaceae	g__Lentihominibacter	s__Lentihominibacter excrementavium
PD	PD92	bin29	MAG2579	d__Bacteria	p__Bacillota_A	c__Clostridia	o__Peptostreptococcales	f__Anaerovoracaceae	g__Lentihominibacter	s__Lentihominibacter excrementavium

PD	PD56	bin38	MAG3299	d__Bacteria	p__Bacillota_A	c__Clostridia	o__Peptostreptococcales	f__Anaerovoracaceae	g__Lentihominibacter	s__Lentihominibacter excrementavium
PD	PD85	bin11	MAG607	d__Bacteria	p__Bacillota_A	c__Clostridia	o__Peptostreptococcales	f__Peptostreptococcaceae	g__Romboutsia	s__Romboutsia timonensis
PD	PD20	bin4	MAG2863	d__Bacteria	p__Bacillota_A	c__Clostridia	o__Peptostreptococcales	f__Peptostreptococcaceae	g__Romboutsia	s__Romboutsia timonensis
PD	PD42	bin22	MAG2367	d__Bacteria	p__Bacillota_A	c__Clostridia	o__Peptostreptococcales	f__Peptostreptococcaceae	g__Romboutsia	s__Romboutsia timonensis
PD	PD78	bin7	MAG3503	d__Bacteria	p__Bacillota_A	c__Clostridia	o__Peptostreptococcales	f__Peptostreptococcaceae	g__Romboutsia	s__Romboutsia timonensis
PD	PD25	bin1	MAG3221	d__Bacteria	p__Bacillota_A	c__Clostridia	o__Peptostreptococcales	f__Peptostreptococcaceae	g__Romboutsia	s__Romboutsia timonensis
PD	PD77	bin41	MAG631	d__Bacteria	p__Bacillota_A	c__Clostridia	o__Peptostreptococcales	f__Peptostreptococcaceae	g__Romboutsia	s__Romboutsia timonensis
PD	PD64	bin3	MAG1955	d__Bacteria	p__Bacillota_A	c__Clostridia	o__Peptostreptococcales	f__Anaerovoracaceae	g__Baileyella	s__Baileyella intestinalis
PD	PD70	bin53	MAG1833	d__Bacteria	p__Bacillota_A	c__Clostridia	o__Peptostreptococcales	f__Anaerovoracaceae	g__Lentihominibacter	s__Lentihominibacter faecis
PD	PD58	bin10	MAG1204	d__Bacteria	p__Bacillota_A	c__Clostridia	o__Peptostreptococcales	f__Anaerovoracaceae	g__Lentihominibacter	s__Lentihominibacter faecis
PD	PD73	bin47	MAG824	d__Bacteria	p__Bacillota_A	c__Clostridia	o__Peptostreptococcales	f__Anaerovoracaceae	g__Lentihominibacter	s__Lentihominibacter faecis
PD	PD46	bin7	MAG3397	d__Bacteria	p__Bacillota_A	c__Clostridia	o__Peptostreptococcales	f__Anaerovoracaceae	g__Lentihominibacter	s__Lentihominibacter faecis
PD	PD85	bin30	MAG1099	d__Bacteria	p__Bacillota_A	c__Clostridia	o__Peptostreptococcales	f__Anaerovoracaceae	g__Lentihominibacter	s__Lentihominibacter faecis
PD	PD2	bin33	MAG903	d__Bacteria	p__Bacillota_A	c__Clostridia	o__Peptostreptococcales	f__Anaerovoracaceae	g__Lentihominibacter	s__Lentihominibacter faecis
PD	PD64	bin64	MAG178	d__Bacteria	p__Bacillota_A	c__Clostridia	o__Peptostreptococcales	f__Anaerovoracaceae	g__Lentihominibacter	s__Lentihominibacter faecis
PD	PD10	bin17	MAG1797	d__Bacteria	p__Bacillota_A	c__Clostridia	o__Peptostreptococcales	f__Anaerovoracaceae	g__Lentihominibacter	s__Lentihominibacter faecis
PD	PD9	bin74	MAG426	d__Bacteria	p__Bacillota_A	c__Clostridia	o__Peptostreptococcales	f__Anaerovoracaceae	g__Lentihominibacter	s__Lentihominibacter faecis
PD	PD70	bin7	MAG2422	d__Bacteria	p__Bacillota_A	c__Clostridia	o__Peptostreptococcales	f__Anaerovoracaceae	g__Gallibacter	s__Gallibacter intestinalis
PD	PD16	bin68	MAG2405	d__Bacteria	p__Bacillota_A	c__Clostridia	o__Peptostreptococcales	f__Anaerovoracaceae	g__Gallibacter	s__Gallibacter intestinalis
PD	PD40	bin34	MAG697	d__Bacteria	p__Bacillota_A	c__Clostridia	o__Peptostreptococcales	f__Anaerovoracaceae	g__Zhenpiania	s__Zhenpiania hominis
PD	PD82	bin21	MAG1059	d__Bacteria	p__Bacillota_A	c__Clostridia	o__Peptostreptococcales	f__Anaerovoracaceae	g__Zhenpiania	s__Zhenpiania hominis
PD	PD22	bin1	MAG295	d__Bacteria	p__Bacillota_A	c__Clostridia	o__Peptostreptococcales	f__Anaerovoracaceae	g__Eubacterium_B	s__Eubacterium_B sulci
PD	PD70	bin52	MAG1892	d__Bacteria	p__Bacillota_A	c__Clostridia	o__TANB77	f__CAG-508	g__CAG-269	s__CAG-269 sp001916035
PD	PD71	bin22	MAG2989	d__Bacteria	p__Bacillota_A	c__Clostridia	o__TANB77	f__CAG-508	g__CAG-269	s__CAG-269 sp001916035
PD	PD22	bin17	MAG2202	d__Bacteria	p__Bacillota_A	c__Clostridia	o__TANB77	f__CAG-508	g__CAG-245	s__CAG-245 sp000434195
PD	PD25	bin64	MAG997	d__Bacteria	p__Bacillota_A	c__Clostridia	o__TANB77	f__CAG-508	g__CAG-269	s__CAG-269 sp900556345
PD	PD19	bin37	MAG1992	d__Bacteria	p__Bacillota_A	c__Clostridia	o__TANB77	f__CAG-508	g__CAG-269	s__CAG-269 sp001916055
PD	PD25	bin37	MAG1280	d__Bacteria	p__Bacillota_A	c__Clostridia	o__TANB77	f__CAG-508	g__CAG-269	s__CAG-269 sp000437215
PD	PD41	bin68	MAG3298	d__Bacteria	p__Bacillota_A	c__Clostridia	o__TANB77	f__CAG-508	g__CAG-269	s__CAG-269 sp001916065
PD	PD12	bin38	MAG678	d__Bacteria	p__Bacillota_A	c__Clostridia	o__TANB77	f__CAG-508	g__CAG-492	s__Unclassified
PD	PD13	bin30	MAG408	d__Bacteria	p__Bacillota_A	c__Clostridia	o__TANB77	f__CAG-508	g__CAG-452	s__CAG-452 sp000434035
PD	PD50	bin50	MAG276	d__Bacteria	p__Bacillota_A	c__Clostridia	o__TANB77	f__CAG-508	g__CAG-452	s__CAG-452 sp000434035
PD	PD22	bin37	MAG2473	d__Bacteria	p__Bacillota_A	c__Clostridia	o__TANB77	f__CAG-508	g__CAG-452	s__CAG-452 sp000434035
PD	PD22	bin5	MAG698	d__Bacteria	p__Bacillota_A	c__Clostridia	o__TANB77	f__CAG-508	g__CAG-273	s__CAG-273 sp000437855
PD	PD47	bin47	MAG33	d__Bacteria	p__Bacillota_A	c__Clostridia	o__TANB77	f__CAG-508	g__CAG-245	s__CAG-245 sp000435175
PD	PD68	bin62	MAG3364	d__Bacteria	p__Bacillota_A	c__Clostridia	o__TANB77	f__CAG-508	g__CAG-245	s__CAG-245 sp000435175
PD	PD32	bin3	MAG3345	d__Bacteria	p__Bacillota_A	c__Clostridia	o__TANB77	f__CAG-508	g__CAG-245	s__CAG-245 sp000435175
PD	PD9	bin64	MAG2299	d__Bacteria	p__Bacillota_A	c__Clostridia	o__TANB77	f__CAG-508	g__CAG-269	s__CAG-269 sp003525075
PD	PD94	bin17	MAG3296	d__Bacteria	p__Bacillota_A	c__Clostridia	o__TANB77	f__CAG-508	g__CAG-273	s__CAG-273 sp003507395
PD	PD2	bin39	MAG3382	d__Bacteria	p__Bacillota_A	c__Clostridia	o__TANB77	f__CAG-508	g__CAG-273	s__CAG-273 sp003507395
PD	PD76	bin12	MAG1318	d__Bacteria	p__Bacillota_A	c__Clostridia	o__TANB77	f__CAG-508	g__CAG-269	s__CAG-269 sp000438255
PD	PD97	bin31	MAG1911	d__Bacteria	p__Bacillota_A	c__Clostridia	o__TANB77	f__CAG-508	g__CAG-492	s__CAG-492 sp000434335
PD	PD68	bin16	MAG2781	d__Bacteria	p__Bacillota_A	c__Clostridia	o__TANB77	f__CAG-508	g__CAG-269	s__CAG-269 sp000431335
PD	PD41	bin44	MAG621	d__Bacteria	p__Bacillota_A	c__Clostridia	o__TANB77	f__CAG-508	g__CAG-273	s__CAG-273 sp003534295
PD	PD12	bin37	MAG2628	d__Bacteria	p__Bacillota_A	c__Clostridia	o__TANB77	f__CAG-508	g__CAG-273	s__CAG-273 sp003534295
PD	PD64	bin16	MAG1771	d__Bacteria	p__Bacillota_A	c__Clostridia	o__TANB77	f__CAG-508	g__CAG-273	s__CAG-273 sp003534295
PD	PD73	bin59	MAG539	d__Bacteria	p__Bacillota_A	c__Clostridia	o__TANB77	f__CAG-508	g__CAG-273	s__CAG-273 sp003534295
PD	PD34	bin44	MAG1738	d__Bacteria	p__Bacillota_A	c__Clostridia	o__Tissierellales	f__Peptoniphilaceae	g__Fenollaria	s__Fenollaria massiliensis
PD	PD22	bin16	MAG734	d__Bacteria	p__Bacillota_A	c__Clostridia	o__UBA1381	f__UBA9506	g__Congzhengia	s__Congzhengia minquanensis
PD	PD9	bin27	MAG1416	d__Bacteria	p__Bacillota_A	c__Clostridia	o__UBA1381	f__UBA9506	g__Congzhengia	s__Congzhengia minquanensis
PD	PD60	bin10	MAG1922	d__Bacteria	p__Bacillota_A	c__Clostridia	o__UBA1381	f__UBA1381	g__CAG-41	s__CAG-41 sp900066215

PD	PD9	bin17	MAG861	d__Bacteria	p__Bacillota_A	c__Clostridia	o__UBA1381	f__UBA1381	g__CAG-41	s__CAG-41 sp900066215
PD	PD10	bin37	MAG2068	d__Bacteria	p__Bacillota_A	c__Clostridia	o__UBA1381	f__UBA1381	g__CAG-41	s__CAG-41 sp900066215
PD	PD32	bin8	MAG304	d__Bacteria	p__Bacillota_A	c__Clostridia	o__UBA1381	f__UBA1381	g__CAG-41	s__CAG-41 sp900066215
PD	PD68	bin35	MAG1622	d__Bacteria	p__Bacillota_A	c__Clostridia	o__UBA1381	f__UBA1381	g__CAG-41	s__CAG-41 sp900066215
PD	PD78	bin2	MAG1238	d__Bacteria	p__Bacillota_A	c__Clostridia	o__UBA1381	f__UBA1381	g__CAG-41	s__CAG-41 sp900066215
PD	PD76	bin58	MAG895	d__Bacteria	p__Bacillota_A	c__Clostridia	o__UBA1381	f__UBA1381	g__CAG-41	s__CAG-41 sp900066215
PD	PD14	bin38	MAG754	d__Bacteria	p__Bacillota_A	c__Clostridia	o__UBA1381	f__UBA1381	g__CAG-41	s__CAG-41 sp900066215
PD	PD46	bin68	MAG3314	d__Bacteria	p__Bacillota_A	c__Clostridia	o__UBA1381	f__UBA1381	g__CAG-41	s__CAG-41 sp900066215
PD	PD23	bin9	MAG3180	d__Bacteria	p__Bacillota_A	c__Clostridia	o__UBA1381	f__UBA1381	g__CAG-41	s__CAG-41 sp900066215
PD	PD81	bin28	MAG2867	d__Bacteria	p__Bacillota_A	c__Clostridia	o__UBA1381	f__UBA1381	g__CAG-41	s__CAG-41 sp900066215
PD	PD35	bin28	MAG3084	d__Bacteria	p__Bacillota_A	c__Clostridia	o__UBA1381	f__UBA1381	g__CAG-41	s__CAG-41 sp900066215
PD	PD29	bin4	MAG1986	d__Bacteria	p__Bacillota_A	c__Clostridia	o__UBA1381	f__UBA1381	g__CAG-41	s__CAG-41 sp900066215
PD	PD47	bin32	MAG1966	d__Bacteria	p__Bacillota_A	c__Clostridia	o__UBA1381	f__UBA1381	g__CAG-41	s__CAG-41 sp900066215
PD	PD45	bin61	MAG2277	d__Bacteria	p__Bacillota_A	c__Clostridia	o__UBA1381	f__UBA1381	g__CAG-41	s__CAG-41 sp900066215
PD	PD48	bin37	MAG250	d__Bacteria	p__Bacillota_A	c__Clostridia	o__UBA1381	f__UBA1381	g__CAG-41	s__CAG-41 sp900066215
PD	PD83	bin13	MAG522	d__Bacteria	p__Bacillota_A	c__Clostridia	o__UBA1381	f__UBA1381	g__CAG-41	s__CAG-41 sp900066215
PD	PD3	bin33	MAG1654	d__Bacteria	p__Bacillota_A	c__Clostridia	o__UBA1381	f__UBA1381	g__CAG-41	s__CAG-41 sp900066215
PD	PD40	bin21	MAG2176	d__Bacteria	p__Bacillota_A	c__Clostridia	o__UBA1381	f__UBA1381	g__CAG-41	s__CAG-41 sp900066215
PD	PD69	bin45	MAG1098	d__Bacteria	p__Bacillota_A	c__Clostridia	o__UBA1381	f__UBA1381	g__CAG-41	s__CAG-41 sp900066215
PD	PD75	bin10	MAG2987	d__Bacteria	p__Bacillota_A	c__Clostridia	o__UBA1381	f__UBA1381	g__CAG-41	s__CAG-41 sp900066215
PD	PD44	bin10	MAG1929	d__Bacteria	p__Bacillota_A	c__Clostridia	o__UBA1381	f__UBA1381	g__CAG-41	s__CAG-41 sp900066215
PD	PD2	bin38	MAG2192	d__Bacteria	p__Bacillota_A	c__Clostridia	o__UBA1381	f__UBA1381	g__CAG-41	s__CAG-41 sp900066215
PD	PD64	bin46	MAG346	d__Bacteria	p__Bacillota_A	c__Clostridia	o__UBA1381	f__UBA1381	g__CAG-41	s__CAG-41 sp900066215
PD	PD64	bin12	MAG2615	d__Bacteria	p__Bacillota_A	c__Clostridia	o__UBA1381	f__UBA9506	g__Congzhengia	s__Unclassified
PD	PD58	bin43	MAG1648	d__Bacteria	p__Bacillota_A	c__Clostridia	o__UBA1381	f__UMGS1253	g__Aphodoplasma	s__Aphodoplasma sp900550065
PD	PD29	bin31	MAG34	d__Bacteria	p__Bacillota_A	c__Clostridia	o__UMGS1883	f__HGM11501	g__HGM11501	s__HGM11501 sp900766735
Con	Con34	bin23	MAG3372	d__Bacteria	p__Bacillota_A	c__Clostridia	o__Christensenellales	f__CAG-74	g__Ventricola	s__Ventricola sp900542395
Con	Con27	bin53	MAG177	d__Bacteria	p__Bacillota_A	c__Clostridia	o__Christensenellales	f__CAG-74	g__UBA11524	s__UBA11524 sp000437595
Con	Con4	bin59	MAG2642	d__Bacteria	p__Bacillota_A	c__Clostridia	o__Christensenellales	f__CAG-74	g__UBA11524	s__UBA11524 sp000437595
Con	Con49	bin23	MAG2850	d__Bacteria	p__Bacillota_A	c__Clostridia	o__Christensenellales	f__CAG-74	g__UBA11524	s__UBA11524 sp000437595
Con	Con10	bin20	MAG3181	d__Bacteria	p__Bacillota_A	c__Clostridia	o__Christensenellales	f__CAG-74	g__UBA11524	s__UBA11524 sp000437595
Con	Con39	bin26	MAG3020	d__Bacteria	p__Bacillota_A	c__Clostridia	o__Christensenellales	f__CAG-74	g__UBA11524	s__UBA11524 sp000437595
Con	Con17	bin1	MAG2040	d__Bacteria	p__Bacillota_A	c__Clostridia	o__Christensenellales	f__CAG-74	g__UBA11524	s__UBA11524 sp000437595
Con	Con2	bin60	MAG2157	d__Bacteria	p__Bacillota_A	c__Clostridia	o__Christensenellales	f__CAG-74	g__Ventricola	s__Ventricola sp900542445
Con	Con4	bin16	MAG2775	d__Bacteria	p__Bacillota_A	c__Clostridia	o__Christensenellales	f__CAG-74	g__Ventricola	s__Ventricola sp900542445
Con	Con26	bin21	MAG2969	d__Bacteria	p__Bacillota_A	c__Clostridia	o__Christensenellales	f__CAG-74	g__Limiplasma	s__Limiplasma sp900548145
Con	Con49	bin52	MAG348	d__Bacteria	p__Bacillota_A	c__Clostridia	o__Christensenellales	f__CAG-74	g__Limiplasma	s__Limiplasma sp900548145
Con	Con2	bin69	MAG1563	d__Bacteria	p__Bacillota_A	c__Clostridia	o__Christensenellales	f__CAG-74	g__Faecivicinus	s__Faecivicinus sp900553265
Con	Con34	bin9	MAG702	d__Bacteria	p__Bacillota_A	c__Clostridia	o__Christensenellales	f__CAG-138	g__PeH17	s__PeH17 sp000435055
Con	Con29	bin15	MAG1080	d__Bacteria	p__Bacillota_A	c__Clostridia	o__Clostridiales	f__Clostridiaceae	g__Clostridium	s__Clostridium tertium
Con	Con31	bin34	MAG718	d__Bacteria	p__Bacillota_A	c__Clostridia	o__Clostridiales	f__Clostridiaceae	g__Clostridium	s__Clostridium saudiense
Con	Con4	bin10	MAG2407	d__Bacteria	p__Bacillota_A	c__Clostridia	o__Clostridiales	f__Clostridiaceae	g__Clostridium	s__Clostridium sp900540255
Con	Con45	bin1	MAG2907	d__Bacteria	p__Bacillota_A	c__Clostridia	o__Eubacteriales	f__Eubacteriaceae	g__Eubacterium	s__Eubacterium callanderi
Con	Con51	bin30	MAG869	d__Bacteria	p__Bacillota_A	c__Clostridia	o__Lachnospirales	f__Lachnospiraceae	g__Pararoseburia	s__Pararoseburia sp900546435
Con	Con48	bin20	MAG2789	d__Bacteria	p__Bacillota_A	c__Clostridia	o__Lachnospirales	f__CAG-274	g__CAG-274	s__CAG-274 sp900545305
Con	Con22	bin24	MAG1820	d__Bacteria	p__Bacillota_A	c__Clostridia	o__Lachnospirales	f__CAG-274	g__CAG-274	s__CAG-274 sp900545305
Con	Con27	bin39	MAG428	d__Bacteria	p__Bacillota_A	c__Clostridia	o__Lachnospirales	f__CAG-274	g__CAG-274	s__CAG-274 sp900545305
Con	Con10	bin47	MAG1370	d__Bacteria	p__Bacillota_A	c__Clostridia	o__Lachnospirales	f__Lachnospiraceae	g__Acetatifactor	s__Acetatifactor intestinalis
Con	Con11	bin19	MAG2258	d__Bacteria	p__Bacillota_A	c__Clostridia	o__Lachnospirales	f__Lachnospiraceae	g__Anaerostipes	s__Anaerostipes caccae
Con	Con24	bin8	MAG3151	d__Bacteria	p__Bacillota_A	c__Clostridia	o__Lachnospirales	f__Anaerotignaceae	g__Anaerotignum	s__Anaerotignum faecicola
Con	Con11	bin23	MAG424	d__Bacteria	p__Bacillota_A	c__Clostridia	o__Lachnospirales	f__Lachnospiraceae	g__Blautia_A	s__Blautia_A caecimuris

Con	Con44	bin37	MAG2639	d__Bacteria	p__Bacillota_A	c__Clostridia	o__Lachnospirales	f__Lachnospiraceae	g__Blautia_A	s__Blautia_A caecimuris
Con	Con8	bin35	MAG871	d__Bacteria	p__Bacillota_A	c__Clostridia	o__Lachnospirales	f__Lachnospiraceae	g__Hominisplanchenecus	s__Hominisplanchenecus faecis
Con	Con52	bin10	MAG2567	d__Bacteria	p__Bacillota_A	c__Clostridia	o__Lachnospirales	f__Lachnospiraceae	g__Hominisplanchenecus	s__Hominisplanchenecus faecis
Con	Con35	bin22	MAG858	d__Bacteria	p__Bacillota_A	c__Clostridia	o__Lachnospirales	f__Lachnospiraceae	g__Hominisplanchenecus	s__Hominisplanchenecus faecis
Con	Con32	bin39	MAG788	d__Bacteria	p__Bacillota_A	c__Clostridia	o__Lachnospirales	f__Lachnospiraceae	g__Blautia_A	s__Blautia_A massiliensis
Con	Con20	bin26	MAG1237	d__Bacteria	p__Bacillota_A	c__Clostridia	o__Lachnospirales	f__Lachnospiraceae	g__Blautia_A	s__Blautia_A massiliensis
Con	Con3	bin4	MAG2984	d__Bacteria	p__Bacillota_A	c__Clostridia	o__Lachnospirales	f__Lachnospiraceae	g__Blautia_A	s__Blautia_A massiliensis
Con	Con17	bin18	MAG3202	d__Bacteria	p__Bacillota_A	c__Clostridia	o__Lachnospirales	f__Lachnospiraceae	g__Blautia_A	s__Blautia_A massiliensis
Con	Con48	bin35	MAG2516	d__Bacteria	p__Bacillota_A	c__Clostridia	o__Lachnospirales	f__Lachnospiraceae	g__Blautia_A	s__Blautia_A massiliensis
Con	Con40	bin21	MAG1925	d__Bacteria	p__Bacillota_A	c__Clostridia	o__Lachnospirales	f__Lachnospiraceae	g__Blautia_A	s__Blautia_A massiliensis
Con	Con45	bin54	MAG3017	d__Bacteria	p__Bacillota_A	c__Clostridia	o__Lachnospirales	f__Lachnospiraceae	g__Butyribacter	s__Butyribacter sp003524945
Con	Con53	bin24	MAG442	d__Bacteria	p__Bacillota_A	c__Clostridia	o__Lachnospirales	f__Lachnospiraceae	g__Hungatella	s__Hungatella effluvii
Con	Con30	bin19	MAG451	d__Bacteria	p__Bacillota_A	c__Clostridia	o__Lachnospirales	f__Lachnospiraceae	g__Hungatella	s__Hungatella effluvii
Con	Con40	bin12	MAG949	d__Bacteria	p__Bacillota_A	c__Clostridia	o__Lachnospirales	f__Lachnospiraceae	g__Anaerostipes	s__Anaerostipes hadrus_A
Con	Con49	bin89	MAG1475	d__Bacteria	p__Bacillota_A	c__Clostridia	o__Lachnospirales	f__Lachnospiraceae	g__Anaerostipes	s__Anaerostipes hadrus_A
Con	Con40	bin26	MAG2470	d__Bacteria	p__Bacillota_A	c__Clostridia	o__Lachnospirales	f__Lachnospiraceae	g__Coprococcus	s__Coprococcus sp000154245
Con	Con1	bin1	MAG66	d__Bacteria	p__Bacillota_A	c__Clostridia	o__Lachnospirales	f__Lachnospiraceae	g__CAG-882	s__CAG-882 sp003486385
Con	Con12	bin32	MAG247	d__Bacteria	p__Bacillota_A	c__Clostridia	o__Lachnospirales	f__Lachnospiraceae	g__Clostridium_AP	s__Clostridium_AP scindens
Con	Con6	bin13	MAG511	d__Bacteria	p__Bacillota_A	c__Clostridia	o__Lachnospirales	f__Lachnospiraceae	g__Coprococcus_A	s__Coprococcus_A catus
Con	Con12	bin19	MAG2321	d__Bacteria	p__Bacillota_A	c__Clostridia	o__Lachnospirales	f__Lachnospiraceae	g__Enterocloster	s__Enterocloster lavalensis
Con	Con30	bin11	MAG663	d__Bacteria	p__Bacillota_A	c__Clostridia	o__Lachnospirales	f__Lachnospiraceae	g__Clostridium_Q	s__Clostridium_Q symbiosum
Con	Con44	bin26	MAG3227	d__Bacteria	p__Bacillota_A	c__Clostridia	o__Lachnospirales	f__Lachnospiraceae	g__Schaedlerella	s__Schaedlerella glycyrrhizinilytica_A
Con	Con38	bin66	MAG2190	d__Bacteria	p__Bacillota_A	c__Clostridia	o__Lachnospirales	f__Lachnospiraceae	g__Dorea_A	s__Dorea_A longicatena
Con	Con2	bin35	MAG1811	d__Bacteria	p__Bacillota_A	c__Clostridia	o__Lachnospirales	f__Lachnospiraceae	g__Dorea_A	s__Dorea_A longicatena
Con	Con1	bin18	MAG517	d__Bacteria	p__Bacillota_A	c__Clostridia	o__Lachnospirales	f__Lachnospiraceae	g__Acetatifactor	s__Acetatifactor sp900066565
Con	Con35	bin16	MAG3140	d__Bacteria	p__Bacillota_A	c__Clostridia	o__Lachnospirales	f__Lachnospiraceae	g__Acetatifactor	s__Acetatifactor sp900066565
Con	Con5	bin41	MAG1898	d__Bacteria	p__Bacillota_A	c__Clostridia	o__Lachnospirales	f__Lachnospiraceae	g__Acetatifactor	s__Acetatifactor sp900066565
Con	Con45	bin57	MAG705	d__Bacteria	p__Bacillota_A	c__Clostridia	o__Lachnospirales	f__Lachnospiraceae	g__Acetatifactor	s__Acetatifactor sp900066565
Con	Con27	bin37	MAG3048	d__Bacteria	p__Bacillota_A	c__Clostridia	o__Lachnospirales	f__Lachnospiraceae	g__Agathobacter	s__Agathobacter rectalis
Con	Con4	bin26	MAG1538	d__Bacteria	p__Bacillota_A	c__Clostridia	o__Lachnospirales	f__Lachnospiraceae	g__Agathobacter	s__Agathobacter rectalis
Con	Con48	bin34	MAG2790	d__Bacteria	p__Bacillota_A	c__Clostridia	o__Lachnospirales	f__Lachnospiraceae	g__Agathobacter	s__Agathobacter rectalis
Con	Con34	bin31	MAG3305	d__Bacteria	p__Bacillota_A	c__Clostridia	o__Lachnospirales	f__Lachnospiraceae	g__Agathobacter	s__Agathobacter rectalis
Con	Con38	bin63	MAG409	d__Bacteria	p__Bacillota_A	c__Clostridia	o__Lachnospirales	f__Lachnospiraceae	g__Agathobacter	s__Agathobacter rectalis
Con	Con39	bin42	MAG1933	d__Bacteria	p__Bacillota_A	c__Clostridia	o__Lachnospirales	f__Lachnospiraceae	g__Agathobacter	s__Agathobacter rectalis
Con	Con3	bin5	MAG1617	d__Bacteria	p__Bacillota_A	c__Clostridia	o__Lachnospirales	f__Lachnospiraceae	g__Agathobacter	s__Agathobacter rectalis
Con	Con8	bin3	MAG2017	d__Bacteria	p__Bacillota_A	c__Clostridia	o__Lachnospirales	f__Lachnospiraceae	g__Agathobacter	s__Agathobacter rectalis
Con	Con50	bin15	MAG1796	d__Bacteria	p__Bacillota_A	c__Clostridia	o__Lachnospirales	f__Lachnospiraceae	g__Agathobacter	s__Agathobacter rectalis
Con	Con20	bin8	MAG3106	d__Bacteria	p__Bacillota_A	c__Clostridia	o__Lachnospirales	f__Lachnospiraceae	g__Agathobacter	s__Agathobacter rectalis
Con	Con19	bin1	MAG165	d__Bacteria	p__Bacillota_A	c__Clostridia	o__Lachnospirales	f__Lachnospiraceae	g__Agathobacter	s__Agathobacter rectalis
Con	Con35	bin39	MAG1945	d__Bacteria	p__Bacillota_A	c__Clostridia	o__Lachnospirales	f__Lachnospiraceae	g__RUG115	s__RUG115 sp900066395
Con	Con3	bin38	MAG1915	d__Bacteria	p__Bacillota_A	c__Clostridia	o__Lachnospirales	f__Lachnospiraceae	g__RUG115	s__RUG115 sp900066395
Con	Con20	bin38	MAG770	d__Bacteria	p__Bacillota_A	c__Clostridia	o__Lachnospirales	f__Lachnospiraceae	g__Clostridium_Q	s__Clostridium_Q fessum
Con	Con53	bin21	MAG418	d__Bacteria	p__Bacillota_A	c__Clostridia	o__Lachnospirales	f__Lachnospiraceae	g__Clostridium_Q	s__Clostridium_Q fessum
Con	Con19	bin41	MAG1866	d__Bacteria	p__Bacillota_A	c__Clostridia	o__Lachnospirales	f__Lachnospiraceae	g__Anaerobutyricum	s__Anaerobutyricum soehngenii
Con	Con54	bin16	MAG2968	d__Bacteria	p__Bacillota_A	c__Clostridia	o__Lachnospirales	f__Lachnospiraceae	g__Anaerobutyricum	s__Anaerobutyricum soehngenii
Con	Con32	bin14	MAG691	d__Bacteria	p__Bacillota_A	c__Clostridia	o__Lachnospirales	f__Lachnospiraceae	g__Anaerobutyricum	s__Anaerobutyricum soehngenii
Con	Con3	bin67	MAG226	d__Bacteria	p__Bacillota_A	c__Clostridia	o__Lachnospirales	f__Lachnospiraceae	g__Coprococcus	s__Coprococcus eutactus_A
Con	Con49	bin18	MAG2522	d__Bacteria	p__Bacillota_A	c__Clostridia	o__Lachnospirales	f__Lachnospiraceae	g__Coprococcus	s__Coprococcus sp900066115
Con	Con8	bin5	MAG3317	d__Bacteria	p__Bacillota_A	c__Clostridia	o__Lachnospirales	f__Lachnospiraceae	g__Butyribacter	s__Butyribacter hominis
Con	Con11	bin9	MAG1658	d__Bacteria	p__Bacillota_A	c__Clostridia	o__Lachnospirales	f__Lachnospiraceae	g__Ventrimonas	s__Ventrimonas sp900538475
Con	Con32	bin36	MAG1906	d__Bacteria	p__Bacillota_A	c__Clostridia	o__Lachnospirales	f__Lachnospiraceae	g__Roseburia	s__Roseburia hominis

Con	Con2	bin68	MAG1923	d__Bacteria	p__Bacillota_A	c__Clostridia	o__Lachnospirales	f__Lachnospiraceae	g__Roseburia	s__Roseburia hominis
Con	Con1	bin30	MAG1831	d__Bacteria	p__Bacillota_A	c__Clostridia	o__Lachnospirales	f__Lachnospiraceae	g__Roseburia	s__Roseburia hominis
Con	Con51	bin48	MAG382	d__Bacteria	p__Bacillota_A	c__Clostridia	o__Lachnospirales	f__Lachnospiraceae	g__Roseburia	s__Roseburia hominis
Con	Con24	bin49	MAG497	d__Bacteria	p__Bacillota_A	c__Clostridia	o__Lachnospirales	f__Lachnospiraceae	g__Roseburia	s__Roseburia hominis
Con	Con34	bin3	MAG2333	d__Bacteria	p__Bacillota_A	c__Clostridia	o__Lachnospirales	f__Lachnospiraceae	g__Roseburia	s__Roseburia hominis
Con	Con35	bin6	MAG2195	d__Bacteria	p__Bacillota_A	c__Clostridia	o__Lachnospirales	f__Lachnospiraceae	g__Roseburia	s__Roseburia hominis
Con	Con6	bin25	MAG3304	d__Bacteria	p__Bacillota_A	c__Clostridia	o__Lachnospirales	f__Lachnospiraceae	g__Lachnospira	s__Lachnospira sp000436475
Con	Con31	bin33	MAG269	d__Bacteria	p__Bacillota_A	c__Clostridia	o__Lachnospirales	f__Lachnospiraceae	g__Eisenbergiella	s__Eisenbergiella porci
Con	Con28	bin9	MAG2609	d__Bacteria	p__Bacillota_A	c__Clostridia	o__Lachnospirales	f__Lachnospiraceae	g__Ruminococcus_B	s__Ruminococcus_B gnavus
Con	Con22	bin18	MAG229	d__Bacteria	p__Bacillota_A	c__Clostridia	o__Lachnospirales	f__Lachnospiraceae	g__Ruminococcus_B	s__Ruminococcus_B gnavus
Con	Con44	bin3	MAG713	d__Bacteria	p__Bacillota_A	c__Clostridia	o__Lachnospirales	f__Lachnospiraceae	g__Ruminococcus_B	s__Ruminococcus_B gnavus
Con	Con11	bin13	MAG494	d__Bacteria	p__Bacillota_A	c__Clostridia	o__Lachnospirales	f__Lachnospiraceae	g__Ruminococcus_B	s__Ruminococcus_B gnavus
Con	Con22	bin27	MAG1910	d__Bacteria	p__Bacillota_A	c__Clostridia	o__Lachnospirales	f__Lachnospiraceae	g__Faecalimonas	s__Faecalimonas phoceensis
Con	Con28	bin31	MAG1073	d__Bacteria	p__Bacillota_A	c__Clostridia	o__Lachnospirales	f__Lachnospiraceae	g__Faecalimonas	s__Faecalimonas phoceensis
Con	Con53	bin4	MAG1402	d__Bacteria	p__Bacillota_A	c__Clostridia	o__Lachnospirales	f__Lachnospiraceae	g__Faecalimonas	s__Faecalimonas phoceensis
Con	Con30	bin28	MAG3254	d__Bacteria	p__Bacillota_A	c__Clostridia	o__Lachnospirales	f__Lachnospiraceae	g__Blautia	s__Blautia hansenii
Con	Con31	bin29	MAG3339	d__Bacteria	p__Bacillota_A	c__Clostridia	o__Lachnospirales	f__Lachnospiraceae	g__Blautia_A	s__Blautia_A sp003471165
Con	Con39	bin16	MAG3491	d__Bacteria	p__Bacillota_A	c__Clostridia	o__Lachnospirales	f__Lachnospiraceae	g__Blautia_A	s__Blautia_A sp003471165
Con	Con34	bin8	MAG435	d__Bacteria	p__Bacillota_A	c__Clostridia	o__Lachnospirales	f__Lachnospiraceae	g__Blautia_A	s__Blautia_A sp003471165
Con	Con53	bin27	MAG310	d__Bacteria	p__Bacillota_A	c__Clostridia	o__Lachnospirales	f__Lachnospiraceae	g__Blautia_A	s__Blautia_A sp003471165
Con	Con40	bin3	MAG1431	d__Bacteria	p__Bacillota_A	c__Clostridia	o__Lachnospirales	f__Lachnospiraceae	g__Blautia_A	s__Blautia_A sp003471165
Con	Con44	bin18	MAG2161	d__Bacteria	p__Bacillota_A	c__Clostridia	o__Lachnospirales	f__Lachnospiraceae	g__Blautia	s__Blautia sp000432195
Con	Con3	bin20	MAG1163	d__Bacteria	p__Bacillota_A	c__Clostridia	o__Lachnospirales	f__Lachnospiraceae	g__CAG-95	s__CAG-95 sp000438155
Con	Con12	bin15	MAG1981	d__Bacteria	p__Bacillota_A	c__Clostridia	o__Lachnospirales	f__Lachnospiraceae	g__Eisenbergiella	s__Eisenbergiella tayi
Con	Con1	bin17	MAG2132	d__Bacteria	p__Bacillota_A	c__Clostridia	o__Lachnospirales	f__Lachnospiraceae	g__Anaerobutyricum	s__Anaerobutyricum hallii
Con	Con38	bin45	MAG652	d__Bacteria	p__Bacillota_A	c__Clostridia	o__Lachnospirales	f__Lachnospiraceae	g__Anaerobutyricum	s__Anaerobutyricum hallii
Con	Con48	bin24	MAG909	d__Bacteria	p__Bacillota_A	c__Clostridia	o__Lachnospirales	f__Lachnospiraceae	g__Anaerobutyricum	s__Anaerobutyricum hallii
Con	Con22	bin15	MAG285	d__Bacteria	p__Bacillota_A	c__Clostridia	o__Lachnospirales	f__Lachnospiraceae	g__Anaerobutyricum	s__Anaerobutyricum hallii
Con	Con2	bin46	MAG57	d__Bacteria	p__Bacillota_A	c__Clostridia	o__Lachnospirales	f__Lachnospiraceae	g__Anaerobutyricum	s__Anaerobutyricum hallii
Con	Con4	bin5	MAG1356	d__Bacteria	p__Bacillota_A	c__Clostridia	o__Lachnospirales	f__Lachnospiraceae	g__Anaerobutyricum	s__Anaerobutyricum hallii
Con	Con44	bin16	MAG228	d__Bacteria	p__Bacillota_A	c__Clostridia	o__Lachnospirales	f__Lachnospiraceae	g__Anaerobutyricum	s__Anaerobutyricum hallii
Con	Con20	bin25	MAG261	d__Bacteria	p__Bacillota_A	c__Clostridia	o__Lachnospirales	f__Lachnospiraceae	g__Clostridium_AP	s__Clostridium_AP sp000509125
Con	Con28	bin15	MAG2889	d__Bacteria	p__Bacillota_A	c__Clostridia	o__Lachnospirales	f__Lachnospiraceae	g__Anaerostipes	s__Anaerostipes hadrus
Con	Con45	bin20	MAG2978	d__Bacteria	p__Bacillota_A	c__Clostridia	o__Lachnospirales	f__Lachnospiraceae	g__Anaerostipes	s__Anaerostipes hadrus
Con	Con12	bin49	MAG1511	d__Bacteria	p__Bacillota_A	c__Clostridia	o__Lachnospirales	f__Lachnospiraceae	g__Anaerostipes	s__Anaerostipes hadrus
Con	Con2	bin19	MAG3499	d__Bacteria	p__Bacillota_A	c__Clostridia	o__Lachnospirales	f__Lachnospiraceae	g__Anaerostipes	s__Anaerostipes hadrus
Con	Con17	bin44	MAG3418	d__Bacteria	p__Bacillota_A	c__Clostridia	o__Lachnospirales	f__Lachnospiraceae	g__Anaerostipes	s__Anaerostipes hadrus
Con	Con48	bin19	MAG2554	d__Bacteria	p__Bacillota_A	c__Clostridia	o__Lachnospirales	f__Lachnospiraceae	g__Anaerostipes	s__Anaerostipes hadrus
Con	Con19	bin29	MAG2979	d__Bacteria	p__Bacillota_A	c__Clostridia	o__Lachnospirales	f__Lachnospiraceae	g__Anaerostipes	s__Anaerostipes hadrus
Con	Con22	bin31	MAG3415	d__Bacteria	p__Bacillota_A	c__Clostridia	o__Lachnospirales	f__Lachnospiraceae	g__Anaerostipes	s__Anaerostipes hadrus
Con	Con34	bin57	MAG1508	d__Bacteria	p__Bacillota_A	c__Clostridia	o__Lachnospirales	f__Lachnospiraceae	g__Anaerostipes	s__Anaerostipes hadrus
Con	Con20	bin1	MAG3125	d__Bacteria	p__Bacillota_A	c__Clostridia	o__Lachnospirales	f__Lachnospiraceae	g__Anaerostipes	s__Anaerostipes hadrus
Con	Con4	bin13	MAG2565	d__Bacteria	p__Bacillota_A	c__Clostridia	o__Lachnospirales	f__Lachnospiraceae	g__Anaerostipes	s__Anaerostipes hadrus
Con	Con25	bin33	MAG1757	d__Bacteria	p__Bacillota_A	c__Clostridia	o__Lachnospirales	f__Lachnospiraceae	g__Anaerostipes	s__Anaerostipes hadrus
Con	Con35	bin42	MAG1557	d__Bacteria	p__Bacillota_A	c__Clostridia	o__Lachnospirales	f__Lachnospiraceae	g__Anaerostipes	s__Anaerostipes hadrus
Con	Con50	bin29	MAG1825	d__Bacteria	p__Bacillota_A	c__Clostridia	o__Lachnospirales	f__Lachnospiraceae	g__Anaerostipes	s__Anaerostipes hadrus
Con	Con1	bin44	MAG2191	d__Bacteria	p__Bacillota_A	c__Clostridia	o__Lachnospirales	f__Lachnospiraceae	g__Anaerostipes	s__Anaerostipes hadrus
Con	Con52	bin5	MAG781	d__Bacteria	p__Bacillota_A	c__Clostridia	o__Lachnospirales	f__Lachnospiraceae	g__Anaerostipes	s__Anaerostipes hadrus
Con	Con3	bin52	MAG2637	d__Bacteria	p__Bacillota_A	c__Clostridia	o__Lachnospirales	f__Lachnospiraceae	g__Anaerostipes	s__Anaerostipes hadrus
Con	Con27	bin44	MAG2303	d__Bacteria	p__Bacillota_A	c__Clostridia	o__Lachnospirales	f__Lachnospiraceae	g__Blautia_A	s__Blautia_A faecis
Con	Con18	bin23	MAG3176	d__Bacteria	p__Bacillota_A	c__Clostridia	o__Lachnospirales	f__Lachnospiraceae	g__Blautia_A	s__Blautia_A faecis

Con	Con22	bin32	MAG1129	d__Bacteria	p__Bacillota_A	c__Clostridia	o__Lachnospirales	f__Lachnospiraceae	g__Blautia_A	s__Blautia_A faecis
Con	Con28	bin32	MAG3086	d__Bacteria	p__Bacillota_A	c__Clostridia	o__Lachnospirales	f__Lachnospiraceae	g__Blautia_A	s__Blautia_A faecis
Con	Con24	bin1	MAG254	d__Bacteria	p__Bacillota_A	c__Clostridia	o__Lachnospirales	f__Lachnospiraceae	g__Blautia_A	s__Blautia_A faecis
Con	Con1	bin25	MAG830	d__Bacteria	p__Bacillota_A	c__Clostridia	o__Lachnospirales	f__Lachnospiraceae	g__Blautia_A	s__Blautia_A faecis
Con	Con10	bin68	MAG2606	d__Bacteria	p__Bacillota_A	c__Clostridia	o__Lachnospirales	f__Lachnospiraceae	g__Blautia_A	s__Blautia_A faecis
Con	Con40	bin28	MAG709	d__Bacteria	p__Bacillota_A	c__Clostridia	o__Lachnospirales	f__Lachnospiraceae	g__Blautia_A	s__Blautia_A faecis
Con	Con17	bin41	MAG929	d__Bacteria	p__Bacillota_A	c__Clostridia	o__Lachnospirales	f__Lachnospiraceae	g__Blautia_A	s__Blautia_A faecis
Con	Con2	bin42	MAG2393	d__Bacteria	p__Bacillota_A	c__Clostridia	o__Lachnospirales	f__Lachnospiraceae	g__Blautia_A	s__Blautia_A faecis
Con	Con17	bin5	MAG1639	d__Bacteria	p__Bacillota_A	c__Clostridia	o__Lachnospirales	f__Lachnospiraceae	g__Pararoseburia	s__Pararoseburia lenta
Con	Con4	bin62	MAG3428	d__Bacteria	p__Bacillota_A	c__Clostridia	o__Lachnospirales	f__Lachnospiraceae	g__Pararoseburia	s__Pararoseburia lenta
Con	Con38	bin34	MAG2685	d__Bacteria	p__Bacillota_A	c__Clostridia	o__Lachnospirales	f__Lachnospiraceae	g__Coprococcus	s__Coprococcus eutactus
Con	Con11	bin31	MAG1502	d__Bacteria	p__Bacillota_A	c__Clostridia	o__Lachnospirales	f__Lachnospiraceae	g__Blautia	s__Blautia sp001304935
Con	Con32	bin10	MAG2843	d__Bacteria	p__Bacillota_A	c__Clostridia	o__Lachnospirales	f__Lachnospiraceae	g__Eisenbergiella	s__Eisenbergiella sp900066775
Con	Con1	bin39	MAG2282	d__Bacteria	p__Bacillota_A	c__Clostridia	o__Lachnospirales	f__Lachnospiraceae	g__Eisenbergiella	s__Eisenbergiella sp900066775
Con	Con51	bin7	MAG3476	d__Bacteria	p__Bacillota_A	c__Clostridia	o__Lachnospirales	f__Lachnospiraceae	g__Eisenbergiella	s__Eisenbergiella sp900066775
Con	Con4	bin25	MAG852	d__Bacteria	p__Bacillota_A	c__Clostridia	o__Lachnospirales	f__Lachnospiraceae	g__Eisenbergiella	s__Eisenbergiella sp900066775
Con	Con17	bin4	MAG3213	d__Bacteria	p__Bacillota_A	c__Clostridia	o__Lachnospirales	f__Lachnospiraceae	g__Eisenbergiella	s__Eisenbergiella sp900066775
Con	Con12	bin22	MAG998	d__Bacteria	p__Bacillota_A	c__Clostridia	o__Lachnospirales	f__Lachnospiraceae	g__Copromonas	s__Copromonas sp900066055
Con	Con25	bin3	MAG109	d__Bacteria	p__Bacillota_A	c__Clostridia	o__Lachnospirales	f__Lachnospiraceae	g__Copromonas	s__Copromonas sp000435795
Con	Con2	bin1	MAG1948	d__Bacteria	p__Bacillota_A	c__Clostridia	o__Lachnospirales	f__Lachnospiraceae	g__Dorea	s__Dorea formicigenerans
Con	Con24	bin43	MAG256	d__Bacteria	p__Bacillota_A	c__Clostridia	o__Lachnospirales	f__Lachnospiraceae	g__Dorea	s__Dorea formicigenerans
Con	Con30	bin13	MAG1104	d__Bacteria	p__Bacillota_A	c__Clostridia	o__Lachnospirales	f__Lachnospiraceae	g__Faecalimonas	s__Faecalimonas umbilicata
Con	Con44	bin24	MAG1267	d__Bacteria	p__Bacillota_A	c__Clostridia	o__Lachnospirales	f__Lachnospiraceae	g__Faecalimonas	s__Faecalimonas umbilicata
Con	Con25	bin10	MAG1768	d__Bacteria	p__Bacillota_A	c__Clostridia	o__Lachnospirales	f__Lachnospiraceae	g__Faecalimonas	s__Faecalimonas umbilicata
Con	Con20	bin12	MAG1741	d__Bacteria	p__Bacillota_A	c__Clostridia	o__Lachnospirales	f__Lachnospiraceae	g__Faecalimonas	s__Faecalimonas umbilicata
Con	Con11	bin17	MAG17	d__Bacteria	p__Bacillota_A	c__Clostridia	o__Lachnospirales	f__Lachnospiraceae	g__Faecalimonas	s__Faecalimonas umbilicata
Con	Con32	bin40	MAG1847	d__Bacteria	p__Bacillota_A	c__Clostridia	o__Lachnospirales	f__Lachnospiraceae	g__Lachnospira	s__Lachnospira sp900316325
Con	Con6	bin9	MAG689	d__Bacteria	p__Bacillota_A	c__Clostridia	o__Lachnospirales	f__Lachnospiraceae	g__Agathobacter	s__Agathobacter faecis
Con	Con45	bin41	MAG1582	d__Bacteria	p__Bacillota_A	c__Clostridia	o__Lachnospirales	f__Lachnospiraceae	g__Agathobacter	s__Agathobacter faecis
Con	Con40	bin62	MAG968	d__Bacteria	p__Bacillota_A	c__Clostridia	o__Lachnospirales	f__Lachnospiraceae	g__Agathobacter	s__Agathobacter faecis
Con	Con8	bin25	MAG1136	d__Bacteria	p__Bacillota_A	c__Clostridia	o__Lachnospirales	f__Lachnospiraceae	g__Agathobacter	s__Agathobacter faecis
Con	Con1	bin37	MAG3069	d__Bacteria	p__Bacillota_A	c__Clostridia	o__Lachnospirales	f__Lachnospiraceae	g__Agathobacter	s__Agathobacter faecis
Con	Con38	bin56	MAG2357	d__Bacteria	p__Bacillota_A	c__Clostridia	o__Lachnospirales	f__Lachnospiraceae	g__Agathobacter	s__Agathobacter faecis
Con	Con50	bin13	MAG2856	d__Bacteria	p__Bacillota_A	c__Clostridia	o__Lachnospirales	f__Lachnospiraceae	g__Agathobacter	s__Agathobacter faecis
Con	Con2	bin58	MAG966	d__Bacteria	p__Bacillota_A	c__Clostridia	o__Lachnospirales	f__Lachnospiraceae	g__Agathobacter	s__Agathobacter faecis
Con	Con45	bin17	MAG141	d__Bacteria	p__Bacillota_A	c__Clostridia	o__Lachnospirales	f__Anaerotignaceae	g__Anaerotignum	s__Anaerotignum sp001304995
Con	Con25	bin20	MAG1886	d__Bacteria	p__Bacillota_A	c__Clostridia	o__Lachnospirales	f__Lachnospiraceae	g__Blautia_A	s__Blautia_A sp900549015
Con	Con50	bin45	MAG1708	d__Bacteria	p__Bacillota_A	c__Clostridia	o__Lachnospirales	f__Lachnospiraceae	g__Blautia_A	s__Blautia_A sp900066335
Con	Con12	bin16	MAG2544	d__Bacteria	p__Bacillota_A	c__Clostridia	o__Lachnospirales	f__Lachnospiraceae	g__Blautia_A	s__Blautia_A sp900066335
Con	Con12	bin33	MAG1788	d__Bacteria	p__Bacillota_A	c__Clostridia	o__Lachnospirales	f__Lachnospiraceae	g__Enterocloster	s__Enterocloster sp001517625
Con	Con48	bin2	MAG3159	d__Bacteria	p__Bacillota_A	c__Clostridia	o__Lachnospirales	f__Lachnospiraceae	g__Mediterraneibacter	s__Mediterraneibacter lactaris
Con	Con6	bin5	MAG2312	d__Bacteria	p__Bacillota_A	c__Clostridia	o__Lachnospirales	f__Lachnospiraceae	g__Mediterraneibacter	s__Mediterraneibacter faecis
Con	Con28	bin6	MAG274	d__Bacteria	p__Bacillota_A	c__Clostridia	o__Lachnospirales	f__Lachnospiraceae	g__Mediterraneibacter	s__Mediterraneibacter faecis
Con	Con19	bin20	MAG2992	d__Bacteria	p__Bacillota_A	c__Clostridia	o__Lachnospirales	f__Lachnospiraceae	g__Mediterraneibacter	s__Mediterraneibacter faecis
Con	Con34	bin1	MAG2916	d__Bacteria	p__Bacillota_A	c__Clostridia	o__Lachnospirales	f__Lachnospiraceae	g__Mediterraneibacter	s__Mediterraneibacter faecis
Con	Con44	bin41	MAG2736	d__Bacteria	p__Bacillota_A	c__Clostridia	o__Lachnospirales	f__Lachnospiraceae	g__Mediterraneibacter	s__Mediterraneibacter faecis
Con	Con24	bin12	MAG2884	d__Bacteria	p__Bacillota_A	c__Clostridia	o__Lachnospirales	f__Lachnospiraceae	g__Mediterraneibacter	s__Mediterraneibacter faecis
Con	Con48	bin4	MAG1541	d__Bacteria	p__Bacillota_A	c__Clostridia	o__Lachnospirales	f__Lachnospiraceae	g__Mediterraneibacter	s__Mediterraneibacter faecis
Con	Con40	bin63	MAG2641	d__Bacteria	p__Bacillota_A	c__Clostridia	o__Lachnospirales	f__Lachnospiraceae	g__Agathobacter	s__Agathobacter sp900543445
Con	Con3	bin9	MAG143	d__Bacteria	p__Bacillota_A	c__Clostridia	o__Lachnospirales	f__Lachnospiraceae	g__Agathobacter	s__Agathobacter sp900543445
Con	Con11	bin21	MAG353	d__Bacteria	p__Bacillota_A	c__Clostridia	o__Lachnospirales	f__Anaerotignaceae	g__Anaerotignum	s__Anaerotignum lactatifermentans

Con	Con32	bin11	MAG1199	d__Bacteria	p__Bacillota_A	c__Clostridia	o__Lachnospirales	f__Lachnospiraceae	g__KLE1615	s__KLE1615 sp900066985
Con	Con24	bin2	MAG1467	d__Bacteria	p__Bacillota_A	c__Clostridia	o__Lachnospirales	f__Lachnospiraceae	g__KLE1615	s__KLE1615 sp900066985
Con	Con25	bin13	MAG2549	d__Bacteria	p__Bacillota_A	c__Clostridia	o__Lachnospirales	f__Lachnospiraceae	g__Clostridium_Q	s__Clostridium_Q sp000435655
Con	Con32	bin27	MAG1905	d__Bacteria	p__Bacillota_A	c__Clostridia	o__Lachnospirales	f__Lachnospiraceae	g__Mediterraneibacter	s__Mediterraneibacter torques
Con	Con39	bin14	MAG1160	d__Bacteria	p__Bacillota_A	c__Clostridia	o__Lachnospirales	f__Lachnospiraceae	g__Mediterraneibacter	s__Mediterraneibacter torques
Con	Con44	bin49	MAG1527	d__Bacteria	p__Bacillota_A	c__Clostridia	o__Lachnospirales	f__Lachnospiraceae	g__Mediterraneibacter	s__Mediterraneibacter torques
Con	Con34	bin46	MAG1301	d__Bacteria	p__Bacillota_A	c__Clostridia	o__Lachnospirales	f__Lachnospiraceae	g__Mediterraneibacter	s__Mediterraneibacter torques
Con	Con21	bin37	MAG798	d__Bacteria	p__Bacillota_A	c__Clostridia	o__Lachnospirales	f__Lachnospiraceae	g__Mediterraneibacter	s__Mediterraneibacter torques
Con	Con31	bin35	MAG1097	d__Bacteria	p__Bacillota_A	c__Clostridia	o__Lachnospirales	f__Lachnospiraceae	g__Roseburia	s__Roseburia sp003470905
Con	Con2	bin31	MAG1306	d__Bacteria	p__Bacillota_A	c__Clostridia	o__Lachnospirales	f__Lachnospiraceae	g__UBA11774	s__UBA11774 sp003507655
Con	Con48	bin11	MAG1207	d__Bacteria	p__Bacillota_A	c__Clostridia	o__Lachnospirales	f__Lachnospiraceae	g__Oliverpabstia	s__Oliverpabstia faecicola
Con	Con32	bin37	MAG2919	d__Bacteria	p__Bacillota_A	c__Clostridia	o__Lachnospirales	f__CAG-274	g__CAG-274	s__CAG-274 sp000432155
Con	Con17	bin50	MAG2257	d__Bacteria	p__Bacillota_A	c__Clostridia	o__Lachnospirales	f__Lachnospiraceae	g__Fusicatenibacter	s__Fusicatenibacter saccharivorans
Con	Con32	bin38	MAG2019	d__Bacteria	p__Bacillota_A	c__Clostridia	o__Lachnospirales	f__Lachnospiraceae	g__Fusicatenibacter	s__Fusicatenibacter saccharivorans
Con	Con8	bin15	MAG3287	d__Bacteria	p__Bacillota_A	c__Clostridia	o__Lachnospirales	f__Lachnospiraceae	g__Fusicatenibacter	s__Fusicatenibacter saccharivorans
Con	Con24	bin28	MAG3359	d__Bacteria	p__Bacillota_A	c__Clostridia	o__Lachnospirales	f__Lachnospiraceae	g__Fusicatenibacter	s__Fusicatenibacter saccharivorans
Con	Con10	bin9	MAG2653	d__Bacteria	p__Bacillota_A	c__Clostridia	o__Lachnospirales	f__Lachnospiraceae	g__Fusicatenibacter	s__Fusicatenibacter saccharivorans
Con	Con4	bin27	MAG3014	d__Bacteria	p__Bacillota_A	c__Clostridia	o__Lachnospirales	f__Lachnospiraceae	g__Fusicatenibacter	s__Fusicatenibacter saccharivorans
Con	Con39	bin40	MAG553	d__Bacteria	p__Bacillota_A	c__Clostridia	o__Lachnospirales	f__Lachnospiraceae	g__Fusicatenibacter	s__Fusicatenibacter saccharivorans
Con	Con50	bin46	MAG758	d__Bacteria	p__Bacillota_A	c__Clostridia	o__Lachnospirales	f__Lachnospiraceae	g__Fusicatenibacter	s__Fusicatenibacter saccharivorans
Con	Con48	bin23	MAG2396	d__Bacteria	p__Bacillota_A	c__Clostridia	o__Lachnospirales	f__Lachnospiraceae	g__Fusicatenibacter	s__Fusicatenibacter saccharivorans
Con	Con27	bin21	MAG2036	d__Bacteria	p__Bacillota_A	c__Clostridia	o__Lachnospirales	f__Lachnospiraceae	g__Fusicatenibacter	s__Fusicatenibacter saccharivorans
Con	Con2	bin48	MAG2439	d__Bacteria	p__Bacillota_A	c__Clostridia	o__Lachnospirales	f__Lachnospiraceae	g__Fusicatenibacter	s__Fusicatenibacter saccharivorans
Con	Con49	bin63	MAG2601	d__Bacteria	p__Bacillota_A	c__Clostridia	o__Lachnospirales	f__Lachnospiraceae	g__Fusicatenibacter	s__Fusicatenibacter saccharivorans
Con	Con21	bin34	MAG886	d__Bacteria	p__Bacillota_A	c__Clostridia	o__Lachnospirales	f__Lachnospiraceae	g__Fusicatenibacter	s__Fusicatenibacter saccharivorans
Con	Con40	bin8	MAG64	d__Bacteria	p__Bacillota_A	c__Clostridia	o__Lachnospirales	f__Lachnospiraceae	g__Fusicatenibacter	s__Fusicatenibacter saccharivorans
Con	Con38	bin42	MAG135	d__Bacteria	p__Bacillota_A	c__Clostridia	o__Lachnospirales	f__Lachnospiraceae	g__Fusicatenibacter	s__Fusicatenibacter saccharivorans
Con	Con51	bin43	MAG2643	d__Bacteria	p__Bacillota_A	c__Clostridia	o__Lachnospirales	f__Lachnospiraceae	g__Fusicatenibacter	s__Fusicatenibacter saccharivorans
Con	Con6	bin34	MAG874	d__Bacteria	p__Bacillota_A	c__Clostridia	o__Lachnospirales	f__Lachnospiraceae	g__Fusicatenibacter	s__Fusicatenibacter saccharivorans
Con	Con54	bin26	MAG1953	d__Bacteria	p__Bacillota_A	c__Clostridia	o__Lachnospirales	f__Lachnospiraceae	g__Fusicatenibacter	s__Fusicatenibacter saccharivorans
Con	Con32	bin34	MAG324	d__Bacteria	p__Bacillota_A	c__Clostridia	o__Lachnospirales	f__Lachnospiraceae	g__Anaerostipes	s__Unclassified
Con	Con20	bin3	MAG651	d__Bacteria	p__Bacillota_A	c__Clostridia	o__Lachnospirales	f__Lachnospiraceae	g__Coprococcus_A	s__Coprococcus_A catus_A
Con	Con12	bin45	MAG351	d__Bacteria	p__Bacillota_A	c__Clostridia	o__Lachnospirales	f__Lachnospiraceae	g__Blautia_A	s__Blautia_A sp000436615
Con	Con11	bin2	MAG537	d__Bacteria	p__Bacillota_A	c__Clostridia	o__Lachnospirales	f__Lachnospiraceae	g__Blautia_A	s__Blautia_A sp000436615
Con	Con2	bin21	MAG937	d__Bacteria	p__Bacillota_A	c__Clostridia	o__Lachnospirales	f__Lachnospiraceae	g__Blautia_A	s__Blautia_A sp000436615
Con	Con35	bin47	MAG2244	d__Bacteria	p__Bacillota_A	c__Clostridia	o__Lachnospirales	f__Lachnospiraceae	g__Butyribacter	s__Butyribacter sp003529475
Con	Con8	bin20	MAG1354	d__Bacteria	p__Bacillota_A	c__Clostridia	o__Lachnospirales	f__Lachnospiraceae	g__Enterocloster	s__Enterocloster bolteae
Con	Con24	bin37	MAG3196	d__Bacteria	p__Bacillota_A	c__Clostridia	o__Lachnospirales	f__Lachnospiraceae	g__Enterocloster	s__Enterocloster bolteae
Con	Con53	bin9	MAG2774	d__Bacteria	p__Bacillota_A	c__Clostridia	o__Lachnospirales	f__Lachnospiraceae	g__Enterocloster	s__Enterocloster bolteae
Con	Con22	bin25	MAG2584	d__Bacteria	p__Bacillota_A	c__Clostridia	o__Lachnospirales	f__Lachnospiraceae	g__Sellimonas	s__Sellimonas intestinalis
Con	Con50	bin7	MAG908	d__Bacteria	p__Bacillota_A	c__Clostridia	o__Lachnospirales	f__Lachnospiraceae	g__Eubacterium_G	s__Eubacterium_G sp000435815
Con	Con8	bin39	MAG3172	d__Bacteria	p__Bacillota_A	c__Clostridia	o__Lachnospirales	f__Lachnospiraceae	g__Eubacterium_G	s__Eubacterium_G sp000435815
Con	Con35	bin4	MAG2126	d__Bacteria	p__Bacillota_A	c__Clostridia	o__Lachnospirales	f__Lachnospiraceae	g__Roseburia	s__Roseburia intestinalis
Con	Con22	bin29	MAG1960	d__Bacteria	p__Bacillota_A	c__Clostridia	o__Lachnospirales	f__Lachnospiraceae	g__Roseburia	s__Roseburia intestinalis
Con	Con6	bin20	MAG2782	d__Bacteria	p__Bacillota_A	c__Clostridia	o__Lachnospirales	f__Lachnospiraceae	g__Roseburia	s__Roseburia intestinalis
Con	Con27	bin14	MAG3231	d__Bacteria	p__Bacillota_A	c__Clostridia	o__Lachnospirales	f__Lachnospiraceae	g__Roseburia	s__Roseburia intestinalis
Con	Con39	bin41	MAG53	d__Bacteria	p__Bacillota_A	c__Clostridia	o__Lachnospirales	f__Lachnospiraceae	g__Roseburia	s__Roseburia intestinalis
Con	Con40	bin54	MAG2364	d__Bacteria	p__Bacillota_A	c__Clostridia	o__Lachnospirales	f__Lachnospiraceae	g__Roseburia	s__Roseburia intestinalis
Con	Con40	bin32	MAG98	d__Bacteria	p__Bacillota_A	c__Clostridia	o__Lachnospirales	f__Lachnospiraceae	g__Roseburia	s__Roseburia intestinalis
Con	Con12	bin26	MAG2169	d__Bacteria	p__Bacillota_A	c__Clostridia	o__Lachnospirales	f__Lachnospiraceae	g__CHH4-2	s__CHH4-2 sp002899675
Con	Con11	bin3	MAG1839	d__Bacteria	p__Bacillota_A	c__Clostridia	o__Lachnospirales	f__Lachnospiraceae	g__Blautia_A	s__Blautia_A wexlerae

Con	Con26	bin17	MAG2695	d__Bacteria	p__Bacillota_A	c__Clostridia	o__Lachnospirales	f__Lachnospiraceae	g__Blautia_A	s__Blautia_A wexlerae
Con	Con28	bin7	MAG282	d__Bacteria	p__Bacillota_A	c__Clostridia	o__Lachnospirales	f__Lachnospiraceae	g__Blautia_A	s__Blautia_A wexlerae
Con	Con35	bin46	MAG2290	d__Bacteria	p__Bacillota_A	c__Clostridia	o__Lachnospirales	f__Lachnospiraceae	g__Blautia_A	s__Blautia_A wexlerae
Con	Con50	bin21	MAG2427	d__Bacteria	p__Bacillota_A	c__Clostridia	o__Lachnospirales	f__Lachnospiraceae	g__Blautia_A	s__Blautia_A wexlerae
Con	Con30	bin50	MAG1128	d__Bacteria	p__Bacillota_A	c__Clostridia	o__Lachnospirales	f__Lachnospiraceae	g__Blautia_A	s__Blautia_A wexlerae
Con	Con27	bin6	MAG2039	d__Bacteria	p__Bacillota_A	c__Clostridia	o__Lachnospirales	f__Lachnospiraceae	g__Blautia_A	s__Blautia_A wexlerae
Con	Con45	bin49	MAG1148	d__Bacteria	p__Bacillota_A	c__Clostridia	o__Lachnospirales	f__Lachnospiraceae	g__Blautia_A	s__Blautia_A wexlerae
Con	Con32	bin43	MAG1853	d__Bacteria	p__Bacillota_A	c__Clostridia	o__Lachnospirales	f__Lachnospiraceae	g__Blautia_A	s__Blautia_A wexlerae
Con	Con12	bin18	MAG2289	d__Bacteria	p__Bacillota_A	c__Clostridia	o__Lachnospirales	f__Lachnospiraceae	g__Blautia_A	s__Blautia_A wexlerae
Con	Con44	bin46	MAG2806	d__Bacteria	p__Bacillota_A	c__Clostridia	o__Lachnospirales	f__Lachnospiraceae	g__Blautia_A	s__Blautia_A wexlerae
Con	Con17	bin37	MAG614	d__Bacteria	p__Bacillota_A	c__Clostridia	o__Lachnospirales	f__Lachnospiraceae	g__Blautia_A	s__Blautia_A wexlerae
Con	Con22	bin12	MAG211	d__Bacteria	p__Bacillota_A	c__Clostridia	o__Lachnospirales	f__Lachnospiraceae	g__Blautia	s__Blautia sp900541955
Con	Con24	bin54	MAG506	d__Bacteria	p__Bacillota_A	c__Clostridia	o__Lachnospirales	f__Lachnospiraceae	g__Dorea_A	s__Dorea_A longicatena_B
Con	Con39	bin9	MAG857	d__Bacteria	p__Bacillota_A	c__Clostridia	o__Lachnospirales	f__Lachnospiraceae	g__Dorea_A	s__Dorea_A longicatena_B
Con	Con27	bin36	MAG3506	d__Bacteria	p__Bacillota_A	c__Clostridia	o__Lachnospirales	f__Lachnospiraceae	g__Dorea_A	s__Dorea_A longicatena_B
Con	Con25	bin29	MAG2265	d__Bacteria	p__Bacillota_A	c__Clostridia	o__Lachnospirales	f__Lachnospiraceae	g__Dorea_A	s__Dorea_A longicatena_B
Con	Con4	bin55	MAG514	d__Bacteria	p__Bacillota_A	c__Clostridia	o__Lachnospirales	f__Lachnospiraceae	g__Dorea_A	s__Dorea_A longicatena_B
Con	Con30	bin48	MAG811	d__Bacteria	p__Bacillota_A	c__Clostridia	o__Lachnospirales	f__Lachnospiraceae	g__Enterocloster	s__Enterocloster aldenensis
Con	Con1	bin45	MAG2483	d__Bacteria	p__Bacillota_A	c__Clostridia	o__Lachnospirales	f__Lachnospiraceae	g__HGM12545	s__HGM12545 sp900761925
Con	Con32	bin8	MAG1744	d__Bacteria	p__Bacillota_A	c__Clostridia	o__Lachnospirales	f__Lachnospiraceae	g__Enterocloster	s__Enterocloster sp000431375
Con	Con22	bin23	MAG2769	d__Bacteria	p__Bacillota_A	c__Clostridia	o__Lachnospirales	f__Lachnospiraceae	g__Enterocloster	s__Enterocloster sp000431375
Con	Con1	bin67	MAG1987	d__Bacteria	p__Bacillota_A	c__Clostridia	o__Lachnospirales	f__Lachnospiraceae	g__COE1	s__COE1 sp001916965
Con	Con32	bin29	MAG1779	d__Bacteria	p__Bacillota_A	c__Clostridia	o__Lachnospirales	f__Lachnospiraceae	g__Enterocloster	s__Enterocloster clostridioformis
Con	Con24	bin55	MAG2331	d__Bacteria	p__Bacillota_A	c__Clostridia	o__Lachnospirales	f__Lachnospiraceae	g__Lachnospira	s__Lachnospira eligens_A
Con	Con20	bin5	MAG2007	d__Bacteria	p__Bacillota_A	c__Clostridia	o__Lachnospirales	f__Lachnospiraceae	g__Lachnospira	s__Lachnospira eligens_A
Con	Con4	bin57	MAG542	d__Bacteria	p__Bacillota_A	c__Clostridia	o__Lachnospirales	f__Lachnospiraceae	g__Lachnospira	s__Lachnospira eligens_A
Con	Con31	bin23	MAG1468	d__Bacteria	p__Bacillota_A	c__Clostridia	o__Lachnospirales	f__Lachnospiraceae	g__Lachnospira	s__Lachnospira eligens_A
Con	Con17	bin60	MAG1147	d__Bacteria	p__Bacillota_A	c__Clostridia	o__Lachnospirales	f__Lachnospiraceae	g__Lachnospira	s__Lachnospira eligens_A
Con	Con35	bin9	MAG1814	d__Bacteria	p__Bacillota_A	c__Clostridia	o__Lachnospirales	f__Lachnospiraceae	g__Wujia	s__Wujia chipingensis
Con	Con45	bin18	MAG2199	d__Bacteria	p__Bacillota_A	c__Clostridia	o__Lachnospirales	f__Lachnospiraceae	g__Wujia	s__Wujia chipingensis
Con	Con3	bin12	MAG1717	d__Bacteria	p__Bacillota_A	c__Clostridia	o__Lachnospirales	f__Lachnospiraceae	g__Wujia	s__Wujia chipingensis
Con	Con18	bin9	MAG315	d__Bacteria	p__Bacillota_A	c__Clostridia	o__Lachnospirales	f__Lachnospiraceae	g__Scybalocola	s__Scybalocola sp900557175
Con	Con38	bin54	MAG809	d__Bacteria	p__Bacillota_A	c__Clostridia	o__Lachnospirales	f__Lachnospiraceae	g__Eubacterium_G	s__Eubacterium_G ventriosum
Con	Con4	bin22	MAG2261	d__Bacteria	p__Bacillota_A	c__Clostridia	o__Lachnospirales	f__Lachnospiraceae	g__Eubacterium_G	s__Eubacterium_G ventriosum
Con	Con40	bin14	MAG1902	d__Bacteria	p__Bacillota_A	c__Clostridia	o__Lachnospirales	f__Lachnospiraceae	g__Eubacterium_G	s__Eubacterium_G ventriosum
Con	Con8	bin12	MAG3324	d__Bacteria	p__Bacillota_A	c__Clostridia	o__Lachnospirales	f__Lachnospiraceae	g__Eubacterium_G	s__Eubacterium_G ventriosum
Con	Con6	bin38	MAG1159	d__Bacteria	p__Bacillota_A	c__Clostridia	o__Lachnospirales	f__Lachnospiraceae	g__Eubacterium_G	s__Eubacterium_G ventriosum
Con	Con22	bin6	MAG520	d__Bacteria	p__Bacillota_A	c__Clostridia	o__Lachnospirales	f__Lachnospiraceae	g__Eubacterium_G	s__Eubacterium_G ventriosum
Con	Con51	bin5	MAG2624	d__Bacteria	p__Bacillota_A	c__Clostridia	o__Lachnospirales	f__Lachnospiraceae	g__Roseburia	s__Roseburia inulinivorans
Con	Con35	bin12	MAG1369	d__Bacteria	p__Bacillota_A	c__Clostridia	o__Lachnospirales	f__Lachnospiraceae	g__Roseburia	s__Roseburia inulinivorans
Con	Con45	bin27	MAG119	d__Bacteria	p__Bacillota_A	c__Clostridia	o__Lachnospirales	f__Lachnospiraceae	g__Roseburia	s__Roseburia inulinivorans
Con	Con52	bin50	MAG2177	d__Bacteria	p__Bacillota_A	c__Clostridia	o__Lachnospirales	f__Lachnospiraceae	g__Roseburia	s__Roseburia inulinivorans
Con	Con1	bin63	MAG2837	d__Bacteria	p__Bacillota_A	c__Clostridia	o__Lachnospirales	f__Lachnospiraceae	g__Roseburia	s__Roseburia inulinivorans
Con	Con21	bin5	MAG1364	d__Bacteria	p__Bacillota_A	c__Clostridia	o__Lachnospirales	f__Lachnospiraceae	g__Roseburia	s__Roseburia inulinivorans
Con	Con17	bin10	MAG3482	d__Bacteria	p__Bacillota_A	c__Clostridia	o__Lachnospirales	f__Lachnospiraceae	g__Blautia_A	s__Blautia_A hydrogenotrophica
Con	Con32	bin4	MAG1608	d__Bacteria	p__Bacillota_A	c__Clostridia	o__Lachnospirales	f__Lachnospiraceae	g__Blautia	s__Blautia stercoris
Con	Con6	bin14	MAG3237	d__Bacteria	p__Bacillota_A	c__Clostridia	o__Lachnospirales	f__Lachnospiraceae	g__Bariatricus	s__Bariatricus comes
Con	Con31	bin15	MAG203	d__Bacteria	p__Bacillota_A	c__Clostridia	o__Lachnospirales	f__Lachnospiraceae	g__Bariatricus	s__Bariatricus comes
Con	Con18	bin7	MAG3192	d__Bacteria	p__Bacillota_A	c__Clostridia	o__Lachnospirales	f__Lachnospiraceae	g__Bariatricus	s__Bariatricus comes
Con	Con4	bin6	MAG1484	d__Bacteria	p__Bacillota_A	c__Clostridia	o__Lachnospirales	f__Lachnospiraceae	g__Bariatricus	s__Bariatricus comes
Con	Con3	bin50	MAG2631	d__Bacteria	p__Bacillota_A	c__Clostridia	o__Lachnospirales	f__Lachnospiraceae	g__Bariatricus	s__Bariatricus comes

Con	Con40	bin46	MAG1183	d__Bacteria	p__Bacillota_A	c__Clostridia	o__Lachnospirales	f__Lachnospiraceae	g__Bariatricus	s__Bariatricus comes
Con	Con44	bin4	MAG787	d__Bacteria	p__Bacillota_A	c__Clostridia	o__Lachnospirales	f__Lachnospiraceae	g__Bariatricus	s__Bariatricus comes
Con	Con48	bin17	MAG632	d__Bacteria	p__Bacillota_A	c__Clostridia	o__Lachnospirales	f__Lachnospiraceae	g__Bariatricus	s__Bariatricus comes
Con	Con48	bin8	MAG666	d__Bacteria	p__Bacillota_A	c__Clostridia	o__Lachnospirales	f__Lachnospiraceae	g__Eubacterium_I	s__Eubacterium_I ramulus
Con	Con17	bin28	MAG3443	d__Bacteria	p__Bacillota_A	c__Clostridia	o__Monoglobales	f__Monoglobaceae	g__Monoglobus	s__Monoglobus pectinilyticus
Con	Con49	bin5	MAG3090	d__Bacteria	p__Bacillota_A	c__Clostridia	o__Monoglobales	f__Monoglobaceae	g__UMGS1820	s__UMGS1820 sp900545865
Con	Con22	bin17	MAG640	d__Bacteria	p__Bacillota_A	c__Clostridia	o__Oscillospirales	f__Ruminococcaceae	g__Faecalibacterium	s__Faecalibacterium longum
Con	Con31	bin17	MAG1010	d__Bacteria	p__Bacillota_A	c__Clostridia	o__Oscillospirales	f__Ruminococcaceae	g__Faecalibacterium	s__Faecalibacterium longum
Con	Con32	bin2	MAG2921	d__Bacteria	p__Bacillota_A	c__Clostridia	o__Oscillospirales	f__Ruminococcaceae	g__Faecalibacterium	s__Faecalibacterium longum
Con	Con5	bin51	MAG3004	d__Bacteria	p__Bacillota_A	c__Clostridia	o__Oscillospirales	f__Ruminococcaceae	g__Faecalibacterium	s__Faecalibacterium longum
Con	Con45	bin42	MAG1743	d__Bacteria	p__Bacillota_A	c__Clostridia	o__Oscillospirales	f__Ruminococcaceae	g__Faecalibacterium	s__Faecalibacterium longum
Con	Con24	bin40	MAG2723	d__Bacteria	p__Bacillota_A	c__Clostridia	o__Oscillospirales	f__Ruminococcaceae	g__Faecalibacterium	s__Faecalibacterium longum
Con	Con4	bin37	MAG551	d__Bacteria	p__Bacillota_A	c__Clostridia	o__Oscillospirales	f__Ruminococcaceae	g__Faecalibacterium	s__Faecalibacterium longum
Con	Con27	bin27	MAG1982	d__Bacteria	p__Bacillota_A	c__Clostridia	o__Oscillospirales	f__Ruminococcaceae	g__Faecalibacterium	s__Faecalibacterium longum
Con	Con35	bin18	MAG3204	d__Bacteria	p__Bacillota_A	c__Clostridia	o__Oscillospirales	f__Ruminococcaceae	g__Faecalibacterium	s__Faecalibacterium longum
Con	Con6	bin37	MAG1721	d__Bacteria	p__Bacillota_A	c__Clostridia	o__Oscillospirales	f__Ruminococcaceae	g__Faecalibacterium	s__Faecalibacterium longum
Con	Con53	bin18	MAG118	d__Bacteria	p__Bacillota_A	c__Clostridia	o__Oscillospirales	f__Ruminococcaceae	g__Faecalibacterium	s__Faecalibacterium longum
Con	Con48	bin38	MAG2776	d__Bacteria	p__Bacillota_A	c__Clostridia	o__Oscillospirales	f__Ruminococcaceae	g__Faecalibacterium	s__Faecalibacterium duncaniae
Con	Con39	bin51	MAG1308	d__Bacteria	p__Bacillota_A	c__Clostridia	o__Oscillospirales	f__Ruminococcaceae	g__Faecalibacterium	s__Faecalibacterium duncaniae
Con	Con17	bin22	MAG475	d__Bacteria	p__Bacillota_A	c__Clostridia	o__Oscillospirales	f__Ruminococcaceae	g__Faecalibacterium	s__Faecalibacterium duncaniae
Con	Con6	bin39	MAG3424	d__Bacteria	p__Bacillota_A	c__Clostridia	o__Oscillospirales	f__Ruminococcaceae	g__Faecalibacterium	s__Faecalibacterium duncaniae
Con	Con38	bin26	MAG1863	d__Bacteria	p__Bacillota_A	c__Clostridia	o__Oscillospirales	f__Ruminococcaceae	g__Faecalibacterium	s__Faecalibacterium duncaniae
Con	Con5	bin29	MAG3152	d__Bacteria	p__Bacillota_A	c__Clostridia	o__Oscillospirales	f__Ruminococcaceae	g__Faecalibacterium	s__Faecalibacterium duncaniae
Con	Con27	bin43	MAG3085	d__Bacteria	p__Bacillota_A	c__Clostridia	o__Oscillospirales	f__Ruminococcaceae	g__Faecalibacterium	s__Faecalibacterium duncaniae
Con	Con2	bin11	MAG2411	d__Bacteria	p__Bacillota_A	c__Clostridia	o__Oscillospirales	f__Ruminococcaceae	g__Faecalibacterium	s__Faecalibacterium duncaniae
Con	Con40	bin4	MAG1213	d__Bacteria	p__Bacillota_A	c__Clostridia	o__Oscillospirales	f__Ruminococcaceae	g__Faecalibacterium	s__Faecalibacterium duncaniae
Con	Con52	bin25	MAG197	d__Bacteria	p__Bacillota_A	c__Clostridia	o__Oscillospirales	f__Ruminococcaceae	g__Faecalibacterium	s__Faecalibacterium duncaniae
Con	Con32	bin28	MAG1790	d__Bacteria	p__Bacillota_A	c__Clostridia	o__Oscillospirales	f__Acutalibacteraceae	g__UMGS1071	s__UMGS1071 sp900542375
Con	Con20	bin37	MAG837	d__Bacteria	p__Bacillota_A	c__Clostridia	o__Oscillospirales	f__Acutalibacteraceae	g__UMGS1071	s__UMGS1071 sp900542375
Con	Con53	bin26	MAG422	d__Bacteria	p__Bacillota_A	c__Clostridia	o__Oscillospirales	f__Acutalibacteraceae	g__UMGS1071	s__UMGS1071 sp900542375
Con	Con25	bin37	MAG987	d__Bacteria	p__Bacillota_A	c__Clostridia	o__Oscillospirales	f__Ruminococcaceae	g__Ruthenibacterium	s__Ruthenibacterium lactatiformans
Con	Con18	bin3	MAG3214	d__Bacteria	p__Bacillota_A	c__Clostridia	o__Oscillospirales	f__Ruminococcaceae	g__Ruthenibacterium	s__Ruthenibacterium lactatiformans
Con	Con34	bin48	MAG2629	d__Bacteria	p__Bacillota_A	c__Clostridia	o__Oscillospirales	f__Ruminococcaceae	g__Ruthenibacterium	s__Ruthenibacterium lactatiformans
Con	Con26	bin20	MAG1063	d__Bacteria	p__Bacillota_A	c__Clostridia	o__Oscillospirales	f__Ruminococcaceae	g__Ruthenibacterium	s__Ruthenibacterium lactatiformans
Con	Con31	bin3	MAG720	d__Bacteria	p__Bacillota_A	c__Clostridia	o__Oscillospirales	f__Ruminococcaceae	g__Ruthenibacterium	s__Ruthenibacterium lactatiformans
Con	Con45	bin28	MAG2318	d__Bacteria	p__Bacillota_A	c__Clostridia	o__Oscillospirales	f__Ruminococcaceae	g__Ruthenibacterium	s__Ruthenibacterium lactatiformans
Con	Con30	bin43	MAG1351	d__Bacteria	p__Bacillota_A	c__Clostridia	o__Oscillospirales	f__Ruminococcaceae	g__Ruthenibacterium	s__Ruthenibacterium lactatiformans
Con	Con1	bin21	MAG3029	d__Bacteria	p__Bacillota_A	c__Clostridia	o__Oscillospirales	f__Ruminococcaceae	g__Ruthenibacterium	s__Ruthenibacterium lactatiformans
Con	Con12	bin10	MAG2964	d__Bacteria	p__Bacillota_A	c__Clostridia	o__Oscillospirales	f__Ruminococcaceae	g__Ruthenibacterium	s__Ruthenibacterium lactatiformans
Con	Con10	bin38	MAG1525	d__Bacteria	p__Bacillota_A	c__Clostridia	o__Oscillospirales	f__Ruminococcaceae	g__Ruthenibacterium	s__Ruthenibacterium lactatiformans
Con	Con40	bin42	MAG2671	d__Bacteria	p__Bacillota_A	c__Clostridia	o__Oscillospirales	f__Ruminococcaceae	g__Faecalibacterium	s__Faecalibacterium prausnitzii
Con	Con51	bin60	MAG920	d__Bacteria	p__Bacillota_A	c__Clostridia	o__Oscillospirales	f__Ruminococcaceae	g__Faecalibacterium	s__Faecalibacterium prausnitzii
Con	Con6	bin28	MAG445	d__Bacteria	p__Bacillota_A	c__Clostridia	o__Oscillospirales	f__Ruminococcaceae	g__Faecalibacterium	s__Faecalibacterium prausnitzii
Con	Con48	bin31	MAG3392	d__Bacteria	p__Bacillota_A	c__Clostridia	o__Oscillospirales	f__Ruminococcaceae	g__Faecalibacterium	s__Faecalibacterium prausnitzii
Con	Con49	bin60	MAG1693	d__Bacteria	p__Bacillota_A	c__Clostridia	o__Oscillospirales	f__Ruminococcaceae	g__Faecalibacterium	s__Faecalibacterium prausnitzii
Con	Con51	bin57	MAG73	d__Bacteria	p__Bacillota_A	c__Clostridia	o__Oscillospirales	f__Acutalibacteraceae	g__Fimivicinus	s__Fimivicinus sp900544375
Con	Con17	bin32	MAG569	d__Bacteria	p__Bacillota_A	c__Clostridia	o__Oscillospirales	f__Acutalibacteraceae	g__Fimenecus	s__Fimenecus sp000432435
Con	Con52	bin32	MAG2545	d__Bacteria	p__Bacillota_A	c__Clostridia	o__Oscillospirales	f__Acutalibacteraceae	g__Fimenecus	s__Fimenecus sp000432435
Con	Con34	bin32	MAG1058	d__Bacteria	p__Bacillota_A	c__Clostridia	o__Oscillospirales	f__Acutalibacteraceae	g__Fimenecus	s__Fimenecus sp000432435
Con	Con49	bin29	MAG657	d__Bacteria	p__Bacillota_A	c__Clostridia	o__Oscillospirales	f__Acutalibacteraceae	g__Fimenecus	s__Fimenecus sp000432435
Con	Con25	bin1	MAG990	d__Bacteria	p__Bacillota_A	c__Clostridia	o__Oscillospirales	f__Oscillospiraceae	g__Evtepia	s__Evtepia gabavorous

Con	Con19	bin26	MAG3440	d__Bacteria	p__Bacillota_A	c__Clostridia	o__Oscillospirales	f__Oscillospiraceae	g__Evtepia	s__Evtepia gabavorous
Con	Con4	bin33	MAG917	d__Bacteria	p__Bacillota_A	c__Clostridia	o__Oscillospirales	f__Oscillospiraceae	g__Evtepia	s__Evtepia gabavorous
Con	Con12	bin24	MAG1002	d__Bacteria	p__Bacillota_A	c__Clostridia	o__Oscillospirales	f__Oscillospiraceae	g__Evtepia	s__Evtepia gabavorous
Con	Con26	bin9	MAG1171	d__Bacteria	p__Bacillota_A	c__Clostridia	o__Oscillospirales	f__Oscillospiraceae	g__Evtepia	s__Evtepia gabavorous
Con	Con34	bin20	MAG163	d__Bacteria	p__Bacillota_A	c__Clostridia	o__Oscillospirales	f__Ruminococcaceae	g__Ruminococcus_D	s__Ruminococcus_D bicirculans
Con	Con1	bin64	MAG1520	d__Bacteria	p__Bacillota_A	c__Clostridia	o__Oscillospirales	f__Ruminococcaceae	g__Ruminococcus_D	s__Ruminococcus_D bicirculans
Con	Con5	bin43	MAG2266	d__Bacteria	p__Bacillota_A	c__Clostridia	o__Oscillospirales	f__Ruminococcaceae	g__Ruminococcus_D	s__Ruminococcus_D bicirculans
Con	Con39	bin10	MAG1701	d__Bacteria	p__Bacillota_A	c__Clostridia	o__Oscillospirales	f__Ruminococcaceae	g__Ruminococcus_D	s__Ruminococcus_D bicirculans
Con	Con35	bin15	MAG2622	d__Bacteria	p__Bacillota_A	c__Clostridia	o__Oscillospirales	f__Ruminococcaceae	g__Ruminococcus_D	s__Ruminococcus_D bicirculans
Con	Con4	bin15	MAG2121	d__Bacteria	p__Bacillota_A	c__Clostridia	o__Oscillospirales	f__Ruminococcaceae	g__Ruminococcus_D	s__Ruminococcus_D bicirculans
Con	Con1	bin29	MAG3246	d__Bacteria	p__Bacillota_A	c__Clostridia	o__Oscillospirales	f__Acutalibacteraceae	g__Ruminococcus_E	s__Ruminococcus_E sp003438075
Con	Con1	bin47	MAG817	d__Bacteria	p__Bacillota_A	c__Clostridia	o__Oscillospirales	f__Acutalibacteraceae	g__Eubacterium_R	s__Eubacterium_R sp000436835
Con	Con38	bin18	MAG221	d__Bacteria	p__Bacillota_A	c__Clostridia	o__Oscillospirales	f__Acutalibacteraceae	g__Eubacterium_R	s__Eubacterium_R sp000436835
Con	Con26	bin13	MAG2023	d__Bacteria	p__Bacillota_A	c__Clostridia	o__Oscillospirales	f__Acutalibacteraceae	g__Eubacterium_R	s__Eubacterium_R sp000436835
Con	Con2	bin37	MAG2690	d__Bacteria	p__Bacillota_A	c__Clostridia	o__Oscillospirales	f__Acutalibacteraceae	g__Eubacterium_R	s__Eubacterium_R sp000436835
Con	Con50	bin8	MAG989	d__Bacteria	p__Bacillota_A	c__Clostridia	o__Oscillospirales	f__Acutalibacteraceae	g__Eubacterium_R	s__Eubacterium_R sp000436835
Con	Con10	bin34	MAG440	d__Bacteria	p__Bacillota_A	c__Clostridia	o__Oscillospirales	f__Acutalibacteraceae	g__Eubacterium_R	s__Eubacterium_R sp000434995
Con	Con49	bin70	MAG653	d__Bacteria	p__Bacillota_A	c__Clostridia	o__Oscillospirales	f__Ruminococcaceae	g__Angelakisella	s__Angelakisella sp004557855
Con	Con24	bin7	MAG2300	d__Bacteria	p__Bacillota_A	c__Clostridia	o__Oscillospirales	f__Butyricicoccaceae	g__Agathobaculum	s__Agathobaculum butyriciproducens
Con	Con40	bin34	MAG170	d__Bacteria	p__Bacillota_A	c__Clostridia	o__Oscillospirales	f__Butyricicoccaceae	g__Agathobaculum	s__Agathobaculum butyriciproducens
Con	Con44	bin45	MAG1754	d__Bacteria	p__Bacillota_A	c__Clostridia	o__Oscillospirales	f__Butyricicoccaceae	g__Agathobaculum	s__Agathobaculum butyriciproducens
Con	Con50	bin41	MAG2110	d__Bacteria	p__Bacillota_A	c__Clostridia	o__Oscillospirales	f__Butyricicoccaceae	g__Agathobaculum	s__Agathobaculum butyriciproducens
Con	Con30	bin45	MAG396	d__Bacteria	p__Bacillota_A	c__Clostridia	o__Oscillospirales	f__Butyricicoccaceae	g__Agathobaculum	s__Agathobaculum butyriciproducens
Con	Con35	bin5	MAG2279	d__Bacteria	p__Bacillota_A	c__Clostridia	o__Oscillospirales	f__Butyricicoccaceae	g__Agathobaculum	s__Agathobaculum butyriciproducens
Con	Con34	bin36	MAG1107	d__Bacteria	p__Bacillota_A	c__Clostridia	o__Oscillospirales	f__Butyricicoccaceae	g__Agathobaculum	s__Agathobaculum butyriciproducens
Con	Con18	bin21	MAG2981	d__Bacteria	p__Bacillota_A	c__Clostridia	o__Oscillospirales	f__Butyricicoccaceae	g__Agathobaculum	s__Agathobaculum butyriciproducens
Con	Con27	bin18	MAG1766	d__Bacteria	p__Bacillota_A	c__Clostridia	o__Oscillospirales	f__Butyricicoccaceae	g__Agathobaculum	s__Agathobaculum butyriciproducens
Con	Con54	bin20	MAG2548	d__Bacteria	p__Bacillota_A	c__Clostridia	o__Oscillospirales	f__Butyricicoccaceae	g__Agathobaculum	s__Agathobaculum butyriciproducens
Con	Con38	bin39	MAG2106	d__Bacteria	p__Bacillota_A	c__Clostridia	o__Oscillospirales	f__Butyricicoccaceae	g__Agathobaculum	s__Agathobaculum butyriciproducens
Con	Con39	bin7	MAG1181	d__Bacteria	p__Bacillota_A	c__Clostridia	o__Oscillospirales	f__Butyricicoccaceae	g__Agathobaculum	s__Agathobaculum butyriciproducens
Con	Con52	bin40	MAG3012	d__Bacteria	p__Bacillota_A	c__Clostridia	o__Oscillospirales	f__Butyricicoccaceae	g__Agathobaculum	s__Agathobaculum butyriciproducens
Con	Con21	bin40	MAG2482	d__Bacteria	p__Bacillota_A	c__Clostridia	o__Oscillospirales	f__Butyricicoccaceae	g__Agathobaculum	s__Agathobaculum butyriciproducens
Con	Con48	bin1	MAG2247	d__Bacteria	p__Bacillota_A	c__Clostridia	o__Oscillospirales	f__Butyricicoccaceae	g__Agathobaculum	s__Agathobaculum butyriciproducens
Con	Con6	bin24	MAG320	d__Bacteria	p__Bacillota_A	c__Clostridia	o__Oscillospirales	f__Butyricicoccaceae	g__Agathobaculum	s__Agathobaculum butyriciproducens
Con	Con1	bin9	MAG3420	d__Bacteria	p__Bacillota_A	c__Clostridia	o__Oscillospirales	f__Ruminococcaceae	g__Ruthenibacterium	s__Ruthenibacterium merdipullorum
Con	Con34	bin17	MAG1969	d__Bacteria	p__Bacillota_A	c__Clostridia	o__Oscillospirales	f__Acutalibacteraceae	g__Ruminococcus_E	s__Ruminococcus_E sp003526955
Con	Con40	bin20	MAG311	d__Bacteria	p__Bacillota_A	c__Clostridia	o__Oscillospirales	f__Acutalibacteraceae	g__Ruminococcus_E	s__Ruminococcus_E sp003526955
Con	Con50	bin42	MAG745	d__Bacteria	p__Bacillota_A	c__Clostridia	o__Oscillospirales	f__Acutalibacteraceae	g__Ruminococcus_E	s__Ruminococcus_E sp003526955
Con	Con32	bin45	MAG2918	d__Bacteria	p__Bacillota_A	c__Clostridia	o__Oscillospirales	f__Ruminococcaceae	g__Faecalibacterium	s__Faecalibacterium prausnitzii_D
Con	Con39	bin44	MAG1740	d__Bacteria	p__Bacillota_A	c__Clostridia	o__Oscillospirales	f__Ruminococcaceae	g__Faecalibacterium	s__Faecalibacterium prausnitzii_D
Con	Con28	bin12	MAG590	d__Bacteria	p__Bacillota_A	c__Clostridia	o__Oscillospirales	f__Ruminococcaceae	g__Gemmiger	s__Gemmiger qucibialis
Con	Con39	bin11	MAG2697	d__Bacteria	p__Bacillota_A	c__Clostridia	o__Oscillospirales	f__Ruminococcaceae	g__Gemmiger	s__Gemmiger qucibialis
Con	Con40	bin18	MAG2454	d__Bacteria	p__Bacillota_A	c__Clostridia	o__Oscillospirales	f__Ruminococcaceae	g__Gemmiger	s__Gemmiger qucibialis
Con	Con52	bin21	MAG152	d__Bacteria	p__Bacillota_A	c__Clostridia	o__Oscillospirales	f__Ruminococcaceae	g__Gemmiger	s__Gemmiger qucibialis
Con	Con6	bin35	MAG2397	d__Bacteria	p__Bacillota_A	c__Clostridia	o__Oscillospirales	f__Ruminococcaceae	g__Gemmiger	s__Gemmiger qucibialis
Con	Con17	bin45	MAG2701	d__Bacteria	p__Bacillota_A	c__Clostridia	o__Oscillospirales	f__Ruminococcaceae	g__Gemmiger	s__Gemmiger qucibialis
Con	Con35	bin36	MAG711	d__Bacteria	p__Bacillota_A	c__Clostridia	o__Oscillospirales	f__Ruminococcaceae	g__Gemmiger	s__Gemmiger qucibialis
Con	Con4	bin58	MAG3258	d__Bacteria	p__Bacillota_A	c__Clostridia	o__Oscillospirales	f__Ruminococcaceae	g__Gemmiger	s__Gemmiger qucibialis
Con	Con9	bin9	MAG1807	d__Bacteria	p__Bacillota_A	c__Clostridia	o__Oscillospirales	f__Ruminococcaceae	g__Gemmiger	s__Gemmiger qucibialis
Con	Con49	bin87	MAG344	d__Bacteria	p__Bacillota_A	c__Clostridia	o__Oscillospirales	f__Ruminococcaceae	g__Gemmiger	s__Gemmiger qucibialis
Con	Con51	bin41	MAG3409	d__Bacteria	p__Bacillota_A	c__Clostridia	o__Oscillospirales	f__Ruminococcaceae	g__Gemmiger	s__Gemmiger qucibialis

Con	Con27	bin46	MAG1556	d__Bacteria	p__Bacillota_A	c__Clostridia	o__Oscillospirales	f__Oscillospiraceae	g__ER4	s__ER4 sp000765235
Con	Con21	bin41	MAG2361	d__Bacteria	p__Bacillota_A	c__Clostridia	o__Oscillospirales	f__Oscillospiraceae	g__ER4	s__ER4 sp000765235
Con	Con34	bin51	MAG161	d__Bacteria	p__Bacillota_A	c__Clostridia	o__Oscillospirales	f__Oscillospiraceae	g__ER4	s__ER4 sp000765235
Con	Con4	bin44	MAG2447	d__Bacteria	p__Bacillota_A	c__Clostridia	o__Oscillospirales	f__Oscillospiraceae	g__ER4	s__ER4 sp000765235
Con	Con39	bin21	MAG2232	d__Bacteria	p__Bacillota_A	c__Clostridia	o__Oscillospirales	f__Oscillospiraceae	g__ER4	s__ER4 sp000765235
Con	Con5	bin38	MAG461	d__Bacteria	p__Bacillota_A	c__Clostridia	o__Oscillospirales	f__Oscillospiraceae	g__ER4	s__ER4 sp000765235
Con	Con3	bin41	MAG105	d__Bacteria	p__Bacillota_A	c__Clostridia	o__Oscillospirales	f__Oscillospiraceae	g__Dysosmobacter	s__Dysosmobacter sp001916835
Con	Con49	bin47	MAG1965	d__Bacteria	p__Bacillota_A	c__Clostridia	o__Oscillospirales	f__Oscillospiraceae	g__Dysosmobacter	s__Dysosmobacter sp001916835
Con	Con19	bin55	MAG2436	d__Bacteria	p__Bacillota_A	c__Clostridia	o__Oscillospirales	f__Oscillospiraceae	g__Dysosmobacter	s__Dysosmobacter sp001916835
Con	Con2	bin7	MAG314	d__Bacteria	p__Bacillota_A	c__Clostridia	o__Oscillospirales	f__Ruminococcaceae	g__Gemmiger	s__Gemmiger sp900540595
Con	Con8	bin29	MAG822	d__Bacteria	p__Bacillota_A	c__Clostridia	o__Oscillospirales	f__Ruminococcaceae	g__Gemmiger	s__Gemmiger sp900540595
Con	Con17	bin67	MAG1498	d__Bacteria	p__Bacillota_A	c__Clostridia	o__Oscillospirales	f__Acutalibacteraceae	g__CAG-217	s__CAG-217 sp000436335
Con	Con21	bin39	MAG1465	d__Bacteria	p__Bacillota_A	c__Clostridia	o__Oscillospirales	f__Acutalibacteraceae	g__CAG-217	s__CAG-217 sp000436335
Con	Con27	bin48	MAG3167	d__Bacteria	p__Bacillota_A	c__Clostridia	o__Oscillospirales	f__Acutalibacteraceae	g__CAG-217	s__CAG-217 sp000436335
Con	Con38	bin30	MAG13	d__Bacteria	p__Bacillota_A	c__Clostridia	o__Oscillospirales	f__Acutalibacteraceae	g__CAG-217	s__CAG-217 sp000436335
Con	Con40	bin17	MAG3129	d__Bacteria	p__Bacillota_A	c__Clostridia	o__Oscillospirales	f__Acutalibacteraceae	g__CAG-217	s__CAG-217 sp000436335
Con	Con3	bin25	MAG3422	d__Bacteria	p__Bacillota_A	c__Clostridia	o__Oscillospirales	f__Acutalibacteraceae	g__CAG-217	s__CAG-217 sp000436335
Con	Con48	bin22	MAG463	d__Bacteria	p__Bacillota_A	c__Clostridia	o__Oscillospirales	f__Acutalibacteraceae	g__CAG-217	s__CAG-217 sp000436335
Con	Con4	bin41	MAG564	d__Bacteria	p__Bacillota_A	c__Clostridia	o__Oscillospirales	f__Acutalibacteraceae	g__CAG-217	s__CAG-217 sp000436335
Con	Con5	bin12	MAG2069	d__Bacteria	p__Bacillota_A	c__Clostridia	o__Oscillospirales	f__Acutalibacteraceae	g__CAG-217	s__CAG-217 sp000436335
Con	Con27	bin13	MAG1698	d__Bacteria	p__Bacillota_A	c__Clostridia	o__Oscillospirales	f__Oscillospiraceae	g__CAG-170	s__CAG-170 sp900545925
Con	Con51	bin36	MAG1248	d__Bacteria	p__Bacillota_A	c__Clostridia	o__Oscillospirales	f__Ruminococcaceae	g__Faecalibacterium	s__Faecalibacterium prausnitzii_E
Con	Con10	bin29	MAG1973	d__Bacteria	p__Bacillota_A	c__Clostridia	o__Oscillospirales	f__Ruminococcaceae	g__Faecalibacterium	s__Faecalibacterium prausnitzii_E
Con	Con50	bin43	MAG783	d__Bacteria	p__Bacillota_A	c__Clostridia	o__Oscillospirales	f__Ruminococcaceae	g__Faecalibacterium	s__Faecalibacterium prausnitzii_E
Con	Con35	bin45	MAG3031	d__Bacteria	p__Bacillota_A	c__Clostridia	o__Oscillospirales	f__Acutalibacteraceae	g__Ruminococcus_E	s__Ruminococcus_E sp934476515
Con	Con18	bin24	MAG1486	d__Bacteria	p__Bacillota_A	c__Clostridia	o__Oscillospirales	f__Acutalibacteraceae	g__Ruminococcus_E	s__Ruminococcus_E sp934476515
Con	Con19	bin51	MAG1755	d__Bacteria	p__Bacillota_A	c__Clostridia	o__Oscillospirales	f__Acutalibacteraceae	g__Ruminococcus_E	s__Ruminococcus_E sp934476515
Con	Con21	bin14	MAG2434	d__Bacteria	p__Bacillota_A	c__Clostridia	o__Oscillospirales	f__Acutalibacteraceae	g__CAG-177	s__CAG-177 sp003514385
Con	Con2	bin53	MAG3234	d__Bacteria	p__Bacillota_A	c__Clostridia	o__Oscillospirales	f__Acutalibacteraceae	g__CAG-177	s__CAG-177 sp003514385
Con	Con49	bin42	MAG1957	d__Bacteria	p__Bacillota_A	c__Clostridia	o__Oscillospirales	f__Acutalibacteraceae	g__CAG-177	s__CAG-177 sp003538135
Con	Con35	bin41	MAG672	d__Bacteria	p__Bacillota_A	c__Clostridia	o__Oscillospirales	f__Ruminococcaceae	g__Faecalibacterium	s__Faecalibacterium prausnitzii_J
Con	Con6	bin1	MAG1990	d__Bacteria	p__Bacillota_A	c__Clostridia	o__Oscillospirales	f__Ruminococcaceae	g__Faecalibacterium	s__Faecalibacterium prausnitzii_I
Con	Con19	bin45	MAG1246	d__Bacteria	p__Bacillota_A	c__Clostridia	o__Oscillospirales	f__Oscillospiraceae	g__CAG-170	s__CAG-170 sp000436735
Con	Con49	bin14	MAG93	d__Bacteria	p__Bacillota_A	c__Clostridia	o__Oscillospirales	f__Oscillospiraceae	g__CAG-170	s__CAG-170 sp000436735
Con	Con12	bin40	MAG1879	d__Bacteria	p__Bacillota_A	c__Clostridia	o__Oscillospirales	f__Oscillospiraceae	g__Dysosmobacter	s__Dysosmobacter welbionis
Con	Con10	bin59	MAG656	d__Bacteria	p__Bacillota_A	c__Clostridia	o__Oscillospirales	f__Oscillospiraceae	g__Dysosmobacter	s__Dysosmobacter welbionis
Con	Con18	bin30	MAG725	d__Bacteria	p__Bacillota_A	c__Clostridia	o__Oscillospirales	f__Oscillospiraceae	g__Dysosmobacter	s__Dysosmobacter welbionis
Con	Con4	bin29	MAG3282	d__Bacteria	p__Bacillota_A	c__Clostridia	o__Oscillospirales	f__Ruminococcaceae	g__Gemmiger	s__Gemmiger formicilis
Con	Con46	bin1	MAG233	d__Bacteria	p__Bacillota_A	c__Clostridia	o__Oscillospirales	f__Ruminococcaceae	g__Gemmiger	s__Gemmiger formicilis
Con	Con50	bin16	MAG673	d__Bacteria	p__Bacillota_A	c__Clostridia	o__Oscillospirales	f__Ruminococcaceae	g__Gemmiger	s__Gemmiger formicilis
Con	Con54	bin11	MAG965	d__Bacteria	p__Bacillota_A	c__Clostridia	o__Oscillospirales	f__Ruminococcaceae	g__Gemmiger	s__Gemmiger formicilis
Con	Con39	bin4	MAG1730	d__Bacteria	p__Bacillota_A	c__Clostridia	o__Oscillospirales	f__Ruminococcaceae	g__Gemmiger	s__Gemmiger formicilis
Con	Con51	bin62	MAG1972	d__Bacteria	p__Bacillota_A	c__Clostridia	o__Oscillospirales	f__Ruminococcaceae	g__Gemmiger	s__Gemmiger formicilis
Con	Con17	bin34	MAG145	d__Bacteria	p__Bacillota_A	c__Clostridia	o__Oscillospirales	f__Ruminococcaceae	g__Gemmiger	s__Gemmiger formicilis
Con	Con49	bin71	MAG805	d__Bacteria	p__Bacillota_A	c__Clostridia	o__Oscillospirales	f__Ruminococcaceae	g__Gemmiger	s__Gemmiger formicilis
Con	Con27	bin1	MAG1072	d__Bacteria	p__Bacillota_A	c__Clostridia	o__Oscillospirales	f__Ruminococcaceae	g__Gemmiger	s__Gemmiger formicilis
Con	Con48	bin25	MAG88	d__Bacteria	p__Bacillota_A	c__Clostridia	o__Oscillospirales	f__Ruminococcaceae	g__Gemmiger	s__Gemmiger formicilis
Con	Con6	bin4	MAG293	d__Bacteria	p__Bacillota_A	c__Clostridia	o__Oscillospirales	f__Ruminococcaceae	g__Gemmiger	s__Gemmiger formicilis
Con	Con5	bin21	MAG816	d__Bacteria	p__Bacillota_A	c__Clostridia	o__Oscillospirales	f__Ruminococcaceae	g__Gemmiger	s__Gemmiger formicilis
Con	Con10	bin5	MAG2755	d__Bacteria	p__Bacillota_A	c__Clostridia	o__Oscillospirales	f__Acutalibacteraceae	g__Pseudoruminococcus	s__Pseudoruminococcus massiliensis
Con	Con19	bin22	MAG3353	d__Bacteria	p__Bacillota_A	c__Clostridia	o__Oscillospirales	f__Acutalibacteraceae	g__UBA1417	s__UBA1417 sp003531055

Con	Con10	bin16	MAG3203	d__Bacteria	p__Bacillota_A	c__Clostridia	o__Oscillospirales	f__Acutalibacteraceae	g__UBA1417	s__UBA1417 sp003531055
Con	Con34	bin10	MAG3153	d__Bacteria	p__Bacillota_A	c__Clostridia	o__Oscillospirales	f__Acutalibacteraceae	g__UBA1417	s__UBA1417 sp003531055
Con	Con9	bin22	MAG2238	d__Bacteria	p__Bacillota_A	c__Clostridia	o__Oscillospirales	f__Acutalibacteraceae	g__UBA1417	s__UBA1417 sp003531055
Con	Con49	bin55	MAG47	d__Bacteria	p__Bacillota_A	c__Clostridia	o__Oscillospirales	f__Ruminococcaceae	g__Ruminiclostridium_E	s__Ruminiclostridium_E siraeum
Con	Con4	bin61	MAG609	d__Bacteria	p__Bacillota_A	c__Clostridia	o__Oscillospirales	f__Ruminococcaceae	g__Ruminiclostridium_E	s__Ruminiclostridium_E siraeum
Con	Con27	bin12	MAG1215	d__Bacteria	p__Bacillota_A	c__Clostridia	o__Oscillospirales	f__Ruminococcaceae	g__Faecalibacterium	s__Faecalibacterium sp900539945
Con	Con39	bin47	MAG1319	d__Bacteria	p__Bacillota_A	c__Clostridia	o__Oscillospirales	f__Ruminococcaceae	g__Faecalibacterium	s__Faecalibacterium sp900539945
Con	Con17	bin7	MAG962	d__Bacteria	p__Bacillota_A	c__Clostridia	o__Oscillospirales	f__Ruminococcaceae	g__Faecalibacterium	s__Faecalibacterium sp900539945
Con	Con45	bin51	MAG2431	d__Bacteria	p__Bacillota_A	c__Clostridia	o__Oscillospirales	f__Ruminococcaceae	g__Faecalibacterium	s__Faecalibacterium sp900539945
Con	Con51	bin2	MAG3270	d__Bacteria	p__Bacillota_A	c__Clostridia	o__Oscillospirales	f__Ruminococcaceae	g__Faecalibacterium	s__Faecalibacterium sp900539945
Con	Con40	bin9	MAG2996	d__Bacteria	p__Bacillota_A	c__Clostridia	o__Oscillospirales	f__Ruminococcaceae	g__Faecalibacterium	s__Faecalibacterium sp900539945
Con	Con10	bin18	MAG284	d__Bacteria	p__Bacillota_A	c__Clostridia	o__Oscillospirales	f__Ruminococcaceae	g__Faecalibacterium	s__Faecalibacterium sp900539945
Con	Con4	bin42	MAG785	d__Bacteria	p__Bacillota_A	c__Clostridia	o__Oscillospirales	f__Ruminococcaceae	g__Faecalibacterium	s__Faecalibacterium sp900539945
Con	Con48	bin30	MAG3464	d__Bacteria	p__Bacillota_A	c__Clostridia	o__Oscillospirales	f__Ruminococcaceae	g__Faecalibacterium	s__Faecalibacterium sp900539945
Con	Con3	bin55	MAG743	d__Bacteria	p__Bacillota_A	c__Clostridia	o__Oscillospirales	f__Ruminococcaceae	g__Ruminococcus_C	s__Ruminococcus_C sp000433635
Con	Con26	bin1	MAG2080	d__Bacteria	p__Bacillota_A	c__Clostridia	o__Oscillospirales	f__Ruminococcaceae	g__Ruminococcus_C	s__Ruminococcus_C sp000433635
Con	Con34	bin53	MAG1838	d__Bacteria	p__Bacillota_A	c__Clostridia	o__Oscillospirales	f__Ruminococcaceae	g__Ruminococcus_C	s__Ruminococcus_C sp000433635
Con	Con34	bin13	MAG2626	d__Bacteria	p__Bacillota_A	c__Clostridia	o__Oscillospirales	f__Acutalibacteraceae	g__UMGS1484	s__UMGS1484 sp900552285
Con	Con24	bin19	MAG1276	d__Bacteria	p__Bacillota_A	c__Clostridia	o__Oscillospirales	f__Oscillospiraceae	g__Flavonifractor	s__Flavonifractor plautii
Con	Con45	bin52	MAG2718	d__Bacteria	p__Bacillota_A	c__Clostridia	o__Oscillospirales	f__Oscillospiraceae	g__Flavonifractor	s__Flavonifractor plautii
Con	Con3	bin18	MAG412	d__Bacteria	p__Bacillota_A	c__Clostridia	o__Oscillospirales	f__Oscillospiraceae	g__Flavonifractor	s__Flavonifractor plautii
Con	Con53	bin14	MAG54	d__Bacteria	p__Bacillota_A	c__Clostridia	o__Oscillospirales	f__Oscillospiraceae	g__Flavonifractor	s__Flavonifractor plautii
Con	Con21	bin35	MAG2006	d__Bacteria	p__Bacillota_A	c__Clostridia	o__Oscillospirales	f__Oscillospiraceae	g__Flavonifractor	s__Flavonifractor plautii
Con	Con32	bin20	MAG3267	d__Bacteria	p__Bacillota_A	c__Clostridia	o__Oscillospirales	f__Oscillospiraceae	g__Flavonifractor	s__Flavonifractor plautii
Con	Con1	bin57	MAG1943	d__Bacteria	p__Bacillota_A	c__Clostridia	o__Oscillospirales	f__Oscillospiraceae	g__Vescimonas	s__Vescimonas coprocola
Con	Con17	bin43	MAG3087	d__Bacteria	p__Bacillota_A	c__Clostridia	o__Oscillospirales	f__Oscillospiraceae	g__Vescimonas	s__Vescimonas coprocola
Con	Con19	bin25	MAG1458	d__Bacteria	p__Bacillota_A	c__Clostridia	o__Oscillospirales	f__Ruminococcaceae	g__Gemmiger	s__Gemmiger sp900539695
Con	Con17	bin56	MAG3026	d__Bacteria	p__Bacillota_A	c__Clostridia	o__Oscillospirales	f__Ruminococcaceae	g__Gemmiger	s__Gemmiger sp900539695
Con	Con3	bin36	MAG2848	d__Bacteria	p__Bacillota_A	c__Clostridia	o__Oscillospirales	f__Ruminococcaceae	g__Gemmiger	s__Gemmiger sp900539695
Con	Con35	bin43	MAG2342	d__Bacteria	p__Bacillota_A	c__Clostridia	o__Oscillospirales	f__Ruminococcaceae	g__Gemmiger	s__Gemmiger sp900539695
Con	Con21	bin1	MAG728	d__Bacteria	p__Bacillota_A	c__Clostridia	o__Oscillospirales	f__Ruminococcaceae	g__Gemmiger	s__Gemmiger sp900539695
Con	Con52	bin11	MAG1461	d__Bacteria	p__Bacillota_A	c__Clostridia	o__Oscillospirales	f__Ruminococcaceae	g__Gemmiger	s__Gemmiger sp900539695
Con	Con10	bin45	MAG2122	d__Bacteria	p__Bacillota_A	c__Clostridia	o__Oscillospirales	f__Ruminococcaceae	g__Gemmiger	s__Gemmiger sp900539695
Con	Con27	bin50	MAG768	d__Bacteria	p__Bacillota_A	c__Clostridia	o__Oscillospirales	f__Ruminococcaceae	g__Gemmiger	s__Gemmiger sp900539695
Con	Con30	bin32	MAG2558	d__Bacteria	p__Bacillota_A	c__Clostridia	o__Oscillospirales	f__Butyricicoccaceae	g__Agathobaculum	s__Agathobaculum sp900291975
Con	Con8	bin23	MAG799	d__Bacteria	p__Bacillota_A	c__Clostridia	o__Oscillospirales	f__Butyricicoccaceae	g__Butyricicoccus	s__Butyricicoccus pullicaecorum
Con	Con1	bin42	MAG1647	d__Bacteria	p__Bacillota_A	c__Clostridia	o__Oscillospirales	f__Ruminococcaceae	g__Negativibacillus	s__Negativibacillus massiliensis
Con	Con45	bin31	MAG1051	d__Bacteria	p__Bacillota_A	c__Clostridia	o__Oscillospirales	f__Acutalibacteraceae	g__UMGS1071	s__UMGS1071 sp900541905
Con	Con49	bin27	MAG2170	d__Bacteria	p__Bacillota_A	c__Clostridia	o__Oscillospirales	f__Oscillospiraceae	g__CAG-170	s__CAG-170 sp000432135
Con	Con49	bin86	MAG3295	d__Bacteria	p__Bacillota_A	c__Clostridia	o__Oscillospirales	f__Oscillospiraceae	g__Vescimonas	s__Vescimonas sp000435555
Con	Con9	bin4	MAG1778	d__Bacteria	p__Bacillota_A	c__Clostridia	o__Oscillospirales	f__Oscillospiraceae	g__Evtepia	s__Evtepia excrementipullorum
Con	Con34	bin37	MAG3205	d__Bacteria	p__Bacillota_A	c__Clostridia	o__Oscillospirales	f__Oscillospiraceae	g__Evtepia	s__Evtepia excrementipullorum
Con	Con34	bin25	MAG1008	d__Bacteria	p__Bacillota_A	c__Clostridia	o__Oscillospirales	f__Oscillospiraceae	g__CAG-170	s__CAG-170 sp900553545
Con	Con1	bin66	MAG2420	d__Bacteria	p__Bacillota_A	c__Clostridia	o__Oscillospirales	f__Oscillospiraceae	g__F23-B02	s__F23-B02 sp002472405
Con	Con26	bin27	MAG2760	d__Bacteria	p__Bacillota_A	c__Clostridia	o__Oscillospirales	f__Ruminococcaceae	g__Faecalibacterium	s__Unclassified
Con	Con1	bin2	MAG724	d__Bacteria	p__Bacillota_A	c__Clostridia	o__Oscillospirales	f__Ruminococcaceae	g__Ruminococcus_C	s__Ruminococcus_C callidus
Con	Con34	bin18	MAG1460	d__Bacteria	p__Bacillota_A	c__Clostridia	o__Oscillospirales	f__Oscillospiraceae	g__Vescimonas	s__Vescimonas sp003487665
Con	Con38	bin1	MAG913	d__Bacteria	p__Bacillota_A	c__Clostridia	o__Oscillospirales	f__Oscillospiraceae	g__Vescimonas	s__Vescimonas sp003487665
Con	Con12	bin21	MAG1805	d__Bacteria	p__Bacillota_A	c__Clostridia	o__Oscillospirales	f__Ruminococcaceae	g__Bittarella	s__Bittarella massiliensis
Con	Con35	bin40	MAG563	d__Bacteria	p__Bacillota_A	c__Clostridia	o__Oscillospirales	f__Ruminococcaceae	g__Ruminococcus_C	s__Ruminococcus_C sp000980705
Con	Con51	bin35	MAG2772	d__Bacteria	p__Bacillota_A	c__Clostridia	o__Oscillospirales	f__Oscillospiraceae	g__ER4	s__ER4 sp900550165

Con	Con19	bin34	MAG2103	d__Bacteria	p__Bacillota_A	c__Clostridia	o__Oscillospirales	f__Oscillospiraceae	g__CAG-103	s__CAG-103 sp900543625
Con	Con27	bin22	MAG3173	d__Bacteria	p__Bacillota_A	c__Clostridia	o__Oscillospirales	f__Acutalibacteraceae	g__Ruminococcus_E	s__Ruminococcus_E bromii_B
Con	Con31	bin42	MAG2623	d__Bacteria	p__Bacillota_A	c__Clostridia	o__Oscillospirales	f__Acutalibacteraceae	g__Ruminococcus_E	s__Ruminococcus_E bromii_B
Con	Con45	bin35	MAG2167	d__Bacteria	p__Bacillota_A	c__Clostridia	o__Oscillospirales	f__Acutalibacteraceae	g__Ruminococcus_E	s__Ruminococcus_E bromii_B
Con	Con6	bin18	MAG575	d__Bacteria	p__Bacillota_A	c__Clostridia	o__Oscillospirales	f__Acutalibacteraceae	g__Ruminococcus_E	s__Ruminococcus_E bromii_B
Con	Con39	bin48	MAG140	d__Bacteria	p__Bacillota_A	c__Clostridia	o__Oscillospirales	f__Acutalibacteraceae	g__Ruminococcus_E	s__Ruminococcus_E bromii_B
Con	Con48	bin27	MAG893	d__Bacteria	p__Bacillota_A	c__Clostridia	o__Oscillospirales	f__Acutalibacteraceae	g__Ruminococcus_E	s__Ruminococcus_E bromii_B
Con	Con34	bin7	MAG2783	d__Bacteria	p__Bacillota_A	c__Clostridia	o__Oscillospirales	f__Acutalibacteraceae	g__Ruminococcus_E	s__Ruminococcus_E bromii_B
Con	Con40	bin2	MAG1533	d__Bacteria	p__Bacillota_A	c__Clostridia	o__Oscillospirales	f__Acutalibacteraceae	g__Ruminococcus_E	s__Ruminococcus_E bromii_B
Con	Con17	bin64	MAG2213	d__Bacteria	p__Bacillota_A	c__Clostridia	o__Oscillospirales	f__Acutalibacteraceae	g__Ruminococcus_E	s__Ruminococcus_E bromii_B
Con	Con26	bin15	MAG3156	d__Bacteria	p__Bacillota_A	c__Clostridia	o__Oscillospirales	f__Acutalibacteraceae	g__Ruminococcus_E	s__Ruminococcus_E bromii_B
Con	Con49	bin28	MAG2373	d__Bacteria	p__Bacillota_A	c__Clostridia	o__Oscillospirales	f__Acutalibacteraceae	g__Ruminococcus_E	s__Ruminococcus_E bromii_B
Con	Con21	bin36	MAG3485	d__Bacteria	p__Bacillota_A	c__Clostridia	o__Oscillospirales	f__Acutalibacteraceae	g__Ruminococcus_E	s__Ruminococcus_E bromii_B
Con	Con2	bin65	MAG3233	d__Bacteria	p__Bacillota_A	c__Clostridia	o__Oscillospirales	f__Acutalibacteraceae	g__Ruminococcus_E	s__Ruminococcus_E bromii_B
Con	Con2	bin64	MAG810	d__Bacteria	p__Bacillota_A	c__Clostridia	o__Peptostreptococcales	f__Anaerovoracaceae	g__Lentihominibacter	s__Lentihominibacter sp934281605
Con	Con17	bin26	MAG2523	d__Bacteria	p__Bacillota_A	c__Clostridia	o__Peptostreptococcales	f__Anaerovoracaceae	g__Lentihominibacter	s__Lentihominibacter sp934281605
Con	Con9	bin26	MAG904	d__Bacteria	p__Bacillota_A	c__Clostridia	o__Peptostreptococcales	f__Anaerovoracaceae	g__Lentihominibacter	s__Lentihominibacter sp934281605
Con	Con4	bin20	MAG3119	d__Bacteria	p__Bacillota_A	c__Clostridia	o__Peptostreptococcales	f__Peptostreptococcaceae	g__Peptacetobacter	s__Peptacetobacter hiranonis
Con	Con26	bin5	MAG2497	d__Bacteria	p__Bacillota_A	c__Clostridia	o__Peptostreptococcales	f__Peptostreptococcaceae	g__Peptacetobacter	s__Peptacetobacter sp900539645
Con	Con2	bin8	MAG137	d__Bacteria	p__Bacillota_A	c__Clostridia	o__Peptostreptococcales	f__Anaerovoracaceae	g__Lentihominibacter	s__Lentihominibacter sp900066305
Con	Con40	bin52	MAG1003	d__Bacteria	p__Bacillota_A	c__Clostridia	o__Peptostreptococcales	f__Peptostreptococcaceae	g__Romboutsia	s__Romboutsia timonensis
Con	Con24	bin5	MAG341	d__Bacteria	p__Bacillota_A	c__Clostridia	o__Peptostreptococcales	f__Peptostreptococcaceae	g__Romboutsia	s__Romboutsia timonensis
Con	Con19	bin43	MAG1039	d__Bacteria	p__Bacillota_A	c__Clostridia	o__Peptostreptococcales	f__Peptostreptococcaceae	g__Romboutsia	s__Romboutsia timonensis
Con	Con34	bin60	MAG110	d__Bacteria	p__Bacillota_A	c__Clostridia	o__Peptostreptococcales	f__Peptostreptococcaceae	g__Romboutsia	s__Romboutsia timonensis
Con	Con17	bin59	MAG2662	d__Bacteria	p__Bacillota_A	c__Clostridia	o__Peptostreptococcales	f__Peptostreptococcaceae	g__Romboutsia	s__Romboutsia timonensis
Con	Con49	bin9	MAG648	d__Bacteria	p__Bacillota_A	c__Clostridia	o__TANB77	f__CAG-508	g__CAG-273	s__CAG-273 sp003534295
Con	Con34	bin6	MAG2368	d__Bacteria	p__Bacillota_A	c__Clostridia	o__TANB77	f__CAG-508	g__CAG-269	s__CAG-269 sp001916005
Con	Con19	bin14	MAG1167	d__Bacteria	p__Bacillota_A	c__Clostridia	o__TANB77	f__CAG-508	g__CAG-269	s__CAG-269 sp000437215
Con	Con34	bin19	MAG436	d__Bacteria	p__Bacillota_A	c__Clostridia	o__TANB77	f__CAG-508	g__CAG-269	s__CAG-269 sp003525075
Con	Con38	bin51	MAG2743	d__Bacteria	p__Bacillota_A	c__Clostridia	o__TANB77	f__CAG-508	g__CAG-245	s__CAG-245 sp000435175
Con	Con9	bin3	MAG2869	d__Bacteria	p__Bacillota_A	c__Clostridia	o__TANB77	f__CAG-508	g__CAG-245	s__CAG-245 sp000435175
Con	Con38	bin40	MAG358	d__Bacteria	p__Bacillota_A	c__Clostridia	o__TANB77	f__CAG-508	g__CAG-269	s__CAG-269 sp905203195
Con	Con49	bin35	MAG2375	d__Bacteria	p__Bacillota_A	c__Clostridia	o__TANB77	f__CAG-508	g__CAG-269	s__CAG-269 sp001916055
Con	Con27	bin32	MAG2827	d__Bacteria	p__Bacillota_A	c__Clostridia	o__TANB77	f__CAG-508	g__CAG-269	s__CAG-269 sp900556345
Con	Con34	bin38	MAG2221	d__Bacteria	p__Bacillota_A	c__Clostridia	o__TANB77	f__CAG-508	g__CAG-269	s__CAG-269 sp900556345
Con	Con38	bin24	MAG771	d__Bacteria	p__Bacillota_A	c__Clostridia	o__TANB77	f__CAG-508	g__CAG-245	s__CAG-245 sp000434195
Con	Con32	bin46	MAG3316	d__Bacteria	p__Bacillota_A	c__Clostridia	o__UBA1381	f__UBA1381	g__CAG-41	s__CAG-41 sp900066215
Con	Con40	bin24	MAG2796	d__Bacteria	p__Bacillota_A	c__Clostridia	o__UBA1381	f__UBA1381	g__CAG-41	s__CAG-41 sp900066215
Con	Con1	bin4	MAG1151	d__Bacteria	p__Bacillota_A	c__Clostridia	o__UBA1381	f__UBA1381	g__CAG-41	s__CAG-41 sp900066215
Con	Con4	bin52	MAG1950	d__Bacteria	p__Bacillota_A	c__Clostridia	o__UBA1381	f__UBA1381	g__CAG-41	s__CAG-41 sp900066215
Con	Con48	bin15	MAG635	d__Bacteria	p__Bacillota_A	c__Clostridia	o__UBA1381	f__UBA1381	g__CAG-41	s__CAG-41 sp900066215
Con	Con2	bin14	MAG2897	d__Bacteria	p__Bacillota_A	c__Clostridia	o__UBA1381	f__UBA1381	g__CAG-41	s__CAG-41 sp900066215
Con	Con34	bin55	MAG2920	d__Bacteria	p__Bacillota_A	c__Clostridia	o__UBA1381	f__UBA1381	g__CAG-41	s__CAG-41 sp900066215
Con	Con18	bin29	MAG3040	d__Bacteria	p__Bacillota_A	c__Clostridia	o__UBA1381	f__UBA1381	g__CAG-41	s__CAG-41 sp900066215
Con	Con45	bin37	MAG751	d__Bacteria	p__Bacillota_A	c__Clostridia	o__UBA1381	f__UBA1381	g__CAG-41	s__CAG-41 sp900066215
Con	Con44	bin30	MAG458	d__Bacteria	p__Bacillota_A	c__Clostridia	o__UBA1381	f__UBA1381	g__CAG-41	s__CAG-41 sp900066215
Con	Con10	bin51	MAG1865	d__Bacteria	p__Bacillota_A	c__Clostridia	o__UBA1381	f__UBA1381	g__CAG-41	s__CAG-41 sp900066215
Con	Con35	bin26	MAG1363	d__Bacteria	p__Bacillota_A	c__Clostridia	o__UBA1381	f__UBA1381	g__CAG-41	s__CAG-41 sp900066215
Con	Con27	bin10	MAG364	d__Bacteria	p__Bacillota_A	c__Clostridia	o__UBA1381	f__UBA1381	g__CAG-41	s__CAG-41 sp900066215
Con	Con22	bin7	MAG2741	d__Bacteria	p__Bacillota_A	c__Clostridia	o__UBA1381	f__UBA1381	g__CAG-41	s__CAG-41 sp900066215
Con	Con39	bin31	MAG495	d__Bacteria	p__Bacillota_A	c__Clostridia	o__UBA1381	f__UBA1381	g__CAG-41	s__CAG-41 sp900066215

Con	Con26	bin4	MAG1093	d__Bacteria	p__Bacillota_A	c__Clostridia	o__UBA1381	f__UBA1381	g__CAG-41	s__CAG-41 sp900066215
Con	Con28	bin5	MAG448	d__Bacteria	p__Bacillota_A	c__Clostridia	o__UBA1381	f__UBA1381	g__CAG-41	s__CAG-41 sp900066215
