# Supplementary material for: Gut microbial community of patients with Parkinson’s disease analyzed using metagenome-assembled genomes
Source: Neural Regen Res. 2025 Sep 29;21(8):3815–23. doi: 10.4103/NRR.NRR-D-25-00420 (PMC13460800; doi:10.4103/NRR.NRR-D-25-00420)
Supplement: Supplementary file 3 [file NRR-21-3815_Suppl3.pdf]

Additional Table 4 MAGs with different abundance between PD patients and healthy controls

MAG	Participants	Domain	Phylum	Class	Order	Family	Genus	Species
MAG1086	PD	d__Bacteria	p__Synergistota	c__Synergistia	o__Synergistales	f__Synergistaceae	g__Cloacibacillus	s__Cloacibacillus evryensis
MAG845	PD	d__Bacteria	p__Synergistota	c__Synergistia	o__Synergistales	f__Dethiosulfovibrionaceae	g__Pyramidobacter	s__Pyramidobacter piscolens
MAG39	PD	d__Bacteria	p__Pseudomonadota	c__Gammaproteobacteria	o__Burkholderiales	f__Burkholderiaceae_A	g__Parasutterella	s__Parasutterella excrementihominis
MAG1884	PD	d__Bacteria	p__Pseudomonadota	c__Gammaproteobacteria	o__Burkholderiales	f__Burkholderiaceae_A	g__Parasutterella	s__Parasutterella excrementihominis
MAG3013	PD	d__Bacteria	p__Pseudomonadota	c__Gammaproteobacteria	o__Burkholderiales	f__Burkholderiaceae_A	g__Parasutterella	s__Parasutterella excrementihominis
MAG1375	PD	d__Bacteria	p__Desulfobacterota	c__Desulfovibrionia	o__Desulfovibrionales	f__Desulfovibrionaceae	g__Bilophila	s__Bilophila wadsworthia
MAG255	PD	d__Bacteria	p__Desulfobacterota	c__Desulfovibrionia	o__Desulfovibrionales	f__Desulfovibrionaceae	g__Bilophila	s__Bilophila wadsworthia
MAG2511	PD	d__Bacteria	p__Desulfobacterota	c__Desulfovibrionia	o__Desulfovibrionales	f__Desulfovibrionaceae	g__Bilophila	s__Bilophila wadsworthia
MAG3179	PD	d__Bacteria	p__Bacteroidota	c__Bacteroidia	o__Bacteroidales	f__Tannerellaceae	g__Parabacteroides	s__Parabacteroides merdae
MAG3240	PD	d__Bacteria	p__Bacteroidota	c__Bacteroidia	o__Bacteroidales	f__Tannerellaceae	g__Parabacteroides	s__Parabacteroides merdae
MAG548	PD	d__Bacteria	p__Bacteroidota	c__Bacteroidia	o__Bacteroidales	f__Tannerellaceae	g__Parabacteroides	s__Parabacteroides merdae
MAG3375	PD	d__Bacteria	p__Bacteroidota	c__Bacteroidia	o__Bacteroidales	f__Tannerellaceae	g__Parabacteroides	s__Parabacteroides merdae
MAG3174	PD	d__Bacteria	p__Bacteroidota	c__Bacteroidia	o__Bacteroidales	f__Rikenellaceae	g__Alistipes_A	s__Alistipes_A pullistercoris
MAG1803	PD	d__Bacteria	p__Bacteroidota	c__Bacteroidia	o__Bacteroidales	f__Rikenellaceae	g__Alistipes_A	s__Alistipes_A pullistercoris
MAG2687	PD	d__Bacteria	p__Bacteroidota	c__Bacteroidia	o__Bacteroidales	f__Rikenellaceae	g__Alistipes_A	s__Alistipes_A pullistercoris
MAG1187	PD	d__Bacteria	p__Bacteroidota	c__Bacteroidia	o__Bacteroidales	f__Rikenellaceae	g__Alistipes	s__Alistipes shahii
MAG1445	PD	d__Bacteria	p__Bacteroidota	c__Bacteroidia	o__Bacteroidales	f__Rikenellaceae	g__Alistipes	s__Alistipes shahii
MAG659	PD	d__Bacteria	p__Bacteroidota	c__Bacteroidia	o__Bacteroidales	f__Rikenellaceae	g__Alistipes	s__Alistipes shahii
MAG1404	PD	d__Bacteria	p__Bacteroidota	c__Bacteroidia	o__Bacteroidales	f__Rikenellaceae	g__Alistipes	s__Alistipes onderdonkii
MAG3096	PD	d__Bacteria	p__Bacteroidota	c__Bacteroidia	o__Bacteroidales	f__Rikenellaceae	g__Alistipes	s__Alistipes onderdonkii
MAG235	PD	d__Bacteria	p__Bacteroidota	c__Bacteroidia	o__Bacteroidales	f__Rikenellaceae	g__Alistipes	s__Alistipes onderdonkii
MAG2281	PD	d__Bacteria	p__Bacteroidota	c__Bacteroidia	o__Bacteroidales	f__Rikenellaceae	g__Alistipes	s__Alistipes onderdonkii
MAG2746	PD	d__Bacteria	p__Bacteroidota	c__Bacteroidia	o__Bacteroidales	f__Rikenellaceae	g__Alistipes	s__Alistipes onderdonkii
MAG3318	PD	d__Bacteria	p__Bacteroidota	c__Bacteroidia	o__Bacteroidales	f__Rikenellaceae	g__Alistipes	s__Alistipes onderdonkii
MAG3072	PD	d__Bacteria	p__Bacteroidota	c__Bacteroidia	o__Bacteroidales	f__Rikenellaceae	g__Alistipes	s__Alistipes onderdonkii
MAG3030	PD	d__Bacteria	p__Bacteroidota	c__Bacteroidia	o__Bacteroidales	f__Rikenellaceae	g__Alistipes	s__Alistipes finegoldii
MAG2512	PD	d__Bacteria	p__Bacteroidota	c__Bacteroidia	o__Bacteroidales	f__Rikenellaceae	g__Alistipes	s__Alistipes finegoldii
MAG2524	PD	d__Bacteria	p__Bacteroidota	c__Bacteroidia	o__Bacteroidales	f__Rikenellaceae	g__Alistipes	s__Alistipes finegoldii
MAG199	PD	d__Bacteria	p__Bacteroidota	c__Bacteroidia	o__Bacteroidales	f__Rikenellaceae	g__Alistipes	s__Alistipes communis
MAG433	PD	d__Bacteria	p__Bacteroidota	c__Bacteroidia	o__Bacteroidales	f__Rikenellaceae	g__Alistipes	s__Alistipes communis
MAG2391	PD	d__Bacteria	p__Bacteroidota	c__Bacteroidia	o__Bacteroidales	f__Marinifilaceae	g__Butyricimonas	s__Butyricimonas virosa
MAG2937	PD	d__Bacteria	p__Bacteroidota	c__Bacteroidia	o__Bacteroidales	f__Marinifilaceae	g__Butyricimonas	s__Butyricimonas virosa
MAG1179	PD	d__Bacteria	p__Bacteroidota	c__Bacteroidia	o__Bacteroidales	f__Marinifilaceae	g__Butyricimonas	s__Butyricimonas virosa
MAG722	PD	d__Bacteria	p__Bacteroidota	c__Bacteroidia	o__Bacteroidales	f__Marinifilaceae	g__Butyricimonas	s__Butyricimonas virosa
MAG2972	PD	d__Bacteria	p__Bacteroidota	c__Bacteroidia	o__Bacteroidales	f__Marinifilaceae	g__Butyricimonas	s__Butyricimonas virosa
MAG3427	Con	d__Bacteria	p__Bacteroidota	c__Bacteroidia	o__Bacteroidales	f__Bacteroidaceae	g__Bacteroides	s__Bacteroides thetaiotaomicron
MAG2967	PD	d__Bacteria	p__Bacteroidota	c__Bacteroidia	o__Bacteroidales	f__Bacteroidaceae	g__Bacteroides	s__Bacteroides thetaiotaomicron
MAG828	PD	d__Bacteria	p__Bacteroidota	c__Bacteroidia	o__Bacteroidales	f__Bacteroidaceae	g__Bacteroides	s__Bacteroides intestinalis_A
MAG2847	PD	d__Bacteria	p__Bacillota_C	c__Negativicutes	o__Selenomonadales	f__Selenomonadaceae	g__Megamonas	s__Megamonas funiformis
MAG1843	PD	d__Bacteria	p__Bacillota_C	c__Negativicutes	o__Acidaminococcales	f__Acidaminococcaceae	g__Phascolarctobacterium	s__Phascolarctobacterium faecium
MAG1184	PD	d__Bacteria	p__Bacillota_C	c__Negativicutes	o__Acidaminococcales	f__Acidaminococcaceae	g__Phascolarctobacterium	s__Phascolarctobacterium faecium
MAG2644	PD	d__Bacteria	p__Bacillota_C	c__Negativicutes	o__Acidaminococcales	f__Acidaminococcaceae	g__Phascolarctobacterium	s__Phascolarctobacterium faecium
MAG919	PD	d__Bacteria	p__Bacillota_C	c__Negativicutes	o__Acidaminococcales	f__Acidaminococcaceae	g__Phascolarctobacterium	s__Phascolarctobacterium faecium
MAG1654	Con	d__Bacteria	p__Bacillota_A	c__Clostridia	o__UBA1381	f__UBA1381	g__CAG-41	s__CAG-41 sp900066215
MAG697	PD	d__Bacteria	p__Bacillota_A	c__Clostridia	o__Peptostreptococcales	f__Anaerovoracaceae	g__Zhenpiania	s__Zhenpiania hominis
MAG178	PD	d__Bacteria	p__Bacillota_A	c__Clostridia	o__Peptostreptococcales	f__Anaerovoracaceae	g__Lentihominibacter	s__Lentihominibacter faecis
MAG824	PD	d__Bacteria	p__Bacillota_A	c__Clostridia	o__Peptostreptococcales	f__Anaerovoracaceae	g__Lentihominibacter	s__Lentihominibacter faecis
MAG3397	PD	d__Bacteria	p__Bacillota_A	c__Clostridia	o__Peptostreptococcales	f__Anaerovoracaceae	g__Lentihominibacter	s__Lentihominibacter faecis
MAG903	PD	d__Bacteria	p__Bacillota_A	c__Clostridia	o__Peptostreptococcales	f__Anaerovoracaceae	g__Lentihominibacter	s__Lentihominibacter faecis

MAG1204	PD	d__Bacteria	p__Bacillota_A	c__Clostridia	o__Peptostreptococcales	f__Anaerovoracaceae	g__Lentihominibacter	s__Lentihominibacter faecis
MAG1833	PD	d__Bacteria	p__Bacillota_A	c__Clostridia	o__Peptostreptococcales	f__Anaerovoracaceae	g__Lentihominibacter	s__Lentihominibacter faecis
MAG1099	PD	d__Bacteria	p__Bacillota_A	c__Clostridia	o__Peptostreptococcales	f__Anaerovoracaceae	g__Lentihominibacter	s__Lentihominibacter faecis
MAG2344	PD	d__Bacteria	p__Bacillota_A	c__Clostridia	o__Peptostreptococcales	f__Anaerovoracaceae	g__Fimisoma	s__Fimisoma sp000435715
MAG527	PD	d__Bacteria	p__Bacillota_A	c__Clostridia	o__Oscillospirales	f__UBA644	g__UBA644	s__UBA644 sp900547165
MAG701	PD	d__Bacteria	p__Bacillota_A	c__Clostridia	o__Oscillospirales	f__Ruminococcaceae	g__Ruthenibacterium	s__Ruthenibacterium lactatiformans
MAG3399	PD	d__Bacteria	p__Bacillota_A	c__Clostridia	o__Oscillospirales	f__Ruminococcaceae	g__Ruthenibacterium	s__Ruthenibacterium lactatiformans
MAG2134	PD	d__Bacteria	p__Bacillota_A	c__Clostridia	o__Oscillospirales	f__Ruminococcaceae	g__Ruthenibacterium	s__Ruthenibacterium lactatiformans
MAG1112	PD	d__Bacteria	p__Bacillota_A	c__Clostridia	o__Oscillospirales	f__Ruminococcaceae	g__Ruthenibacterium	s__Ruthenibacterium lactatiformans
MAG2186	PD	d__Bacteria	p__Bacillota_A	c__Clostridia	o__Oscillospirales	f__Ruminococcaceae	g__Ruthenibacterium	s__Ruthenibacterium lactatiformans
MAG2380	PD	d__Bacteria	p__Bacillota_A	c__Clostridia	o__Oscillospirales	f__Ruminococcaceae	g__Ruthenibacterium	s__Ruthenibacterium lactatiformans
MAG916	PD	d__Bacteria	p__Bacillota_A	c__Clostridia	o__Oscillospirales	f__Ruminococcaceae	g__Ruthenibacterium	s__Ruthenibacterium lactatiformans
MAG769	PD	d__Bacteria	p__Bacillota_A	c__Clostridia	o__Oscillospirales	f__Ruminococcaceae	g__Ruthenibacterium	s__Ruthenibacterium lactatiformans
MAG2534	PD	d__Bacteria	p__Bacillota_A	c__Clostridia	o__Oscillospirales	f__Ruminococcaceae	g__Ruthenibacterium	s__Ruthenibacterium lactatiformans
MAG1036	PD	d__Bacteria	p__Bacillota_A	c__Clostridia	o__Oscillospirales	f__Ruminococcaceae	g__Ruthenibacterium	s__Ruthenibacterium lactatiformans
MAG1258	PD	d__Bacteria	p__Bacillota_A	c__Clostridia	o__Oscillospirales	f__Ruminococcaceae	g__Ruthenibacterium	s__Ruthenibacterium lactatiformans
MAG3493	PD	d__Bacteria	p__Bacillota_A	c__Clostridia	o__Oscillospirales	f__Ruminococcaceae	g__Ruthenibacterium	s__Ruthenibacterium lactatiformans
MAG3071	PD	d__Bacteria	p__Bacillota_A	c__Clostridia	o__Oscillospirales	f__Ruminococcaceae	g__Ruthenibacterium	s__Ruthenibacterium lactatiformans
MAG2013	PD	d__Bacteria	p__Bacillota_A	c__Clostridia	o__Oscillospirales	f__Ruminococcaceae	g__Ruthenibacterium	s__Ruthenibacterium lactatiformans
MAG1663	PD	d__Bacteria	p__Bacillota_A	c__Clostridia	o__Oscillospirales	f__Ruminococcaceae	g__Ruthenibacterium	s__Ruthenibacterium lactatiformans
MAG415	PD	d__Bacteria	p__Bacillota_A	c__Clostridia	o__Oscillospirales	f__Ruminococcaceae	g__Ruminococcus_D	s__Ruminococcus_D bicirculans
MAG3395	PD	d__Bacteria	p__Bacillota_A	c__Clostridia	o__Oscillospirales	f__Ruminococcaceae	g__Ruminococcus_D	s__Ruminococcus_D bicirculans
MAG1265	PD	d__Bacteria	p__Bacillota_A	c__Clostridia	o__Oscillospirales	f__Ruminococcaceae	g__Ruminococcus_D	s__Ruminococcus_D bicirculans
MAG741	PD	d__Bacteria	p__Bacillota_A	c__Clostridia	o__Oscillospirales	f__Ruminococcaceae	g__Ruminococcus_D	s__Ruminococcus_D bicirculans
MAG1019	PD	d__Bacteria	p__Bacillota_A	c__Clostridia	o__Oscillospirales	f__Ruminococcaceae	g__Ruminococcus_D	s__Ruminococcus_D bicirculans
MAG807	PD	d__Bacteria	p__Bacillota_A	c__Clostridia	o__Oscillospirales	f__Ruminococcaceae	g__Ruminococcus_D	s__Ruminococcus_D bicirculans
MAG3413	PD	d__Bacteria	p__Bacillota_A	c__Clostridia	o__Oscillospirales	f__Ruminococcaceae	g__Faecalibacterium	s__Faecalibacterium prausnitzii_D
MAG562	PD	d__Bacteria	p__Bacillota_A	c__Clostridia	o__Oscillospirales	f__Ruminococcaceae	g__Faecalibacterium	s__Faecalibacterium prausnitzii_D
MAG2468	PD	d__Bacteria	p__Bacillota_A	c__Clostridia	o__Oscillospirales	f__Ruminococcaceae	g__Faecalibacterium	s__Faecalibacterium prausnitzii_D
MAG2256	PD	d__Bacteria	p__Bacillota_A	c__Clostridia	o__Oscillospirales	f__Ruminococcaceae	g__Faecalibacterium	s__Faecalibacterium prausnitzii_D
MAG1780	PD	d__Bacteria	p__Bacillota_A	c__Clostridia	o__Oscillospirales	f__Ruminococcaceae	g__Faecalibacterium	s__Faecalibacterium prausnitzii_D
MAG11	PD	d__Bacteria	p__Bacillota_A	c__Clostridia	o__Oscillospirales	f__Ruminococcaceae	g__Angelakisella	s__Angelakisella sp900547385
MAG2865	PD	d__Bacteria	p__Bacillota_A	c__Clostridia	o__Oscillospirales	f__Ruminococcaceae	g__Angelakisella	s__Angelakisella sp900547385
MAG491	PD	d__Bacteria	p__Bacillota_A	c__Clostridia	o__Oscillospirales	f__Oscillospiraceae	g__Vescimonas	s__Vescimonas sp003539495
MAG2654	PD	d__Bacteria	p__Bacillota_A	c__Clostridia	o__Oscillospirales	f__Oscillospiraceae	g__Vescimonas	s__Vescimonas coprocola
MAG1795	PD	d__Bacteria	p__Bacillota_A	c__Clostridia	o__Oscillospirales	f__Oscillospiraceae	g__Vescimonas	s__Vescimonas coprocola
MAG71	PD	d__Bacteria	p__Bacillota_A	c__Clostridia	o__Oscillospirales	f__Oscillospiraceae	g__Vescimonas	s__Vescimonas coprocola
MAG2797	PD	d__Bacteria	p__Bacillota_A	c__Clostridia	o__Oscillospirales	f__Oscillospiraceae	g__Vescimonas	s__Vescimonas coprocola
MAG74	PD	d__Bacteria	p__Bacillota_A	c__Clostridia	o__Oscillospirales	f__Oscillospiraceae	g__Pseudoflavonifractor	s__Pseudoflavonifractor sp014287675
MAG1282	PD	d__Bacteria	p__Bacillota_A	c__Clostridia	o__Oscillospirales	f__Oscillospiraceae	g__NSJ-51	s__NSJ-51 sp900549795
MAG2585	PD	d__Bacteria	p__Bacillota_A	c__Clostridia	o__Oscillospirales	f__Oscillospiraceae	g__Faecousia	s__Faecousia sp003525905
MAG993	PD	d__Bacteria	p__Bacillota_A	c__Clostridia	o__Oscillospirales	f__Oscillospiraceae	g__Faecousia	s__Faecousia sp003525905
MAG249	PD	d__Bacteria	p__Bacillota_A	c__Clostridia	o__Oscillospirales	f__Oscillospiraceae	g__Faecousia	s__Faecousia sp003525905
MAG994	PD	d__Bacteria	p__Bacillota_A	c__Clostridia	o__Oscillospirales	f__Oscillospiraceae	g__Faecousia	s__Faecousia sp003525905
MAG176	PD	d__Bacteria	p__Bacillota_A	c__Clostridia	o__Oscillospirales	f__Oscillospiraceae	g__Faecousia	s__Faecousia sp003525905
MAG99	PD	d__Bacteria	p__Bacillota_A	c__Clostridia	o__Oscillospirales	f__Oscillospiraceae	g__Faecousia	s__Faecousia sp003525905
MAG623	PD	d__Bacteria	p__Bacillota_A	c__Clostridia	o__Oscillospirales	f__Oscillospiraceae	g__Faecousia	s__Faecousia sp003525905
MAG1542	PD	d__Bacteria	p__Bacillota_A	c__Clostridia	o__Oscillospirales	f__Oscillospiraceae	g__Faecousia	s__Faecousia sp003525905
MAG1426	PD	d__Bacteria	p__Bacillota_A	c__Clostridia	o__Oscillospirales	f__Oscillospiraceae	g__Faecousia	s__Faecousia sp000435995
MAG3414	PD	d__Bacteria	p__Bacillota_A	c__Clostridia	o__Oscillospirales	f__Oscillospiraceae	g__Faecousia	s__Faecousia sp000434635
MAG1692	PD	d__Bacteria	p__Bacillota_A	c__Clostridia	o__Oscillospirales	f__Oscillospiraceae	g__Evtepia	s__Evtepia gabavorous

MAG3195	PD	d__Bacteria	p__Bacillota_A	c__Clostridia	o__Oscillospirales	f__Oscillospiraceae	g__Evtepia	s__Evtepia gabavorous
MAG712	PD	d__Bacteria	p__Bacillota_A	c__Clostridia	o__Oscillospirales	f__Oscillospiraceae	g__ER4	s__ER4 sp900317525
MAG3236	PD	d__Bacteria	p__Bacillota_A	c__Clostridia	o__Oscillospirales	f__Oscillospiraceae	g__ER4	s__ER4 sp900317525
MAG521	PD	d__Bacteria	p__Bacillota_A	c__Clostridia	o__Oscillospirales	f__Oscillospiraceae	g__ER4	s__ER4 sp900317525
MAG1610	PD	d__Bacteria	p__Bacillota_A	c__Clostridia	o__Oscillospirales	f__Oscillospiraceae	g__ER4	s__ER4 sp900317525
MAG2268	PD	d__Bacteria	p__Bacillota_A	c__Clostridia	o__Oscillospirales	f__Oscillospiraceae	g__ER4	s__ER4 sp900317525
MAG2049	PD	d__Bacteria	p__Bacillota_A	c__Clostridia	o__Oscillospirales	f__Oscillospiraceae	g__ER4	s__ER4 sp000765235
MAG2679	Con	d__Bacteria	p__Bacillota_A	c__Clostridia	o__Oscillospirales	f__Oscillospiraceae	g__ER4	s__ER4 sp000765235
MAG3407	PD	d__Bacteria	p__Bacillota_A	c__Clostridia	o__Oscillospirales	f__Oscillospiraceae	g__ER4	s__ER4 sp000765235
MAG1234	PD	d__Bacteria	p__Bacillota_A	c__Clostridia	o__Oscillospirales	f__Oscillospiraceae	g__ER4	s__ER4 sp000765235
MAG3062	PD	d__Bacteria	p__Bacillota_A	c__Clostridia	o__Oscillospirales	f__Oscillospiraceae	g__CAG-170	s__CAG-170 sp002404795
MAG2945	PD	d__Bacteria	p__Bacillota_A	c__Clostridia	o__Oscillospirales	f__Oscillospiraceae	g__CAG-103	s__CAG-103 sp900757655
MAG2951	PD	d__Bacteria	p__Bacillota_A	c__Clostridia	o__Oscillospirales	f__Oscillospiraceae	g__CAG-103	s__CAG-103 sp900757655
MAG3374	PD	d__Bacteria	p__Bacillota_A	c__Clostridia	o__Oscillospirales	f__Oscillospiraceae	g__CAG-103	s__CAG-103 sp900757655
MAG2506	PD	d__Bacteria	p__Bacillota_A	c__Clostridia	o__Oscillospirales	f__Oscillospiraceae	g__CAG-103	s__CAG-103 sp900757655
MAG2915	PD	d__Bacteria	p__Bacillota_A	c__Clostridia	o__Oscillospirales	f__Oscillospiraceae	g__CAG-103	s__CAG-103 sp000432375
MAG1405	PD	d__Bacteria	p__Bacillota_A	c__Clostridia	o__Oscillospirales	f__Oscillospiraceae	g__CAG-103	s__CAG-103 sp000432375
MAG2200	PD	d__Bacteria	p__Bacillota_A	c__Clostridia	o__Oscillospirales	f__Oscillospiraceae	g__CAG-103	s__CAG-103 sp000432375
MAG3160	PD	d__Bacteria	p__Bacillota_A	c__Clostridia	o__Oscillospirales	f__Acutalibacteraceae	g__Eubacterium_R	s__Eubacterium_R sp000434995
MAG1069	PD	d__Bacteria	p__Bacillota_A	c__Clostridia	o__Oscillospirales	f__Acutalibacteraceae	g__CAG-177	s__CAG-177 sp003514385
MAG1616	PD	d__Bacteria	p__Bacillota_A	c__Clostridia	o__Oscillospirales	f__Acutalibacteraceae	g__CAG-177	s__CAG-177 sp003514385
MAG94	PD	d__Bacteria	p__Bacillota_A	c__Clostridia	o__Oscillospirales	f__Acutalibacteraceae	g__CAG-177	s__CAG-177 sp003514385
MAG201	PD	d__Bacteria	p__Bacillota_A	c__Clostridia	o__Oscillospirales	f__Acutalibacteraceae	g__CAG-177	s__CAG-177 sp003514385
MAG3377	PD	d__Bacteria	p__Bacillota_A	c__Clostridia	o__Oscillospirales	f__Acutalibacteraceae	g__CAG-177	s__CAG-177 sp003514385
MAG2382	PD	d__Bacteria	p__Bacillota_A	c__Clostridia	o__Oscillospirales	f__Acutalibacteraceae	g__CAG-177	s__CAG-177 sp003514385
MAG887	PD	d__Bacteria	p__Bacillota_A	c__Clostridia	o__Oscillospirales	f__Acutalibacteraceae	g__CAG-177	s__CAG-177 sp003514385
MAG954	PD	d__Bacteria	p__Bacillota_A	c__Clostridia	o__Oscillospirales	f__Acutalibacteraceae	g__CAG-177	s__CAG-177 sp003514385
MAG208	PD	d__Bacteria	p__Bacillota_A	c__Clostridia	o__Oscillospirales	f__Acutalibacteraceae	g__CAG-177	s__CAG-177 sp003514385
MAG1337	PD	d__Bacteria	p__Bacillota_A	c__Clostridia	o__Oscillospirales	f__Acutalibacteraceae	g__CAG-177	s__CAG-177 sp003514385
MAG3082	PD	d__Bacteria	p__Bacillota_A	c__Clostridia	o__Oscillospirales	f__Acutalibacteraceae	g__Avimonas_A	s__Avimonas_A narfia
MAG1636	PD	d__Bacteria	p__Bacillota_A	c__Clostridia	o__Oscillospirales	f__Acutalibacteraceae	g__Anaeromassilibacillus	s__Anaeromassilibacillus stercoravium
MAG1699	PD	d__Bacteria	p__Bacillota_A	c__Clostridia	o__Oscillospirales	f__Acutalibacteraceae	g__Acutalibacter	s__Acutalibacter stercoravium
MAG3056	PD	d__Bacteria	p__Bacillota_A	c__Clostridia	o__Lachnospirales	f__Lachnospiraceae	g__Wujia	s__Wujia chipingensis
MAG1137	PD	d__Bacteria	p__Bacillota_A	c__Clostridia	o__Lachnospirales	f__Lachnospiraceae	g__Wujia	s__Wujia chipingensis
MAG1289	PD	d__Bacteria	p__Bacillota_A	c__Clostridia	o__Lachnospirales	f__Lachnospiraceae	g__Wujia	s__Wujia chipingensis
MAG2580	PD	d__Bacteria	p__Bacillota_A	c__Clostridia	o__Lachnospirales	f__Lachnospiraceae	g__Wujia	s__Wujia chipingensis
MAG3357	PD	d__Bacteria	p__Bacillota_A	c__Clostridia	o__Lachnospirales	f__Lachnospiraceae	g__Wujia	s__Wujia chipingensis
MAG1219	PD	d__Bacteria	p__Bacillota_A	c__Clostridia	o__Lachnospirales	f__Lachnospiraceae	g__Wujia	s__Wujia chipingensis
MAG558	PD	d__Bacteria	p__Bacillota_A	c__Clostridia	o__Lachnospirales	f__Lachnospiraceae	g__Wujia	s__Wujia chipingensis
MAG1710	PD	d__Bacteria	p__Bacillota_A	c__Clostridia	o__Lachnospirales	f__Lachnospiraceae	g__Wujia	s__Wujia chipingensis
MAG755	PD	d__Bacteria	p__Bacillota_A	c__Clostridia	o__Lachnospirales	f__Lachnospiraceae	g__Wujia	s__Wujia chipingensis
MAG1261	PD	d__Bacteria	p__Bacillota_A	c__Clostridia	o__Lachnospirales	f__Lachnospiraceae	g__UBA11774	s__UBA11774 sp003507655
MAG1300	PD	d__Bacteria	p__Bacillota_A	c__Clostridia	o__Lachnospirales	f__Lachnospiraceae	g__UBA11774	s__UBA11774 sp003507655
MAG2144	PD	d__Bacteria	p__Bacillota_A	c__Clostridia	o__Lachnospirales	f__Lachnospiraceae	g__UBA11774	s__UBA11774 sp003507655
MAG308	PD	d__Bacteria	p__Bacillota_A	c__Clostridia	o__Lachnospirales	f__Lachnospiraceae	g__UBA11774	s__UBA11774 sp003507655
MAG2360	PD	d__Bacteria	p__Bacillota_A	c__Clostridia	o__Lachnospirales	f__Lachnospiraceae	g__UBA11774	s__UBA11774 sp003507655
MAG1988	PD	d__Bacteria	p__Bacillota_A	c__Clostridia	o__Lachnospirales	f__Lachnospiraceae	g__UBA11774	s__UBA11774 sp003507655
MAG760	PD	d__Bacteria	p__Bacillota_A	c__Clostridia	o__Lachnospirales	f__Lachnospiraceae	g__Schaedlerella	s__Schaedlerella glycyrrhizinilytica_A
MAG3309	PD	d__Bacteria	p__Bacillota_A	c__Clostridia	o__Lachnospirales	f__Lachnospiraceae	g__Lachnospira	s__Lachnospira sp000436475
MAG379	PD	d__Bacteria	p__Bacillota_A	c__Clostridia	o__Lachnospirales	f__Lachnospiraceae	g__Lachnospira	s__Lachnospira sp000436475
MAG182	PD	d__Bacteria	p__Bacillota_A	c__Clostridia	o__Lachnospirales	f__Lachnospiraceae	g__Lachnospira	s__Lachnospira sp000436475

MAG2211	PD	d__Bacteria	p__Bacillota_A	c__Clostridia	o__Lachnospirales	f__Lachnospiraceae	g__Lachnospira	s__Lachnospira sp000436475
MAG1004	PD	d__Bacteria	p__Bacillota_A	c__Clostridia	o__Lachnospirales	f__Lachnospiraceae	g__Lachnospira	s__Lachnospira sp000436475
MAG1850	PD	d__Bacteria	p__Bacillota_A	c__Clostridia	o__Lachnospirales	f__Lachnospiraceae	g__Lachnospira	s__Lachnospira sp000436475
MAG1503	PD	d__Bacteria	p__Bacillota_A	c__Clostridia	o__Lachnospirales	f__Lachnospiraceae	g__Lachnospira	s__Lachnospira rogosae
MAG628	PD	d__Bacteria	p__Bacillota_A	c__Clostridia	o__Lachnospirales	f__Lachnospiraceae	g__Lachnospira	s__Lachnospira eligens_A
MAG87	PD	d__Bacteria	p__Bacillota_A	c__Clostridia	o__Lachnospirales	f__Lachnospiraceae	g__Lachnospira	s__Lachnospira eligens_A
MAG2479	PD	d__Bacteria	p__Bacillota_A	c__Clostridia	o__Lachnospirales	f__Lachnospiraceae	g__Lachnospira	s__Lachnospira eligens_A
MAG388	PD	d__Bacteria	p__Bacillota_A	c__Clostridia	o__Lachnospirales	f__Lachnospiraceae	g__Lachnospira	s__Lachnospira eligens_A
MAG681	PD	d__Bacteria	p__Bacillota_A	c__Clostridia	o__Lachnospirales	f__Lachnospiraceae	g__Lachnospira	s__Lachnospira eligens_A
MAG1726	PD	d__Bacteria	p__Bacillota_A	c__Clostridia	o__Lachnospirales	f__Lachnospiraceae	g__Eubacterium_G	s__Eubacterium_G sp000432355
MAG1719	PD	d__Bacteria	p__Bacillota_A	c__Clostridia	o__Lachnospirales	f__Lachnospiraceae	g__Eisenbergiella	s__Eisenbergiella tayi
MAG2111	PD	d__Bacteria	p__Bacillota_A	c__Clostridia	o__Lachnospirales	f__Lachnospiraceae	g__Copromonas	s__Copromonas sp000435795
MAG1417	PD	d__Bacteria	p__Bacillota_A	c__Clostridia	o__Lachnospirales	f__Lachnospiraceae	g__Copromonas	s__Copromonas sp000435795
MAG2703	PD	d__Bacteria	p__Bacillota_A	c__Clostridia	o__Lachnospirales	f__Lachnospiraceae	g__Coprococcus_A	s__Coprococcus_A catus_A
MAG2171	PD	d__Bacteria	p__Bacillota_A	c__Clostridia	o__Lachnospirales	f__Lachnospiraceae	g__Coprococcus	s__Coprococcus eutactus_A
MAG2575	PD	d__Bacteria	p__Bacillota_A	c__Clostridia	o__Lachnospirales	f__Lachnospiraceae	g__Butyribacter	s__Butyribacter sp003529475
MAG1640	PD	d__Bacteria	p__Bacillota_A	c__Clostridia	o__Lachnospirales	f__Lachnospiraceae	g__Butyribacter	s__Butyribacter sp003529475
MAG970	Con	d__Bacteria	p__Bacillota_A	c__Clostridia	o__Lachnospirales	f__Lachnospiraceae	g__Blautia_A	s__Blautia_A wexlerae
MAG3450	Con	d__Bacteria	p__Bacillota_A	c__Clostridia	o__Lachnospirales	f__Lachnospiraceae	g__Blautia	s__Blautia sp900541955
MAG149	PD	d__Bacteria	p__Bacillota_A	c__Clostridia	o__Lachnospirales	f__Lachnospiraceae	g__Acetatifactor	s__Acetatifactor intestinalis
MAG2334	PD	d__Bacteria	p__Bacillota_A	c__Clostridia	o__Lachnospirales	f__Lachnospiraceae	g__Acetatifactor	s__Acetatifactor intestinalis
MAG175	PD	d__Bacteria	p__Bacillota_A	c__Clostridia	o__Clostridiales	f__Clostridiaceae	g__Clostridium	s__Clostridium sp900540255
MAG3225	PD	d__Bacteria	p__Bacillota_A	c__Clostridia	o__Clostridiales	f__Clostridiaceae	g__Clostridium	s__Clostridium sp001916075
MAG2833	PD	d__Bacteria	p__Bacillota_A	c__Clostridia	o__Christensenellales	f__CAG-74	g__Ventricola	s__Ventricola sp900548125
MAG1428	PD	d__Bacteria	p__Bacillota_A	c__Clostridia	o__Christensenellales	f__CAG-74	g__Ventricola	s__Ventricola sp900548125
MAG2873	PD	d__Bacteria	p__Bacillota_A	c__Clostridia	o__Christensenellales	f__CAG-74	g__Limiplasma	s__Limiplasma merdipullorum
MAG1132	PD	d__Bacteria	p__Bacillota_A	c__Clostridia	o__Christensenellales	f__CAG-74	g__Limiplasma	s__Limiplasma merdipullorum
MAG2381	PD	d__Bacteria	p__Bacillota_A	c__Clostridia	o__Christensenellales	f__CAG-74	g__Limiplasma	s__Limiplasma merdipullorum
MAG2505	PD	d__Bacteria	p__Bacillota_A	c__Clostridia	o__Christensenellales	f__CAG-74	g__Limiplasma	s__Limiplasma merdipullorum
MAG1901	PD	d__Bacteria	p__Bacillota_A	c__Clostridia	o__Christensenellales	f__CAG-74	g__Limiplasma	s__Limiplasma merdipullorum
MAG377	PD	d__Bacteria	p__Bacillota_A	c__Clostridia	o__Christensenellales	f__CAG-74	g__Fimadaptatus	s__Fimadaptatus sp900553645
MAG2488	PD	d__Bacteria	p__Bacillota_A	c__Clostridia	o__Christensenellales	f__CAG-138	g__SFEL01	s__SFEL01 sp004557245
MAG2316	PD	d__Bacteria	p__Bacillota_A	c__Clostridia	o__Christensenellales	f__CAG-138	g__SFEL01	s__SFEL01 sp004557245
MAG1537	PD	d__Bacteria	p__Bacillota_A	c__Clostridia	o__Christensenellales	f__CAG-138	g__SFEL01	s__SFEL01 sp004557245
MAG368	PD	d__Bacteria	p__Bacillota_A	c__Clostridia	o__Christensenellales	f__CAG-138	g__Phil1	s__Phil1 sp001940855
MAG543	PD	d__Bacteria	p__Bacillota_A	c__Clostridia	o__Christensenellales	f__CAG-138	g__Phil1	s__Phil1 sp001940855
MAG1934	PD	d__Bacteria	p__Bacillota_A	c__Clostridia	o__Christensenellales	f__CAG-138	g__Phil1	s__Phil1 sp001940855
MAG464	PD	d__Bacteria	p__Bacillota_A	c__Clostridia	o__Christensenellales	f__CAG-138	g__PeH17	s__PeH17 sp000435055
MAG3358	PD	d__Bacteria	p__Bacillota_A	c__Clostridia	o__Christensenellales	f__CAG-138	g__PeH17	s__PeH17 sp000435055
MAG899	PD	d__Bacteria	p__Bacillota_A	c__Clostridia	o__Christensenellales	f__CAG-138	g__PeH17	s__PeH17 sp000435055
MAG3228	PD	d__Bacteria	p__Bacillota_A	c__Clostridia	o__Christensenellales	f__CAG-138	g__PeH17	s__PeH17 sp000435055
MAG925	PD	d__Bacteria	p__Bacillota_A	c__Clostridia	o__Christensenellales	f__CAG-138	g__PeH17	s__PeH17 sp000435055
MAG2750	PD	d__Bacteria	p__Bacillota_A	c__Clostridia	o__Christensenellales	f__CAG-138	g__PeH17	s__PeH17 sp000435055
MAG1787	PD	d__Bacteria	p__Bacillota_A	c__Clostridia	o__Christensenellales	f__CAG-138	g__PeH17	s__PeH17 sp000435055
MAG3102	PD	d__Bacteria	p__Bacillota_A	c__Clostridia	o__Christensenellales	f__CAG-138	g__PeH17	s__PeH17 sp000435055
MAG669	PD	d__Bacteria	p__Bacillota_A	c__Clostridia	o__Christensenellales	f__CAG-138	g__PeH17	s__PeH17 sp000435055
MAG1896	PD	d__Bacteria	p__Bacillota_A	c__Clostridia	o__Christensenellales	f__CAG-138	g__PeH17	s__PeH17 sp000435055
MAG782	PD	d__Bacteria	p__Bacillota_A	c__Clostridia	o__Christensenellales	f__CAG-138	g__PeH17	s__PeH17 sp000435055
MAG1837	PD	d__Bacteria	p__Bacillota_A	c__Clostridia	o__Christensenellales	f__CAG-138	g__PeH17	s__PeH17 sp000435055
MAG1759	PD	d__Bacteria	p__Bacillota	c__Bacilli	o__RF39	f__UBA660	g__CAG-302	s__CAG-302 sp000431795
MAG2832	PD	d__Bacteria	p__Bacillota	c__Bacilli	o__RF39	f__UBA660	g__CAG-302	s__CAG-302 sp000431795

MAG2308	PD	d__Bacteria	p__Bacillota	c__Bacilli	o__RF39	f__UBA660	g__CAG-302	s__CAG-302 sp000431795
MAG245	PD	d__Bacteria	p__Bacillota	c__Bacilli	o__RF39	f__UBA660	g__CAG-302	s__CAG-302 sp000431795
MAG160	PD	d__Bacteria	p__Bacillota	c__Bacilli	o__RF39	f__UBA660	g__CAG-302	s__CAG-302 sp000431795
MAG2322	PD	d__Bacteria	p__Bacillota	c__Bacilli	o__RF39	f__UBA660	g__CAG-302	s__CAG-302 sp000431795
MAG151	PD	d__Bacteria	p__Bacillota	c__Bacilli	o__RF39	f__UBA660	g__CAG-302	s__CAG-302 sp000431795
MAG1859	PD	d__Bacteria	p__Bacillota	c__Bacilli	o__RF39	f__UBA660	g__CAG-302	s__CAG-302 sp000431795
MAG2270	PD	d__Bacteria	p__Bacillota	c__Bacilli	o__RF39	f__UBA660	g__CAG-302	s__CAG-302 sp000431795
MAG2607	PD	d__Bacteria	p__Bacillota	c__Bacilli	o__RF39	f__UBA660	g__CAG-302	s__CAG-302 sp000431795
MAG1709	PD	d__Bacteria	p__Bacillota	c__Bacilli	o__RF39	f__UBA660	g__CAG-302	s__CAG-302 sp000431795
MAG3229	PD	d__Bacteria	p__Bacillota	c__Bacilli	o__RF39	f__UBA660	g__CAG-302	s__CAG-302 sp000431795
MAG2343	PD	d__Bacteria	p__Bacillota	c__Bacilli	o__RF39	f__UBA660	g__CAG-302	s__CAG-302 sp000431795
MAG3208	PD	d__Bacteria	p__Bacillota	c__Bacilli	o__RF39	f__UBA660	g__CAG-302	s__CAG-302 sp000431795
MAG2702	PD	d__Bacteria	p__Bacillota	c__Bacilli	o__Lactobacillales	f__Lactobacillaceae	g__Lactobacillus	s__Lactobacillus paragasseri
MAG251	PD	d__Bacteria	p__Actinomycetota	c__Coriobacteriia	o__Coriobacteriales	f__Eggerthellaceae	g__Senegalimassilia	s__Senegalimassilia anaerobia
MAG1120	PD	d__Bacteria	p__Actinomycetota	c__Coriobacteriia	o__Coriobacteriales	f__Eggerthellaceae	g__Senegalimassilia	s__Senegalimassilia anaerobia
MAG5	PD	d__Bacteria	p__Actinomycetota	c__Coriobacteriia	o__Coriobacteriales	f__Coriobacteriaceae	g__Collinsella	s__Unclassified
MAG14	PD	d__Bacteria	p__Actinomycetota	c__Actinomycetia	o__Actinomycetales	f__Bifidobacteriaceae	g__Bifidobacterium	s__Bifidobacterium bifidum
MAG2778	PD	d__Bacteria	p__Bacteroidota	c__Bacteroidia	o__Bacteroidales	f__Muribaculaceae	g__Limisoma	s__Limisoma sp000437795
MAG2484	PD	d__Bacteria	p__Bacillota_A	c__Clostridia	o__Oscillospirales	f__Oscillospiraceae	g__Scatomorpha	s__Scatomorpha intestinigallinarum
MAG1415	PD	d__Bacteria	p__Bacillota_A	c__Clostridia	o__Christensenellales	f__CAG-138	g__SFEL01	s__SFEL01 sp004557245
MAG3444	PD	d__Bacteria	p__Bacillota_A	c__Clostridia	o__Christensenellales	f__CAG-74	g__Fimadaptatus	s__Fimadaptatus sp900553645
MAG1285	PD	d__Bacteria	p__Bacillota_A	c__Clostridia	o__Christensenellales	f__CAG-138	g__SFEL01	s__SFEL01 sp004557245
MAG226	Con	d__Bacteria	p__Bacillota_A	c__Clostridia	o__Lachnospirales	f__Lachnospiraceae	g__Coprococcus	s__Coprococcus eutactus_A
MAG3304	PD	d__Bacteria	p__Bacillota_A	c__Clostridia	o__Lachnospirales	f__Lachnospiraceae	g__Lachnospira	s__Lachnospira sp000436475
MAG364	Con	d__Bacteria	p__Bacillota_A	c__Clostridia	o__UBA1381	f__UBA1381	g__CAG-41	s__CAG-41 sp900066215
MAG2434	Con	d__Bacteria	p__Bacillota_A	c__Clostridia	o__Oscillospirales	f__Acutalibacteraceae	g__CAG-177	s__CAG-177 sp003514385
MAG1306	Con	d__Bacteria	p__Bacillota_A	c__Clostridia	o__Lachnospirales	f__Lachnospiraceae	g__UBA11774	s__UBA11774 sp003507655
MAG1886	Con	d__Bacteria	p__Bacillota_A	c__Clostridia	o__Lachnospirales	f__Lachnospiraceae	g__Blautia_A	s__Blautia_A sp900549015
MAG2897	Con	d__Bacteria	p__Bacillota_A	c__Clostridia	o__UBA1381	f__UBA1381	g__CAG-41	s__CAG-41 sp900066215
MAG751	Con	d__Bacteria	p__Bacillota_A	c__Clostridia	o__UBA1381	f__UBA1381	g__CAG-41	s__CAG-41 sp900066215
MAG2920	Con	d__Bacteria	p__Bacillota_A	c__Clostridia	o__UBA1381	f__UBA1381	g__CAG-41	s__CAG-41 sp900066215
MAG2407	PD	d__Bacteria	p__Bacillota_A	c__Clostridia	o__Clostridiales	f__Clostridiaceae	g__Clostridium	s__Clostridium sp900540255
MAG2741	Con	d__Bacteria	p__Bacillota_A	c__Clostridia	o__UBA1381	f__UBA1381	g__CAG-41	s__CAG-41 sp900066215
MAG1717	Con	d__Bacteria	p__Bacillota_A	c__Clostridia	o__Lachnospirales	f__Lachnospiraceae	g__Wujia	s__Wujia chipingensis
MAG1814	PD	d__Bacteria	p__Bacillota_A	c__Clostridia	o__Lachnospirales	f__Lachnospiraceae	g__Wujia	s__Wujia chipingensis
MAG1865	Con	d__Bacteria	p__Bacillota_A	c__Clostridia	o__UBA1381	f__UBA1381	g__CAG-41	s__CAG-41 sp900066215
MAG1943	PD	d__Bacteria	p__Bacillota_A	c__Clostridia	o__Oscillospirales	f__Oscillospiraceae	g__Vescimonas	s__Vescimonas coprocola
MAG2199	Con	d__Bacteria	p__Bacillota_A	c__Clostridia	o__Lachnospirales	f__Lachnospiraceae	g__Wujia	s__Wujia chipingensis
MAG3316	Con	d__Bacteria	p__Bacillota_A	c__Clostridia	o__UBA1381	f__UBA1381	g__CAG-41	s__CAG-41 sp900066215
MAG109	Con	d__Bacteria	p__Bacillota_A	c__Clostridia	o__Lachnospirales	f__Lachnospiraceae	g__Copromonas	s__Copromonas sp000435795
MAG3214	PD	d__Bacteria	p__Bacillota_A	c__Clostridia	o__Oscillospirales	f__Ruminococcaceae	g__Ruthenibacterium	s__Ruthenibacterium lactatiformans
MAG720	PD	d__Bacteria	p__Bacillota_A	c__Clostridia	o__Oscillospirales	f__Ruminococcaceae	g__Ruthenibacterium	s__Ruthenibacterium lactatiformans
MAG2629	PD	d__Bacteria	p__Bacillota_A	c__Clostridia	o__Oscillospirales	f__Ruminococcaceae	g__Ruthenibacterium	s__Ruthenibacterium lactatiformans
MAG1073	Con	d__Bacteria	p__Bacillota_A	c__Clostridia	o__Lachnospirales	f__Lachnospiraceae	g__Faecalimonas	s__Faecalimonas phoceensis
MAG1199	Con	d__Bacteria	p__Bacillota_A	c__Clostridia	o__Lachnospirales	f__Lachnospiraceae	g__KLE1615	s__KLE1615 sp900066985
MAG1063	PD	d__Bacteria	p__Bacillota_A	c__Clostridia	o__Oscillospirales	f__Ruminococcaceae	g__Ruthenibacterium	s__Ruthenibacterium lactatiformans
MAG256	PD	d__Bacteria	p__Bacillota_A	c__Clostridia	o__Lachnospirales	f__Lachnospiraceae	g__Dorea	s__Dorea formicigenerans
MAG2039	Con	d__Bacteria	p__Bacillota_A	c__Clostridia	o__Lachnospirales	f__Lachnospiraceae	g__Blautia_A	s__Blautia_A wexlerae
MAG1370	Con	d__Bacteria	p__Bacillota_A	c__Clostridia	o__Lachnospirales	f__Lachnospiraceae	g__Acetatifactor	s__Acetatifactor intestinalis
MAG1556	Con	d__Bacteria	p__Bacillota_A	c__Clostridia	o__Oscillospirales	f__Oscillospiraceae	g__ER4	s__ER4 sp000765235
MAG2290	Con	d__Bacteria	p__Bacillota_A	c__Clostridia	o__Lachnospirales	f__Lachnospiraceae	g__Blautia_A	s__Blautia_A wexlerae

MAG1402	Con	d__Bacteria	p__Bacillota_A	c__Clostridia	o__Lachnospirales	f__Lachnospiraceae	g__Faecalimonas	s__Faecalimonas phoceensis
MAG2695	Con	d__Bacteria	p__Bacillota_A	c__Clostridia	o__Lachnospirales	f__Lachnospiraceae	g__Blautia_A	s__Blautia_A wexlerae
MAG1910	Con	d__Bacteria	p__Bacillota_A	c__Clostridia	o__Lachnospirales	f__Lachnospiraceae	g__Faecalimonas	s__Faecalimonas phoceensis
MAG987	PD	d__Bacteria	p__Bacillota_A	c__Clostridia	o__Oscillospirales	f__Ruminococcaceae	g__Ruthenibacterium	s__Ruthenibacterium lactatiformans
MAG211	Con	d__Bacteria	p__Bacillota_A	c__Clostridia	o__Lachnospirales	f__Lachnospiraceae	g__Blautia	s__Blautia sp900541955
MAG1948	PD	d__Bacteria	p__Bacillota_A	c__Clostridia	o__Lachnospirales	f__Lachnospiraceae	g__Dorea	s__Dorea formicigenerans
MAG2361	PD	d__Bacteria	p__Bacillota_A	c__Clostridia	o__Oscillospirales	f__Oscillospiraceae	g__ER4	s__ER4 sp000765235
MAG2609	Con	d__Bacteria	p__Bacillota_A	c__Clostridia	o__Lachnospirales	f__Lachnospiraceae	g__Ruminococcus_B	s__Ruminococcus_B gnavus
MAG229	Con	d__Bacteria	p__Bacillota_A	c__Clostridia	o__Lachnospirales	f__Lachnospiraceae	g__Ruminococcus_B	s__Ruminococcus_B gnavus
MAG810	Con	d__Bacteria	p__Bacillota_A	c__Clostridia	o__Peptostreptococcales	f__Anaerovoracaceae	g__Lentihominibacter	s__Lentihominibacter sp934281605
MAG3391	Con							
MAG2402	PD							
MAG1815	PD							
MAG223	PD							
MAG1697	PD							
MAG1567	PD							
MAG144	PD							
MAG948	PD							
MAG927	PD							
MAG595	PD							
MAG3047	PD							
MAG2888	PD							
MAG922	Con							
MAG3411	PD							
MAG3232	PD							
MAG356	PD							
MAG1899	PD							
MAG3290	PD							
MAG2732	PD							
MAG2787	PD							
MAG1108	PD							
MAG2076	PD							
MAG682	PD							
MAG703	PD							
MAG2185	PD							
MAG2444	PD							
MAG2151	PD							
MAG49	PD							
MAG834	Con							
MAG1725	PD							
MAG2934	PD							
MAG459	PD							
MAG1472	Con							
MAG3480	PD							
MAG1079	PD							
MAG2509	PD							
MAG1224	PD							
MAG2875	Con							
MAG264	PD							
MAG588	PD							
MAG3410	PD							

MAG1681	PD
MAG2230	PD
MAG2664	PD
MAG1515	PD
MAG1739	PD
MAG3046	PD
MAG3389	PD
MAG3326	PD
MAG2084	PD
MAG2814	PD
MAG3083	PD
MAG3321	PD
MAG3385	PD
MAG3041	PD
MAG1913	PD
MAG51	PD
MAG696	PD
MAG2602	PD
MAG1861	PD
MAG729	PD
MAG534	PD
MAG2686	Con
MAG1644	PD
MAG2212	PD
MAG2267	PD
MAG3137	PD
MAG1454	PD
MAG1930	Con
MAG1942	Con
MAG1928	PD
MAG933	PD
MAG288	PD
MAG1688	Con
MAG2936	PD
MAG2993	Con
MAG1311	PD
MAG416	PD
MAG3322	PD
MAG1673	PD
MAG1463	Con
MAG2372	Con
MAG1646	Con
MAG1889	Con
MAG2032	Con
